# ﻿Diversity underfoot of agromyzids (Agromyzidae, Diptera) mining thalli of liverworts and hornworts

**DOI:** 10.3897/zookeys.1133.94530

**Published:** 2022-11-30

**Authors:** Makoto Kato, Luna Yamamori, Yume Imada

**Affiliations:** 1 Kyoto University Kyoto Japan; 2 Kyoto University Shirahama Japan; 3 Ehime University Matsuyama Japan

**Keywords:** Agromyzidae, bryophytivore, *Conocephalum*, epandrium, *Marchantia*, *Phytoliriomyza*, thallus-miner

## Abstract

Agromyzidae is a dipteran family that has diversified as internal plant feeders. Although most agromyzid species feed on herbaceous angiosperms, only a limited number of species has been recorded as miners of bryophytes. Extensive searches and rearing of bryophytivores in the Japanese Archipelago were made, resulting in that thallus-mining agromyzids are overwhelmingly widespread and diverse on thalloid liverworts and hornworts. By examining the morphology of adult flies, it was revealed that the agromyzid fauna comprise 39 species, of which 37 species are newly described. All the species are assigned to the genus *Phytoliriomyza* Hendel based on some shared morphological character states as follows: costa reaching M_1_; orbital setulae minute and erect (rarely proclinate); male epandrium with combs of fused tubercle-like setae and/or hypertrophied arms bearing tubercle-like setae; male distiphallus comprising a pair of stout, extended tubules; female cercus with two stout, apical, trichoid sensilla. Of the 39 agromyzid species in Japan, 36 species are associated with liverworts: 5 spp. on *Marchantia* (Marchantiaceae), 2 spp. on *Dumortiera* (Dumortieraceae), 3 spp. on *Plagiochasma*, 1 sp. on *Asterella*, 6 spp. on *Reboulia* (Aytoniaceae), 1 sp. on *Wiesnerella* (Wiesnerellaceae), 15 spp. on *Conocephalum* (Conocephalaceae), and 3 spp. on *Riccia* (Ricciaceae). Three species are associated with hornworts: 1 sp. on *Folioceros* (Anthocerotaceae), 1 sp. on *Megaceros* (Dendrocerotaceae), and 1 sp. on *Notothylas*,*Phaeoceros* (Notothyladaceae), and *Anthoceros* (Anthocerotaceae). The results suggest that 37 of the 39 species are host-specific at least to plant genus level, and that the inter-specific differences in male genitalia and color patterns of scutum, antenna, and maxillary palpus have contributed to reproductive isolation on the bryophytes that the flies share.

## ﻿Introduction

In vascular plants, a leaf represents a flat lamina borne on a shoot with abaxial-adaxial polarity, and the adaxial side is oriented towards the sun for the primary function of photosynthesis. The leaves are highly nutritious organs and thus are often consumed by various arthropods with various feeding strategies. Some insects consume specific layers of foliage while dwelling inside the internal plant tissues; such a means of herbivory is known as leaf mining (Hering 1951). The earliest known credible trace fossils of leaf mining are known from the Middle-Late Triassic (Labandeira 2006; Imada et al. 2022), but the culprits are unknown. Many leaf-mining insect clades in the megadiverse insect orders, Coleoptera, Lepidoptera, Hymenoptera and Diptera, have become diverse in the Cenozoic. For some fossil leaf mines during the Late Cretaceous, the insect culprits have been assigned to families, such as the lepidopteran Nepticulidae (Doorenweerd et al. 2015) and Lyonetiidae (Maccracken et al. 2021), and the coleopteran Buprestidae (Ding and Labandeira 2014).

The bryophytes (non-vascular plants) consist of three major clades: hornworts, liverworts, and mosses (Puttick et al. 2018). The bryophytes can be characterized by the dominance of the gametophyte phase of the life cycle where they bear photosynthetic structures (Hofmeister 1862). In mosses and some liverworts, the gametophyte is leafy in appearance; the leaves comprise one-cell thick which would prevent many insects of mining within them. However, in hornworts and some liverworts, the gametophyte is flattened to form a thallus. The thalli of thalloid bryophytes comprise multiple cell layers and can be used by internal tissue-feeders, such as thallus-miners of Rhagionidae (Imada and Kato 2016a, b) and Agromyzidae (Spencer 1990).

The Agromyzidae is the largest clades of leaf mining flies. The larvae of agromyzid flies feed on a wide variety of mostly angiospermous plants, and particularly of herbaceous plants (Spencer 1990). Recent molecular phylogenetic analyses of the agromyzids suggest that host-plant shifts repeatedly occurred during their diversification (Scheffer et al. 2007; Winkler et al. 2009). As for the thallus-miners on bryophytes, only one agromyzid species, *Phytoliriomyzamesnili* d’Aguilar, has been described from liverworts, *Riccia* L., in France (d’Aguilar 1944). In addition, larvae and pupae of undescribed thallus-mining agromyzid species have also been found in thalli of thalloid liverworts (*Monoclea* Hook) and hornworts (*Anthoceros* L.) in the Neotropics (Hering 1957, 1966), and gall-inducing larvae of an undescribed species of *Phytoliriomyza* Hendel have been recorded from a thalloid liverworts, *Monoclea*, in Peru (Ohgue et al. 2018). Although the previous records on bryophyte-feeding agromyzids are scant, diversity of agromyzids associated with thalloid liverworts and hornworts may be greatly underestimated. Much of the diversity of agromyzids associated with thalloid bryophytes, and the evolutionary history of the interactions between bryophytes and agromyzid flies remain unexplored.

*Phytoliriomyza* is a heterogenous genus whose biology is poorly known (Lonsdale 2021). Among the 116 described species, host plants were known for only 17 species that feed on liverworts (1 sp.), ferns (7 spp.), and angiosperms (9 spp.) (Spencer 1990). Due to the association with the basal lineages of plants, along with the plesiomorphic character states in larval morphology, Hering (1966) claimed that *Phytoliriomyza* was an early member of Agromyzidae. Spencer (1965) erected a new genus *Lemurimyza* for a species in Madagascar mainly based on the distinctive male genital morphology. Subsequently, however, the genus was synonymized with *Phytoliriomyza* by von Tschirnhaus (1971), mainly based on the external morphology of adults.

We have conducted an extensive taxonomic and ecological assessment of phytophagous insects on bryophytes in the Japanese archipelago. We discovered an immense diversity of agromyzid fauna on thalloid liverworts and hornworts. The morphology of the imagoes of the collected agromyzid flies is consistent with that of *Phytoliriomyza*. Herein we redefine the genus *Phytoliriomyza*; notably, diagnoses for the 39 species are provided, with descriptions of 37 new species. The recorded agromyzid species had a remarkably high morphological diversity in male genitalia and high host-specificity on bryophytes. The results unveil the diversity and host plant specificity of *Phytoliriomyza*, providing an insight into the evolutionary history of the associations with bryophytes.

## ﻿Materials and methods

### ﻿Sampling

In total, 128 thalloid bryophyte species have been recorded in the Japanese Archipelago: 111 thalloid liverwort species (2 classes, 5 orders, 19 families, 31 genera) and 17 hornwort species (1 class, 3 orders, 3 families, 6 genera) (Table 1). The explored habitats and sites ranged from natural forests to secondary vegetation, from soil to rocky substrates, from warm temperate evergreen forests to cool temperate deciduous forests, and from the Ryukyu Archipelago to northern Hokkaido; we carefully monitored stream sides, cliffs, limestone areas, and paddy fields.

**Table 1.** Thalloid liverwort and hornwort genera, with number of species of each genus and phytophagous insect species associated with the genera in Japan.

**Division**	**Class**	**Order**	**Family**	**Genus**	**No. of species**	**No. of ex- amined spp.**	** No. of bryophagous insect species**		
							**Micropterigidae**	**Rhagionidae**	**Agromyzidae**
Marchantiophyta	Jungermanniopsida	Treubiales	Treubiaceae	*Apotreubia*	1	0			
		Jungermanniales	Aneuraceae	*Aneura*	5	2			
				*Lobatiriccardia*	1	1			
				*Riccardia*	22	1			
			Metzgeriaceae	*Apometzgeria*	1	1			
				*Metzgeria*	10	2			
		Pelliales s.lat.	Pelliaceae	*Pellia*	3	2		5	
			Calyculariaceae	*Calycularia*	1	1			
			Makinoaceae	*Makinoa*	1	1			
			Fossombroniaceae	*Fossombronia*	3	1			
			Pallaviciniaceae	*Hattorianthus*	1	0			
				*Moerckia*	2	0			
				*Pallavicinia*	4	1			
	Marchantiopsida	Blasiales	Blasiaceae	*Blasia*	1	1			
				*Cavicularia*	1	1			
		Marchantiales	Marchantiaceae	*Marchantia*	6	6			5
				*Preissia*	1	1			
			Dumortieraceae	*Dumortiera*	1	1			2
			Aytoniaceae	*Plagiochasma*	2	2			3
				*Asterella*	7	3			2
				*Mannia*	4	1			
				*Reboulia*	1	1		4	6
			Cleveaceae	*Athalamia*	1	1			
				*Peltolepis*	2	0			
				*Sauteria*	3	0			
			Wiesnerellaceae	*Wiesnerella*	1	1		1	1
			Targionioideae	*Targionia*	1	1			
			Monosoleniaceae	*Monosolenium*	1	0			
			Conocephalaceae	*Conocephalum*	5	5	19	4	15
	Anthocerotae		Ricciaceae	*Riccia*	18	8			3
				*Ricciocarpos*	1	1			
			Cyathodiaceae	*Cyathodium*	1	0			
Anthocerotophyta	Anthocerotae	Anthocerotales	Anthocerotaceae	*Anthoceros*	5	1			1
				*Folioceros*	2	1			2
		Notothyladales	Notothyladaceae	*Notothylas*	3	2			1
				*Phaeoceros*	4	1			1
		Dendrocerotales	Dendrocerotaceae	*Dendroceros*	2	1			
				*Megaceros*	1	1			1
Total number of species					130	54	19	14	43 (39)*

*Because two agromyzid species are associated with plural bryophyte genera, total number of agromyzid species is 39.

At nearly 120 sites in Japan, we extensively sampled mined thalli of thalloid liverworts and hornworts during the 2000s. Collected thalli were placed in plastic cases (13.6 × 8.7 × 2.5 or 13.6 × 8.7 × 3.5 cm, “clean-cup NK”, Risu-pack, Gifu Plastic Industry Co.) and kept in incubators with temperature and light conditions maintained similar to their natural habitats. The thalli were prevented from desiccation by modestly spraying with water during incubation. We checked the plastic cases frequently for emergence of adult flies. For each emerged fly specimen, the date of thallus collection (as larva or puparium) and the date of adult emergence are recorded. Additional searches for agromyzid flies walking on thalli were also performed. Emerged and collected flies were pinned with minute pins and freeze-dried in the ice boxes of the refrigerators, dried, or fixed in 99% ethanol.

### ﻿Morphological survey

The morphology of adult agromyzid fly specimens was examined under a microscope (VHS-7000; Keyence); it was photographed by synthesizing virtual images from a sequence of corresponding depth images. The morphological characters that were important to discriminate species were color of antenna, frons, and maxillary palpus; color and pattern of scutum and scutellum; and color of haltere.

For observation of genitalia, the abdomens of fly specimens were dipped in 10% KOH for 1 day, then rinsed off with water, and dissected under a binocular microscope. Male and female genitalia of prepared specimens were photographed under a microscope (VHS-7000; Keyence) by synthesizing virtual images from a sequence of corresponding depth images. The genital morphological characters that were important to discriminate species were number and position of tubercle-like setae on male epandrium, along with the shape and sclerotization pattern of hypophallus, mesophallus, and distiphallus.

Terminology follows that outlined in Lonsdale (2021), with the fronto-orbital setae divided into the inferior orbital setae (**ori**) and the superior orbital setae (**ors**); antenna treated as scape, pedicel, 1^st^ flagellomere, and arista toward tip; and male genitalia treated as epiphallus, phallophorus, basiphallus, hypophallus, paraphallus (if present), mesophallus, and distiphallus. Wing vein terminology follows Lonsdale and von Tschirnhaus (2021). Figures of male genitalia are generally shown as ventral view of dissected genitalia.

The type specimens (holotypes and paratypes) and other materials are deposited in the National Museum of Nature and Science, Tokyo (**NMNS**) and the Kyoto University Museum (**KUM**), respectively. All the specimens were collected in Japan by M. Kato unless otherwise noted.

## ﻿Results

With rearing of phytophagous insects on 47 thalloid liverwort and 7 hornwort species (Table 1), 3096 agromyzid flies emerged; these belonged to 39 species. Among the 39 species, 36 species are associated with thalloid liverworts and three species are associated with hornworts. With the exception of two species, *Phytoliriomyzadorsata* (Siebke, 1864) and *Phytoliriomyzaalpicola* (Strobl, 1898), the remaining 37 species are new to science. Except for two species only associated, the other 37 species are host-genus specific; all species associated with liverworts are host-genus specific: five on *Marchantia*, two on *Dumortiera*, two on *Plagiochasma*, one on *Asterella*, six on *Reboulia*, one on *Wiesnerella*, 13 on *Conocephalum*, and three on *Riccia* (Table 2). Among hornwort-associated species, one species is specific to *Folioceros*, one species is specific to *Megaceros*, and one species emerged from *Phaeoceros*, *Anthoceros*, and *Notothylas*.

**Table 2.** Agromyzid species associated with thalloid liverworts and hornworts in Japan, with their host plants and distributions.

**Code**	***Phytoliriomyza*** **species**	**Host plant division**	**Host plant family**	**Host plant genus**	**Host plant species**	**No. host genera**	**No. host species**	**Distribution^1^**							
								**HK**	**HN**	**SK**	**KS**	**YK**	**AM**	**OK**	**YY**
1	*dorsata*	Marchantiophyta	Marchantiaceae	*Marchantia*	*M.polymorpha*	1	1	1							
2	*igniculus*			*Marchantia*	*M.polymorpha*	1	1	1	1						
3	*tsukuyomi*			*Marchantia*	*M.polymorpha*	1	1		1						
4	*marchantiae*			*Marchantia*	*M.paleaceapaleacea*, *M.p.diptera*, *M.polymorpha*, *M.papillatagrossibarba*	1	3	1	1	1	1				
5	*nubatama*			*Marchantia*	*M.papillatagrossibarba*, *M.emarginatacuneiloba*, *M.pinnata*	1	3		1	1	1		1	1	1
6	*dumortierae*		Dumortieraceae	*Dumortiera*	*D.hirsuta*	1	1		1	1	1	1	1		
7	*conocephali*			*Dumortiera*	*D.hirsuta*	1	1		1	1	1				
8	*arcus*		Aytoniaceae	*Plagiochasma*	*P.pterospermum*, *P.appendiculatum*	1	2		1						
9	*plagiochasmatos*			*Plagiochasma*, *Asterella*	*P.pterospermum*, *A.cruciata*	2	2		1						
10	*calcicola*			*Plagiochasma*	*P.pterospermum*	1	1		1						
11	*iriomotensis*			*Asterella*	*A.liukiuensis*	1	1								1
12	*cometiformis*			*Reboulia*	*R.hemisphaericaorientalis*	1	1		1	1	1				
13	*argentifasciata*			*Reboulia*	*R.hemisphaericaorientalis*	1	1		1	1	1	1			
14	*longifurcae*			*Reboulia*	*R.hemisphaericaorientalis*	1	1		1	1					
15	*falcata*			*Reboulia*	*R.hemisphaericaorientalis*	1	1		1						
16	*aratriformis*			*Reboulia*	*R.hemisphaericaorientalis*	1	1		1	1					
17	*rebouliae*			*Reboulia*	*R.hemisphaericaorientalis*	1	1		1	1			1		
18	*wiesnerellae*		Wiesnerellaceae	*Wiesnerella*	*W. denudata*	1	1				1				
19	*luna*		Conocephalaceae	*Conocephalum*	*C.orientalis*, *C.salebrosum*	1	2	1	1	1					
20	*izayoi*			*Conocephalum*	*C.orientalis*, *C.purpureorubrum*	1	3	1	1	1					
21	*chichibuensis*			*Conocephalum*	*C.orientalis*, *C.salebrosum*	1	2		1						
22	*caliginosa*			*Conocephalum*	*C.orientalis*	1	1		1	1	1				
23	*ugetsu*			*Conocephalum*	*C.orientalis*	1	1		1	1	1	1	1		
24	*nigroflava*			*Conocephalum*	*C.orientalis*, *C.salebrosum*	1	2	1	1						
25	*brunofasciata*			*Conocephalum*	*C.orientalis*, *C.purpureorubrum*, *C.salebrosum*	1	3		1						
26	*pallidofasciata*			*Conocephalum*	*C.orientalis*	1	1		1	1	1				
27	*luteola*			*Conocephalum*	*C.orientalis*, *C.purpureorubrum*, *C.salebrosum*	1	3	1	1						
28	*helva*			*Conocephalum*	*C.purpureorubrum*, *C.salebrosum*	1	2	1							
29	*bifasciata*			*Conocephalum*	*C.orientalis*, *C.salebrosum*	1	2	1	1	1	1				
30	*alpicola*			*Conocephalum*	*C.orientalis*, *C.salebrosum*	1	2	1	1						
31	*lanternaria*			*Conocephalum*	*C.orientalis*	1	1	1	1		1	1			
32	*conocephali*			*Conocephalum*	*C.orientalis*, *C.purpureorubrum*, *C.salebrosum*, *C.japonicum*	1	4		1	1	1				
33	*suetsugui*			*Conocephalum*	*C.orientalis*	1	1						1	1	
34	*ricciae*		Ricciaceae	*Riccia*	*Riccianipponica*, *R.miyakeana*, *R.oryzicola*, *R.bifurca*, *R.lamellosa*, *R.huebeneriana*, *R.canaliculata*	1	7		1	1	1			1	
35	*sexfasciata*			*Riccia*	*Riccialamellosa*, *R.bifurca*, *R.sorocarpa*	1	3		1						
36	*caerulescens*			*Riccia*	*R.billardieri*, *R.huebeneriana*	1	2								1
37	*foliocerotis*	Anthocerotophyta	Foliocerotaceae	*Folioceros*	*F. fuciformis*	1	1				1				
38	*megacerotis*		Megacerotaceae	*Megaceros*	*M. flagellaris*	1	1		1		1	1			
39	*phaeocerotis*		Notothyladaceae, Anthocerotaceae	*Anthoceros*, *Nototylas*, *Phaeoceros*	*Phaeoceroscarolinianus*, *Anthocerospunctatus*, *Nototylastemperata*	3	3		1	1	1		1	1	1
	Total number of species							11	32	18	17	5	6	4	4

^1^ HK, Hokkaido; HN, Honshu; SK, Shikoku; KS, Kyushu; YK, Yakushima Is.; Am, Amami Is.; OK, Okinawa Is.; YY, Yaeyama Isls.

All these species are thallus-miners; pupation takes place within their mines, except for some species mining with small thin thalli, which pupate in soil outside of the thalli. Mines of most species are linear, at least in young instars; they often enter the midrib or thicker parts of thalli in the last instar. In some species, mines are obscure from the outside. Adult fly emergence of most species occurs once in spring, while at least a few species are multivoltine.

All these flies had the following common morphological characteristics: costa extending to vein M_1_; orbital setulae minute and erect or proclinate; male epandrium each side with a row of fused long tubercle-like setae and/or one or a few well-developed tubercle-like setae (rarely lacking both); distiphallus comprising a pair of unfused sclerotized tubules; female tergite 10 trifurcate or cruciform, cercus with two stout, apical, trichoid sensilla. These characteristics suggest that all the 39 species belong to the genus *Phytoliriomyza*.

### ﻿﻿ Phytoliriomyza

Taxon classificationAnimaliaDipteraAgromyzidae

Hendel, 1931

4ED5243A-A8C6-5E0E-B190-495FA77138D7

Phytoliriomyza Hendel, 1931: 203 [as subgenus of Liriomyza]. Type species: Agromyzaperpusilla Meigen 1830: 181, by monotypy. Frick 1952: 410, 1959: 413; Spencer 1969: 201; Spencer and Steyskal 1986b: 151; Papp and Černý 2017: 313; Londsale 2021: 376.
Xyraeomyia Frick, 1952: 412. Type species: XyraeomyiaconjuctimontisFrick 1952: 413, by original designation. Spencer 1965 [synonymy].
Pteridomyza Nowakowski, 1962: 97. Type species: Agromyzahilarella Zetterstedt 1848: 2776, by original designation. von Tschirnhaus 1971 [synonymy]. Manual of North American Agromyzidae 377.
Lemurimyza Spencer, 1965: 26. Type species: LiriomyzaenormisSpencer 1963c: 114, by original designation. von Tschirnhaus 1971 [synonymy].
Nesomyza Spencer in Spencer and Stegmaier 1973: 190. Type species: Nesomyzafusculoides Spencer in Spencer and Stegmaier 1973: 190, by original designation. Spencer 1973 [synonymy].

#### Diagnosis.

***Head*:** Head yellow, frons often with pruinosity. Orbital plates more or less emerge from the plane of frons. Front orbitals three pairs; one ori directed inward and two ors directed up. 1–2(3) pairs of medio-clinate lower orbital setae, two pairs of reclinate upper orbital setae. Orbital setulae proclinate, upright or partly reclinate. Postocellar and ocellar setae well developed. First flagellomere round.

***Thorax*:** Scutum with 1+3 dorsocentrals. Acrostichal setulae in two rows, but lacking in some species. Postpronotal, two propleural, presutural, and propleural setae normal or strong. Anepisternum with a long posterior seta and with two or three shorter posterior setae. Katepisternum with one or two long setae. Legs simple, only ventroapical seta on middle tibia present. Costal vein ends at apex of M_1_. The ratio of ultimate and penultimate sections of M_4_ various.

***Abdomen*:** Male abdomen lacks stridulatory organs.

***Male genitalia*:** Epandrium round apically, often with a comb of several fused tubercle-like setae and/or elongated tubercle-like setae on the inner surface; surstylus setigerous and sometimes with one or a few tubercle-like setae. Subepandrial sclerite short but very broad, connecting bases of surstyli through a special sclerite. Hypandrium very long but very thin. Hypophallus membranous often with a pair of parallel sclerites medially. Paraphallus present or absent. Mesophallus cylindrical and well sclerotized. Distiphallus with basal part formed by usually a paired sclerite and an apical part, which terminates in paired tubes or in extremely long less sclerotized tubules. Ejaculatory apodeme variable, but basal part usually broad and blade not too large.

***Female postabdomen*:** Tergite 10 trifurcate or cruciform posteriorly. Cercus with two stout, apical, trichoid sensilla.

##### ﻿Classification of *Phytoliriomyza* species associated with bryophytes

The morphological characteristics of the species associated with bryophytes coincided with the characteristics unique to the genus *Phytoliriomyza*. In addition to these characteristics of adult males, we found characteristics of adult females unique to the bryophyte-associated species, in which tergite 10 was trifurcate or cruciform and each cercus bears two stout, apical, trichoid sensilla.

All the species associated with bryophytes are distinguished by color of antenna, color of maxillary palpus, color pattern of scutum and morphology of male genitalia. Colors of 1^st^ flagellomere of antenna and maxillary palpus are dark or yellow, and unique to each species. Scutum was yellow or dark, and the yellow scutum often had three pairs of dark longitudinal stripes: medial stripes on anterior 2/3 (always merged together except in one species), intra-alar stripes (inner lateral stripes) and supra-alar stripes (outer lateral stripes) on anterior 4/5. In some species, the intra-alar stripe and the supra-alar stripe are merged together to shape a wide band, and often merged with the medial stripe. The color pattern of scutum is unique to each species at least among the species sharing the same host bryophyte genus. Although some species are very similar in external morphology, they could be clearly distinguished by the number of tubercle-like setae in a comb on the male epandrium and other male genital morphological characters.

The 39 recorded *Phytoliriomyza* species can be classified into four groups (*phaeocerotis* group, *mesnili* group, *alpicola* group, and *dorsata* group) based on presumed synapomorphy of the following characteristics: distribution of acrostichal setulae on scutum, morphology of epandrium and distiphallus of male genitalia, and color of legs. By assessing the morphological characteristics of the *Phytoliriomyza* species (Table 3), we obtained a key to species as follows.

**Table 3.** A synopsis of morphological characteristics of Japanese bryophyte-associated *Phytoliriomyza* species.

**Group**	***Phytoliriomyza*** ** species**	**Color of 1st flagellomere**	**Color of maxillary pulpus**	**Wing length (mm)**	**No. pairs of acro-stichal setulae**	**Color of scutum**	**Color of scutellum**	**Color of leg**	**Medial, inter-alar and supra-alar stripes^1^**	**color of haltere**	**Surstylus**	**No. tubercle-like setae of comb on epan-drium^2^**	**No. hyper-trohied arm on epan-drium**	**No. tubercle-like setae on surstylus**	**Distal end of disti-phalus**	**Length of disti-phalus**	**Host plant genus**
*phaeocerotis*-G	*megacerotis*	dark	dark	1.2–1.4	2–3	dark	dark	dark	0-0-0	dark	lobate	6	0	6	tapering out	short	*Megaceros*
	*foliocerotis*	yellow	yellow	1.1–1.3	3–4	dark	partly yellow	yellow	0-0-0	yellow	lobate	-	0	0	tapering out	long	*Folioceros*
	*phaeocerotis*	dark	yellow	1.2–1.5	0	dark	dark	yellow	0-0-0	dark	lobate	-	0	0	tapering out	long	*Phaeoceros*, *Nothotylas*, *Anthoceros*
*mesnili*-G	*caerulescens*	dark	yellow	1.1–1.3	3–4	dark	dark	dark	0-0-0	dark	elongated	-	0	0	tapering out	short	*Riccia*
	*ricciae*	dark	dark	1.0–1.3	0–2	dark	dark	yellow	1-1-1	dark	elongated	(2)	0	2	tapering out	short	*Riccia*
	*sexfasciata*	dark	yellow	1.2–1.5	1–3	dark	dark	dark	0-0-0	dark	elongated	(40)	0	0	tapering out	short	*Riccia*
	*iriomotensis*	dark	yellow	1.4–1.7	0	dark	dark	dark	0-0-0	dark	elongated	(3)	0	1	tapering out	short	*Asterella*
*alpicola*-G	*tsukuyomi*	dark	dark	1.6–1.7	6–7	dark	yellow	dark	0-0-0	dark	lobate	4	2	1	truncated	short	*Marchantia*
	*alpicola*	dark	dark	1.7–1.8	5–6	dark	yellow	dark	0-0-0	dark	lobate	6	0	1	truncated	short	*Conocephalum*
	*marchantiae*	dark	dark	1.6–1.8	5–6	dark	yellow	dark	0-0-0	dark	lobate	8	0	0	truncated	short	*Marchantia*
	*rebouliae*	dark	dark	1.3–1.7	6–7	dark	yellow	dark	0-0-0	dark	lobate	7	0	0	truncated	short	*Reboulia*
	*conocephali*	dark	dark	1.3–1.7	5–6	dark	yellow	dark	0-0-0	dark	lobate	5–6	0	2	truncated	short	*Conocephalum*
	*lanternaria*	dark	dark	1.8–1.9	5–6	dark	yellow	dark	0-0-0	dark	lobate	7	0	1	truncated	short	*Conocephalum*
	*suetsugui*	dark	dark	1.3–1.5	5–6	dark	dark	dark	0-0-0	dark	lobate	6	0	1	truncated	short	*Conocephalum*
	*dumortierae*	dark	dark	1.9–2.2	7–8	dark	yellow	dark	0-0-0	yellow	lobate	2	0	1	truncated	short	*Dumortiera*
	*wiesnerellae*	dark	dark	2.0–2.3	7	dark	yellow	dark	0-0-0	yellow	lobate	(1)	0	2	truncated	short	*Wiesnerella*
	*nubatama*	dark	yellow	1.3–1.6	5	dark	yellow	dark	0-0-0	yellow	lobate	7	0	3	truncated	short	*Marchantia*
*dorsata*-G	*ugetsu*	dark	yellow	2.1–2.7	10–12	dark	dark	yellow	0-0-0	dark	lobate	6	0	1	truncated	short	*Conocephalum*
	*luteola*	dark	yellow	1.9–2.0	8–9	yellow	yellow	yellow	0-0-0	yellow	lobate	3–4	0	1	truncated	short	*Conocephalum*
	*helva*	yellow	yellow	1.8–2.1	5–6	yellow	yellow	yellow	0-0-0	yellow	lobate	3	0	1	truncated	short	*Conocephalum*
	*pallidofasciata*	dark	yellow	1.9–2.0	8–9	yellow	yellow	yellow	0-1-1	yellow	lobate	4–5	0	1	truncated	short	*Conocephalum*
	*argentifasciata*	yellow	yellow	1.5–1.9	5	yellow	yellow	yellow	1-1-1	yellow	lobate	4–5	0	1	truncated	short	*Reboulia*
	*longifurcae*	yellow	yellow	1.5–1.6	5	yellow	yellow	yellow	1-1-1	yellow	lobate	6	1	1	truncated	short	*Reboulia*
	*calcicola*	yellow	yellow	1.6–1.7	7	yellow	yellow	yellow	1-1-1	yellow	lobate	6	1	1	truncated	short	*Plagiochasma*
	*brunofasciata*	dark	yellow	1.9–2.2	7–8	yellow	yellow	yellow	1-1-1	yellow	lobate	5–7	0	1	truncated	short	*Conocephalum*
	*dorsata*	dark	yellow	1.7–1.8	8–9	yellow	yellow	yellow	1-1-1	yellow	lobate	6	0	2	truncated	short	*Marchantia*
	*nigroflava*	yellow/dark	yellow	2.2–2.3	8–10	yellow	yellow	yellow	1-1-1	yellow	lobate	5–7	0	1	truncated	short	*Conocephalum*
	*bifasciata*	dark	yellow	2.2–2.3	22–26*	yellow	yellow	yellow	1-1-0	yellow	lobate	3–4	1	1	truncated	short	*Conocephalum*
	*cometiformis*	dark	yellow	2.0–2.2	8	yellow	yellow	yellow	1-1/1	yellow	lobate	3+3	2	1	truncated	short	*Reboulia*
	*luna*	dark	yellow	2.7–2.9	8–10	yellow	yellow	yellow	1-1/1	yellow	lobate	7–8	0	1	truncated	short	*Conocephalum*
	*izayoi*	dark	yellow	2.4–2.5	7–9	yellow	yellow	yellow	1-1/1	yellow	lobate	9–12	0	1	truncated	short	*Conocephalum*
	*chichibuensis*	dark	yellow	2.2–2.9	8–9	yellow	yellow	yellow	1/1/1	yellow	lobate	6	0	1	truncated	short	*Conocephalum*
	*caliginosa*	dark	yellow	2.1–2.3	8–9	yellow	yellow	yellow	1/1/1	yellow	lobate	8	0	1	truncated	short	*Conocephalum*
	*igniculus*	dark	yellow	2.0–2.1	6–7	yellow	yellow	yellow	1/1/1	yellow	lobate	5	0	1	truncated	short	*Marchantia*
	*crepusculum*	dark	yellow	1.4–1.8	5	yellow	yellow	yellow	1/1/1	dark	lobate	5	0	0	truncated	short	*Dumortiera*
	*arcus*	yellow	yellow	1.3–1.6	6	yellow	yellow	yellow	1/1/1	yellow	elongated	(2)	1	1	truncated	short	*Plagiochasma*
	*plagiochasmatos*	yellow	yellow	1.4–1.5	6	yellow	yellow	yellow	1/1/1	yellow	elongated	(2)	0	1	truncated	short	*Plagiochasma*, *Asterella*
	*falcata*	yellow	yellow	1.6–2.0	6–8	yellow	yellow	yellow	1/1/1	yellow	elongated	(2)	1	1	truncated	short	*Reboulia*
	*aratriformis*	yellow	yellow	1.9–2.3	7–8	yellow	yellow	yellow	1/1/1	yellow	elongated	(2)	1	1	truncated	short	*Reboulia*

^1^ 1, present; 0, absent; 1-1, stripes unfused; 1/1, stripes fused.<br/> ^2^ Number of unfused tubercle-like setae forming a row is shown in parenthesis; -, lacking tubercle-like setae forming a row.<br/> ^*^ Number of setulae in four rows.

##### ﻿Key to the Japanese *Phytoliriomyza*

**Table:** 

1	Acrostichal setulae vestigial, at most 4 pairs in 2 rows (Fig. 74D); male epandrium with convex inner ventral margin (Fig. 74I); distal end of distiphallus tapering out (Fig. 74G); small species with wing length ranging from 1.1 to 1.8 mm	**2**
–	Acrostichal setulae at least 5 pairs in 2 (or rarely 4) rows (Fig. 1D); male epandrium with concave inner ventral margin (Fig. 1J); distal end of distiphallus truncated (Fig. 1H); small to large species with wing length ranging from 1.3 and 2.9 mm	**8**
2	Surstylus small, lobate, not elongated, not setose apically (Fig. 74I)	**3** **[** ***phaeocerotis*** **-group: associated with hornworts]**
–	Surstylus large, elongated, setose apically (Fig. 63K)	**5** **[** ***mesnili*** **-group: associated with** ***Riccia*** ***and*** ***Asterella*** **]**
3	Legs dark brown (Fig. 72A); epandrium with a comb of tubercle-like setae (Fig. 72H); distiphallus shorter than phallapodeme (Fig. 72G)	***Phytoliriomyzamegacerotis*** **[host:** ***Megaceros*** **]**
–	Legs yellow or brownish yellow (Fig. 74A); epandrium without a comb of tubercle-like setae (Fig. 74I); distiphallus longer than phallapodeme (Fig. 74G)	**4**
4	1^st^ flagellomere and haltere yellow (Fig. 70B); scutellum dark brown, medially with a yellow patch (Fig. 70D)	***P.foliocerotis*** **[host:** ***Folioceros*** **]**
–	1^st^ flagellomere and haltere dark (Fig. 74B); scutellum wholly gray (Fig. 74D)	***P.phaeocerotis*** **[host:** ***Phaeoceros*** **,** ***Notothylas*** **,** ***Anthoceros*** **]**
5	Maxillary palpus dark-colored (Fig. 63C); legs yellow (Fig. 63A); scutum yellowish gray with dark intra-alar and supra-alar stripes (Fig. 63D)	***P.ricciae*** **[host:** ***Riccia*** **]**
–	Maxillary palpus yellow (Fig. 68F); legs dark-colored (Fig. 68A); scutum completely gray or gray with longitudinal dark stripes (Fig. 66I)	**6**
6	Scutum completely gray (Fig. 21D); acrostichal setula absent (Fig. 21D)	***P.iriomotensis*** **[host:** ***Asterella*** **]**
–	Scutum bluish gray with longitudinal dark stripes (Fig. 66I); acrostichal setulae 1–4 pairs in two rows (Fig. 66I)	**7**
7	Scutum with 3 pairs of longitudinal dark gray stripes (Fig. 66I); epandrium with a dense cluster of short tubercle-like setae at inner margin (Fig. 66L)	***P.sexfasciata*** **[host:** ***Riccia*** **]**
–	Scutum with a medial stripe and a pair of lateral longitudinal dark gray stripes (Fig. 68I); epandrium with 3 strong tubercle-like setae at inner margin (Fig. 68N)	***P.caerulescens*** **[host:** ***Riccia*** **]**
8	Legs dark-colored (Fig. 56B); scutum uniformly dark-colored (Fig. 56 D)	**9** **[** ***alpicola*** ** group]**
–	Legs yellow (Fig. 1B); scutum yellow with or without longitudinal lateral stripes (Fig. 1D); if scutum dark-colored, arista yellow (Fig. 44D, F)	**18** **[** ***dorsata*** ** group] **
9	Haltere yellow (Fig. 10E)	**10**
–	Haltere dark-colored (Fig. 56B)	**12**
10	Maxillary palpus yellow (Fig. 8C); wing length < 2 mm	***P.nubatama*** **[host:** ***Marchantia*** **]**
–	Maxillary palpus dark-colored (Fig. 10B); wing length > 2 mm	**11**
11	Pleuron with ventral half dark-colored (Fig. 10B); surstylus with 1 tubercle-like seta (Fig. 10K)	***P.dumortierae*** **[host:** ***Dumortiera*** **]**
–	Pleuron wholly yellow (Fig. 35B); surstylus with 2 tubercle-like setae (Fig. 35I)	***P.wiesnerellae*** **[host:** ***Wiesnerella*** **] **
12	Scutellum wholly gray (Fig. 62D)	***P.suetsugui*** **[host:** ***Conocephalum*** **]**
–	Scutellum wholly yellow or at least partly yellow (Fig. 56B)	**13**
13	Pedicel of antenna yellow (Fig. 5C); scutum completely dark gray (Fig. 5D); male surstylus basally with a hypertrophied columnar arm, bearing tubercle-like seta apically (Fig. 5I)	***P.tsukuyomi*** **[host: ** ***Marchantia*** **]**
–	Pedicel of antenna brown (Fig. 56C); scutum dark gray except for posterior margin or mid-posterior area (Figs 56D, 58D); male surstylus lacking a hypertrophied columnar arm (Fig. 5I)	**14**
14	Legs dark brown (Fig. 56B); Scutum dark gray with posterior margin yellow (Fig. 56D)	***P.alpicola*** **[host:** ***Conocephalum*** **] **
–	Legs yellowish brown (Fig. 58B); scutum dark gray with a semicircular yellow patch at mid posterior area (Fig. 58D)	**15**
15	Male surstylus lacking tubercle-like seta (Fig. 6I); epandrium lacking an anteriorly directed tubercle-like seta on inner-lateral surface (Fig. 6I)	**16**
–	Male surstylus with 1 or 2 tubercle-like setae (Fig. 59K); epandrium with an anteriorly directed tubercle-like seta on inner-lateral surface (Fig. 59K)	**17**
16	Male epandrium with a comb comprising 8 basally fused, long, tubercle-like setae (Fig. 6I); epandrium with one tubercle-like seta at inner-lateral margin (Fig. 6G)	***P.marchantiae*** **[host:** ***Marchantia*** **]**
–	Male epandrium with a comb comprising 7 basally fused, long, tubercle-like setae (Fig. 33J); epandrium with 1 tubercle-like seta at inner-posterior margin (Fig. 33H)	***P.rebouliae*** **[host:** ***Reboulia*** **]**
17	Male epandrium with a comb comprising 5 or 6 basally fused, long, tubercle-like setae (Fig. 59I); Surstylus with 2 tubercle-like setae (Fig. 59K)	***P.conocephali*** **[host:** ***Conocephalum*** **]**
–	Male epandrium with a comb comprising 7 basally fused long tubercle-like setae (Fig. 58I); surstylus with 1 tubercle-like seta (Fig. 58I)	***P.lanternaria*** **[host:** ***Conocephalum*** **]**
18	Scutum and scutellum gray (Fig. 44D); arista yellow (Fig. 44F)	***P.ugetsu*** **[host:** ***Conocephalum*** **]**
–	Scutum yellow at least in part, and scutellum yellow (Fig. 44D); arista black (Fig. 44F)	**19**
19	Scutum wholly yellow, lacking dark stripes (Fig. 49D)	**20**
–	Scutum yellow, with 1 or 2 pairs of dark longitudinal stripes (Fig. 49D)	**21**
20	1^st^ flagellomere black (Fig. 51B); tubercle-like seta on inner surface of epandrium 1.5 × as long as the seta of a comb (Fig. 51I)	***P.luteola*** **[host:** ***Conocephalum*** **]**
–	1^st^ flagellomere yellow (Fig. 52B); tubercle-like seta on inner surface of epandrium as long as the seta of a comb (Fig. 52I)	***P.helva*** **[host:** ***Conocephalum*** **]**
21	Scutum lacking a medial dark stripe, but with brown unfused intra-alar and supra-alar stripes (Fig. 49D)	***P.pallidofasciata*** **[host:** ***Conocephalum*** **]**
–	Scutum with a medial dark stripe (Fig. 1D)	**22**
22	Intra-alar and supra-alar stripes of scutum separate and unfused (Fig. 1D)	**23**
–	Intra-alar and supra-alar stripes of scutum fused, forming a pair of wide bands (Fig. 3D)	**29**
23	Scutum subglossy with pruinosity; intra-alar and supra-alar stipes with similar thickness (Fig. 46B); acrostichal setulae in 2 rows (Fig. 46B)	**24**
–	Scutum glossy; supra-alar stipe is pale and indistinct (Fig. 54D); acrostichal setulae in 4 rows (Fig. 54D)	***P.bifasciata*** **[host:** ***Conocephalum*** **]**
24	Intra-alar stripes not confluent with a pair of lateral presutural dark ovoid spots (Fig. 46B); 1^st^ flagellomere yellow in male, black in female (Fig. 46A, D)	***P.nigroflava*** **[host:** ***Conocephalum*** **]**
–	Intra-alar stripes adjoining and confluent with 1 pair of lateral presutural dark ovoid spots (Fig. 1D); 1^st^ flagellomere yellow or black, same in both sexes	**25**
25	1^st^ flagellomere yellow (Fig. 24B)	**26**
–	1^st^ flagellomere black (Fig. 1B)	**28**
26	Intra-alar stripes and lateral presutural dark ovoid spots with silvery pruinosity (Fig. 24D); male epandrium with a comb comprising 4 or 5 tubercle-like setae (Fig. 24H	***P.argentifasciata*** **[host:** ***Reboulia*** **]**
–	Intra-alar stripes and lateral presutural dark ovoid spots without silvery pruinosity (Fig. 27D); male epandrium with a comb comprising 6 tubercle-like setae (Fig. 27I)	**27**
27	Male epandrium with an extremely elongated arm, bearing 2, dark, ventrally curved, tubercle-like setae borne at wide angle (Fig. 27H); surstylus lobate, setose apically, with 1 short tubercle-like seta subapically (Fig. 27K)	***P.longifurcae*** **[host:** ***Reboulia*** **]**
–	Male epandrium with a basally enlarged, slightly flattened hypertrophied arm, which bears a dark laterally enlarged tubercle-like seta (Fig. 18G); surstylus narrow, bare, with 1 long tubercle-like seta apically (Fig. 18G)	***P.calcicola*** **[host: ** ***Plagiochasma*** **]**
28	Scutellum yellow with lateral margins dark-colored (Fig. 1D); surstylus with 2 tubercle-like setae (Fig. 1J)	***P.dorsata*** **[host:** ***Marchantia*** **]**
–	Scutellum wholly yellow (Fig. 48D); surstylus with 1 tubercle-like seta (Fig. 48I)	***P.brunofasciata*** **[host:** ***Conocephalum*** **]**
29	1^st^ flagellomere black (Fig. 37C); male epandrium with a comb of tubercle-like setae (Fig. 37I)	**30**
–	1^st^ flagellomere yellow (Fig. 14B); male epandrium lacking a comb of tubercle-like setae (Fig. 14K)	**36 **
30	Scutellum brownish yellow, tergite of abdomen brown (Fig. 13D); haltere brown (Fig. 13D); wing length < 1.8 mm	***P.crepusculum*** **[host:** ***Dumortiera*** **]**
–	Scutellum yellow, tergite of abdomen yellow (Fig. 47D); haltere yellow (Fig. 47D); wing length > 1.9 mm	**31**
31	Lateral fused band of scutum reaching just before scutellum (Fig. 22D); male epandrium with a comb comprising 3 or 4 fused long tubercle-like setae, and with an enlarged arm bearing a strong tubercle-like seta (Fig. 22J)	***P.cometiformis*** **[host: ** ***Reboulia*** **]**
–	Lateral fused band of scutum ending at anterior 7/8 length of scutum before scutellum (Fig. 37D); male epandrium with a comb comprising 7–12 fused long tubercle-like setae (Fig. 37I)	**32**
32	Medial stripe of scutum not confluent with the lateral fused band, and the 2 bands are delimited by a yellow line (Fig. 37D); yellow patch ranging from posterior scutum to scutellum well-defined (Fig. 37D); male epandrium with 1 pair of tubercle setae at posterior end on inner margin (Fig. 37G)	**33**
–	Medial stripe of scutum confluent with the lateral fused band (Fig. 43D); yellow patch ranging from posterior scutum to scutellum oblong and ill-defined (Fig. 43D); male epandrium with 1 pair of tubercle setae on subposterior inner margin (Fig. 43G)	**34**
33	Scutellum wholly yellow (Fig. 37D); male epandrium with a comb comprising 7 or 8 fused tubercle-like setae (Fig. 37I)	***P.luna*** **[host:** ***Conocephalum*** **]**
–	Scutellum yellow with lateral margins darkened (Fig. 40D); male epandrium with a comb comprising 9–12 fused tubercle-like setae (Fig. 40J)	***P.izayoi*** **[host:** ***Conocephalum*** **]**
34	Abdomen dorsally with a pair of brown lateral semicircular patches on anterior half of the 2^nd^ tergite (Fig. 3E); male epandrium with a comb comprising 5 fused tubercle-like setae (Fig. 3I)	***P.igniculus*** **[host:** ***Marchantia*** **]**
–	Abdomen dorsally without dark patches on tergites (Fig. 42B); male epandrium with a comb comprising 6–8 fused tubercle-like setae (Fig. 42J)	**35**
35	Male epandrium with a comb comprising 6 fused tubercle-like setae (Fig. 42J)	***P.chichibuensis*** **[host:** ***Conocephalum*** **]**
–	Male epandrium with a comb comprising 8 fused tubercle-like setae (Fig. 43I)	***P.caliginosa*** **[host:** ***Conocephalum*** **]**
36	Yellow patch of midposterior scutum darkened and ill-defined (Fig. 32D); Tergite of abdomen brownish yellow (Fig. 32E)	***P.aratriformis*** **[host:** ***Conocephalum*** **]**
–	Yellow patch of midposterior scutum rectangular and well-defined (Fig. 14D); Tergite of abdomen yellow (Fig. 14E)	**37 **
37	Male epandrium with a pair of elongated, hypertrophied arms, each bearing a tubercle-like seta apically (Fig. 14K)	**38**
–	Male epandrium without a pair of elongated, hypertrophied arms (Fig. 17I)	***P.plagiochasmatos*** **[host:** ***Plagiochasma*** **]**
38	Hypertrophied arm on male epandrium strongly curved outward (Fig. 14K)	***P.arcus*** **[host:** ***Plagiochasma*** **]**
–	Hypertrophied arm on male epandrium protruded forward (Fig. 29I)	***P.falcata*** **[host:** ***Reboulia*** **]**

##### ﻿Taxonomy

We describe 39 *Phytoliriomyza* species in order of phylogenetic position of their host plants (as shown in Tables 1 and 2), focusing on morphological characteristics, geographical variation, host-plant records, and life history of each species.

###### Species associated with *Marchantia*

### ﻿1.﻿ Phytoliriomyza dorsata

Taxon classificationAnimaliaDipteraAgromyzidae

Siebke, 1864

5B469994-271C-5909-8298-62F00FE0BA49

Fig. 1

Agromyza dorsata Siebke, 1864: 169.
Liriomyza reverberata . Frick 1952: 375; 1959: 409.
Lemurimyza dorsata . Spencer 1965: 28, 1969: 194.
Phytoliriomyza dorsata . Spencer 1976: 294; Spencer and Steyskal 1986: 303; Papp and Černý 2017: 317; Lonsdale 2021: 398; Černý et al. 2020: 214.

#### Material examined.

Japan: 1♂1♀ (MK-AG-a410, a26), Namari-kawa, Yakumo, Futami, Hokkaido (42.201151°N, 140.135658°E, 145 m asl), 10-VI-2012 (as larva), emerged on 13–17-VI-2012, NMNS.

#### Diagnosis.

 A medium-sized yellow species (wing length 1.7–1.8 mm) that has a pruinose yellow scutum with one medial and two pairs of gray lateral stripes, black 1^st^ flagellomeres, yellow maxillary palpi, yellow halteres, and yellow legs. Male epandrium inner-laterally with a comb consisting of six fused long tubercle-like setae, and surstylus with two tubercle-like setae. Larva mines the thallus of *Marchantiapolymorpha* L.

#### Description.

**Figure 1.**
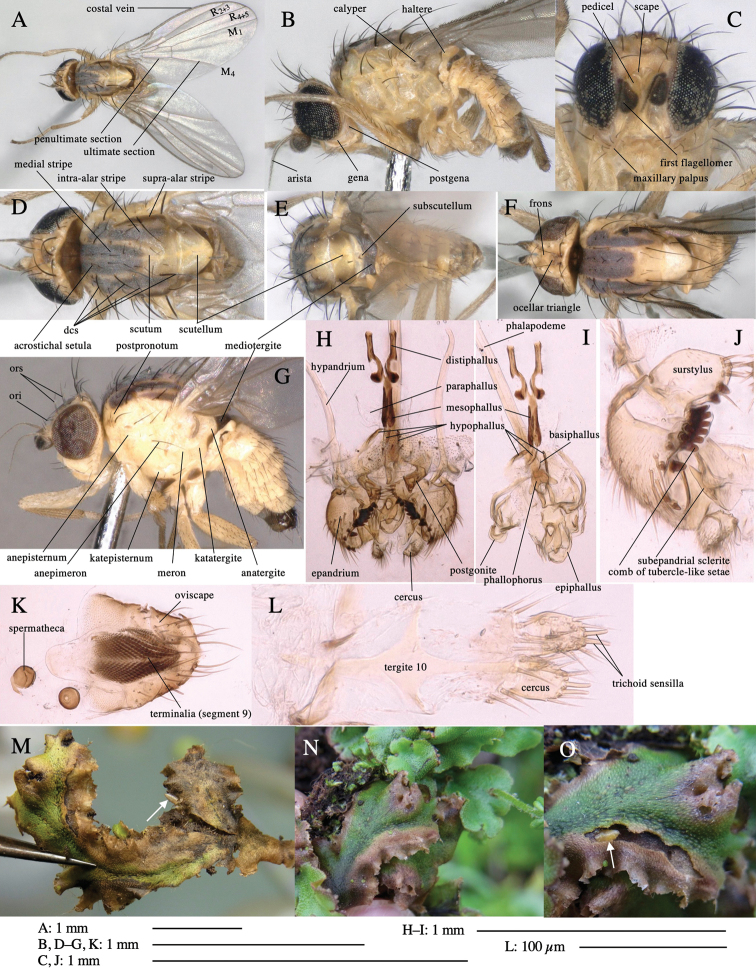
*Phytoliriomyzadorsata***A–E** male at Yakumo **A** habitus **B** lateral body **C** frontal head **D** dorsal thorax **E** thorax in posterior view **F, G **female at Yakumo **F **dorsal thorax **G** lateral body **H–J** male genitalia **H** whole genitalia, ventral **I** phallic complex, ventral **J** epandrium, ventral **K, L** female postabdomen **K** oviscape and spermatheca **L** tergite 10 **M–O** mined thalli of *Marchantiapolymorpha***M** mined thallus dissected to show interior and puparium (arrow) **N** mined thallus with exit hole **O** the same thallus with the mine dissected to show a puparium (arrow).

**Adult male** (Fig. 1A–E).

***Head*:** Head light yellow, with ocellar tubercle brown, back of head dark brown (Fig. 1A). Lunule distinct and slightly sunken. Antenna porrect, first flagellomere black, pedicel and scape yellow. Arista subbasal, black pubescent. Eye upright, bare. Face slightly convex. Gena straight. Clypeus, face, and postgena yellow. Proboscis normal, yellow; palpus yellow, cylindrical. ***Chaetotaxy*:** Front orbitals three pairs; one ori directed inward; two ors directed upward (Fig. 1B). Orbital setulae minute and erect, in a single row.

***Thorax*:** Thorax pruinose. Scutum yellow with medial dark stripe on anterior 2/3, with a pair of wide dark intra-alar stripes and a pair of narrower dark supra-alar stripes, adjoining a pair of lateral presutural dark ovoid spots (Fig. 1D). Scutellum yellow with lateral corner brown. Subscutellum with anterior half yellow, ventral half brown. Mediotergite brown, anatergite and katatergite yellow. Pleuron largely yellow, venters of katepisternum and meron brown. Haltere yellow. Calypter margin and hairs gray. Leg segments yellow; tibiae and tarsi darker. ***Chaetotaxy*:** Scutum with 1+3 dorsocentrals, shortened in length anteriorly (Fig. 1D). Acrostichal setulae eight or nine pairs in two rows. ***Wing*:** Wing length 1.7 mm, costa reaching M_1_ (Fig. 1A). Length of ultimate section of vein M_4_ divided by penultimate section 1.7.

***Abdomen*:** Abdomen dorsally subshiny yellow; epandrium dark brown (Fig. 1B). ***Genitalia*:** (Fig. 1H–J) Epandrium rounded apically, with a short tubercle-like seta on inner-middle surface, a comb comprising six long fused tubercle-like setae on inner-anterior surface, a few short tubercle-like setae immediately outward of the comb; and a row of minute tubercle-like setae on ventral inner margin (Fig. 1J). Surstylus rounded, setose apically, with two short tubercle-like setae posteriorly. Cercus narrow, setose. Subepandrial sclerite with one pair of medial flat, plate-like ventral processes and hook-shaped protrusion directing anteriorly (Fig. 1J). Hypandrium slightly sclerotized along outer margin (Fig. 1H). Postgonite bare and broadly rounded apically (Fig. 1I). Phallophorus with deep incision below, articulated with phallapodeme, fused to epiphallus (Fig. 1I). Basiphallus with a narrow plate on left side (Fig. 1I). Hypophallus membranous with a pair of small, parallel sclerites medially (Fig. 1I). Paraphallus broad membranous, with pointed, lightly sclerotized margins; paraphalli diverging, angled anteroventrally, jointed basally (Fig. 1H). Mesophallus dark, cylindrical, widest subbasally, length similar to that of distiphallus. Distiphallus comprising one pair of stout tubules; basal half composed of dorsal bulbous dark sclerites and weaker medial region; distal half cylindrical, dorsally and laterally pigmented (Fig. 1H).

**Female** (Fig. 1F, G). Similar to male, but slightly larger and frons wider. Wing length 1.8 mm. ***Postabdomen*:** (Fig. 1K, L) Oviscape dark brown, setigerous (Fig. 1K). Tergite 10 cruciform, laterally uniting narrow pleural sclerites (Fig. 1L). Sternite bearing four pairs of marginal setae. Each cercus with two stout, apical, trichoid sensilla, 1/3 length of cercus (Fig. 1L). Spermathecae orbicular (Fig. 1K).

#### Japanese name.

Usuobi-zenigoke-hamoguribae.

#### Host plant.

*Marchantiapolymorpha* (Marchantiaceae).

#### Mine.

(Fig. 1M–O) Larvae construct linear mines in the thallus of liverwort in early instars, later entering the midrib, and pupating there, while mines are obscure from outside.

#### Biological notes.

 The host plants from which this species emerged grow on wet cliffs along roads in a cool temperate forest of *Quercuscrispula*.

#### Distribution.

**Figure 2.**
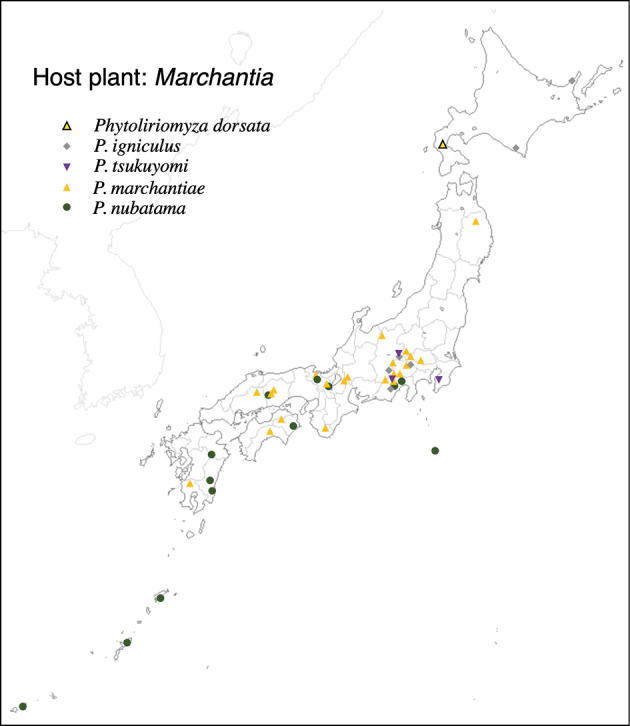
Locality records of three *Phytoliriomyza* species associated with *Marchantia* spp.: *P.dorsata*, *P.igniculus*, *P.tsukuyomi*, *P.marchantiae*and*P.nubatama*.

Thus far, recorded from Europe, North America, and Japan. The authors recorded only from southern Hokkaido (Fig. 2).

#### Remarks.

 The morphology of the Japanese specimens closely coincided with the description of *P.dorsata* by Spencer (1976), but not with the description by Papp and Černý (2017), particularly concerning the morphology of the epandrium and distiphallus, which suggests the presence of multiple related species in Europe. Related *Phytoliriomyza* species (*P.bornholmensis* and *P.islandica*) were described by Spencer (1976) and Ólafsson (1988), but they were synonymized with *P.dorsata* by Zlobin (2005). The taxonomic confusion would be caused by scarcity of specimens of these rare species and lack of information on their biology. Our numerous reared specimens and extensive records of host plants suggest that bryophyte-associated *Phytoliriomyza* species are diverse, and that the synonymized species may be definite species circumscribed by external and genital morphology.

This species resembles *P.brunofasciata* in having two pairs of dark lateral stripes on the scutum, but it is distinguished from *P.brunofasciata* by the dark-sided scutellum (scutellum wholly yellow in *P.brunofasciata*) and the number of tubercle-like setae on the surstylus of the male epandrium (two in *P.dorsata*; one in *P.brunofasciata*).

### ﻿2.﻿ Phytoliriomyza igniculus

Taxon classificationAnimaliaDipteraAgromyzidae

Kato sp. nov.

F9AF04D8-2EF2-567E-88D9-828E1A0C131B

https://zoobank.org/482483ED-DBE9-4882-BF5A-BB033A32FA6B

Figs 3 , 4

#### Material examined.

***Holotype*:** Japan: 1♂ (MK-AG-a323), Matsubara-ko, Koumi, Nagano Pref. (36.053896°N, 138.461847°E, 1110 m), 18-IV-2021 (as larva), emerged on 17-V-2021, NSMT-I-Dip 31890. ***Paratypes*:** Japan: 1♂1♀ (MK-AG-a454, a453), same data as holotype, emerged on 17-V-2021, NSMT-I-Dip 31891, 31892; 1♀ (MK-AG-a324), Iwaobetsu, Shari, Hokkaido, 1-V-2021 (as larva), emerged on 28-V-2021, NSMT-I-Dip 31893; 1♀ (MK-AG-a325), Odarumi, Makioka, Yamanashi Pref., 30-VI-2021 (as larva), emerged on 4-VII-2021, NSMT-I-Dip 31894; 1♀ (MK-AG-a326), Ikawa-toge, Aoi-ku, Shisuoka Pref., 26-V-2021 (as larva), emerged on 9-VI-2021, NSMT-I-Dip 31895; 1♀ (MK-AG-a28), Shirabiso-toge, Kamimura, Iida, Nagano Pref., 27-IV-2014 (as larva), emerged on 22-V-2014, NSMT-I-Dip 31896.

#### Other material.

Japan: 35♂37♀, same data as holotype, emerged on 8–17-V-2021; 9♂5♀, Iwaobetsu, Shari, Hokkaido, 1-V-2021 (as larva), emerged on 26–28-V-2021; 3♂1♀, Horoman-kyo, Samani, Hokkaido, 30-IV-2021 (as larva), emerged on 26–31-V-2021; 1♂2♀, Odarumi, Makioka, Yamanashi Pref., 27-VI-2014 (as larva), emerged on 15-VII-2021; 1♂1♀, Shirabiso-toge, Kamimura, Iida, Nagano Pref., 27-IV-2021 (as larva), emerged on 23–25-V-2021; 22♂20♀, Ikawa-toge, Aoi-ku, Shisuoka Pref., 26-V-2021 (as larva), emerged on 8–17-VI-2021.

#### Diagnosis.

 A large yellow species (wing length 2.0–2.1 mm) having a pruinose dark gray scutum with a trapezoid yellow patch medially on posterior 1/3, a yellow scutellum, black 1^st^ flagellomeres, yellow maxillary palpi, yellow halteres, and yellow legs. Male epandrium with a comb comprising five or six fused long tubercle-like setae. Larva mines the thallus of *Marchantiapolymorpha*.

#### Description.

**Figure 3.**
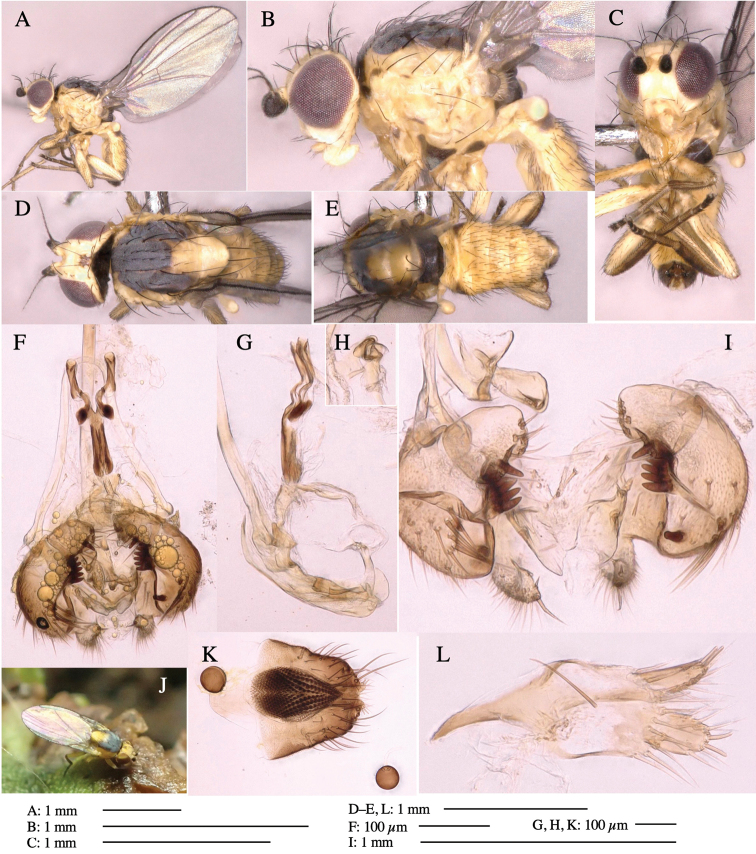
*Phytoliriomyzaigniculus* sp. nov. **A–E** holotype male **A** habitus **B** lateral body **C** frontal head and ventral body **D** dorsal thorax **E** thorax in posterior view **F–H** male genitalia **F** whole genitalia **G** phallic complex **H** postgonite **I** epandrium **J** a living female fly on a thallus of *Marchantiapolymorpha***K, L** female postabdomen **K** oviscape and spermatheca **L** tergite 10.

**Adult male** (Fig. 3A–E).

***Head*:** Head light yellow, with ocellar tubercle dark brown, frons yellowish brown, back of head dark brown excluding margins (Fig. 3C). Antenna porrect, first flagellomere black, pedicel and scape yellow. Arista subbasal, black, pubescent. Clypeus, face, gena, parafacial and postgena yellow. Proboscis normal, yellow; palpus yellow, cylindrical. ***Chaetotaxy*:** Front orbitals three pairs; one ori directed inward; two ors directed upward (Fig. 3B). Orbital setulae minute and erect, in a single row.

***Thorax*:** Thorax pruinose. Scutum dark gray with a trapezoid pale yellow patch medially on posterior 1/3 (Fig. 3D). Scutellum pale yellow. Subscutellum with anterior half yellow, ventral half brown. Mediotergite brown, anatergite and katatergite yellow (Fig. 3B). Pleuron yellow; propleuron with small brownish spots on venter; anepisternum with three brown spots on anterior and middle dorsal margins and middle venter; several small brown spots on anepimeron, katepisternum and meron with brow patches on venter (Fig. 3B). Haltere yellow. Calypter margin and hairs gray (Fig. 3B). Leg segments yellow; tibia and tarsus darker (Fig. 3A). ***Chaetotaxy*:** Scutum with 1+3 dorsocentrals, shortened anteriorly (Fig. 3D). Acrostichal setulae six or seven pairs in two rows. ***Wing***: Wing length 2.1 mm, costa reaching M_1_ (Fig. 3A). Length of ultimate section of vein M_4_ divided by penultimate section 1.3–1.7.

***Abdomen*:** Abdomen dorsally subshiny yellow, with a pair of brown lateral semicircular patches on anterior half of the 2^nd^ tergite; epandrium dark brown (Fig. 3E). ***Genitalia*:** (Fig. 3F–I). Epandrium rounded apically, with a long tubercle-like seta on subposterior inner-lateral surface; inner-anterior surface with a comb comprising five or six fused long tubercle-like setae (Fig. 3I). Surstylus hood-like, curved inward, setose apically, with single long tubercle-like seta on outer distal margin. Cercus narrow, setose, with a strong seta apically (Fig. 3I). Subepandrial sclerite with one pair of flat, pale, blade-like ventral process and one pair of setae outward (Fig. 3I). Hypandrium slightly sclerotized along outer margin (Fig. 3F). Postgonite bare and broadly rounded apically (Fig. 3H). Phallophorus with deep incision below, articulated with phallapodeme, fused to epiphallus (Fig. 3F, G). Basiphallus with a narrow plate on left side; length similar to that of mesophallus (Fig. 3G). Hypophallus broad, membranous, medially with a pair of fused linear sclerites; distal half diverging (Fig. 3G). Paraphallus absent. Mesophallus dark, cylindrical, widest subbasally, slightly shorter than distiphallus (Fig. 3G). Distiphallus comprising one pair of stout tubules; basal half composed of ventral dark bulbous sclerite and weaker medial region curving outward; distal half cylindrical and well-pigmented, with truncated, unpigmented apex (Fig. 3G). Ejaculatory apodeme fan-shaped, blade pale with clear margin, with narrow stalk, broad base, and clear sperm pump.

**Female.** Similar to male, but slightly larger and frons wider. Wing length 2.1 mm. ***Postabdomen*:** (Fig. 3K, L) Oviscape dark brown, setigerous. Tergite 10 cruciform, laterally uniting narrow pleural sclerites (Fig. 3K). Each cercus with two stout, apical, trichoid sensilla, 1/3 length (Fig. 3L). Spermathecae orbicular (Fig. 3K).

#### Etymology.

 The specific name (*igniculus* = small fire) refers to the yellow oblong patch on the scutum.

#### Japanese name.

Kitsunebi-zenigoke-hamoguribae.

#### Host plant.

*Marchantiapolymorpha* (Marchantiaceae).

#### Mine.

**Figure 4.**
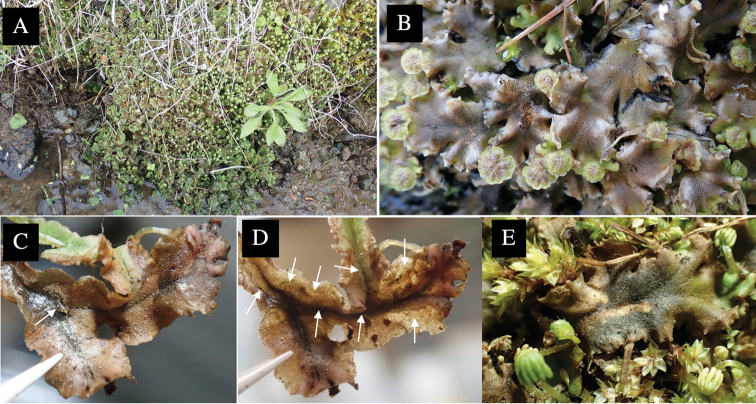
Habitat of *Phytoliriomyzaigniculus* sp. nov. and its host plant, *Marchantiapolymorpha***A** habitat at levee of a rice field at type locality **B** thalli with male receptacles **C** mined thallus containing an internal puparium (arrow) **D** the same thallus in transmitted light (arrows indicating mines) **E** mined thallus at Ikawa-toge.

Larvae construct linear-botch mines in the thallus, particularly in the midrib, and pupate in the mines (Fig. 4C–E).

#### Biological notes.

 The host plants from which this species emerged, grow on mesic soils along roads in cool temperate forests (beech forests dominated by *Faguscrenata* in Honshu and deciduous oak forests dominated by *Quercuscrispula* in Hokkaido) and on levees of paddy fields in a cool temperate forest ecosystem (Fig. 4A, B). Our rearing records suggest that this species is univoltine, and that adults emerge from overwintered pupae in spring.

#### Distribution.

Japan: Hokkaido and Honshu (Fig. 2).

#### Remarks.

This species is distinguished from all other species of *Phytoliriomyza* with black-banded yellow thorax by the unique brown patches on the 2^nd^ abdominal tergite (absent in other species). This species resembles *P.izayoi*,*P.chichibuensis* and *P.caliginosa* in the yellow pattern of the scutum and scutellum; it is distinguished from them by the wholly yellow scutellum without dark lateral corners and by the number of tubercle-like setae of the comb on the male epandrium (*P.igniculus*, 5–6; *P.izayoi*, 6–8; *P.caliginosa*, 8–11). This species also resembles *P.cometiformis* and *P.luna* in having a yellow scutellum; it is distinguished from them by the number of tubercle-like setae of the comb on the male epandrium (*P.igniculus*, 5–6; *P.cometiformis*, 3; *P.luna*, 7–8). This species also resembles *P.islandica* in the yellow pattern of the scutellum; it is distinguished by the black first flagellomere (brown in *P.islandica*).

### ﻿3.﻿ Phytoliriomyza tsukuyomi

Taxon classificationAnimaliaDipteraAgromyzidae

Kato sp. nov.

0F458DB6-BFF1-54D7-A1F7-79428A74D083

https://zoobank.org/B0DF693B-ABD0-45C9-B129-AB3D75DB681E

Fig. 5

#### Material examined.

***Holotype*:** Japan: 1♂ (MK-AG-a397), Matsubara-ko, Koumi, Nagano Pref. (36.053896°N, 138.461847°E, 1110 m asl), 12-V-2021 (as larva), emerged on 18-VI-2021, NSMT-I-Dip 31897. ***Paratypes*:** Japan: 1♂1♀ (MK-AG-a455, a443), same data as holotype, emerged on 17–22-VI-2021, NSMT-I-Dip 31898, 31899; 1♂1♀ (MK-AG-a521, a528), Ikawa-toge, Aoi-ku, Shisuoka Pref., 26-V-2021 (as larva), emerged on 31-V–2-VI-2021, NSMT-I-Dip 31900, 31901; 1♂ (MK-AG-a577), Tokuzo-ji, Yana, Kisarazu, Chiba Pref., 1-XI-2021 (as larva), emerged on 3-XII-2021, NSMT-I-Dip 31902.

#### Other material.

Japan: 1♂1♀, Ikawa-toge, Aoi-ku, Shizuoka Pref., 26-V-2021 (as larva), emerged on 31-V–2-VI-2021; 1♂, Tokuzo-ji, Yana, Kisarazu, Chiba Pref., I-XI-2021 (as larva), emerged on 3-XII-2021.

#### Diagnosis.

 A medium-sized species (wing length 1.6–1.7 mm) having pruinose dark gray scutum, light yellow scutellum, black 1^st^ flagellomere, dark maxillary palpus, dark halteres, and dark legs. Male epandrium inner-basally with a comb of four unfused tubercle-like setae, and inner-laterally with a comb of three or four fused, long, tubercle-like setae; surstylus bilobed and hypertrophied dorsal arm with one tubercle-like seta. Larva mines the thallus of *Marchantiapolymorpha*.

#### Description.

**Figure 5.**
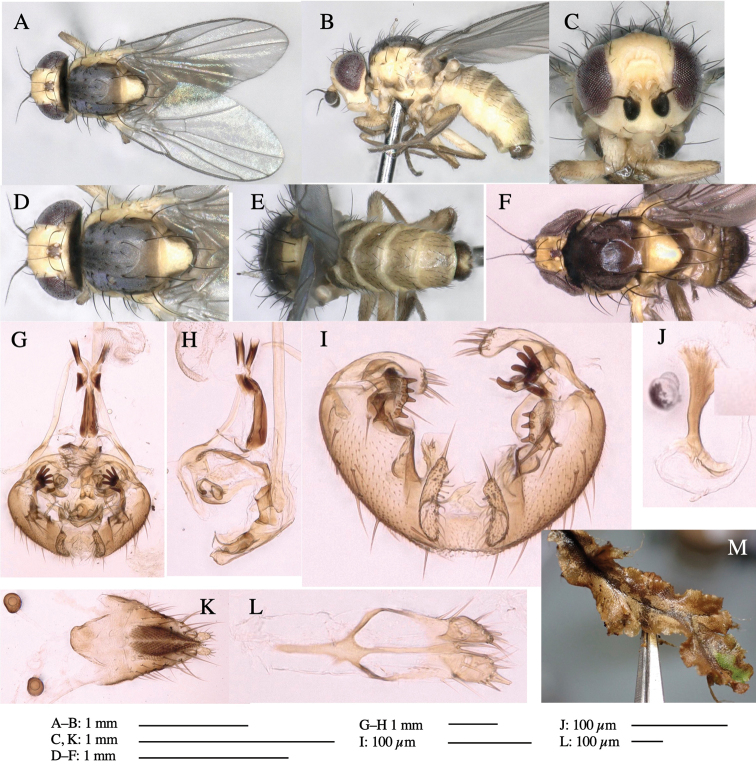
*Phytoliriomyzatsukuyomi* sp. nov. **A–E** holotype male **A** habitus, **B** lateral **C** frontal **D** dorsal **E** posterior** F** paratype female (MK-AG-a442) **G–J** male genitalia **G** whole genitalia, ventral **H** phallus complex, lateral **I** epandrium **J** ejaculatory apodeme, lateral **K, L** female postabdomen **K** oviscape and spermatheca **L** tergite 10 **M** mined thallus of *Marchantiapolymorpha*.

**Adult male** (Fig. 5A–E).

***Head*:** Head light yellow, with ocellar tubercle brown, frons light yellow, back of head dark brown (Fig. 5C). Antenna porrect, first flagellomere black, pedicel and scape light yellow. Arista subbasal, black, pubescent. Clypeus, face, gena, parafacial and postgena yellow. Proboscis normal, yellow; palpus brownish gray, cylindrical. ***Chaetotaxy*:** Front orbitals three pairs; one ori directed inward; two ors directed upward (Fig. 5D). Orbital setulae minute and erect, in a single row.

***Thorax*:** Thorax pruinose. Scutum pruinose bluish gray with a medial black band and a pair of lateral black bands (Fig. 5D). Scutellum pale yellow with lateral corner dark brown. Subscutellum dark brown with anterior margin pale yellow. Mediotergite, anatergite, and katatergite brown. Pleuron largely yellow; postpronotal lobe with anterior brown spot; notopleuron with brown spot on venter; anepisternum and anepimeron with brown venters; katepisternum an meron with dark brown patches on venter (Fig. 5B). Haltere gray, with grayish yellow stalk. Calypter margin and hairs gray. Leg segments brownish gray; tarsus darker. ***Chaetotaxy*:** Scutum with 1+3 dorsocentrals, shortened anteriorly (Fig. 5D). Acrostichal setulae six or seven pairs in two rows. ***Wing***: Wing length 1.7 mm, costa reaching M_1_ (Fig. 5A). Length of ultimate section of vein M_4_ divided by penultimate section 1.9.

***Abdomen*:** Abdomen dorsally subshiny brown; epandrium dark brown (Fig. 5E). ***Genitalia*:** (Fig. 5G–J) Epandrium rounded apically; inner-lateral margin with a large round arm having three or four unfused tubercle-like setae apically; inner-anterior surface with a large arm having a hand-shaped comb comprising four basally fused long tubercle-like setae apically, and one tubercle-like seta immediately inward from the protrusion (Fig. 5I). Surstylus lobate, setose apically; inner-basally with a long, S-curved, hypertrophied, columnar arm, bearing one long tubercle-like seta apically (Fig. 5I). Cercus narrow, setose, with a strong seta apically (Fig. 5I). Subepandrial sclerite consisting of one pair of lateral plates (one seta each subapically on inner surface) forming (Fig. 5I). Hypandrium slightly sclerotized along outer margin (Fig. 5G). Postgonite bare, goose barnacle-shaped and cleft apically; upper lobe pointed apically (Fig. 5H). Phallophorus with deep incision below (Fig. 5G), articulated with phallapodeme, fused to epiphallus (Fig. 5H). Basiphallus with a narrow plate on left side; dorsally lightly sclerotized; shorter than mesophallus. Hypophallus broad, membranous, ventral side covered with microtrichia, medially with a pair of short fused linear sclerites (Fig. 5G, H). Paraphalli lobe-like, membranous; posterior margin slightly sclerotized (Fig. 5G, H). Mesophallus dark, cylindrical, widest subbasally, slightly longer than distiphallus (Fig. 5H). Distiphallus comprising one pair of stout tubules; basal half composed of ventral dark triangular sclerite and weaker medial region; distal half cylindrical, dorsally pigmented, with truncated, unpigmented apex (Fig. 5H). Ejaculatory apodeme fan-shaped, blade pale with clear margin, with sclerotized stalk, broad asymmetric base, and clear sperm pump (Fig. 5J).

**Female** (Fig. 5F–I). Similar to male, but slightly larger and frons narrower (Fig. 5F). Wing length 1.7 mm. ***Postabdomen*:** (Fig. 5K, L) Oviscape dark brown, setigerous (Fig. 5K). Tergite 10 cruciform, laterally uniting narrow pleural sclerites (Fig. 5L). Each cercus with two stout, apical, trichoid sensilla, 1/3 length of cercus. Spermathecae orbicular (Fig. 5K).

#### Etymology.

 The specific name *tsukuyomi* is a Japanese word meaning brightness of moonlight, and refers to the bright yellow scutellum.

#### Japanese name.

Tsukuyomi-zenigoke-hamoguribae.

#### Host plant.

*Marchantiapolymorpha* (Marchantiaceae).

#### Mine.

Larvae mine the thallus of the host plant and pupate in the mines. The mines are not apparent from the outside.

#### Biological notes.

The host plants from which this species emerged, grow on mesic soils on levee of paddy fields and along roads in natural beech forests (Fig. 5T). Our rearing records suggest that this species is univoltine, and that adults emerge from overwintered pupae in spring.

#### Distribution.

Japan: Honshu (Fig. 2).

#### Remarks.

 This species resembles *P.alpicola* in color pattern of the scutum (entirely dark mesothorax and uniformly pale scutellum); it is distinguished from the latter by the yellow pedicel and scape of the antenna (pedicel and scape of *P.alpicola* are brown), yellow maxillary palpus (dark gray in *P.alpicola*), and number and arrangement of tubercle-like setae in a comb on the male epandrium (4 hand-shaped in *P.tsukuyomi*; 6 fused in *P.alpicola*).

### ﻿4.﻿ Phytoliriomyza marchantiae

Taxon classificationAnimaliaDipteraAgromyzidae

Kato sp. nov.

190EAA65-1A70-51A2-B2CE-ABEBB1717315

https://zoobank.org/FD0892D0-011B-4A1A-A1FE-F1ECC92FF89E

Figs 6 , 7

#### Material examined.

***Holotype*:** Japan: 1♂ (MK-AG-a320), Namari-kawa, Yakumo, Futami, Hokkaido (42.187765°N, 140.122182°E, 190 m asl), 2-VI-2021 (as larva on *Marchantiapaleaceapaleacea*), emerged on 17-VI-2021, NSMT-I-Dip 31903. ***Paratypes*:** Japan: 1♂2♀ (MK-AG-a484, a485, a442), same data as holotype, emerged on 18-VI–3-VII-2021, NSMT-I-Dip 31904–31906; 1♂ (MK-AG-a322), Renge-onsen, Itoigawa, Niigata Pref., 11-VII-2021 (as larva on *M.p.paleacea*), emerged on 5-VIII-2021, NSMT-I-Dip 31907; 1♂ (MK-AG-a321), Nippara, Okutama, Tokyo Pref., 27-III-2021 (as larva on *M.p.paleacea*), emerged on 27-V-2021, NSMT-I-Dip 31908; 1♀ (MK-AG-302), Umegashima, Aoi-ku, Shizuoka Pref., 5-I-2014 (as larva on *M.p.diptera*), emerged on ?-V-2014, NSMT-I-Dip 31909; 1♂ (MK-AG-a356), Inago, Shibakawa, Fujinomiya, Shizuoka Pref., 20-III-2000 (as larva on *M.p.diptera*), emerged on 17-IV-2000, NSMT-I-Dip 31910; 1♂ (MK-AG-a355), Muramatsu, Iwakura, Sakyo-ku, Kyoto Pref., 11-VI-2021 (as larva on *M.polymorpha*), emerged on 21-VII-2021, NSMT-I-Dip 31911; 1♂ (MK-AG-a358), Naiku, Oe, Fukuchiyama, Kyoto Pref., 19-V-2021 (as larva on *M.papillatagrossibarba*), emerged on 3-VII-2021, NSMT-I-Dip 31912.

#### Other material.

 Japan: On *Marchantiapaleaceapaleacea*: 22♂16♀, Namari-kawa, Yakumo, Futami, Hokkaido, 2-VI-2021 (as larva), emerged on 9-VI–7-VII-2021; 38♂44♀, Renge-onsen, Itoigawa, Niigata Pref., I-VII-2021 (as larva), emerged on 29-VII–9-VIII-2021; 2♂1♀, Narahara, Ueno, Tano, Gunnma Pref., 18-IV-2021 (as larva), emerged on 30–31-V-2021; 2♂4♀, Kanna-gawa, Nakatsugawa, Chichibu, Saitama Pref., 14-XI-2010 (as larva), emerged on 26-IV-2010; 2♂1♀, Nippara, Okutama, Tokyo Pref., 27-III-2021 (as larva), emerged on 27-IV–21-V-2021; 5♂5♀, Yashajin-toge, Minami-arupusu, Yamanashi Pref., 25-III-2021 (as larva), emerged on 2-V–1-VI-2021.

On *Marchantiapaleaceadiptera*: 2♂3♀, Inago, Shibakawa, Fujinomiya, Shizuoka Pref., 26-V-2021 (as larva), emerged on 17–27-VI-2021; 1♂5♀, Abe-toge, Aoi-ku, Shizuoka Pref., 30-XI-2014 (as larva), emerged on 30-IV–8-V-2014; 3♂5♀, Kuchisakamoto, Aoi-ku, Shizuoka Pref., 26-V-2021 (as larva), emerged on 1–26-VI-2021; 6♂8♀, Tsudono, Aoi-ku, Shizuoka Pref., 30-XI-2014 (as larva), emerged on 30-IV–6-V-2014; 2♂4♀, Tosayama, Kochi, Kochi Pref., 27-II-2011 (as larva), emerged on 23–28-IV-2011; 1♂3♀, Mt. Nabejiri, Taga, Shiga Pref., 4-V-2021 (as larva), emerged on 27-V–15-VI-2021; 6♂3♀, Naiku, Oe, Fukuchiyama, Kyoto Pref., 19-V-2021 (as larva), emerged on 2–10-VII-2021; 2♂3♀, Iya-kei, Ikeda, Miyoshi, Tokushima Pref., 1-II-2014 (as larva), emerged on 4–4-V-2014; 2♂5♀, Okujisso, Isa, Kagoshima Pref., 17-XII-2017 (as larva), emerged on 22–30-III-2012.

On *Marchantiapolymorpha*: 1♀, Matsubara-ko, Koumi, Nagano Pref., 18-IV-2021 (as larva), emerged on 18-VI-2021; 7♂2♀, Odarumi, Makioka, Yamanashi Pref., 30-VI-2021 (as larva), emerged on 28-VII–4-VII-2021; 5♂15♀, Muramatsu, Iwakura, Sakyo-ku, Kyoto Pref., 31-XII-2013 (as larva), emerged on ?-V-2013.

On *Marchantiapapillatagrossibarba*: 2♂1♀, Inago, Shibakawa, Fujinomiya, Shizuoka Pref., 26-V-2021 (as larva), emerged on 18-VII-2021; 3♂5♀, Naiku, Oe, Fukuchiyama, Kyoto Pref., 19-V-2021 (as larva), emerged on 2–10-VII-2021; 2♂1♀, Seikandoro, Kumanogawa, Shingu, Wakayama Pref., 7-VII-2021 (as larva), emerged on 27-VII–4-VIII-2021; 1♀, Nagabuchi, Ume, Saeki, Oita Pref., 29-XI-2011 (as larva), emerged on 3-V-2011.

#### Diagnosis.

 A medium-sized species (wing length 1.6–1.8 mm) having dark gray scutum, dark-cornered yellow scutellum, black 1^st^ flagellomere, black maxillary palpus, gray halteres, and brown legs. Male epandrium with a comb comprising seven or eight fused long tubercle-like setae. Larva mines the thallus of *Marchantia* spp.

#### Description.

**Figure 6.**
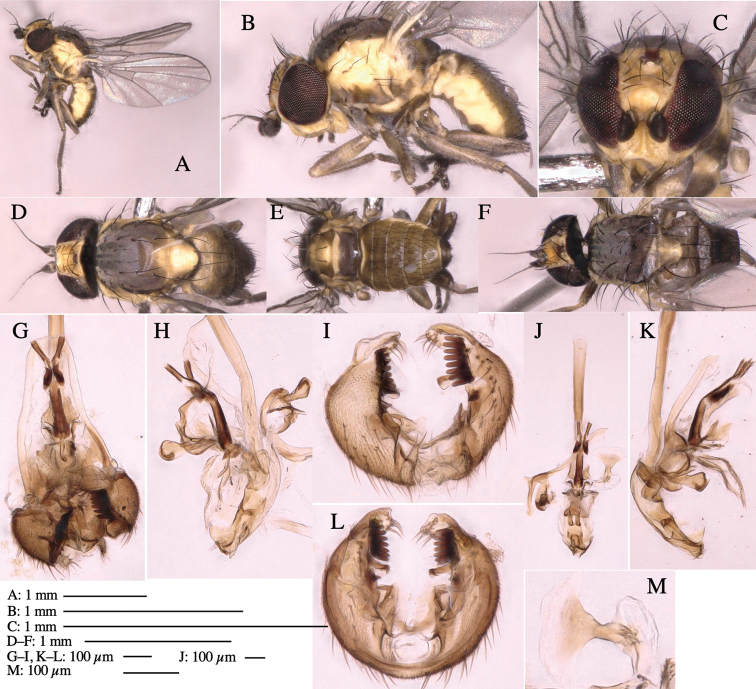
*Phytoliriomyzamarchantiae*sp. nov. **A–E** holotype male **A** habitus **B** lateral **C** frontal **D** dorsal **E** posterior** F** paratype female (MK-AG-a442), dorsal **G–I** male genitalia **G–I** emerged from *Marchantiapaleaceapaleacea* at type locality **J–M** emerged from *M.papillatagrossibarba*at Mt. Oe. **G** whole genitalia, ventral **H **phallus complex, lateral** I, L** epandrium, ventral** J, K **phallic complex, ventral and lateral **M** ejaculatory apodeme, lateral.

**Adult male** (Fig. 6A–E).

***Head*:** Head light yellow, with ocellar tubercle brown, frons light yellow, back of head dark brown (Fig. 6C). Antenna porrect, first flagellomere black, pedicel and scape brown. Arista subbasal, black, pubescent. Clypeus, face, gena, parafacial and postgena yellow. Proboscis normal, yellow; palpus brown, cylindrical. ***Chaetotaxy*:** Front orbitals three pairs; one ori directed inward; two ors directed upward (Fig. 6B). Orbital setulae minute and erect, in a single row.

***Thorax*:** Thorax pruinose. Scutum pruinose gray, with a dark gray medial stripe on anterior 2/3, one pair of dark gray lateral stripes, and with narrow yellow patch along posterior margin (Fig. 6D). Scutellum light yellow with lateral corners widely brownish. Subscutellum brown with anterior margin light yellow. Mediotergite, anatergite and katatergite dark brown. Pleuron largely yellow; postpronotal lobe with anterior brown spot; notopleuron with brown narrow spot along anterior lower margin; anepisternum and anepimeron with brown venters; katepisternum and meron with dark brown patches on venter (Fig. 6B). Haltere yellowish gray, with yellow stalk. Calypter margin and hairs gray. Leg segments brown; tibia and tarsus darker (Fig. 6A). ***Chaetotaxy*:** Scutum with 1+3 dorsocentrals, shortened anteriorly (Fig. 6D). Acrostichal setulae five or six pairs largely in two rows. ***Wing***: Wing length 1.6–1.7 mm, costa reaching M_1_ (Fig. 6A). Length of ultimate section of vein M_4_ divided by penultimate section 1.6–1.8.

***Abdomen*:** Abdomen dorsally subshiny brown (Fig. 6E). ***Genitalia*:** (Fig. 6G–M) Epandrium dark brown, rounded apically, inner-lateral surface medially with one tubercle-like setae; inner-anterior surface ventrally with a comb comprising eight fused long tubercle-like setae; ventral margin with a row of several minute tubercle-like setae (Fig. 6I, L). Surstylus subrectangular, setose apically. Cercus narrow, setose. Subepandrial sclerite with one pair of spines and one pair of plate-like ventral processes; tips hook-like (Fig. 6I). Hypandrium slightly sclerotized along outer margin. Postgonite bare and goose barnacle-shaped (Fig. 6H). Phallophorus with shallow incision below (Fig. 6J), articulated with phallapodeme, fused to epiphallus (Fig. 6H, K). Basiphallus with subtriangular dorsal sclerite; 2/3 × as long as mesophallus (Fig. 6H, K). Hypophallus subtriangular membranous, lined with numerous microtrichia ventrally, medially with a pair of fused linear pale sclerites (Fig. 6H, K). Paraphalli consisting of a pair of incurved narrow pigmented sclerites and a pair of triangular plate-like pigmented sclerites (Fig. 6H, K). Mesophallus dark, cylindrical, widest subbasally, slightly longer than distiphallus (Fig. 6H). Distiphallus comprising one pair of stout tubules; basal half composed of ventral dark scalpel-shaped sclerite and weaker medial region; distal half cylindrical, dorsally and laterally pigmented, with truncated, unpigmented apex (Fig. 6H, K). Ejaculatory apodeme fan-shaped, blade pale with apical half clear; sclerite of sperm pump with lateral extension; sperm pump clear (Fig. 6M).

**Figure 7.**
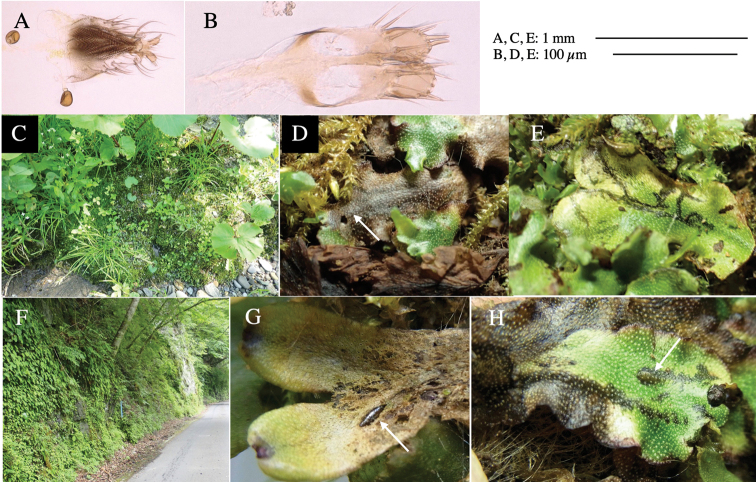
Female morphology and larval ecology of *Phytoliriomyzamarchantiae*sp. nov. **A, B** female postabdomen (at Renge-onsen) **A **oviscape and spermatheca **B **tergite 10 **C–E** habitat and mined thalli on *Marchantiapaleaceapaleacea***C** habitat at type locality **D, E** mined thalli at Yashajin-toge and Renge-onsen** F–H **habitat and mined thalli on *M.p.diptera*** F **habitat at Kuchisakamoto **G, H** mined thalli at Iwaya-kei and Tosayama. Arrows indicate internal puparia.

**Female** (Fig. 6I–K). Similar to male, but scutellum darker than male (Fig. 6F). Wing length 1.6 mm. ***Postabdomen*:** (Fig. 7A, B) Oviscape dark brown, setigerous (Fig. 7A). Tergite 10 cruciform, laterally uniting narrow pleural sclerites (Fig. 7B). Cercus with two stout, apical, trichoid sensilla, ½ length of cercus. Spermathecae semi-orbicular (Fig. 7A).

#### Variation.

The yellow patch on posterior scutum slightly varied from distinct to obscure ones among localities but not among host liverwort species/subspecies (Fig, 6F–K).

#### Etymology.

 The specific name refers to host plant genus, *Marchantia*.

#### Japanese name.

Uzumibi-zenigoke-hamoguribae.

#### Host plants.

 The main host plants are *Marchantiapaleaceapaleacea* and *M.p.diptera* (Marchantiaceae), with *M.polymorpha* and *M.papillatagrossibarba* also recorded as host in some localities.

#### Mine.

Larvae construct linear mines in the thallus, particularly in the midrib, and pupate in the mined thalli (Fig. 7D, E, G, H).

#### Biological notes.

*Marchantiapaleaceapaleacea* grows on rocky substrates of cliffs, slopes and riverbanks in cool temperate deciduous forests (Fig. 7C) and *Marchantiap.diptera* in warm temperate evergreen forests (Fig. 7F). While these subspecies are frequently utilized by *P.marchantiae*, they are never utilized by the other three *Marchantia*-associated species, *P.igniculus*,*P.tsukuyomi*, and *P.nubatama*. *P.marchantiae* is also recorded from *M.polymorpha* in cool temperate forests and *M.papillatagrossibarba* in warm temperate evergreen forests in some localities. Our rearing records suggest that this species is multivoltine.

#### Distribution.

Japan: Hokkaido, Honshu, Shikoku, Kyushu (Fig. 2).

#### Remarks.

 This species resembles *P.rebouliae*,*P.lanternaria*, and *P.conocephali* in the narrow yellow posterior margin of the scutum and medial yellow stripe of the scutellum; it is distinguished from *P.rebouliae* by number of tubercle-like setae in a comb of the male epandrium (8 in *P.marchantiae*; 7 in *P.rebouliae*), from *P.lanternaria* and *P.conocephali* by the number of tubercle-like setae on the surstylus of the male epandrium (0 in *P.marchantiae*; 1–2 in *P.lanternaria* and *P.conocephali*).

This species also resembles *Phytoliriomyzamiki* (Strobl, 1898) in color pattern of the scutum and morphology of the male genitalia; it is distinguished from the latter by the number of tubercle-like setae in a comb on the male epandrium (8 in *P.marchantiae*; 5 in *P.miki*).

### ﻿5.﻿ Phytoliriomyza nubatama

Taxon classificationAnimaliaDipteraAgromyzidae

Kato sp. nov.

2120AFBE-6347-50BE-B76E-25D25A4D841B

https://zoobank.org/B7A9AD45-5940-4EF5-AF24-D768250DC41A

Figs 8 , 9

#### Material examined.

***Holotype*:** Japan: 1♂ (MK-AG-a413), Mt. Mihara, Hachijo Is. Tokyo Pref. (33.11383°N, 139.82415°E, 170m), 17-II-2012 (as larva on *Marchantiaemarginatacuneiloba*), emerged on 23-IV-2012, NSMT-I-Dip 31913. ***Paratypes*:** Japan: 1♀ (MK-AG-323), same data as holotype, NSMT-I-Dip 31914; 1♀ (MK-AG-a379), Naiku, Oe, Fukuchiyama, Kyoto Pref., 19-V-2021 (as larva on *M.papillatagrossibarba*), emerged on 9-VI-2021, NMNS; 1♀ (MK-AG-a383), Tsudono, Aoi-ku, Shizuoka Pref., 19-V-2021 (as larva on *M.papillatagrossibarba*), emerged on 10-V-2014, NSMT-I-Dip 31916; 1♂1♀ (MK-AG-a456, a359), Mt. Osuzu, Tsuno, Miyazaki Pref., 11-IV-2021 (as larva on *M.papillatagrossibarba*), emerged on 21–22-V-2021, NSMT-I-Dip 31917, 31918; 1♀ (MK-AG-344), Jizodo, Mariya, Kisarazu, Chiba Pref., 17-III-2016 (as larva on *M.pinnata*), emerged on 27-IV-2016, NSMT-I-Dip 31919.

#### Other material.

 Japan: On *Marchantiapapillatagrossibarba*: 9♂9♀, Inago, Shibakawa, Fujinomiya, Shizuoka Pref., 26-V-2021 (as larva), emerged on 11-VI–2-VII-2021; 3♂3♀, Tsudono, Aoi-ku, Shizuoka Pref., 30-XI-2014 (as larva), emerged on 29-VII–9-VIII-2021; 6♂6♀, Naiku, Oe, Fukuchiyama, Kyoto Pref., 19-V-2021 (as larva), emerged on 1–16-VI-2021; 2♂, Muramatsu, Iwakura, Sakyo-ku, Kyoto Pref., 27-X-2017 (as larva), emerged on 9-XII-2017; 5♂1♀, Nagabuchi, Ume, Saeki, Oita Pref., 29-XI-2011 (as larva), emerged on 1–5-V-2011; 1♂9♀, Mt. Osuzu, Tsuno, Miyazaki Pref., 11-IV-2021 (as larva), emerged on 16–25-V-2021.

On *Marchantiaemarginatacuneiloba*: 2♀, Takae, Higashi-son, Kunigami, Okinawa Pref., 11-XI-2021 (as larva), emerged on 22–28-XI-2021; 2♂2♀, Nagura, Ishigaki Is., Yaeyama, Okinawa Pref., 7-XI-2021 (as larva), emerged on 13-XI–16-XII-2021.

On *Marchantiapinnata*: 1♀, Jizodo, Mariya, Kisarazu, Chiba Pref., 17-III-2016 (as larva), emerged on 27-IV-2016.

#### Diagnosis.

 A small black species (wing length 1.3–1.6 mm) having subshiny black scutum with an oval yellow pattern extending from mid-posterior margin to scutellum, black 1^st^ flagellomere, yellow maxillary palpus, yellow halteres, and brownish legs. Male epandrium with a comb comprising seven fused tubercle-like setae, and surstylus with a comb comprising three fused tubercle-like setae. Larva mines the thallus of *Marchantiaemarginatacuneiloba*,*M.papillatagrossibarba* and *M.pinnata*.

#### Description.

**Figure 8.**
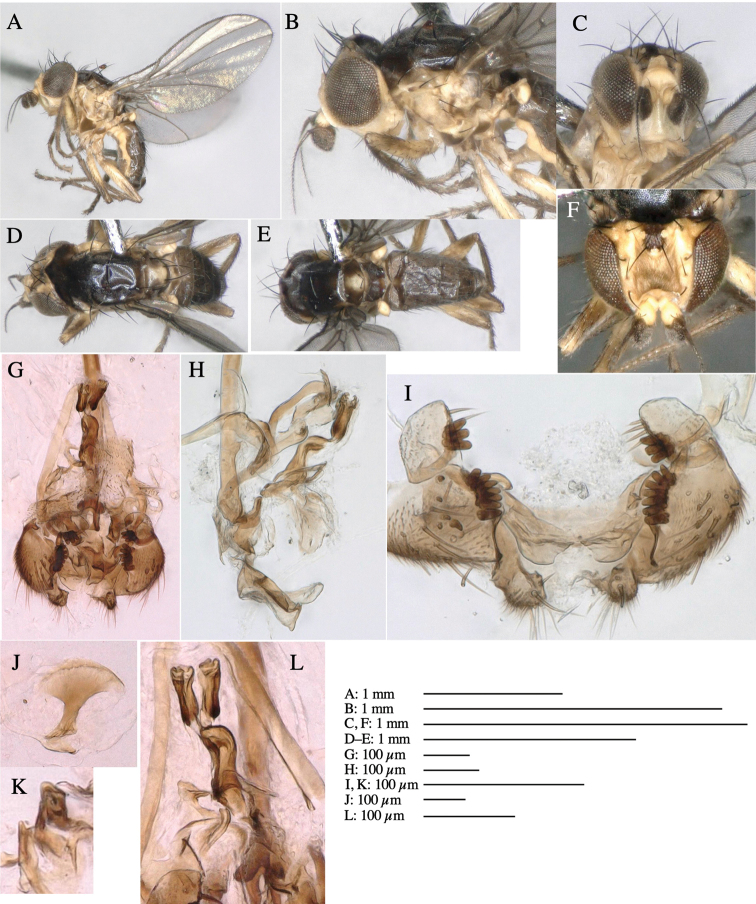
*Phytoliriomyzanubatama*sp. nov. **A–D, G** holotype male **A** habitus **B** lateral **C** frontal **D** dorsal **E, F** paratype female (MK-AG-323),** E** dorsal **F** frontal **G–L** male genitalia (**G–K** type locality **L** Kume Island) **G** whole genitalia, ventral **H** phallic complex, lateral **I** epandrium, ventral **J** ejaculatory apodeme lateral **K** postgonite **L** phallic complex, ventral.

**Adult male** (Fig. 8A–E).

***Head*:** Head light yellow, with ocellar triangle yellow but ocellar tubercle dark brown, back of head dark brown (Fig. 8C). Frons light yellow, with reflective pruinosity. Antenna porrect, first flagellomere dark brown, pedicel and scape brown. Arista subbasal, black, pubescent. Clypeus, face, gena, parafacial and postgena light yellow. Proboscis normal, pale yellow; palpus dark yellow, cylindrical (Fig. 8B). ***Chaetotaxy*:** Front orbitals three pairs; one ori directed inward; two ors directed upward (Fig. 8B). Orbital setulae minute and erect, in a single row.

***Thorax*:** Thorax subshiny. Scutum black. Scutellum black, medially with a small, obscure yellow patch; subscutellum black, anterior margin light yellow (Fig. 8D). Mediotergite dark brown, anatergite and katatergite light yellow. Pleuron largely light yellow; postpronotal lobe with anterior brown patch; notopleuron brownish yellow; anepisternum and anepimeron grayish yellow with brown spots on venters; katepisternum an meron with large dark brown patches on venter (Fig. 8B). Haltere light yellow, stalk grayish yellow. Calypter margin and hairs gray. Leg segments brown; basal half of femur brownish yellow. ***Chaetotaxy*:** Scutum with 1+3 dorsocentrals, shortened anteriorly (Fig. 8D). Acrostichal setulae five pairs in two rows. ***Wing***: Wing length 1.3&nbsp;mm, costa reaching M_1_ (Fig. 8A). Length of ultimate section of vein M_4_ divided by penultimate section 1.0–1.3.

***Abdomen*:** Abdomen dorsally subshiny dark brown. ***Genitalia*:** (Fig. 8G–L) Epandrium dark brown, rounded apically, inner-lateral surface with a comb comprising seven or eight fused long tubercle-like setae (Fig. 8I). Surstylus rounded; curved inwards, only sparsely setose apically; with a comb comprising three long fused tubercle-like setae on posterior margin (Fig. 8I). Cercus narrow, setose. Subepandrial sclerite with one pair of pale, broad, plate-like ventral process (Fig. 8I). Hypandrium slightly sclerotized along outer margin (Fig. 8G). Postgonite bare, goose barnacle-shaped, and cleft apically; upper lobe pointed apically (Fig. 8K). Phallophorus with deep incision below (Fig. 8G), articulated with phallapodeme, fused to epiphallus. Basiphallus 1/2 length of mesophallus (Fig. 8H). Basiphallus with narrow plate-like dorsal sclerite (Fig. 8H). Hypophallus membranous with lateral margins lightly sclerotized, medially with a pair of fused linear ventrally incurved sclerites (Fig. 8H, L). Paraphallus membranous supported by a pair of narrow plate-like pigmented sclerites (Fig. 8H, L). Mesophallus dark, cylindrical, S-curved in lateral view, longer than distiphallus (Fig. 8H). Distiphallus comprising one pair of stout tubules; each cylindrical, with a weak constriction medially, dorsally and laterally pigmented; with truncated apex; opening with indentation (Fig. 8H). Ejaculatory apodeme wide fan-shaped with narrow stalk and stout base; sperm pump clear with ventral sclerotized bar (Fig. 8J).

**Figure 9.**
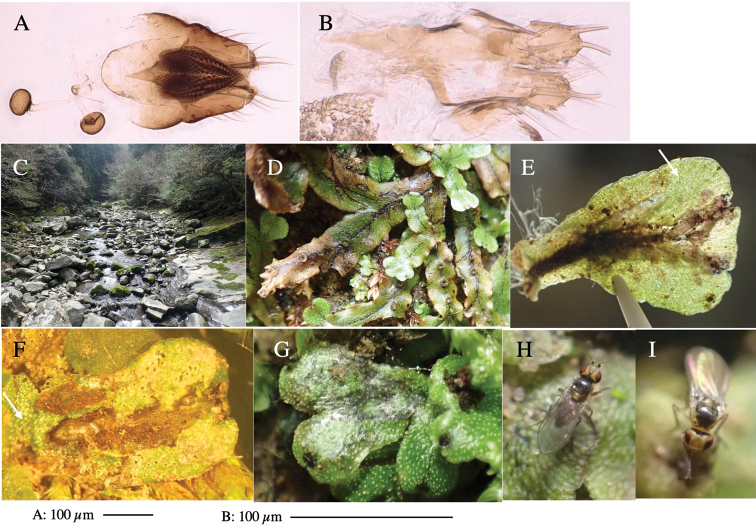
Female morphology (**A, B**) and larval/adult ecology of *Phytoliriomyzanubatama*sp. nov. (**C–E** at Tazukawa-keikoku **F, G** at Inago) **A, B** female postabdomen at type locality **A** oviscape and spermatheca **B** tergite 10 **C** landscape of riparian habitat **D–G** mined thalli of *Marchantiapapillatagrossibarba*(arrows indicate internal puparia) **H, I** live female flies at Iriomote Is on thalli of *Marchantiaemarginatacuneiloba*.

**Female** (Fig. 8F). Similar to male, but slightly larger and frons wider. Wing length 1.4–1.6 mm. ***Postabdomen*:** (Fig. 9A, B) Oviscape dark brown, setigerous (Fig. 9A). Tergite 10 cruciform, laterally uniting narrow pleural sclerites (Fig. 9B). Each cercus with two stout, apical, trichoid sensilla, 1/2 length of cercus (Fig. 9B). Spermathecae semi-orbicular (Fig. 9A).

#### Etymology.

 The specific name *nubatama* is a Japanese word meaning glossy black seed of the blackberry lily (*Irisdomestica* (L.) Goldblatt & Mabb.), to which morphology of adult fly’s scutum of this species is likened.

#### Japanese name.

Nubatama-tosazenigoke-hamoguribae.

#### Host plant.

*Marchantiaemarginatacuneiloba*,*M.papillatagrossibarba* and *M.pinnata* (Marchantiaceae).

#### Mine.

Larvae construct linear mines in the thallus, and pupate in the midrib of the mined thalli (Fig. 9D–G).

#### Biological notes.

 The main habitats of this species are river banks where *Marchantiapapillatagrossibarba* grew on rocky substrate (Fig. 9C). In Izu Islands and Ryukyu Islands, the host plant species *Marchantiaemarginatacuneiloba* is found on damp banking and levee of paddy fields. Our rearing records suggest that this species is multivoltine.

#### Distribution.

Japan: Honshu, Shikoku, Kyushu, Amami, Okinawa and Yaeyama Islands (Fig. 2).

#### Remarks.

 This species resembles *P.foliocerotis* in glossy black scutum and black scutellum with small yellow spot; it is distinguished from the latter by the black 1^st^ flagellomere (yellow in *P.foliocerotis*).

##### Species associated with *Dumortiera*

### ﻿6.﻿ Phytoliriomyza dumortierae

Taxon classificationAnimaliaDipteraAgromyzidae

Kato sp. nov.

214844B5-23DF-5237-817C-A2B70B855E77

https://zoobank.org/68B05020-D8FD-46E0-87FB-2FEBBF2B9A68

Figs 10 , 11

#### Material examined.

***Holotype*:** Japan: 1♂ (MK-AG-246), Higashinakama, Sumiyo, Amami, Kagoshima Pref. (28.269613°N, 129.436562°E, 340 m asl), 21-II-2015 (as larva on *Dumortierahirsuta*), emerged on 1-IV-2015, NSMT-I-Dip 31920. ***Paratypes*:** Japan: 1♂2♀ (MK-AG-a486, a487, a488), same data as holotype, emerged on 30-III–7-IV-2015, NSMT-I-Dip 31921–31923; 1♀ (MK-AG-267), Abe-toge, Aoi-ku, Shizuoka Pref., 1-V-2015 (as larva), emerged on 18-V-2015, NSMT-I-Dip 31924; 1♀ (MK-AG-241), Narutaki, Ichiu, Tsurugi, Tokushima Pref., 12-VI-2017 (as larva), emerged on 21-VI-2017, NSMT-I-Dip 31926; 1♀ (MK-AG-282), Isso, Yaku Is., Kumage, Kagoshima Pref., 29-III-2017 (as larva), emerged on 2-V-2017, NSMT-I-Dip 31927; 1♀ (MK-AG-250), Nagakumo-toge, Tatsugo, Oshima, Kagoshima Pref., 23-II-2016 (as larva), emerged on 25-III-2015, NSMT-I-Dip 31928.

#### Other material.

Japan: 5♂4♀, Abe-toge, Aoi-ku, Shizuoka Pref., 5-I-2015 (as larva), emerged on 18-V-2016; 2♂3♀, Tango-kanzaki, Maizuru, Kyoto Pref., 19-V-2021 (as larva), emerged on 30-V–5-VI-2021; 1♂1♀, Tategasaki, Kumano, Mie Pref., 23-IV-2021 (as larva), emerged on 11-V-2021; 3♀, Gakuen-ji, Bessho, Izumo, Shimane Pref., 31-III-2015 (as larva), emerged on 9-V-2015; 3♂1♀, Tazukawa-keikoku, Katsuura, Tokushima Pref., 30-III-2021 (as larva), emerged on 11–20-V-2021; 3♂4♀, Narutaki, Ichiu, Tsurugi, Tokushima Pref., 12-VI-2017 (as larva), emerged on 17–21-VI-2017; 1♀, Tashiro, Kinko, Kimotsuki, Kagoshima Pref., 22-III-2015 (as larva), emerged on 1-V-2015; 1♂1♀, Isso, Yaku Is., Kumage, Kagoshima Pref., 11-III-2016 (as larva), emerged on 617–19-IV-2016; 11♂14♀, Higashinakama, Sumiyo, Amami, Kagoshima Pref., 21-II-2015 (as larva), emerged on 30-III–8-IV-2015.

#### Diagnosis.

A large dark species (wing length 1.9–2.2 mm) having subshiny dark brown scutum, brownish yellow scutellum, black 1^st^ flagellomere, dark maxillary palpus, dark halteres, and dark gray legs. Male epandrium inner-laterally with an incomplete comb comprising three short, fused, tubercle-like setae medially. Larva mines the thallus of *Dumortierahirsuta*.

#### Description.

**Figure 10.**
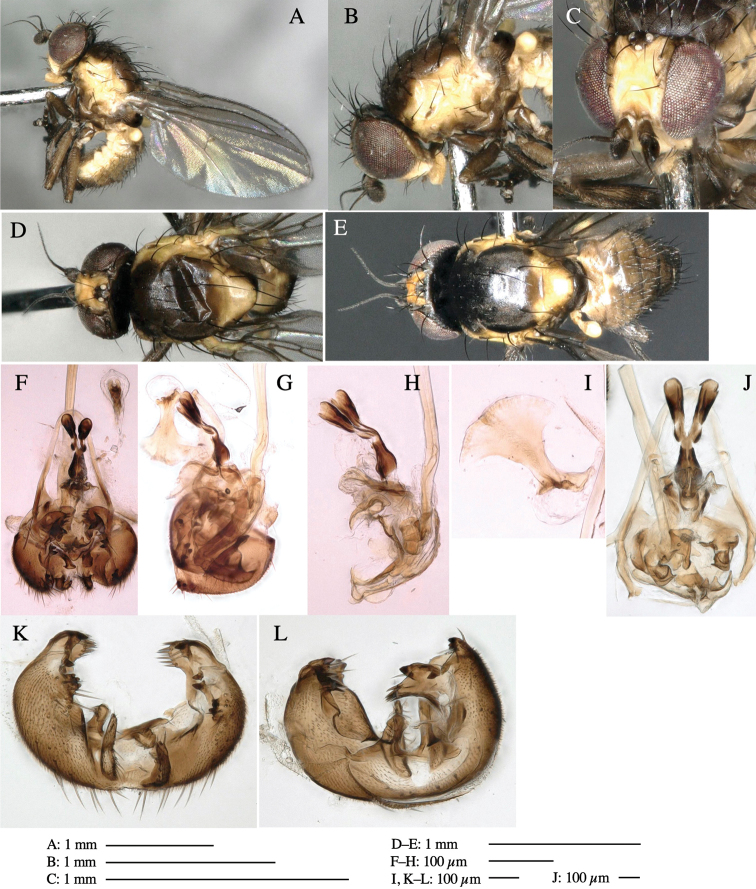
*Phytoliriomyzadumortierae*sp. nov. **A–D **holotype male **A** habitus **B** lateral **C** frontal **D** dorsal **E** paratype female (MK-AG-241) **F–L** male genitalia (**F** at type locality **G–I** at Ichiu **J–L** at Tashiro) **F** whole genitalia, ventral **G** whole genitalia, lateral **H** phallic complex, lateral **I** ejaculatory apodeme, lateral **J** phallic complex, ventral **K–L** epandrium, posterior and anterior.

**Adult male** (Fig. 10A–D).

***Head*:** Head largely light yellow; ocellar triangle yellow but ocellar tubercle dark brown; front-orbital plate brown; back of head brown above foramen excluding margin (Fig. 10C). Antenna porrect, first flagellomere black, pedicel and scape brown. Arista subbasal, pubescent. Face, gena, parafacial and postgena light yellow. Proboscis normal, yellow; palpus brown, cylindrical. ***Chaetotaxy*:** Front orbitals three pairs; one ori directed inward; two ors directed upward (Fig. 10B). Orbital setulae minute and erect, in a single row.

***Thorax*:** Thorax subshiny. Scutum dark brown with a pair of terminal yellow patches adjoining scutellum (Fig. 10D). Scutellum yellow with lateral corner narrowly brown. Mediotergite, katatergite and anatergite dark brown. Pleuron yellow, lower half of anepisternum and anepimeron brown, meron and katepisternum brown (Fig. 10B). Haltere light yellow. Calypter margin and hairs gray. Leg segments brown; tibia and tarsus dark brown. ***Chaetotaxy*:** Scutum with 1+3 dorsocentrals, shortened anteriorly. Acrostichal setulae seven or eight pairs in two rows (Fig. 10D). ***Wing*:** Wing length 1.9&nbsp;mm, costa reaching M_1_ (Fig. 10A). Length of ultimate section of vein M_4_ divided by penultimate section 1.2.

***Abdomen*:** Abdomen dorsally subshiny brown (Fig. 10A). ***Genitalia*:** (Fig. 10F–L) Epandrium dark brown rounded apically, onion-shaped in lateral view (Fig. 10G); inner-anterior margin with two stout tubercle-like setae, and inner-lateral margin with an incomplete comb comprising three short, fused, tubercle-like setae medially (Fig. 10K, L). Surstylus with a row of seven or eight strong setae apically and one or two stout tubercle-like setae posteriorly. Subepandrial sclerite with one pair of long, ventrally directed arms; distal end flattened and expanded (Fig. 10F). Hypandrium sclerotized along outer margin (Fig. 10F). Postgonite bare and goose barnacle-shaped with a pointed apex (Fig. 10J). Phallophorus articulated with phallapodeme, fused to epiphallus (Fig. 10H, J). Basiphallus consisting of a pair of asymmetric dark narrow plate-like sclerites; both protruding ventrally (Fig. 10F–H, J). Hypophallus broad and membranous with lateral margin sclerotized, medially with a pair of short converging sclerites (Fig. 10H, J). Paraphallus narrow, membranous and distally pointed, with distal tip pigmented (Fig. 10F–H, J). Mesophallus dark, turgid, widest subbasally, as long as distiphallus (Fig. 10H). Distiphallus comprising one pair of stout tubules; basal 1/3 composed of dark sclerite basally and weaker medial region; distal 2/3 dark, claviform, tip rounded (Fig. 10H). Ejaculatory apodeme fan-shaped with marginal pale area and broad stalk; base dark and wide to one side; sperm pump clear (Fig. 10I).

**Figure 11.**
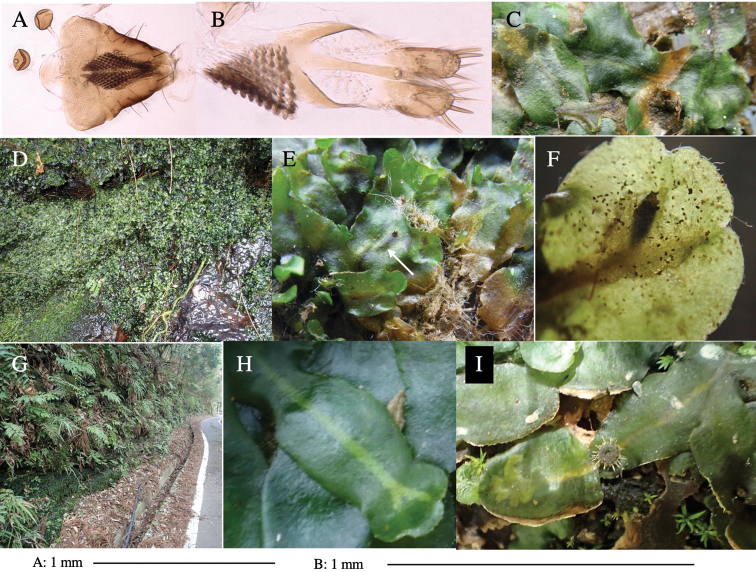
Female morphology and larval ecology of *Phytoliriomyzadumortierae*sp. nov. **A, B **female postabdomen at type locality **A** oviscape and spermatheca **B** tergite 10 **C–I** habitat and the host liverwort *Dumortierahirsuta***C** mined thallus at Gakuenji **D** habitat at type locality **E** mined thallus at type locality (an arrow indicates internal puparium) **F** an internal puparium in thallus at Tazukawa-keikoku **G** habitat at Ichiu, **H, I** mined thalli at Ichiu.

**Female** (Fig. 10E). Similar to male, but slightly larger. Wing length 2.2 mm. ***Postabdomen*:** (Fig. 11A, B) Oviscape dark brown sparsely setigerous (Fig. 11A). Tergite 10 trifurcate, laterally uniting narrow pleural sclerites (Fig. 11B). Each cercus with two stout, apical, trichoid sensilla, 1/3 length of cercus. Spermathecae semi-orbicular, with truncate proximal ends (Fig. 11A).

#### Variation.

The color of frons and scutellum varies from yellow to brownish yellow in some localities.

#### Etymology.

 The specific name refers to its host plant genus *Dumortiera*.

#### Japanese name.

Zangetsu-kezenigoke-hamoguribae.

#### Host plant.

*Dumortierahirsuta* (Dumortieraceae).

#### Mine.

(Fig. 11C, E, F, H, I) Larvae construct linear mines in the midrib of the thallus in early instars, and in the last instar construct radiate or blotch mines by coming out from the midrib, and pupate in the mines.

#### Biological notes.

The main habitats of this species are along streams in warm temperate evergreen forests (Fig. 11D, G). Our rearing records suggest that this species is univoltine, and that adults emerge from overwintered pupae in spring.

#### Distribution.

**Figure 12.**
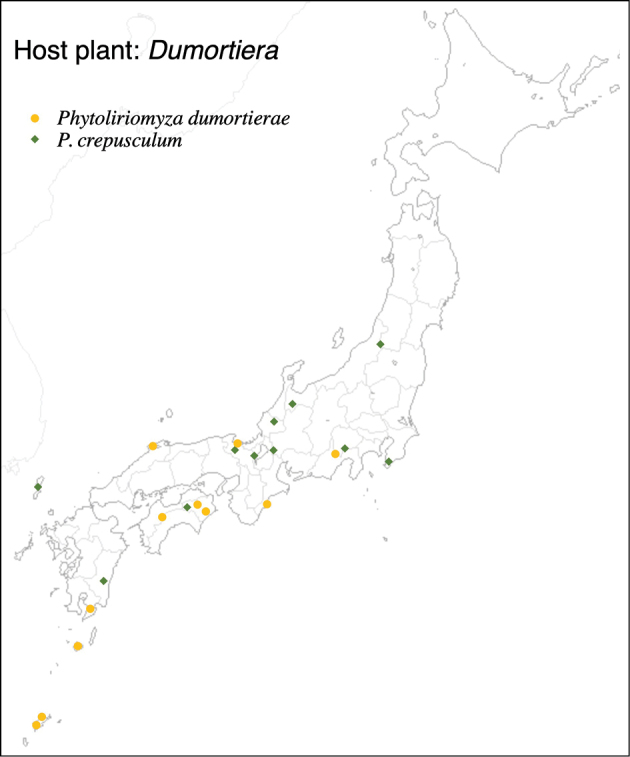
Locality records of three *Phytoliriomyza* species associated with *Dumortiera* spp.: *P.dumortierae*and*P.crepusculum*.

Japan: Honshu, Shikoku, Kyushu, Yaku Island and Amami-oshima Island (Fig. 12).

#### Remarks.

 This species resembles *P.wiesnerellae* in coloration of head, thorax, abdomen, and legs; it is distinguished from the latter by the pleuron with the lower half darkened (pleuron almost wholly yellow in *P.wiesnerellae*). The great morphological difference in male genitalia suggests that these two species are not closely related.

### ﻿7.﻿ Phytoliriomyza crepusculum

Taxon classificationAnimaliaDipteraAgromyzidae

Kato sp. nov.

FC660752-E10E-56E9-A013-4CA0D9B34DC2

https://zoobank.org/421AB9E5-4908-450E-8960-79A097FA8EBA

Fig. 13.

#### Material examined.

***Holotype*:** Japan: 1♂ (MK-AG-a29), Naiku, Oe, Fukuchiyama, Kyoto Pref. (35.433016°N, 135.150397°E, 75 m asl), 17-III-2017 (as larva), emerged on 17-V-2017, NSMT-I-Dip 31929. ***Paratypes*:** Japan: 1♂ (MK-AG-a457), type locality, 19-V-2021 (as larva), on 1-VII-2021, NSMT-I-Dip 31930; 1♂1 ♀ (MK-AG-a399, 274), Inago, Shibakawa, Fujinomiya, Shizuoka Pref., 17-II-2002 (as larva), emerged on 17–23-IV-2002, NSMT-I-Dip 31931, 31932; 1♂ (MK-AG-a309), Mitake, Kamitsushima, Nagasaki Pref., 19-IV-2009 (as larva), emerged on 6-VI-2009, NSMT-I-Dip 31933.

#### Other material.

Japan: 1♀, Sanekawa-keikoku, Iide, Aga, Higashikanbara, Niigata Pref., 3-V-2015 (as larva), emerged on 12-VI -2015; 1♀, Mt. Kiyosumi, Kamogawa, Chiba Pref., 24-I-2012 (as larva), emerged on 25-V–5-VI-2012; 17♂32♀, Inago, Shibakawa, Fujinomiya, Shizuoka Pref., 17-II-2002 (as larva), emerged on 23-IV–5-VI-2021; 1♂, Shogawa-kyo, Tonami, Toyama Pref., 3-V-2016 (as larva), emerged on 9-VI -2016; 1♀, Takeda-gawa, Maruoka, Sakai, Fukui Pref., 6-IV-2002 (as larva), emerged on 13-VI-2019; 2♂2♀, Mt. Nabejiri, Taga, Shiga Pref., 23-V-2015 (as larva), emerged on 26–15-VI-2015; 1♂1♀, Boumura, Kazuragawa, Otsu, Shiga Pref., 7-IV-2002 (as larva), emerged on 24-V-2002; 1♂1♀, Mitake, Kamitsushima, Nagasaki Pref., 9-IV-2009 (as larva), emerged on 6-VI-2009.

#### Diagnosis.

 A medium-sized species (wing length 1.4–1.8 mm) having subshiny brown scutum with brownish yellow pattern extending from mid-posterior margin to scutellum, black 1^st^ flagellomere, brown maxillary palpus, brown halteres, and brownish yellow legs. Male epandrium inner-laterally with a comb comprising five long fused tubercle-like setae. Larva mines the thallus of *Dumortierahirsuta*.

#### Description.

**Figure 13.**
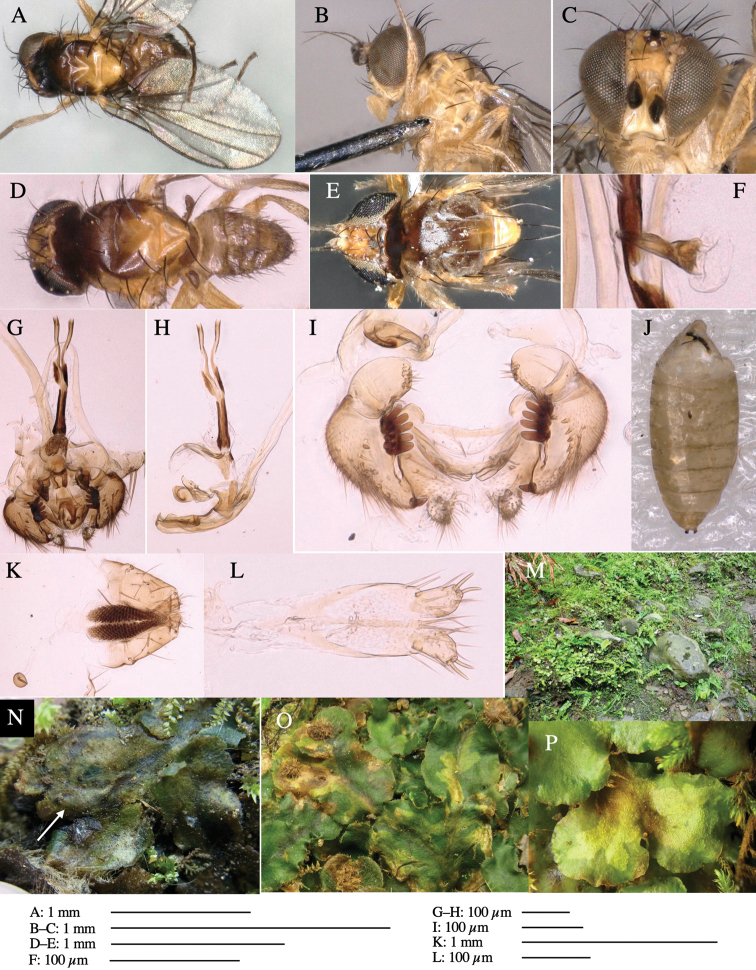
*Phytoliriomyzacrepusculum*sp. nov. **A–C **holotype male **A** habitus **B** lateral **C** frontal **D** paratype male (MK-AG-a309), dorsal **E** paratype female (MK-AG-274), dorsal **F–I** male genitalia **F** ejaculatory apodeme, dorsal **G** whole genitalia, ventral **H** phallic complex, lateral **I** epandrium, ventral **J** puparium **K–L** female postabdomen **K** oviscape and spermatheca **L** tergite 10 **M** habitat of type locality **N–P** mined thalli of *Dumortierahirsuta***(N** type locality **O** Kiyosumi **P** Mt. Nabejiri). An arrow in **N** indicate an puparium.

**Adult male** (Fig. 13A–D).

***Head*:** Head largely light yellow; ocellar triangle yellow but ocellar tubercle dark brown; back of head dark brown above foramen excluding margin (Fig. 13C). Antenna porrect, first flagellomere black, pedicel and scape yellow. Arista subbasal, pubescent. Face, gena, parafacial and postgena light yellow. Proboscis normal, yellow; palpus yellow, cylindrical. ***Chaetotaxy*:** Front orbitals three pairs; one ori directed inward; two ors directed upward (Fig. 13B). Orbital setulae minute and erect, in a single row.

***Thorax*:** Thorax subshiny. Scutum brown with a large posterior trapezoid yellow patch adjacent to scutellum (Fig. 13D). Scutellum yellow with lateral corner narrowly brown. Subscutellum yellow. Mediotergite, katatergite and anatergite brown. Pleuron yellow, but propleuron, meron, anepimeron centrally pale brown (Fig. 13B). Haltere brown. Calypter margin and hairs gray. Leg segments yellow; tibia and tarsus dorsally brown (Fig. 13D). ***Chaetotaxy*:** Scutum with 1+3 dorsocentrals, shortened anteriorly (Fig. 13D). Acrostichal setulae five pairs in two rows. ***Wing*:** Wing length 1.4 mm, costa reaching M_1_ (Fig. 13A). Length of ultimate section of vein M_4_ divided by penultimate section 1.1.

***Abdomen*:** Abdomen dorsally subshiny brown (Fig. 13D). ***Genitalia*:** (Fig. 13F–I) Epandrium dark brown, rounded apically; posterior end of inner margin with a stout tubercle-like seta; inner-ventral surface of epandrium with a comb comprising five fused long tubercle-like setae; inner-ventral margin with a row of several short tubercle-like setae (Fig. 13I). Surstylus rounded, directed inwards, setose apically but without tubercle-like seta. Cercus narrow, setose. Subepandrial sclerite with one pair of posteriorly directed plate-like arms (Fig. 13I). Hypandrium slightly sclerotized along outer margin (Fig. 13G). Postgonite bare, goose barnacle-shaped, narrowly rounded, and cleft apically (Fig. 13H). Phallophorus with deep incision below (Fig. 13G), and articulated with phallapodeme, fused to epiphallus (Fig. 13H). Basiphallus composing of a left anterodorsal sclerite and a right L-shaped lateral sclerite; former narrow, protruding rightward, the latter protruding ventrally (Fig. 13G–H). Hypophallus broad and membranous, supported by one pair of fused sclerites ventrally (Fig. 13G, H). Mesophallus dark, cylindrical, widest subbasally, slightly shorter than distiphallus (Fig. 13H). Distiphallus consists of a pair of moderately long tubules slightly diverging apically; basal half composed of dark narrow lobate sclerite and weaker medial region; distal half cylindrical, dorsally pigmented, with truncated, unpigmented apex (Fig. 13H). Ejaculatory apodeme fan-shaped with short broad stalk, broad base, and clear sperm pump (Fig. 13F).

**Female** (Fig. 13E). Similar to male (Fig. 13E). Wing length 1.4–1.8 mm. ***Postabdomen*:** (Fig. 13K, L) Oviscape brown sparsely setigerous on lateral sides (Fig. 13K). Tergite 10 trifurcate, laterally uniting narrow pleural sclerites (Fig. 13L). Cercus with two stout, apical, trichoid sensilla, ca. 1/2 length of cercus (Fig. 13L). Spermathecae semi-orbicular, with truncate proximal ends (Fig. 13K).

**Immatures.** (Fig. 13J) Puparium internal, slender, and pale brown.

#### Etymology.

The specific name (*crepusculum* = twilight) refers to the brownish yellow patch on the scutum.

#### Japanese name.

Yuuzuki-kezenigoke-hamoguribae.

#### Host plant.

*Dumortierahirsuta* (Dumortieraceae).

#### Mine.

(Fig. 13N–P) Larvae mine the midrib of the thallus in early instars, later construct radiate mines by coming out from the midrib, and pupate in the mines. Puparium is smooth and pale brown, posterior spiracles projecting (Fig. 13J).

#### Biological notes.

 The main habitats of this species are along streams in warm temperate forests and cool temperate deciduous forests (Fig. 13M), and this species was sympatric with *P.dumortierae* in some localities. Our rearing records suggest that this species is univoltine, and that adults emerge from overwintered pupae in spring.

#### Distribution.

Japan: Honshu, Shikoku, Kyushu, Tsushima Island (Fig. 12).

#### Remarks.

 Although this species used the same host plant as *P.dumortierae*, these two species were not sympatric in any localities. This species resembles *P.arcus*,*P.plagiochasmatos* and *P.falcata* in having a pair of brown lateral bands on the scutum; it is distinguished from them by the brownish yellow color of the medial mark on scutum (light yellow in the other species).

##### Species associated with *Plagiochasma*

### ﻿8.﻿ Phytoliriomyza arcus

Taxon classificationAnimaliaDipteraAgromyzidae

Kato sp. nov.

4C17E118-26BD-51A0-A447-FB381DE23DF4

https://zoobank.org/89D711DD-A5C8-4A52-8266-23A5C33BCD92

Figs 14 , 15

#### Material examined.

***Holotype*:** Japan: 1♂ (MK-AG-a411), Nippara, Okutama, Tokyo ﻿Pref. (35.8504°N, 139.0274°E, 650 m asl), 15-III-2016 (as larva on *P.pterospermum*), emerged on 28-IV-2016, NSMT-I-Dip 31934. ***Paratypes*:** Japan: 1♂1♀ (MK-AG-a16, a458), same data as holotype, emerged on 4–5-V-2016, NSMT-I-Dip 31935, 31937; 1♀ (MK-AG-191), Akka, Iwaizumi, Iwate Pref., 5-V-2012 (as larva on *P.pterospermum*), emerged on 13-V-2012, NSMT-I-Dip 31936; 1♀ (MK-AG-a316), Mt. Myogi, Tomioka, Gunma Pref., 10-V-2021 (as larva on *P.pterospermum*), emerged on 16-V-2021, NSMT-I-Dip 31938; 1♀ (MK-AG-189), Todai-shiraiwa, Ina, Nagano Pref., 30-IV-2011 (as larva on *P.pterospermum*), emerged on 28-V-2011, NSMT-I-Dip 31939.

#### Other material.

 Japan: On *Plagiochasmapterospermum*: 4♂4♀, Akka, Iwaizumi, Iwate Pref., 5-V-2012 (as larva), emerged on 27–28-V-2012; 9♂9♀, Mt. Myogi, Tomioka, Gunma Pref., 10-V-2021 (as larva), emerged on 14–21-V-2021; 10♂12♀, Narahara, Ueno, Tano, Gunnma Pref., 18-IV-2021 (as larva), emerged on 15–25-V-2021; 2♂5♀, Mt. Kano, Kanna, Tano, Gunma Pref., 28-XI-2014 (as larva), emerged on 4-V-2015; 2♂1♀, Mt. Futago, Ogano, Chichibu-gun, Saitama Pref., 10-IX-2017 (as larva), emerged on 20-X–12-XI-2017; 1♂3♀, Kanna-gawa, Nakatsugawa, Chichibu, Saitama Pref., 16-X-2012 (as larva), emerged on 5-V-2012; 9♂11♀, Todai-shiraiwa, Ina, Nagano Pref., 12-V-2021 (as larva), emerged on 1–13-VI-2021.

On *Plagiochasmaappendiculatum*: 5♂4♀, Mt. Myogi, Tomioka, Gunma Pref., 10-V-2021 (as larva), emerged on 15–29-V-2021.

#### Diagnosis.

 A small species (wing length 1.3–1.6 mm) having subshiny brown scutum with yellow pattern extending from mid-posterior margin to scutellum, yellow 1^st^ flagellomere, yellow maxillary palpus, yellow halteres, and yellow legs. Male epandrium inner-subdistally with hypertrophied, elongated, strongly curved arm bearing a dark tubercle-like seta. Larva mines the thallus of *Plagiochasmapterospermum* and *P.appendiculatum*.

#### Description.

**Figure 14.**
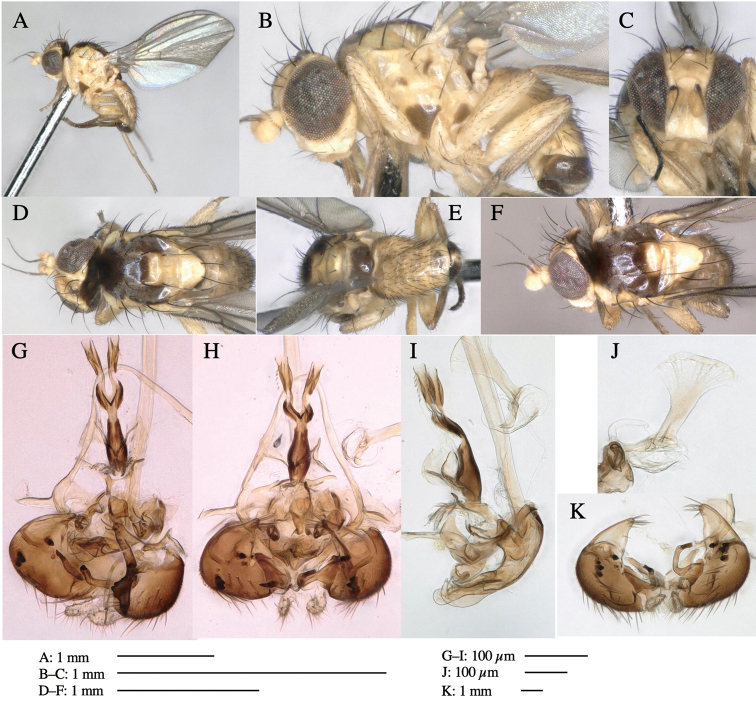
*Phytoliriomyzaarcus*sp. nov. **A–E **holotype male **A** habitus **B** lateral **C** frontal **D** dorsal **E** posterior **F** paratype female (MK-AG-a16), dorsal **G–K** male genitalia (**G** at type locality **H–K** at Todai-shiraiwa) **G, H** whole genitalia, ventral **I** phallic complex, lateral **J **ejaculatory apodeme, lateral **K** epandrium, ventral.

**Adult male** (Fig. 14A–E).

***Head*:** Head light yellow, with ocellar tubercle brown, back of head dark brown (Fig. 14C). Antenna porrect, first flagellomere, pedicel and scape brown. Arista subbasal, black pubescent. Clypeus, face, gena, parafacial and postgena yellow. Proboscis normal, yellow; yellow, cylindrical (Fig. 14C). ***Chaetotaxy*:** Front orbitals three pairs; one ori directed inward; two ors directed upward (Fig. 14B). Orbital setulae minute and erect, in a single row.

***Thorax*:** Thorax subshiny. Scutum subshiny yellow, with a brown medial stripe on anterior 2/3, one pair of brown presutural patches, and a pair of wide postsutural brown bands adjoining with the presutural patches (Fig. 14D). Mediotergite brown, and anatergite and katatergite yellow. Pleuron largely yellow, anepisternum and anepimeron with small brown patches, venters of katepisternum and meron brown (Fig. 14B). Haltere gray. Calypter margin and hairs gray. Leg segments yellow; tibia and tarsus darker (Fig. 14A). ***Chaetotaxy*:** Scutum with 1+3 dorsocentrals, shortened anteriorly. Acrostichal setulae six pairs in two rows (Fig. 14D). ***Wing***: Wing length 1.3&nbsp;mm, costa reaching M_1_ (Fig. 14A). Length of ultimate section of vein M_4_ divided by penultimate section 1.7–1.8.

***Abdomen*:** Abdomen dorsally subshiny grayish yellow (Fig. 14E). ***Genitalia*:** (Fig. 14G–K) Epandrium dark brown, rounded apically; inner-lateral margin with an incomplete comb comprising two or three short tubercle-like setae; inner subdistal surface with basally thickened, remarkably elongated, strongly curved arm bearing a dark tubercle-like seta apically (Fig. 14G, H, K). Surstylus protruding ventrally, tapering toward apex, setose apically, with one short tubercle-like seta on inner-basal surface (Fig. 14K). Cercus narrow, setose. Subepandrial sclerite V-shaped in posterior view, with a pair of ventrally projected plate-like lobes with a spine medially (Fig. 14K). Hypandrium slightly sclerotized along outer margin (Fig. 14G). Postgonite bare, goose barnacle-shaped, and cleft apically (Fig. 14G). Phallophorus with deep incision below, articulated with phallapodeme, fused to epiphallus (Fig. 14I). Basiphallus with a H-shaped dorsal sclerite; anterior arms of which protruding ventrolaterally (Fig. 14G–I). Hypophallus broad, membranous with microtrichia scattered dorsally, medially with a pair of short confronting sclerites (Fig. 14G–I). Paraphalli wing-like, lightly sclerotized posteriorly; diverging, angled anteroventrally, joined basally on to the base of mesophallus. Mesophallus dark, cylindrical, widest subbasally, as long as distiphallus (Fig. 14G–I). Distiphallus comprising one pair of stout tubules; basal half composed of lateral dark sclerites and weaker medial region; distal half cylindrical, composed of a pair of dorsally and laterally pigmented sclerites, widening toward apex, with truncated, flared apex (Fig. 14I). Ejaculatory apodeme pale and fan-shaped with broad stalk; base wide to one side; sperm pump clear.

**Figure 15.**
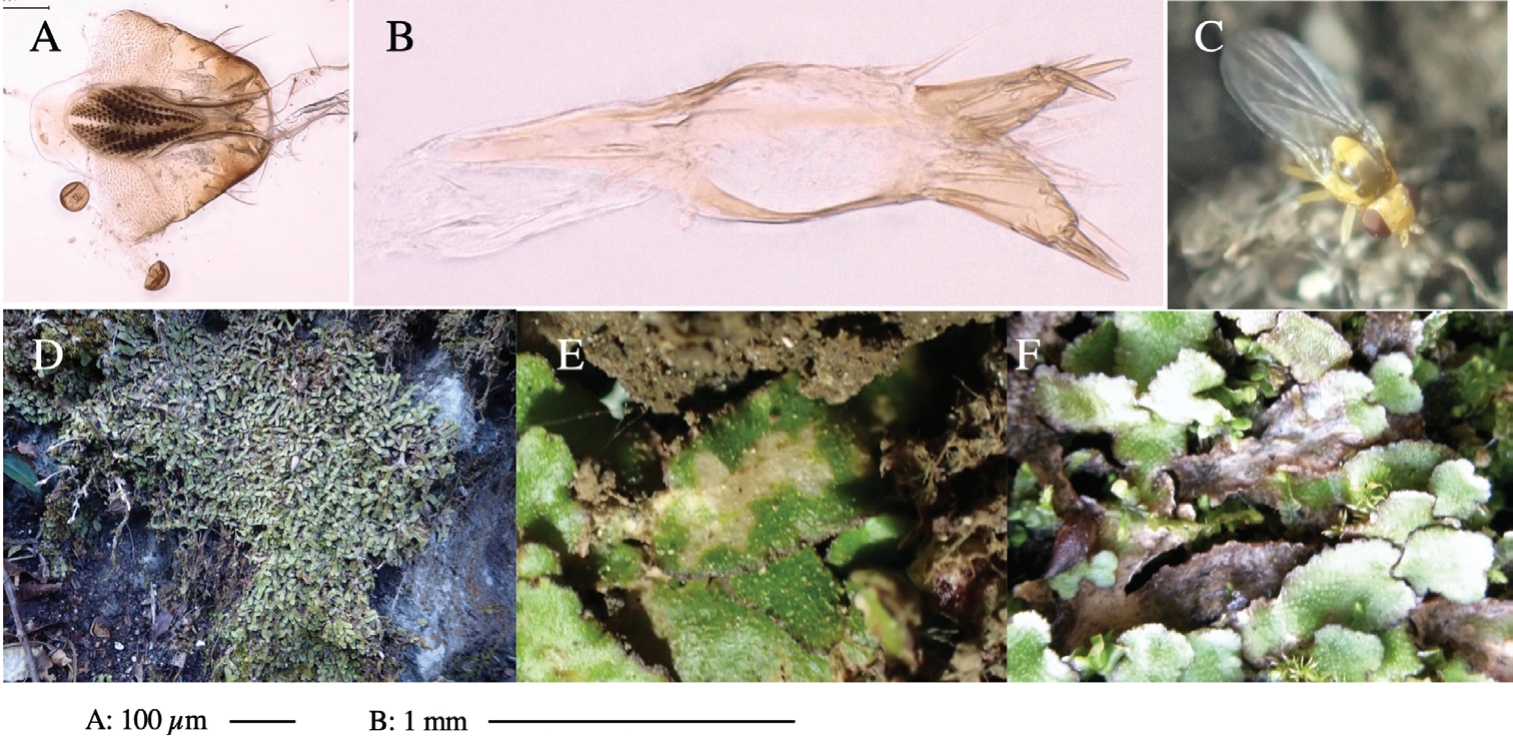
Female morphology and larval/adult ecology of *Phytoliriomyzaarcus*sp. nov. **A, B **female postabdomen **A** oviscape and spermatheca at Shiraizasu **B** tergite 10 at Mt. Myogi **C** live fly at Mt. Myogi **D** habitat at type locality **E** mined thallus of *Plagiochasmapterospermum* at Mt. Myogi **F** mined thalli at Todai-shiraiwa.

**Female** (Figs 14F, 15C). Similar to male, but slightly larger (Fig. 14F). Wing length 1.6 mm. ***Postabdomen*:** (Fig. 15A, B) Oviscape dark brown, setigerous (Fig. 15A). Tergite 10 trifurcate, laterally uniting narrow pleural sclerites (Fig. 14B). Each cercus with two stout, apical, trichoid sensilla, 1/3 length of cercus (Fig. 14B). Spermathecae semi-orbicular, with truncate proximal ends (Fig. 14A).

#### Variation.

The yellow posterior patch on the scutum varied from distinct to obscure ones among localities and even among individuals in some localities.

#### Etymology.

The specific name (*arcus* = bow) refers to the bow-shaped tubercle-like seta on the male epandrium.

#### Japanese name.

Yumihari-tsubozenigoke-hamoguribae.

#### Host plant.

*Plagiochasmapterospermum* and *P.appendiculatum* (Aytoniaceae).

#### Mine.

Larvae construct linear-blotch mines in the thallus and pupate in the mines (Fig. 15D–F).

#### Biological notes.

The habitats of this species are limestone outcrops in temperate deciduous forests, where the host liverworts grow (Fig. 15D). Our rearing records suggest that this species is univoltine, and that adults emerge from overwintered pupae in spring.

#### Distribution.

**Figure 16.**
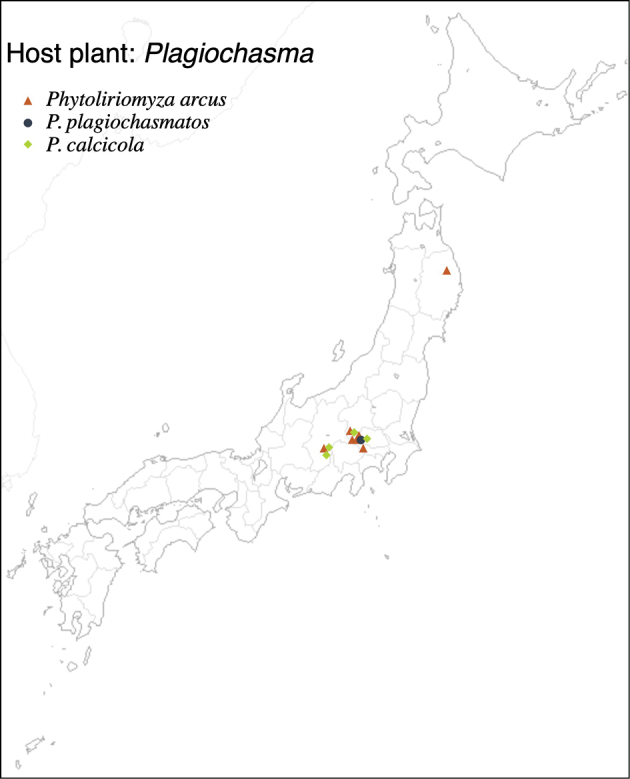
Locality records of three *Phytoliriomyza* species associated with *Plagiochasma* spp.: *P.arcus*, *P.plagiochasmatos*and*P.calcicola*.

Japan: Honshu (Fig. 16). Recorded only from limestone areas in Tohoku and Kanto districts.

#### Remarks.

 This species resembles *P.plagiochasmatos* and *P.falcata* in having a pair of brown lateral bands and a light yellow mark on scutum; it is distinguished from them by the dark haltere (yellow in these species) and by the morphology of the tubercle-like seta on subdistal margin of the male epandrium (curved outward in *P.arcus*; simple and short in *P.plagiochasmatos*; elongated and sickle-like in *P.falcata*).

### ﻿9.﻿ Phytoliriomyza plagiochasmatos

Taxon classificationAnimaliaDipteraAgromyzidae

Kato sp. nov.

8B297B08-1B72-5A07-A3AA-B87620677330

https://zoobank.org/7802A55A-6207-4BEA-AC06-B6E83CE97698

Fig. 17

#### Material examined.

***Holotype*:** Japan: 1♂ (MK-AG-a526), Narahara, Ueno, Tano, Gunnma Pref. (36.089°N,138.689°E, 990 m asl), 18-IV-2021 (as larva), emerged on 15-V-2021, NSMT-I-Dip 31940. ***Paratypes*:** Japan: 1♂1♀ (MK-AG-a524, a523), same data as holotype, emerged on 19–25-V-2021, NSMT-I-Dip 31941, 31942.

#### Other material.

 Japan: 1♂3♀, same data as holotype, emerged on 19–25-V-2021; 3♀, Ozasu, Ogano, Chichibu, Saitama Pref., 28-XI-2014 (as larva on *Asterellacruciata*), emerged on 24–27-IV-2015.

#### Diagnosis.

 A small species (wing length 1.4–1.5 mm) having subshiny brown scutum with an oval yellow pattern extending from mid-posterior margin to scutellum, yellow 1^st^ flagellomere, yellow maxillary palpus, yellow halteres, and yellow legs. Male epandrium with an imperfect comb comprising two short tubercle-setae. Larva mines the thallus of *Plagiochasmapterospermum* and *Asterellacruciata*.

#### Description.

**Figure 17.**
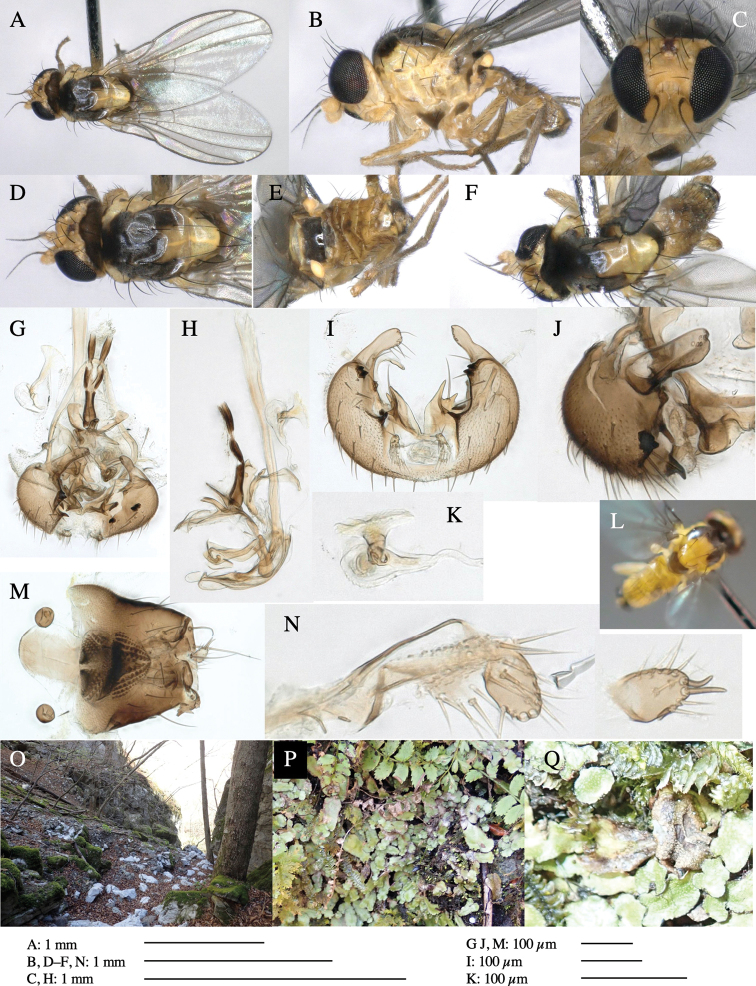
*Phytoliriomyzaplagiochasmatos*sp. nov. **A–E **holotype male **A** habitus **B** lateral **C** frontal **D** dorsal** E** posterior **F** paratype female (MK-AG-a523), dorsal **G–J **male genitalia **G** whole genitalia, ventral** H** phallic complex, lateral **I, J** epandrium, ventral and anterior **K** ejaculatory apodeme, lateral **L** live male fly.

**Adult male** (Fig. 17A–E).

***Head*:** Head light yellow, with ocellar tubercle brown, back of head dark brown (Fig. 17C). Antenna porrect, first flagellomere, pedicel and scape brown. Arista subbasal, black pubescent. Clypeus, face, gena, parafacial and postgena yellow. Proboscis normal, yellow; palpus yellow, cylindrical (Fig. 17C). ***Chaetotaxy*:** Front orbitals three pairs; one ori directed inward; two ors directed upward (Fig. 17B). Orbital setulae minute and erect, in a single row.

***Thorax*:** Thorax subshiny. Scutum subshiny yellow, with a brown medial stripe on anterior 2/3, one pair of brown presutural patches, and a pair of wide postsutural brown bands (Fig. 17B). Mediotergite brown, and anatergite and katatergite yellow. Pleuron largely yellow, anepisternum and anepimeron with small brown patches, venters of katepisternum and meron brown (Fig. 17B). Haltere yellow. Calypter margin and hairs gray. Leg segments yellow; tibia and tarsus darker (Fig. 17B). ***Chaetotaxy*:** Scutum with 1+3 dorsocentrals, shortened anteriorly (Fig. 17D). Acrostichal setulae six pairs in two rows. ***Wing*:** Wing length 1.5 mm, costa reaching M_1_ (Fig. 17A). Length of ultimate section of vein M_4_ divided by penultimate section 1.7.

***Abdomen*:** Abdomen dorsally subshiny grayish yellow (Fig. 17E). ***Genitalia*:** (Fig. 17I–K) Epandrium dark brown, rounded apically; inner-subdistal margin with a short, sharp-pointed tubercle-like seta; inner-anterior margin with an imperfect comb comprising two fused short tubercle-like setae; inner-lateral surface with a long tubercle-like seta. Surstylus extruded ventrally, setose apically (Fig. 17I, J). Cercus narrow, setose. Subepandrial sclerite comprising two pairs of developed plate-like arms; ventral arm with a strong seta directed ventrally and pointed tip; dorsal arm ventrally pointed and with a spine directed outward (Fig. 17I). Hypandrium slightly sclerotized along outer margin (Fig. 17G). Postgonite bare, goose barnacle-shaped (Fig. 17H). Phallophorus with deep incision below (Fig. 17G), articulated with phallapodeme, fused to epiphallus (Fig. 17H). Basiphallus as long as mesophallus, sclerotized basally and narrowly on right side, bifurcating apically (Fig. 17H). Hypophallus broad, membranous and bilobed, with serrated margins; medially with a pair of dark fused sclerites (Fig. 17G, H). Paraphalli dark, plate-like, diverging from base of mesophallus, outer margins sclerotized (Fig. 17G, H). Mesophallus dark, cylindrical, widest subbasally, 3/5 as long as distiphallus (Fig. 17H). Distiphallus comprising one pair of stout tubules; basal half composed of lateral lanceolate dark sclerites and short weaker region; distal half cylindrical, dorsally and laterally pigmented, with truncated, unpigmented apex (Fig. 17H). Ejaculatory apodeme pale and fan-shaped with broad stalk; base wide to one side; sperm pump clear (Fig. 17K).

**Female** (Fig. 17F). Similar to male, but slightly larger. Wing length 1.4 mm. ***Postabdomen*:** (Fig. 17L, M) Oviscape dark brown, setigerous. Tergite 10 trifurcate, laterally uniting narrow pleural sclerites. Each cercus with two stout, apical, trichoid sensilla, 2/3 length of cercus. Spermathecae semi-orbicular, with truncate proximal ends.

#### Etymology.

 The specific name refers to the host plant genus *Plagiochasma*.

#### Japanese name.

Tsukikage-tsubozenigoke-hamoguribae.

#### Host plant.

*Plagiochasmapterospermum* (Aytoniaceae) and *Asterellacruciata* (Asterellaceae).

#### Mine.

Larvae construct linear-blotch mines in the thallus and pupate in the mines (Fig. 17Q).

#### Biological notes.

 The habitats of this species are outcrops of lime stones in temperate deciduous forests, where the host liverworts grow (Fig. 17O, P), and this species was sympatric with *P.arcus* in some localities. Our rearing records suggest that this species is univoltine, and that adults emerge from overwintered pupae in spring.

#### Distribution.

Japan: Honshu (Fig. 16). Recorded only from limestone areas in Kanto districts.

#### Remarks.

 This species resembles *P.arcus*,*P.falcata* and *P.aratriformis* in having a pair of brown lateral bands and a pale yellow mark on the scutum; it is distinguished from *P.arcus* by the yellow halteres (dark in *P.arcus*), and from the last three species by the absence of seta on the surstylus of the male epandrium (surstylus apically setose in *P.falcata* and *P.aratriformis*).

### ﻿10.﻿ Phytoliriomyza calcicola

Taxon classificationAnimaliaDipteraAgromyzidae

Kato sp. nov.

F314FCA4-05C8-5746-9E14-44F23EF714F3

https://zoobank.org/B8B5E03D-C972-451F-8AD4-ED1237A9ABD2

Figs 18 , 19

#### Material examined.

***Holotype*:** Japan: 1♂ (MK-AG-a265), Todai-shiraiwa, Ina, Nagano Pref. (35.7723°N,138.1620°E, 1140 m asl), 30-IV-2011 (as larva), emerged on 24-V-2011, NSMT-I-Dip 31943. ***Paratypes*:** Japan: 1♀ (MK-AG-a459), same data as holotype emerged on 2-VI-2011, NSMT-I-Dip 31944; 1♀ (MK-AG-a222), Ozasu, Ogano, Chichibu, Saitama Pref., 10-IX-2017 (as larva), emerged on 13-X-2017, NSMT-I-Dip 31945.

#### Other material.

Japan: 1♀, Narahara, Ueno, Tano, Gunnma Pref., 18-IV-2021 (as larva), emerged on 1-VI-2021; 1♀1♂, Ozasu, Ogano, Chichibu, Saitama Pref., 10-IX-2017 (as larva), emerged on 9–13-X-2017; 1♀, Irisawai, Oshika, Nagano Pref., 29-IV-2011 (as larva), emerged on 22-V-2011.

#### Diagnosis.

 A medium-sized yellow species (wing length 1.6–1.7 mm) having subshiny yellow scutum with two pairs of lateral stripes, yellow 1^st^ flagellomere, yellow maxillary palpus, yellow halteres, and yellow legs. Male epandrium inner-subdistally with a hypertrophied arm which bears an enlarged tubercle-like seta; inner-basally with a hypertrophied arm which bears a comb comprising five or six fused tubercle-like setae. Larva mines the thallus of *Plagiochasmapterospermum*.

#### Description.

**Figure 18.**
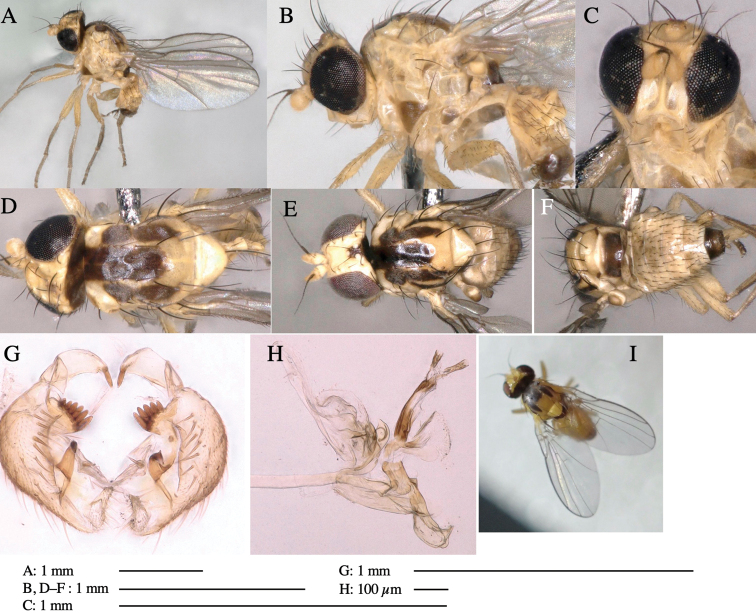
*Phytoliriomyzacalcicola*sp. nov. **A–D **holotype male **A** habitus **B** lateral **C** frontal **D** dorsal** E, F** paratype female (MK-AG-a222) **E** dorsal **F** posterior **G, H** male genitalia **G** epandrium **H** phallic complex in lateral view **I** live female fly.

**Adult male** (Fig. 18A–D).

***Head*:** Head light yellow, with ocellar tubercle brown, back of head dark brown (Fig. 18C). Antenna porrect, first flagellomere, pedicel and scape yellow (Fig. 18B). Arista subbasal, black pubescent. Clypeus, face, gena, parafacial and postgena yellow. Proboscis normal, yellow; palpus yellow, cylindrical (Fig. 18C). ***Chaetotaxy*:** Front orbitals three pairs; one ori directed inward; two ors directed upward (Fig. 18B). Orbital setulae minute and erect, in a single row.

***Thorax*:** Thorax subshiny. Scutum yellow with medial black stripe on anterior 2/3, with a pair of narrow black supra-alar stripes and a pair of wider. Black intra-alar stripes, which adjoin a pair of lateral presutural black ovoid spots (Fig. 18D). Mediotergite brown, and anatergite and katatergite yellow. Pleuron largely yellow, anepisternum and anepimeron with small brown patches, venters of katepisternum and meron brown (Fig. 18B). Haltere yellow, with upper surface grayish. Calypter margin and hairs gray. Leg segments yellow; tibia and tarsus darker (Fig. 18A). ***Chaetotaxy*:** Scutum with 1+3 dorsocentrals, shortened anteriorly (Fig. 18D). Acrostichal setulae seven pairs in two rows. ***Wing***: Wing length 1.7 mm, costa reaching M_1_ (Fig. 18A). Length of ultimate section of vein M_4_ divided by penultimate section 0.95.

***Abdomen*:** Abdomen dorsally subshiny yellow (Fig. 18B). ***Genitalia*:** (Fig. 18I, J) Epandrium dark brown, rounded apically; inner-distal margin with a basally enlarged, slightly flattened hypertrophied arm, which bears a dark laterally enlarged tubercle-like seta; inner-anterior surface also with a basally enlarged, slightly flattened, hypertrophied arm, on which with a comb comprising five or six fused tubercle-like setae (Fig. 18G). Surstylus plate-like, extruded ventrally, bare, with one long tubercle-like seta apically (Fig. 18G). Cercus narrow, setose. Subepandrial sclerite V-shaped in posterior view, with a pair of setae medially (Fig. 18G). Hypandrium slightly sclerotized along outer margin. Postgonite bare and goose barnacle-shaped with incurved pointed apex (Fig. 18H). Phallophorus with deep incision below, articulated with phallapodeme, fused to epiphallus (Fig. 18H). Basiphallus shorter than mesophallus. Hypophallus broad, membranous, with a dark narrow sclerite medially, and with a pair of diverging, ventrally incurved narrow sclerites (Fig. 18H). Paraphallus absent. Mesophallus dorsoventrally flattened tubular, parallel-sided; twofold longer than distiphallus; basal half and distal 1/5 pigmented (Fig. 18H). Distiphallus comprising one pair of tubules; basal half unpigmented; distal half cylindrical, pigmented, with truncated, unpigmented apex (Fig. 18H).

**Figure 19.**
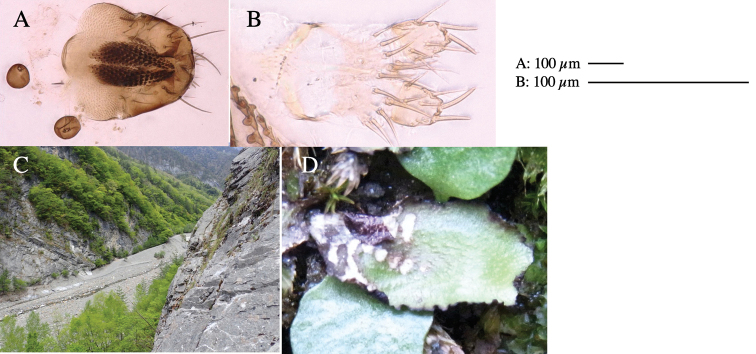
Female morphology and larval/adult ecology of*Phytoliriomyzacalcicola*sp. nov. **A, B **female postabdomen **A** oviscape and spermatheca **B** tergite 10 **C** habitat at type locality **D** mined thallus of *Plagiochasmapterospermum*.

**Female** (Fig. 18E–F). Similar to male, but slightly larger and frons wider. Wing length 1.6 mm. ***Postabdomen*:** (Fig. 19A, B) Oviscape dark brown, setigerous (Fig. 19A). Tergite 10 trifurcate, laterally uniting narrow pleural sclerites. (Fig. 19B) Each cercus with two stout, apical, trichoid sensilla, ¾ length of cercus (Fig. 19B). Spermathecae orbicular.

#### Etymology.

The specific name (*calcis* = limestone) refers to the fact that this species is associated with the host liverwort growing only on limestone.

#### Japanese name.

Kurosuji-tsubozenigoke-hamoguribae.

#### Host plant.

*Plagiochasmapterospermum* (Aytoniaceae).

#### Mine.

Larvae construct radiate mines in the thallus and pupate in the mines (Fig. 19D).

#### Biological notes.

 The habitats of this species are limestone outcrops in temperate deciduous forests, where the host liverworts grow (Fig. 19C). This species is one of the rarest species in liverwort-associated species, and was sympatric with *P.arcus* or *P.plagiochasmatos* in some localities. Our rearing records suggest that this species is univoltine, and that adults emerge from overwintered pupae in spring.

#### Distribution.

Japan: Honshu (Fig. 16). Recorded only from limestone areas in Chichibu Mountains and Akaishi Mountain Range.

#### Remarks.

 This species has several unique characteristics in male genitalia: a flattened stout tubercle-like seta on distal margin of epandrium; a comb of tubercle-like setae borne on enlarged projection of inner surface of epandrium; thin, bare surstylus; short distiphallus unpigmented at basal half. The unique characteristics suggest distant relation of this species from other liverwort-associated species. This species resembles *P.nigroflava* and *P.brunofasciata* in having two pairs of dark stripes on dorsal scutum; it is distinguished from them by the dark haltere (haltere yellow in *P.nigroflava* and *P.brunofasciata*).

##### Species associated with *Asterella*

### ﻿11.﻿ Phytoliriomyza iriomotensis

Taxon classificationAnimaliaDipteraAgromyzidae

Kato sp. nov.

7C29E49E-CD8D-5E1E-8DF8-87380F61E994

https://zoobank.org/FDCB8114-D244-48A4-A86F-D4A04D22AC21

Fig. 20

#### Material examined.

***Holotype*:** Japan: 1♂ (MK-AG-161), Yutsun, Iriomote-Is. Yaeyama, Okinawa Pref. (24.379°N, 123.883°E, 15 m asl), 25-I-2011 (as larva), emerged on 23-IV-2011, NSMT-I-Dip 31949. ***Paratypes*:** Japan: 2♀ (MK-AG-164, a426), same data as holotype, emerged on 2–10-III-2011, NSMT-I-Dip 31950, 31951; 1♂1♀ (MK-AG-a591, a592), type locality, 18-XI-2021 (as larva), emerged on 16–21-XI-2021, NSMT-I-Dip 31952, 31953.

#### Other material.

Japan: 1♀, Yutsun, Iriomote-Is. Yaeyama, Okinawa Pref., 8-XI-2021 (as larva), emerged on 17-XI-2021.

#### Diagnosis.

 A small species (wing length 1.4–1.7 mm) having pruinose gray scutum and scutellum, brown 1^st^ flagellomere, yellow maxillary palpus, dark halteres, and yellow legs. Scutum lacks acrostichal setulae. Male epandrium lacks tubercle-like seta; distiphallus of male genitalia tapering apically. Larva mines the thallus of *Asterellaliukiuensis*.

#### Description.

**Figure 20.**
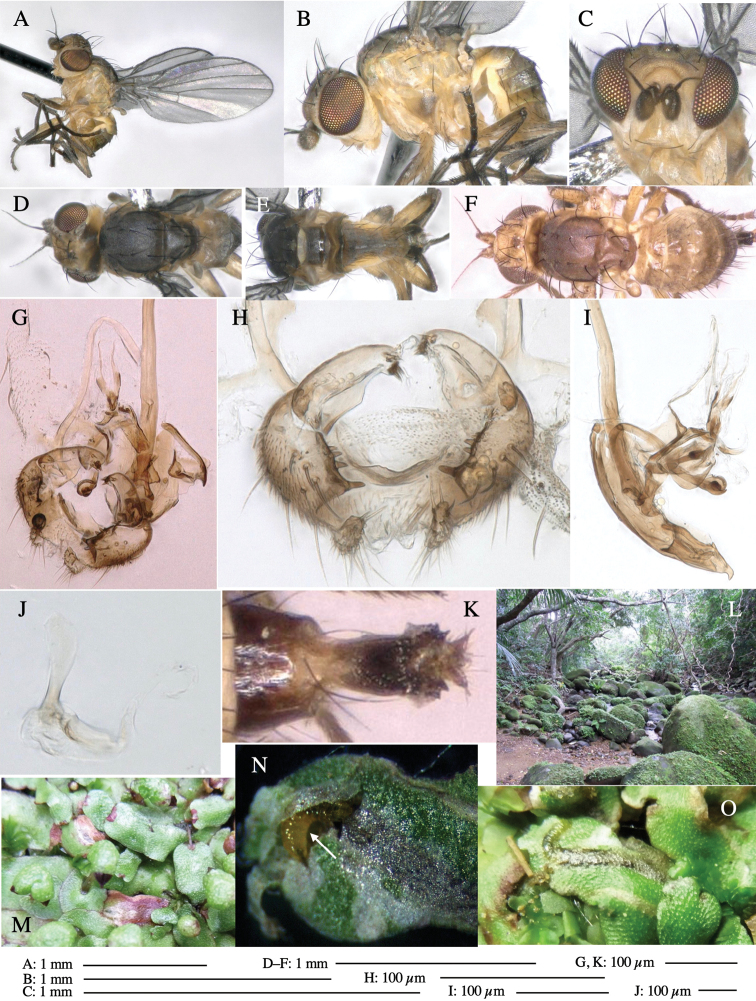
*Phytoliriomyzairiomotensis*sp. nov. **A–E **holotype male **A** habitus **B** lateral **C** frontal **D** dorsal **E** posterodorsal **F** paratype female, dorsal (MK-AG-426) **G–J** male genitalia **G** whole genitalia, ventral **H** epandrium, ventral **I** phallic complex, lateral **J** ejaculatory apodeme, lateral **K** female postabdomen **L** habitat at type locality **M–O** mined thalli of *Asterellaliukiuensis*(an arrow indicates a larva).

**Adult male** (Fig. 20A–E).

***Head*:** Head light yellow, with ocellar tubercle brown, frons brownish yellow, back of head dark brown (Fig. 20C). Antenna porrect, first flagellomere, pedicel and scape brown (Fig. 20B). Arista subbasal, black pubescent. Clypeus, face, gena, parafacial and postgena light yellow. Proboscis normal, light yellow; palpus light yellow, cylindrical (Fig. 20C). ***Chaetotaxy*:** Front orbitals three pairs; one ori directed inward; two ors directed upward (Fig. 20D). Orbital setulae minute and erect, in a single row.

***Thorax*:** Thorax pruinose. Scutum and scutellum dark brown (Fig. 20B). Mediotergite brown, anatergite with circular brown patches on venter, and katatergite yellow (Fig. 20E). Pleuron largely pale yellow, katepisternum and meron with large brown patches on venter (Fig. 20B). Haltere brown with pale yellow stalk. Calypter margin and hairs gray. Leg segments dark yellow; tibia and tarsus darker (Fig. 20A). ***Chaetotaxy*:** Scutum with 1+3 dorsocentrals, shortened anteriorly (Fig. 20D). Acrostichal setulae lacking. ***Wing*:** Wing length 1.4 mm, costa reaching M_1_ (Fig. 20A). Length of ultimate section of vein M_4_ divided by penultimate section 2.0–2.4. M_4_ disappear before reaching wing margin.

***Abdomen*:** Abdomen dorsally subshiny yellowish brown (Fig. 20E). ***Genitalia*:** (Fig. 20G–J) Epandrium dark brown, rounded apically; posterior end of inner margin with a tubercle-like seta; inner-lateral margin with three tubercle-like setae (Fig. 20H). Surstylus elongated, broad basally, sparsely setose apically (Fig. 20H). Cercus narrow, setose. Subepandrial sclerite comprising a pair of plate-like arms (Fig. 20H). Hypandrium slightly sclerotized along outer margin (Fig. 20G). Postgonite bare, goose barnacle-shaped, cleft apically; upper lobe pointed apically, lower lobe rounded (Fig. 20I). Phallophorus with deep incision below, articulated with phallapodeme, fused to epiphallus (Fig. 20G, I). Basiphallus longer than mesophallus, dorsally sclerotized (Fig. 20G). Hypophallus broad, membranous, medially with a pair of fused sclerites (Fig. 20G, I). Paraphallus absent. Mesophallus short, sclerotized dorsally and ventrally in basal 2/3; shorter than distiphallus (Fig. 20G, I). Distiphallus comprising one pair of tubules; basal half dorsally pigmented, with small ventral sclerite basally, distal half unpigmented and tapering toward tip (Fig. 20G, I). Ejaculatory apodeme pale and narrow fan-shaped with long stalk; base wide to one side; sperm pump clear (Fig. 20J).

**Female** (Fig. 20F). Similar to male, but slightly larger; scutum paler, abdomen dorsally paler, and legs paler than male. Wing length 1.7 mm. ***Postabdomen*:** (Fig. 20K) Oviscape dark brown, setigerous. Each cercus with two stout, apical, trichoid sensilla, 1/3 length of cercus.

#### Etymology.

The specific name refers to the type locality, Iriomote Island.

#### Japanese name.

Okinawasaihaigoke-hamoguribae.

#### Host plant.

*Asterellaliukiuensis* (Aytoniaceae).

#### Mine.

Larvae construct linear-blotch mines in the thallus in early instars, later entering the midrib, and pupating there (Fig. 20M–O).

#### Biological notes.

The habitats of this species are rocky stream banks in subtropical evergreen forests (Fig. 20L).

#### Distribution.

**Figure 21.**
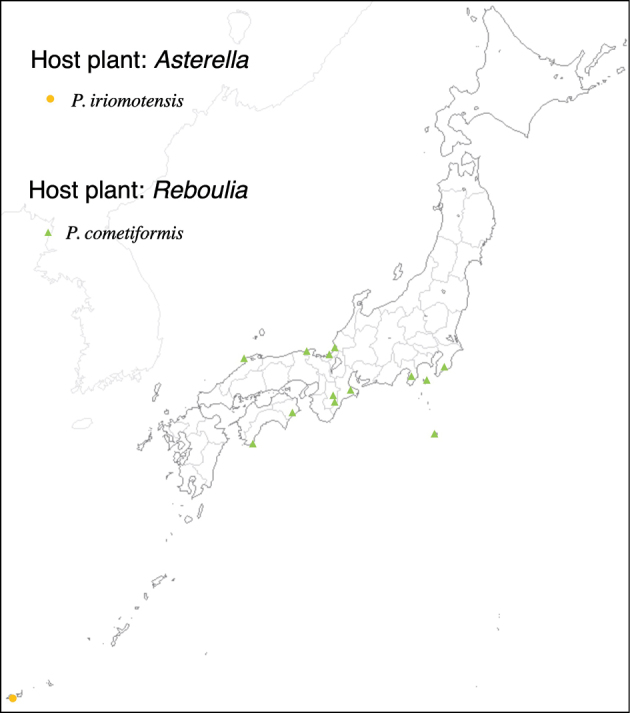
Locality records of three *Phytoliriomyza* species associated with *Asterella*and*Reboulia* spp.: *P.iriomotensis*and*P.cometiformis*.

Japan (Honshu). Recorded only from Iriomote Island in the Ryukyu Archipelago (Fig. 21).

#### Remarks.

 This species resembles *P.ugetsu*,*P.ricciae* and *P.phaeocerotis* in having a wholly dark scutum and yellow maxillary palpus; it is distinguished from *P.ugetsu* by its small size (wing length 1.4 –1.7 mm in *P.iriomotensis*; 2.1–2.7 mm in *P.ugetsu* in wing length), and from *P.ricciae* and *P.phaeocerotis* by the dark brown legs (legs pale brown in the latter two species).

In Japan, six *Asterella* species are distributed in limited areas in central Honshu and Okinawa and Yaeyama Islands, and almost all of them are endangered (Ministry of the Environment Japan 2015). It is remarkable that such rare liverwort species are associated with specific agromyzid species.

##### Species associated with *Reboulia*

### ﻿12.﻿ Phytoliriomyza cometiformis

Taxon classificationAnimaliaDipteraAgromyzidae

Kato sp. nov.

22B3ABC7-3391-5CC7-A306-2A1197061F58

https://zoobank.org/3599CBDF-77C3-4B23-B42B-18F6FD336819

Figs 22 , 23

#### Material examined.

***Holotype*:** Japan: 1♂ (MK-AG-789), Ashizuri-misaki, Tosashimizu, Kochi Pref. (32.7298°N, 132.9971°E, 75 m asl), 26-II-2011 (as larva), emerged on 1-IV-2011 NSMT-I-Dip 31954. ***Paratypes*:** Japan: 1♂1♀ (MK-AG-a427, a428), same data as holotype, emerged on 30-III-2011, NSMT-I-Dip 31955, 31955; 1♀ (MK-AG-a461), Hachijo Is., Tokyo ﻿Pref., 24-IV-2001 (as larva), emerged on 3-V-2001, NSMT-I-Dip 31957; 1♂ (MK-AG-411), Sagiura, Taisha, Izumo, Shimane Pref., 31-III-2015 (as larva), emerged on 30-IV-2015, NSMT-I-Dip 31958.

#### Other material.

Japan: 6♂7♀, Izu-oshima Is. Tokyo Pref., 22-III-2009 (as larva), emerged on 15–20-IV-2009; 2♀, Yugashima, Izu, Shizuoka Pref., 7-III-2012 (as larva), emerged on 12–14-IV-2012; 4♂8♀, Sinjo, Mihama, Mikata, Fukui Pref., 11-III-2012 (as larva), emerged on 18–23-IV-2012; 3♂2♀, Shimaji-gawa, Ujitachi, Ise, Mie Pref., 3-IV-2010 (as larva), emerged on 22-IV–2-V-2010; 2♂1♀, Urashima-jinja, Honjo-hama, Ine, Kyoto Pref., 31-VII-2011 (as larva), emerged on 22-III-2011; 2♂5♀, Ukawa, Tango, Kyotango, Kyoto Pref., 5-III-2021 (as larva), emerged on 2–8-IV-2021; 1♀, Sannoko, Kawakami, Higashi-yoshino, Nara Pref., 26-II-2016 (as larva), emerged on 11-IV-2016; 1♀, Tazukawa-keikoku, Katsuura, Tokushima Pref., 11-X-2016 (as larva), emerged on 19-IV-2011.

#### Diagnosis.

 A large yellow species (wing length 2.0–2.2 mm) having a pruinose dark gray scutum with an oval yellow pattern extending from the mid-posterior margin to the scutellum, a black 1^st^ flagellomere, yellow maxillary palpus, yellow halteres, and yellow legs. Male epandrium inner-laterally with a hand-like comb comprising

Four or five basally fused, long, tubercle-like setae. Larva mines the thallus of *Rebouliahemisphaericaorientalis*.

#### Description.

**Figure 22.**
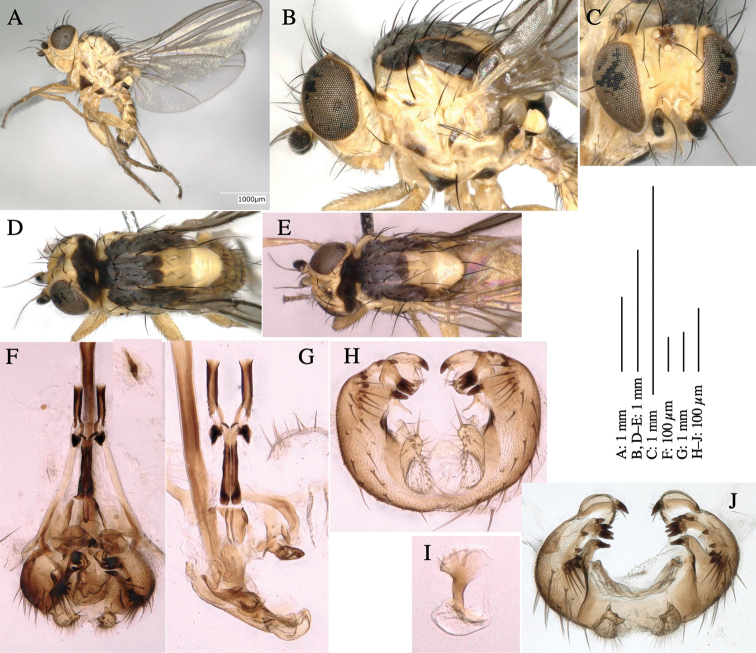
*Phytoliriomyzacometiformis*sp. nov. **A–D **holotype male **A** habitus **B** lateral **C** frontal **D** dorsal** E** paratype female, dorsal (MK-AG-a428) **F–J** male genitalia (**F–H** at type locality **I, J** at Sagiura) **F **whole genitalia, ventral **G** phallic complex, lateral **H, J** epandrium, ventral **I** ejaculatory apodeme, lateral.

**Adult male** (Fig. 22A–D).

***Head*:** Head entirely yellow, with ocellar tubercle brown, and back of head dark brown (Fig. 22C). Antenna porrect; first flagellomere black, pedicel yellow and scape light yellow. Arista subbasal, brown, pubescent. Face, gena, parafacial and postgena yellow. Proboscis normal, yellow; palpus yellow, cylindrical (Fig. 22C). ***Chaetotaxy*:** Front orbitals three pairs; one ori directed inward; two ors directed upward (Fig. 22B). Orbital setulae minute and erect, in a single row.

***Thorax*:** Thorax subshiny. Scutum yellow with a black medial stripe on anterior 2/3, one pair of black suborbicular presutural patches confluent with the medial stripe, and a pair of wide black bands on anterior 7/8, which is confluent with the presutural patches. Scutellum yellow with lateral margins brown (Fig. 22D). Subscutellum yellow. Mediotergite brown, anatergite yellow, katatergite brown (Fig. 22B). Pleuron largely yellow; propleuron with small brown patch on anterior-dorsal corner; anepisternum with a small spot on dorsal corner and a large brown patch near ventral margin; anepimeron with a narrow brown patch on anterior corner; katepisternum and meron with brown patches on venter (Fig. 22B). Haltere yellow. Calypter margin and hairs gray. Leg segments yellow; tibia and tarsus darker. ***Chaetotaxy*:** Scutum with 1+3 dorsocentrals, shortened anteriorly (Fig. 22D). Acrostichal setulae eight or nine pairs in two rows. ***Wing*:** Wing length 2.0–2.2&nbsp;mm, costa reaching M_1_ (Fig. 22A). Length of ultimate section of vein M_4_ divided by penultimate section 1.8–1.9.

***Abdomen*:** Abdomen dorsally subshiny yellow (Fig. 22A). ***Genitalia*:** (Fig. 22F–J) Epandrium dark brown, rounded apically; inner-lateral margin with a row of 5–7 short tubercle-like setae; inner-anterior surface with a comb comprising three or four fused long tubercle-like setae; inner-lateral surface with an enlarged protrusion bearing a strong tubercle-like seta (Fig. 22H, J). Surstylus rounded and bilobed; anterior lobe sparsely setose apically; posterior lobe with one stout tubercle-like seta and a long normal seta. Cercus narrow, setose. Subepandrial sclerite with a pair of plate-like arms, each with a dorsal lobe bearing a seta (Fig. 22H). Hypandrium slightly sclerotized along outer margin. Postgonite bare and goose barnacle-shaped (Fig. 22G). Phallophorus with deep incision below, articulated with phallapodeme, fused to epiphallus (Fig. 22F, G). Basiphallus with long narrow right sclerites and a short basal sclerite. Hypophallus broad and membranous; lateral margins lightly sclerotized, medially with a pair of fused narrow sclerites (Fig. 22G). Mesophallus dark, cylindrical, widest basally, as long as distiphallus (Fig. 22G). Distiphallus comprising one pair of stout tubules; basal 1/3 composed of ventral dark subtriangular sclerite and weaker medial region; distal 2/3 cylindrical, dorsally and laterally pigmented, with truncated, unpigmented apex (Fig. 22G). Ejaculatory apodeme pale brown, fan-shaped with long stalk; sclerite of sperm pump with lateral extension; sperm pump clear (Fig. 22I).

**Figure 23.**
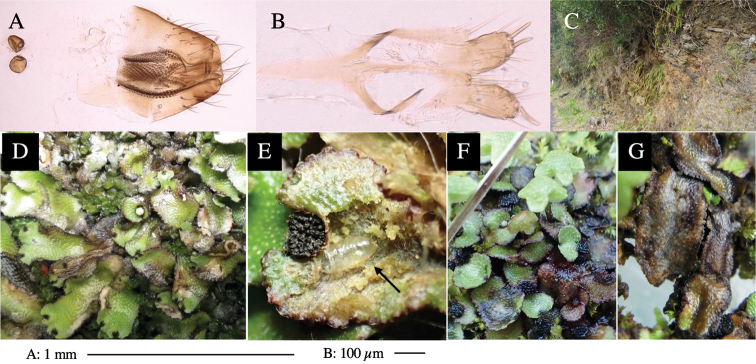
Female morphology and larval ecology of *Phytoliriomyzacometiformis*sp. nov. **A, B **female postabdomen **A** oviscape and spermatheca **B** tergite 10 **C** habitat at type locality **D–G** mined thalli of *Rebouliahemisphaericaorientalis* (**D** at type locality **E** at Ukawa **F, G** at Sagiura). An arrow in **F** indicates an internal puparium.

**Female** (Fig. 22E). Similar to male, but larger. Wing length 2.3 mm. ***Postabdomen*:** (Fig. 23A, B) Oviscape dark brown, setigerous (Fig. 23A). Tergite 10 trifurcate, laterally uniting narrow pleural sclerites (Fig. 23B). Each cercus with two stout, apical, trichoid sensilla, 1/3 length of cercus (Fig. 23B). Spermathecae semi-orbicular, with truncate proximal ends (Fig. 23A).

#### Etymology.

 The specific name (*cometiformis* = comet-shaped) refers to the oblong trail-leaving yellow pattern against the black background on the scutum, which resembles a comet.

#### Japanese name.

Suisei-jingasagoke-hamoguribae.

#### Host plant.

*Rebouliahemisphaericaorientalis* (Aytoniaceae).

#### Mine.

Larvae construct linear-blotch mines in the thallus, and pupate in the mines (Fig. 23D–G).

#### Biological notes.

The habitats of this species are rocky cliffs in warm temperate evergreen forests (Fig. 23C). Our rearing records suggest that this species is univoltine, and that adults emerge from overwintered pupae in spring.

#### Distribution.

Japan (Honshu, Shikoku).

#### Remarks.

 This species resembles *P.igniculus* and *P.luna* in having a pair of black lateral band on scutum and wholly yellow scutellum; it is distinguished from them by the number of tubercle-like setae in a comb of male epandrium (3–4 in *P.cometiformis*; 5–6 in *P.igniculus*; 7–8 in *P.luna*). The morphology of male epandrium of this species closely resembles that of “*P.dorsata*” in Papp and Černý (2017: fig. 101E), suggesting that this species is distributed also in Europe.

### ﻿13.﻿ Phytoliriomyza argentifasciata

Taxon classificationAnimaliaDipteraAgromyzidae

Kato sp. nov.

D5DF54F5-8F4C-5FE9-8616-EA534725D28D

https://zoobank.org/2C7BEF3A-1E11-471A-9002-8DF42A7F0D91

Figs 24 , 25

#### Material examined.

***Holotype*:** Japan: 1♂ (MK-AG-a347), Ukawa, Tango, Kyotango, Kyoto Pref. (35.7102°N, 135.1623°E, 100 m asl), 5-III-2021 (as larva), emerged on 9-IV-2021 NSMT-I-Dip 31959. ***Paratypes*:** Japan: 1♀ (MK-AG-a462), same data as holotype, NSMT-I-Dip 31960; 2♀ (MK-AG-436, 441), Kibune, Sakyo-ku, Kyoto Pref., 24-VI-2011 (as larva), emerged on 12-VII-2011, NSMT-I-Dip 31961, 31961; 1♂ (MK-AG-478), Ryutosen, Higashi-sonogi, Nagasaki Pref., 30-IV-2017 (as larva), emerged on 31-V-2017, NSMT-I-Dip 31963; 1♀ (MK-AG-473), Kibune, Sakyo-ku, Kyoto Pref., 20-VI-2016 (as larva), emerged on 5-VII-2016, NSMT-I-Dip 31964; 1♂1♀ (MK-AG-456, 452), Han-yama, Yaku Is., Kumage, Kagoshima Pref., 29-III-2017 (as larva), emerged on 19-IV-2017, NSMT-I-Dip 31965, 31966; 1♀ (MK-AG-a204), Tachijami, Kume Is. Okinawa Pref., 20-III-2020 (as larva), emerged on 2-V-2020, NSMT-I-Dip 31967.

#### Other material.

Japan: 5♂3♀, Takasuka, Joso, Ibaragi Pref., 2-XI-2021 (as larva), emerged on 11–15-XII-2021; 1♀, Kuchisakamoto, Aoi-ku, Shizuoka Pref., 20-IX-1998 (as larva), emerged on 18–23-IX-1998; 1♀, Mt. Gozaisho, Komono, Mie Pref., 1-V-2001 (as larva), emerged on 25-V-2001; 3♂6♀, Ukawa, Tango, Kyotango, Kyoto Pref., 5-III-2021 (as larva), emerged on 2–8-IV-2021; 16♂10♀, Kibune, Sakyo-ku, Kyoto Pref., 24-VI-2011 (as larva), emerged on 12-VII-2011; 16♂25♀, Wadagawa-kyo, Kumanogawa, Shingu, Wakayama Pref., 7-VI-2021 (as larva), emerged on 17-VII–8-VIII-2021; 1♂1♀, Kibune, Sakyo-ku, Kyoto Pref., 23-IV-2021 (as larva), emerged on 2–6-V-2021; 6♂8♀, Ryugakyo, Yamashiro, Miyoshi, Tokushima Pref., 1-II-2014 (as larva), emerged on 24-IV–3-V-2014; 1♂1♀, Tazukawa-keikoku, Katsuura, Tokushima Pref., 30-III-2021 (as larva), emerged on 26–29-IV-2021; 3♂4♀, Sui, Anan, Tokushima Pref., 30-III-2021 (as larva), emerged on 14–17-IV-2021; 1♂2♀, Kurase-keikoku, Tanbara, Saijo, Ehime Pref., 2-II-2014 (as larva), emerged on 20–26-IV-2014; 1♀, Yasui-keikoku, Niyodogawa, Agawa, Kochi Pref., 27-II-2011 (as larva), emerged on 17-IV-2011; 1♂, Kinsakubaru, Amami, Kagoshima Pref., 4-VII-1999 (as larva), emerged on 25-VII-1999; 5♂11♀, Tachijami, Kume Is. Okinawa Pref., 20-III-2020 (as larva), emerged on 15-IV–8-V-2020.

#### Diagnosis.

 A medium-sized species (wing length 1.5–1.9 mm) having subshiny yellow scutum with a medial and two pairs of lateral dark stripes; inner stripes with silvery reflection. Adults with yellow 1^st^ flagellomere, yellow maxillary palpus, yellow halteres, and yellow legs. Male epandrium inner-laterally with a hand-like comb comprising four or five basally fused, long, tubercle-like setae. Larva mines the thallus of *Rebouliahemisphaericaorientalis*.

#### Description.

**Figure 24.**
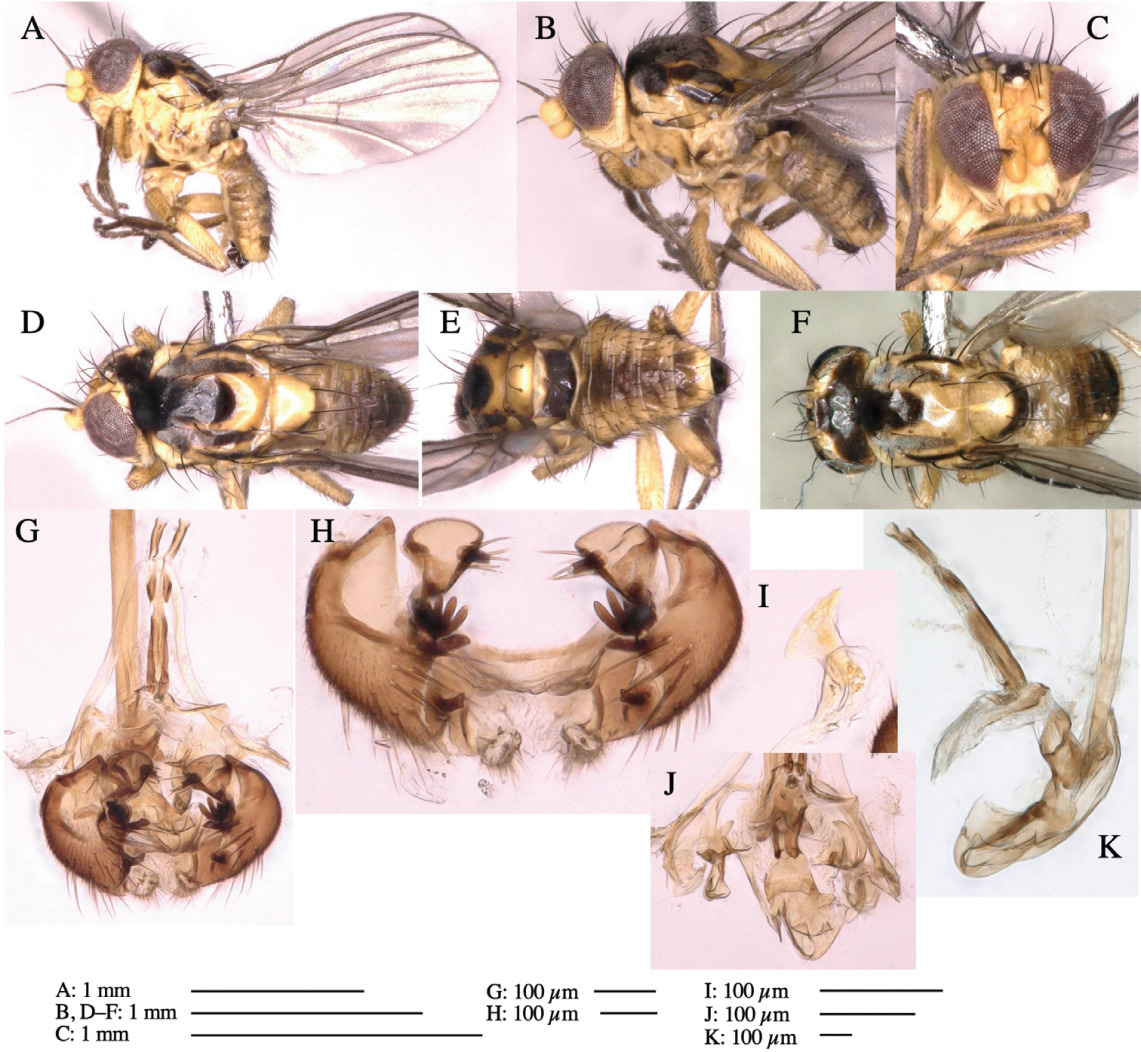
*Phytoliriomyzaargentifasciata*sp. nov. **A–E **holotype male **A** habitus **B** lateral **C** frontal **D** dorsal **E** posterior** F** paratype female (MK-AG-436) **G–J** male genitalia (**G–J** at type locality **K** at Kume Is.) **G** whole genitalia, ventral **H** epandrium, ventral **I** ejaculatory apodeme, lateral, **J** phallic complex, ventral **K** phallic complex, lateral.

**Adult male** (Fig. 24A–E).

***Head*:** Head entirely yellow, with ocellar tubercle brown, and back of head dark brown (Fig. 24C). Antenna porrect, yellow. Arista subbasal, brown, pubescent. Frons with brownish reflective pruinosity. Face, gena, parafacial, and postgena yellow. Proboscis normal, yellow; palpus yellow, cylindrical (Fig. 24C). ***Chaetotaxy*:** Front orbitals three pairs; one ori directed inward; two ors directed upward (Fig. 24B). Orbital setulae minute and erect, in a single row.

***Thorax*:** Thorax subshiny yellow; in some geographic populations, background color grayish yellow (Fig. 24D). Scutum with medial black stripe on anterior 2/3, one pair of gray suborbicular presutural spots confluent with the medial stripe, a pair of narrow black supra-alar stripes and a pair of wider gray intra-alar stripes, which adjoin the pair of lateral presutural gray suborbicular spots; the gray spots and stripes look silver in sunlight. Scutellum and subscutellum yellow (Fig. 24D). Mediotergite brown, anatergite yellow, katatergite brown (Fig. 24E). Pleuron largely yellow (the background color sometimes grayish in some localities); propleuron with small brown patch on mid-anterior corner; anepisternum with two small spots on anterior and posterior corners; anepimeron with a narrow brown patch on anterior corner; katepisternum and meron with brown patches on venter (Fig. 24B). Haltere yellow, while dorsal surface grayish yellow. Calypter margin and hairs gray. Leg segments yellow; tibia and tarsus darker (Fig. 24A). ***Chaetotaxy*:** Scutum with 1+3 dorsocentrals, shortened anteriorly (Fig. 24D). Acrostichal setulae five pairs in two rows. ***Wing***: Wing length 2.0–2.2 mm, costa reaching M_1_ (Fig. 24A). Length of ultimate section of vein M_4_ divided by penultimate section 1.8–1.9.

***Abdomen*:** Abdomen dorsally subshiny yellow (Fig. 24E). ***Genitalia*:** (Fig. 24G–K) Epandrium dark brown, rounded apically; inner-posterior surface with a long apically bifid tubercle-like seta; inner-lateral margin with a long tubercle-like seta, the tip of which is flattened and fan-shaped; inner-anterior surface with a finger-like comb comprising five basally fused tubercle-like setae (Fig. 24H). Surstylus rounded, directed inwards, setose apically, with a single long, apically bifid, tubercle-like seta on posterior margin (Fig. 24H). Cercus narrow, setose. Subepandrial sclerite with a pair of flat, pale, ventral lobes, each bearing a seta basally (Fig. 24J). Hypandrium sclerotized along outer margin (Fig. 24G). Postgonite bare and goose barnacle-shaped (Fig. 24J). Phallophorus with deep incision below, articulated with phallapodeme, fused to epiphallus (Fig. 24K). Basiphallus with a basally bilobed sclerite; each lobe expanded laterally. Hypophallus broad and membranous with lightly sclerotized margins, medially with a pair of fused linear sclerites (Fig. 24K). Mesophallus dark, cylindrical, widest subbasally, 1.2 × longer than distiphallus. Distiphallus comprising one pair of stout tubules basally parallel to each other; basal half composed of lateral dark lanceolate sclerite and weaker medial region; distal half cylindrical, dorsally and laterally pigmented, with truncated, shortly flared unpigmented apex (Fig. 24K). Ejaculatory apodeme pale and fan-shaped with broad stalk; base wide to one side; sperm pump clear (Fig. 24I).

**Figure 25.**
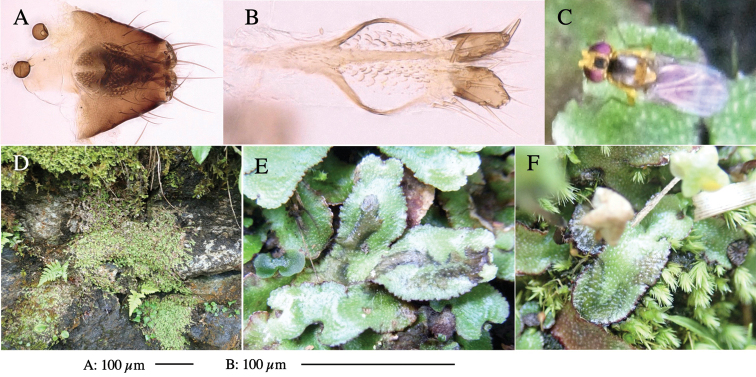
Female morphology and larval ecology of *Phytoliriomyzaargentifasciata*sp. nov. **A, B **female postabdomen **A** oviscape and spermatheca **B** tergite 10 **C** live fly at Kibune **D** habitat at Mt Oe **E, F** mined thalli of *Rebouliahemisphaericaorientalis*(**E** at Kibune **I** Kume Is.).

**Female** (Fig. 24F, 25C). Similar to male, but larger, frons wider. Wing length 2.3&nbsp;mm. ***Postabdomen*:** (Fig. 25A, B) Oviscape dark brown, setigerous (Fig. 25A). Tergite 10 trifurcate, laterally uniting narrow pleural sclerites (Fig. 25B). Each cercus with two stout, apical, trichoid sensilla, 1/3 length of cercus (Fig. 25B). Spermathecae semi-orbicular, with truncate proximal ends (Fig. 25A).

#### Variation.

Background color of scutum and scutellum varies from yellow to grayish yellow, and the blackening is obvious in the Yaku Islands.

#### Etymology.

 The specific name (*argentus* = silver, *fascia* = stripe) refers to silver stripes on the scutum, which are obvious in sunlight.

#### Japanese name.

Ginsuji-jingasagoke-hamoguribae.

#### Host plant.

*Rebouliahemisphaericaorientalis* (Aytoniaceae).

#### Mine.

Larvae construct digitate mines in the thallus, and pupate in the mine (Fig. 25E–I).

#### Biological notes.

The habitats of this species are rocky cliffs in warm temperate evergreen forests (Fig. 25D), and on the ground or on stone walls of temples, shrines and farms in rural ecosystems. Our rearing records suggest that this species is bivoltine, with adults emerging in spring and summer.

#### Distribution.

**Figure 26.**
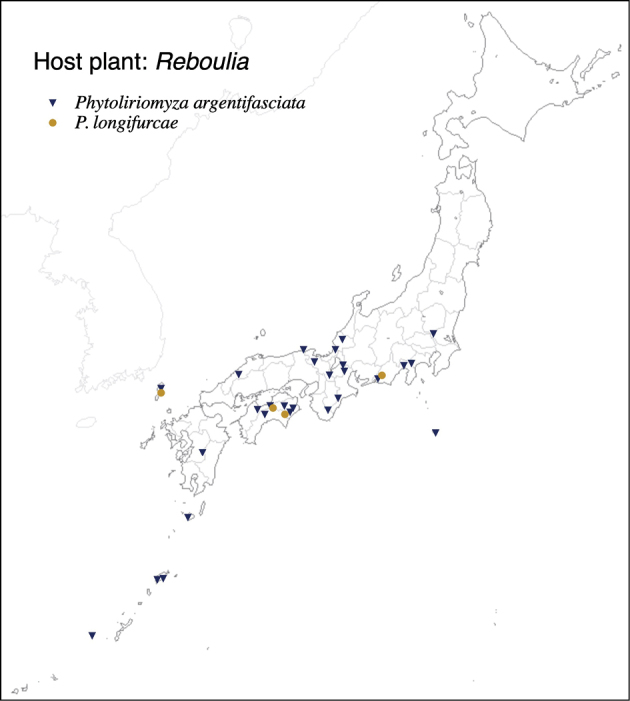
Locality records of three *Phytoliriomyza* species associated with *Reboulia* spp.: *P.argentifasciata* and *P.longifurcae*.

Japan: Honshu, Shikoku, Yaku Island, Amami-Oshima Island, Kume Island (Fig. 26)

#### Remarks.

 This species resembles *P.dorsata*,*P.calcicola*, *P.longifurcae*,*P.nigroflava*, and *P.brunofasciata* in having two pair of dark lateral bands on the scutum; it is distinguished from them by the silverly reflecting inner stripes (inner stripes black or gray in the other species), and by the extended, distorted tubercle-like seta on the subdistal margin of the male epandrium.

### ﻿14.﻿ Phytoliriomyza longifurcae

Taxon classificationAnimaliaDipteraAgromyzidae

Kato sp. nov.

37EBF15E-01AC-5AB8-A65F-13945BB01B43

https://zoobank.org/14E86C9E-CAD6-494B-8E6A-21801A53D403

Figs 27 , 28

#### Material examined.

***Holotype*:** Japan: 1♂ (MK-AG-a500), Sui, Anan, Tokushima Pref. (33.9044°N, 134.5391°E, 40 m asl), 30-III-2021 (as larva), emerged on 14-V-2021, NSMT-I-Dip 31968. ***Paratypes*:** Japan: 3♀ (MK-AG-a497–499), same data as holotype, emerged on 14–17-V-2021, NSMT-I-Dip 31969–31971.

#### Other material.

Japan: 4♂3♀, Kamihirayama, Tatsuyama, Tenryu, Hamamatsu, Shizuoka Pref., 7-XI-2010 (as larva), emerged on 18–28-IV-2011; 1♂1♀, Chiromo, Toyotama, Tsushima, Nagasaki Pref., 28-XI-2011 (as larva), emerged on 1-V-2011; 2♀, Kibune, Sakyo-ku, Kyoto Pref., 20-VI-2016 (as larva), emerged on 14-VII-2021.

#### Diagnosis.

 A medium-sized species (wing length 1.5–1.6 mm) having a subshiny yellow scutum with a medial and two pairs of lateral black stripes, yellow 1^st^ flagellomere, yellow maxillary palpus, yellow halteres, and yellow legs. Male epandrium inner-basally with a comb comprising six fused long tubercle-like setae, and inner-subdistally with an extremely elongated arm, which apically bears two dark, diverging, ventrally curved, tubercle-like setae. Larva mines the thallus of *Rebouliahemisphaericaorientalis*.

#### Description.

**Figure 27.**
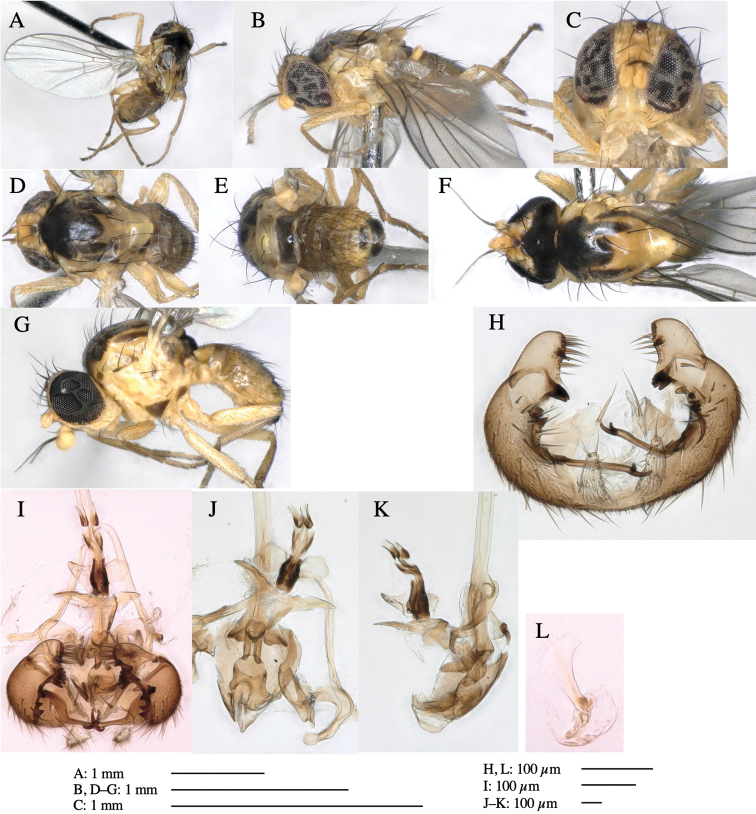
*Phytoliriomyzalongifurcae*sp. nov. **A–E **holotype male **A** habitus **B** lateral **C** frontal **D** dorsal **E** posterior** F, G** paratype female (MK-AG-a497) **F** dorsal **G** lateral **H–L** male genitalia **H** epandrium, ventral **I** whole genitalia, ventral **J, K** phallic complex, ventral and lateral **L** ejaculatory apodeme, lateral.

**Adult male** (Fig. 27A–E).

***Head*:** Head entirely yellow, with ocellar tubercle brown, and back of head dark brown (Fig. 27C). Antenna porrect, yellow. Arista subbasal, brown, pubescent. Frons with brownish reflective pruinosity. Face, gena, parafacial and postgena yellow. Proboscis normal, yellow; palpus yellow, cylindrical (Fig. 27C). ***Chaetotaxy*:** Front orbitals three pairs; one ori directed inward; two ors directed upward (Fig. 27B). Orbital setulae minute and erect, in a single row.

***Thorax*:** Thorax subshiny yellow. Scutum with medial black stripe on anterior 2/3, one pair of black suborbicular presutural spots confluent with the medial stripe, a pair of narrow black supra-alar stripes and a pair of wider black intra-alar stripes, which adjoin the pair of lateral presutural black suborbicular spots (Fig. 27D). Mediotergite brown, anatergite yellow, katatergite brown (Fig. 27E). Pleuron largely yellow (the background color sometimes grayish in some localities); propleuron with small brown patch on mid-anterior corner; anepisternum with two small spots on anterior and posterior corners; anepimeron with a narrow brown patch on anterior corner; katepisternum and meron with brown patches on venter (Fig. 27B). Haltere yellow, while dorsal surface grayish yellow. Calypter margin and hairs gray. Leg segments yellow; tibia and tarsus darker. ***Chaetotaxy*:** Scutum with 1+3 dorsocentrals, shortened anteriorly (Fig. 27D). Acrostichal setulae five pairs in two irregular rows. ***Wing*:** Wing length 1.5 mm, costa reaching M_1_ (Fig. 27A). Length of ultimate section of vein M_4_ divided by penultimate section 1.6.

***Abdomen*:** Abdomen dorsally subshiny yellow (Fig. 27E). ***Genitalia*:** (Fig. 27H–L) Epandrium dark brown, rounded apically; inner-lateral margin with a row of four short tubercle-like setae; inner-subdistal margin with an extremely elongated arm, bearing two, dark, ventrally curved, tubercle-like setae borne at wide angle (90–120°); inner-basal surface with a comb comprising 4–6 fused tubercle-like setae (Fig. 27K). Surstylus lobate, directed inwards, setose apically, with one short tubercle-like seta subapically (Fig. 27K). Cercus narrow, setose. Subepandrial sclerite with a pair of flat, pale, ventral lobes, each of which bearing a long seta subapically (Fig. 27K). Hypandrium thin, slightly sclerotized along outer margin (Fig. 27H). Postgonite bare, goose barnacle-shaped, cleft apically; upper lobe pointed apically (Fig. 27K). Phallophorus with deep incision below (Fig. 27I), articulated with phallapodeme, fused to epiphallus (Fig. 27J). Basiphallus dorsally sclerotized with basal expanded lobes (Fig. 27I, J). Hypophallus broad, lightly sclerotized, lateral lobes expanded anteriorly like wings, clear tubule emerging from median part (Fig. 27I). Mesophallus dark, cylindrical, constricted subapically. Paraphalli membranous, rounded and expanded ventrally, bilaterally asymmetrical; right one larger than left one (Fig. 27I). Distiphallus comprising one pair of stout tubules basally parallel to each other; basal half composed of lateral dark lanceolate sclerite and weaker medial region; distal half cylindrical, dorsally and laterally pigmented, with truncated, flared clear apex (Fig. 27J). Ejaculatory apodeme pale and fan-shaped with broad stalk; base wide to one side; sperm pump clear (Fig. 27L).

**Figure 28.**
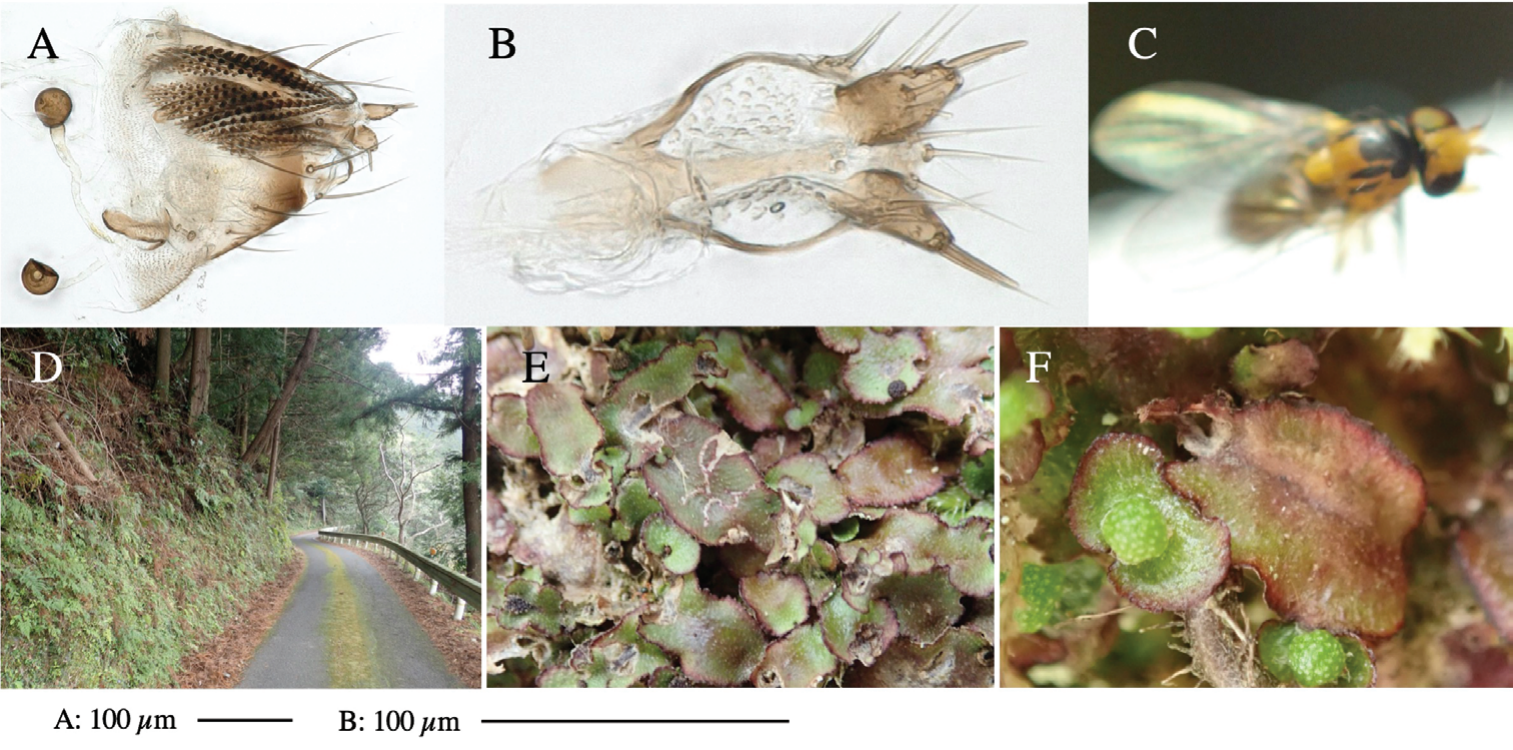
Female morphology and larval/adult ecology of *Phytoliriomyzalongifurcae*sp. nov. **A, B **female postabdomen **A** oviscape and spermatheca **B** tergite 10 **C** live fly **D** habitat at Sui **E** mined thalli of *Rebouliahemisphaericaorientalis*.

**Female** (Fig. 27F, G). Similar to male, but larger, frons wider. Wing length 1.6&nbsp;mm. ***Postabdomen*:** (Fig. 28A, B) Oviscape dark brown, setigerous (Fig. 28A). Tergite 10 trifurcate, laterally uniting narrow pleural sclerites (Fig. 28B). Each cercus with two stout, apical, trichoid sensilla, ¾ length of cercus (Fig. 28B). Spermathecae semi-orbicular, with truncate proximal ends (Fig. 28A).

#### Etymology.

 The specific name (*longus* = long, *furca* = fork) refers to extremely elongated, apically biforked tubercle-like seta on the male epandrium.

#### Japanese name.

Sasumata-jingasagoke-hamoguribae.

#### Host plant.

*Rebouliahemisphaericaorientalis* (Aytoniaceae).

#### Mine.

Larvae construct linear-blotch mines in the thallus, and pupate in the mines (Fig. 28E, F).

#### Biological notes.

The habitats of this species are rocky cliffs in warm temperate evergreen forests (Fig. 28D). This species is rarer than *P.argentifasciata*, and sympatric with the latter in some localities. Our rearing records suggest that adults emerge from overwintered pupae in spring.

#### Distribution.

Japan: Honshu, Shikoku (Fig. 26).

#### Remarks.

 This species resembles *P.argentifasciata* and *P.nigroflava* in having two pair of dark lateral bands on the scutum, and a yellow 1^st^ flagellomere and yellow haltere; it is distinguished from *P.argentifasciata* by the black lateral stripes (inner bands reflecting silverly in sunlight in *P.argentifasciata*), from *P.nigroflava* by the absence of an extremely extended, forked tubercle-like seta on the subdistal margin of the male epandrium.

### ﻿15.﻿ Phytoliriomyza falcata

Taxon classificationAnimaliaDipteraAgromyzidae

Kato sp. nov.

D32F57BD-A41F-5A6D-9691-5338F8693DCC

https://zoobank.org/C62852AE-213D-4143-AB0A-E8472CFFD2AC

Figs 29 , 30

#### Material examined.

***Holotype*:** Japan: 1♂ (MK-AG-a19), Kanna-gawa, Nakatsugawa, Chichibu, Saitama Pref. (36.0044°N,138.8108°E, 760 m asl), 14-XI-2010 (as larva), emerged on 23-IV-2011, NSMT-I-Dip 31972. ***Paratypes*:** Japan: 1♂2♀ (MK-AG-423, 807, a18), same data as holotype, emerged on 28–23-IV-2011, NSMT-I-Dip 31973–31975; 1♀ (MK-AG-a376), Ukawa, Tango, Kyotango, Kyoto Pref., 5-III-2021 (as larva), emerged on 12-IV-2021, NSMT-I-Dip 31976; 1♂ (MK-AG-a287), Seya-gawa, Miyazu, Kyoto Pref., 18-IV-2013 (as larva), emerged on 25-IV-2013, NSMT-I-Dip 31977; 1♂ (MK-AG-803), Ryugakyo, Yamashiro, Miyoshi, Tokushima Pref., 1-II-2014 (as larva), emerged on 25-IV-2014, NSMT-I-Dip 31978; 1♀ (MK-AG-444), Yasui-keikoku, Niyodogawa, Agawa, Kochi Pref., 27-II-2011 (as larva), emerged on 28-IV-2011, NSMT-I-Dip 31979; 1♀ (MK-AG-795), Chiromo, Toyotama, Tsushima, Nagasaki Pref., 11-XI-2011 (as larva), emerged on 28-IV-2012, NSMT-I-Dip 31980.

#### Other material.

Japan: 17♂22♀, Kanna-gawa, Nakatsugawa, Chichibu, Saitama Pref., 14-XI-2010 (as larva), emerged on 14-IV-2011; 6♂11♀, Oochi-gawa, Chichibu, Saitama Pref., 13-III-2017 (as larva), emerged on 25–28-IV-2011; 1♂2♀, Ukawa, Tango, Kyotango, Kyoto Pref., 5-III-2021 (as larva), emerged on 9–17-IV-2021; 1♀, Doro-kyo, Totsugawa-mura, Nara Pref., 29-III-2019 (as larva), emerged on 3-IV-2019; 1♂4♀, Tazukawa-keikoku, Katsuura, Tokushima Pref., 30-III-2021 (as larva), emerged on 23–30-IV-2021; 2♂1♀, Kurase-keikoku, Tanbara, Saijo, Ehime Pref., 2-II-2014 (as larva), emerged on 20–26-IV-2014.

#### Diagnosis.

 A medium-sized yellow species (wing length 1.6–2.0 mm) having subshiny brown scutum with an oval yellow pattern extending from the mid-posterior margin to the scutellum, a yellow 1^st^ flagellomere, yellow maxillary palpus, yellow halteres, and yellow legs. Male epandrium inner-laterally with a long hypertrophied arm which apically bears a dark, long, apically flattened, obliquely truncated tubercle-like seta. Larva mines the thallus of *Rebouliahemisphaericaorientalis*.

#### Description.

**Figure 29.**
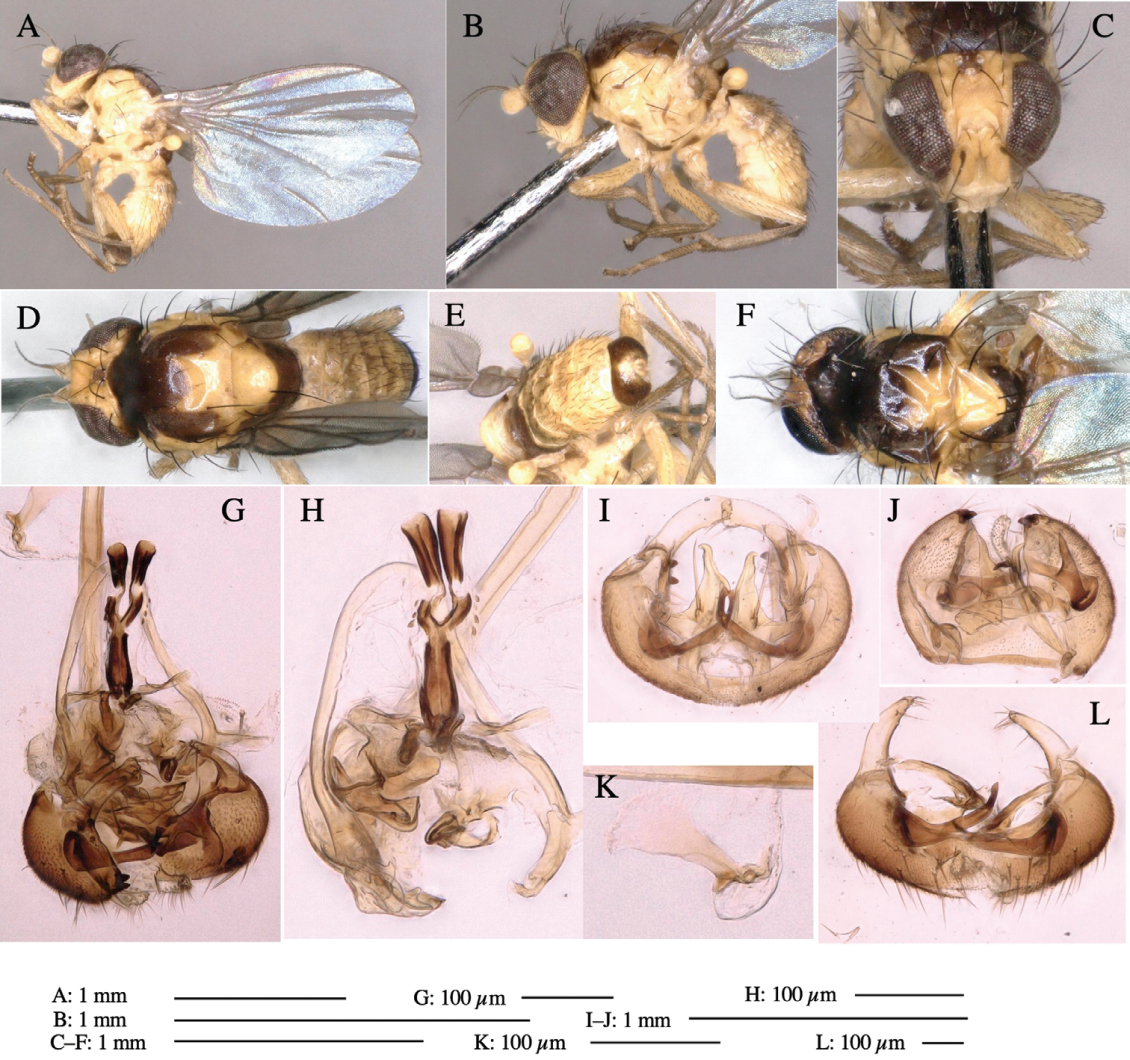
*Phytoliriomyzafalcata*sp. nov. **A–E **holotype male **A** habitus **B** lateral **C** frontal **D** dorsal **E** posterior **F** paratype female (MK-AG-a18) dorsal **G–L** male genitalia (**G–K**, at type locality **L** at Tatsuyama) **G** whole genitalia, ventral **H** phallic complex, ventral **I, J, L** epandrium (**I** anterior **J** anterior-ventral **L** ventral) **K** ejaculatory apodeme, lateral.

**Adult male** (Fig. 29A–E).

***Head*:** Head entirely yellow, with ocellar tubercle brown, and back of head dark brown (Fig. 29C). Antenna porrect, first flagellomere black, pedicel yellow and scape light yellow. Arista subbasal, brown, pubescent. Frons with brownish pruinosity. Face, gena, parafacial and postgena yellow. Proboscis normal, yellow; palpus yellow, cylindrical (Fig. 29C). ***Chaetotaxy*:** Front orbitals three pairs; one ori directed inward; two ors directed upward (Fig. 29B). Orbital setulae minute and erect, in a single row.

***Thorax*:** Thorax subshiny, with a brown medial stripe on anterior 2/3, and a pair of adjacent wide brown bands on anterior 7/8 (Fig. 29D). Scutellum and subscutellum yellow. Mediotergite brown, anatergite and katatergite yellow. Pleuron largely yellow; propleuron with small brown patch on mid-anterior corner; anepisternum with two small spots on anterior and posterior corners; anepimeron with an oblique narrow brown patch on anterior corner; katepisternum and meron with brown patches on venter (Fig. 29B). Haltere yellow. Calypter margin and hairs gray. Leg segments yellow; tibia and tarsus darker (Fig. 29B). ***Chaetotaxy*:** Scutum with 1+3 dorsocentrals, shortened anteriorly (Fig. 29D). Acrostichal setulae 6–8 pairs in two rows. ***Wing***: Wing length 1.6–1.9 mm, costa reaching M_1_ (Fig. 29A). Length of ultimate section of vein M_4_ divided by penultimate section 1.5–1.8.

***Abdomen*:** Abdomen dorsally subshiny yellow (Fig. 29E). ***Genitalia*:** (Fig. 29G–L) Epandrium dark brown, rounded apically; inner-anterior margin with two short tubercle-like setae; inner-lateral surface with a basally enlarged, extremely extended, ventrally curved arm, bearing an apically flattened, obliquely truncated, tubercle-like seta borne (Fig. 29I, J, L). Surstylus long, narrow, setose apically. Cercus narrow, setose. Subepandrial sclerite consisting of a pair of dorsal and ventral arms; ventral arm narrow, extended, plate-like, with hooked apex and a long basal seta; dorsal arm also plate-like but shorter, with a seta basally (Fig. 29I). Hypandrium thin, slightly sclerotized along outer margin (Fig. 29G). Postgonite bare and goose barnacle-shaped, with sideward pointed apex (Fig. 29H). Phallophorus with deep incision below, articulated with phallapodeme, fused to epiphallus (Fig. 29H). Basiphallus with a pair of expanded lightly sclerotized lateral plates. Hypophallus broad, membranous, and bilaterally asymmetrical; right margin sclerotized; medially with a pair of dark fused sclerites (Fig. 29H). Paraphalli lobate, lightly sclerotized; diverging and angled anteroventrally (Fig. 29H). Mesophallus dark, cylindrical, widest subbasally, as long as distiphallus (Fig. 29H). Distiphallus comprising one pair of stout tubules; basal half composed of lateral dark slender sclerite and weaker medial region; covered by membrane bearing four pairs of minute arrowhead-like lateral sclerites; distal half cylindrical, dorsally and laterally pigmented, with truncated, shortly flared apex (Fig. 29H). Ejaculatory apodeme pale and fan-shaped with broad stalk; base wide to one side; sperm pump clear (Fig. 29K).

**Figure 30.**
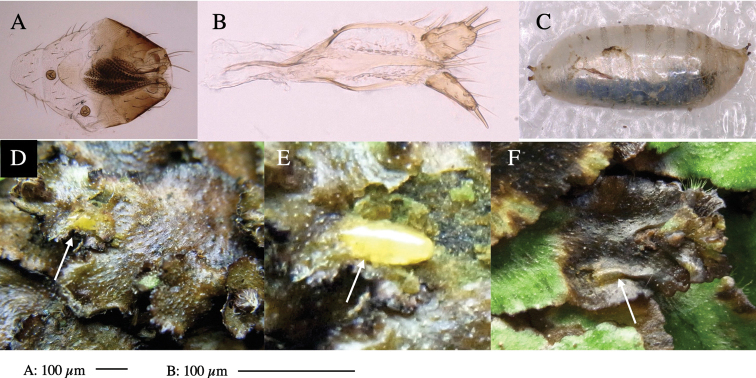
Female morphology and larval ecology of *Phytoliriomyzafalcata*sp. nov. **A, B **female postabdomen **A** oviscape and spermatheca **B** tergite 10 **C** puparium **D–F** mined thalli of *Rebouliahemisphaericaorientalis*(**D, E** at type locality **F **Yasui-keikoku), arrows indicating puparia.

**Female** (Fig. 29F). Similar to male, but larger, the lateral bands on scutum more grayish and more pruinose. Wing length 1.8–2.0 mm. ***Postabdomen*:** (Fig. 30A, B) Oviscape dark brown, setigerous (Fig. 29A). Tergite 10 trifurcate, laterally uniting narrow pleural sclerites (Fig. 29B). Each cercus with two stout, apical, trichoid sensilla, ½ length of cercus (Fig. 29B). Spermathecae semi-orbicular, with truncate proximal ends (Fig. 29A).

**Immatures.** (Fig. 30D–F) Puparium internal, slender, and pale brown, with anterior spiracles just protruded from epidermis of mined thallus.

#### Etymology.

 The specific name (*falcata* = sickle-shaped) refers to the sickle-shaped tubercle-like seta on the male epandrium.

#### Japanese name.

Naginata-jingasagoke-hamoguribae.

#### Host plant.

*Rebouliahemisphaericaorientalis* (Aytoniaceae).

#### Mine.

Larvae construct linear-blotch mines in the thallus, and pupate in the mines (Fig. 30E–I).

#### Biological notes.

 The habitats of this species are rocky cliffs in warm temperate evergreen forests. This species is sympatric with *P.argentifasciata* in some localities. Our rearing records suggest that it is bivoltine, and that adults emerge from overwintered pupae in spring.

#### Distribution.

**Figure 31.**
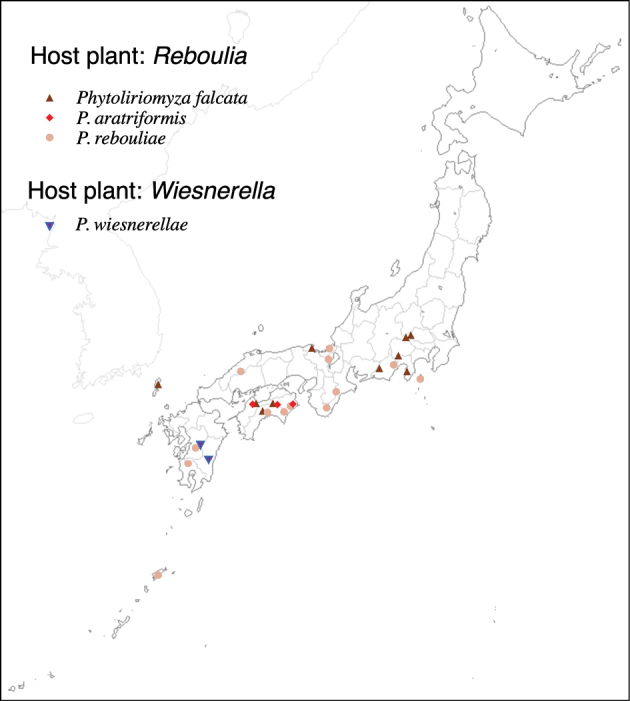
Locality records of three *Phytoliriomyza* species associated with *Reboulia* and *Wiesnerella* spp.: *P.falcata*, *P.aratriformis*, *P.rebouliae* and *P.wiesnerellae*.

Japan: Honshu, Shikoku, Tsushima Island (Fig. 31).

#### Remarks.

 This species resembles *P.arcus*,*P.plagiochasmatos* and *P.aratriformis* in having a pair of brown lateral bands and pale yellow mark on the scutum; it is distinguished from them by the absence of an extremely extended, forked tubercle-like seta on the subdistal margin of the male epandrium. This species is sympatric with *P.aratriformis* on *Reboulia*, and can be distinguished from the latter by the yellow mark on the scutum; the mark is large and well defined by lateral stripes in *P.falcata* but small and obscure in *P.aratriformis*.

### ﻿16.﻿ Phytoliriomyza aratriformis

Taxon classificationAnimaliaDipteraAgromyzidae

Kato sp. nov.

2D18C313-2414-512B-A143-FA846ACA70A6

https://zoobank.org/1693ABD7-C052-499D-93FA-301179B2D102

Fig. 32

#### Material examined.

***Holotype*:** Japan: 1♂ (MK-AG-a311), Tazukawa-keikoku, Katsuura, Tokushima Pref. (33.8952°N, 134.4608°E, 270 m asl), 30-III-2021 (as larva), emerged on 23-IV-2021, NSMT-I-Dip 31981. ***Paratypes*:** Japan: 1♂ (MK-AG-a463), type locality, 11-X-2016 (as larva), emerged on ?-IV-2017, NSMT-I-Dip 31982; 1♀ (MK-AG-427), Nakatsugawa-keikoku, Chichibu, Kyoto Pref., 14-XI-2010 (as larva), emerged on 4-V-2011, NSMT-I-Dip 31983; 1♀ (MK-AG-a17), Kanna-gawa, Nakatsugawa, Chichibu, Saitama Pref., 14-XI-2010 (as larva), emerged on 14-IV-2021, NSMT-I-Dip 31984; 1♂ (MK-AG-a346), Naiku, Oe, Fukuchiyama, Kyoto Pref., 19-V-2010 (as larva), emerged on 20-VI-2021, NSMT-I-Dip 31985.

#### Other material.

Japan: 1♂, Ryugakyo, Yamashiro, Miyoshi, Tokushima Pref., 21-IV-2014 (as larva), emerged on 2-V-2014.

#### Diagnosis.

 A medium-sized yellow species (wing length 1.9–2.3 mm) having a subshiny brown scutum with an obscure oval yellow pattern extending from the mid-posterior margin to the scutellum, a yellow 1^st^ flagellomere, yellow maxillary palpus, yellow halteres, and yellow legs. Male epandrium inner-laterally with a long hypertrophied, ventrally curved arm that apically bears a dark, apically bifid tubercle-like seta. Larva mines the thallus of *Rebouliahemisphaericaorientalis*.

#### Description.

**Figure 32.**
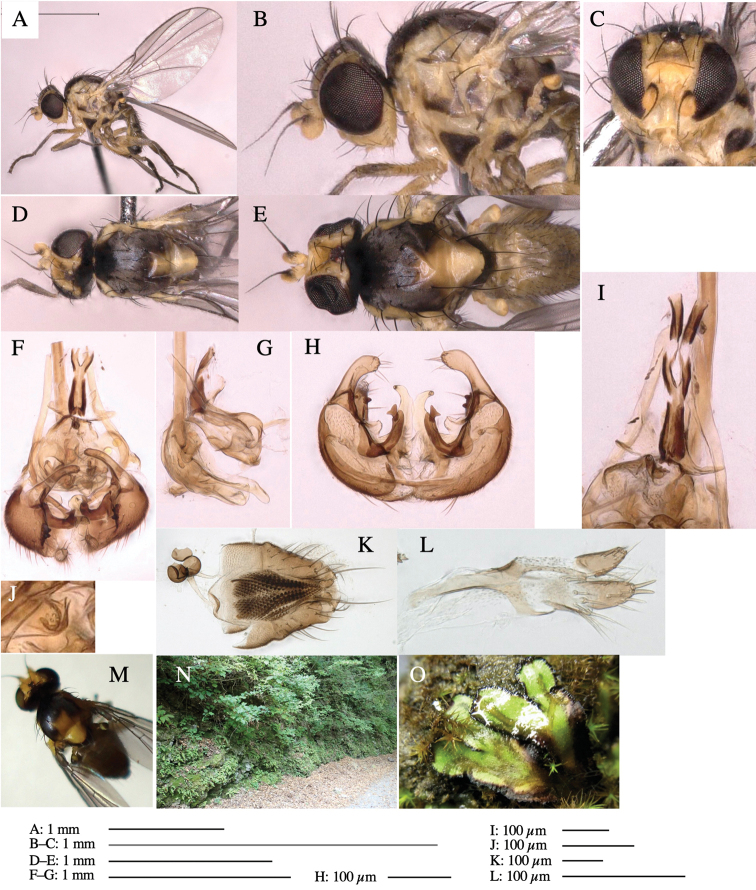
*Phytoliriomyzaaratriformis*sp. nov. **A–D **holotype male **A** habitus **B** lateral **C** frontal **D** dorsal **E** paratype female (MK-AG-a346) **F–I** male genitalia (**K–H** type locality **I, J** Okuchihibu) **F, I** phallic complex, ventral (distiphallus lost) **G** phallic complex, lateral **H** epandrium, ventral **J** postgonite **K, L** female postabdomen **K** oviscape and spermatheca (one spermatheca split into two) **L** tergite 10** M **live female **N** habitat at Nakatsugawa** O** mined thallus of *Rebouliahemisphaericaorientalis* at Nakatsugawa.

**Adult male** (Fig. 32A–D).

***Head*:** Head light yellow, with ocellar tubercle dark brown, frons yellowish brown, back of head dark brown excluding margins (Fig. 32C). Antenna porrect, first flagellomere black, pedicel and scape brown (Fig. 32B). Arista subbasal, black, pubescent. Clypeus, face, gena, parafacial and postgena yellow. Proboscis normal, yellow; palpus brown, cylindrical (Fig. 32C). ***Chaetotaxy*:** Front orbitals three pairs; one ori directed inward; two ors directed upward (Fig. 32B). Orbital setulae minute and erect, in a single row.

***Thorax*:** Thorax pruinose. Scutum pruinose gray, with a small yellow patch along midposterior margin (Fig. 32D). Scutellum light yellow with lateral corner brown, subscutellum light yellow. Mediotergite and anatergite brown, katatergite light yellow. Pleuron yellow with brownish patches on venter of propleuron, anepisternum, katepisternum, anepimeron, and meron (Fig. 32B). Haltere yellow but light yellow basally. Calypter margin and hairs gray. Leg segments brownish, basal half of femur paler (Fig. 32A). ***Chaetotaxy*:** Scutum with 1+3 dorsocentrals, shortened anteriorly (Fig. 32D). Acrostichal setulae seven or eight pairs in two rows. ***Wing***: Wing length 2.2 mm, costa reaching M_1_ (Fig. 32A). Length of ultimate section of vein M_4_ divided by penultimate section 1.3.

***Abdomen*:** Abdomen dorsally subshiny brown; epandrium dark brown (Fig. 32A). ***Genitalia*:** (Fig. 32K–O) Epandrium rounded apically; inner-anterior margin with two short tubercle-like setae; inner-lateral surface with a basally enlarged, extremely extended/thickened, ventrally curved arm, bearing a dark bifid tubercle-like seta borne (Fig. 32H). Surstylus narrow, extended, curved inwards, setose apically (Fig. 32H). Cercus narrow, setose. Subepandrial sclerite consisting of one pair of flat plate-like, basally fused, dorsal sclerites, and one pair of pale plate-like, separated, ventral lobes (Fig. 32H). Hypandrium slightly sclerotized along outer margin (Fig. 32F). Postgonite bare, goose barnacle-shaped, and cleft apically; dorsal lobe pointed apically (Fig. 32J). Phallophorus with deep incision below, articulated with phallapodeme, fused to epiphallus (Fig. 32H). Basiphallus with a pair of broad lateral lobes (Fig. 32F). Hypophallus broad, membranous, and bilaterally asymmetrical; right lateral margin well sclerotized, left lateral margin basally sclerotized; medially with a pair of fused linear sclerites (Fig. 32F, I). Paraphallus 4-winged, with posterior margin lightly sclerotized; paraphalli diverging, angled anteroventrally, jointed basally (Fig. 32H, I). Mesophallus dark, cylindrical, widest subbasally, as long as distiphallus (Fig. 32H). Distiphallus comprising one pair of stout tubules; basal half with pigmented and weaker medial regions; distal half cylindrical, dorsally pigmented, widening toward truncated shortly flared unpigmented apex (Fig. 32I).

**Female** (Fig. 32E, M). Similar to male, but slightly larger, and dorsal abdomen paler (Fig. 32E). Wing length 2.3 mm. ***Postabdomen*:** (Fig. 32K, L) Oviscape dark brown, setigerous (Fig. 32K). Tergite 10 trifurcate, laterally uniting narrow pleural sclerites (Fig. 32L). Each cercus with two stout, apical, trichoid sensilla, 1/3 length of cercus (Fig. 32L). Spermathecae orbicular (Fig. 32K).

#### Etymology.

 The specific name (*aratriformis* = plow-shaped) refers to the plow-shaped tubercle-like seta on the male epandrium.

#### Japanese name.

Karasuki-jingasagoke-hamoguribae.

#### Host plant.

*Rebouliahemisphaericaorientalis* (Aytoniaceae).

#### Mine.

Larva constructs linear mine in the thallus, and pupate in the mine (Fig. 32O).

#### Biological notes.

 The habitats of this species are rocky cliffs in warm temperate evergreen forests (Fig. 32N). This species is rare, sympatric with *P.argentifasciata* and *P.falcata* in some localities. Our rearing records suggest that it is univoltine, and that adults emerge from overwintered pupae in spring.

#### Distribution.

Japan: Honshu, Shikoku, Tsushima Island (Fig. 31).

#### Remarks.

 This species resembles *P.arcus*,*P.plagiochasmatos* and *P.falcata* in having a pair of brown lateral bands and a pale yellow mark on the scutum, but is distinguished from all of these species by the small, ill-defined yellow mark on the scutum (the mark larger and well-defined in the other species), and by the presence of a stout, curved, plow-shaped tubercle-like seta on the subdistal margin of the male epandrium.

### ﻿17.﻿ Phytoliriomyza rebouliae

Taxon classificationAnimaliaDipteraAgromyzidae

Kato sp. nov.

766D267E-5952-554F-97C3-B7CE1DFCF28C

https://zoobank.org/F4792E8-9C4F-4423-87D2-F6BD92E9DB52

Figs 33 , 34

#### Material examined.

***Holotype*:** Japan: 1♂ (MK-AG-a423), Wadagawa-kyo, Kumanogawa, Shingu, Wakayama Pref. (33.7609°N, 135.8260°E, 90 m asl), 7-VII-2021 (as larva), emerged on 2-VIII-2021, NSMT-I-Dip 31986. ***Paratypes*:** Japan: 2♂1♀ (MK-AG-a424, a490, a491), same data as holotype, emerged on 24–30-VII-2021, NSMT-I-Dip 31987–31989; 1♂ (MK-AG-467), Uri-toge, Mikkabi, Hamamatsu, Shizuoka Pref., 7-III-2017 (as larva), emerged on 15-VI-2017, NSMT-I-Dip 31990; 1♂ (MK-AG-a422), Sannoko, Kawakami, Higashi-yoshino, Nara Pref., 26-II-2016 (as larva), emerged on 23-IV-2016, NSMT-I-Dip 31991.

#### Other material.

Japan: 1♀, Izu-oshima Is. Tokyo Pref., 22-III-2009 (as larva), emerged on 19-IV-2009; 2♀, Ashiu, Nantan, Kyoto Pref., 12-III-2018 (as larva), emerged on ?-IV-2018; 2♂6♀, Sannoko, Kawakami, Higashi-yoshino, Nara Pref., 26-II-2016 (as larva), emerged on 18–27-IV-2016; 19♂13♀, WD, 7-VII-2021 (as larva), emerged on 24-VII–6-VIII-2021; 2♂, Yajiemon-jinja, Sakurae, Gotsu, Shimane Pref., 24-VI-2012 (as larva), emerged on 19-VII–23-VIII-2012; 2♂2♀, Tazukawa-keikoku, Katsuura, Tokushima Pref., 11-X-2016 (as larva), emerged on 1-V-2016; 1♂3♀, Yasui-keikoku, Niyodogawa, Agawa, Kochi Pref., 27-II-2011 (as larva), emerged on 18-IV-2011; 1♂3♀, YSI, 27-II-2011 (as larva), emerged on 18-IV-2011; 1♀, Shiibaru, Izumi, Yatsushiro, Kumamoto Pref., 23-III-2015 (as larva), emerged on 28-IV-2015; 1♀, Okujisso, Isa, Kagoshima Pref., 17-XII-2012 (as larva), emerged on 12-IV-2013; 2♂1♀, Sumiyo, Amami, Kagoshima Pref., 17-II-1999 (as larva), emerged on 25–28-II-1999.

#### Diagnosis.

 A small dark species (wing length 1.3–1.7 mm) having pruinose dark gray scutum with a small oval yellow pattern extending from the mid-posterior margin to the scutellum, a black 1^st^ flagellomere, dark maxillary palpus, gray halteres, and brown legs. Male epandrium inner-basally with a comb comprising seven long fused tubercle-like setae. Larva mines the thallus of *Rebouliahemisphaericaorientalis*.

#### Description.

**Figure 33.**
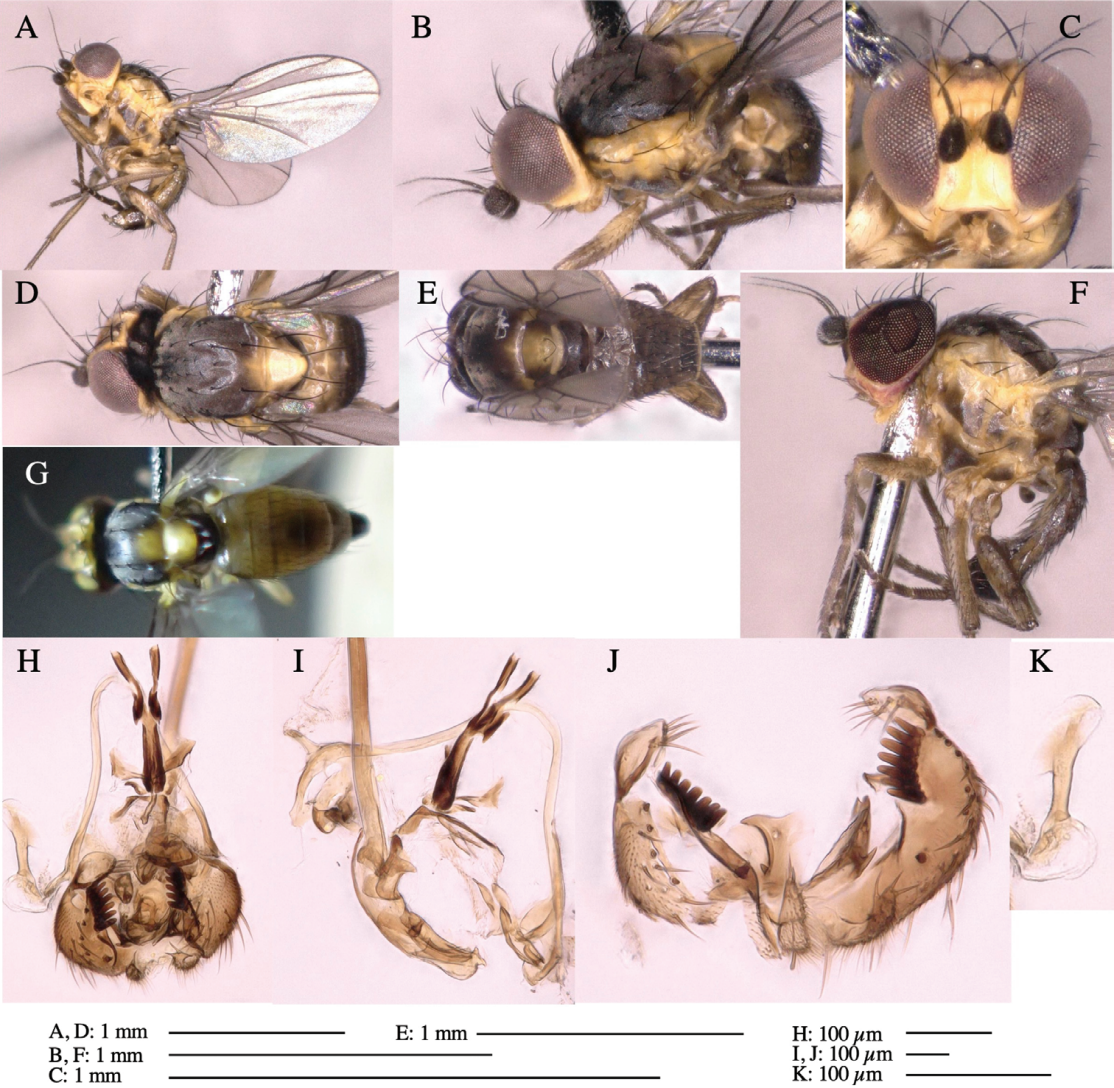
*Phytoliriomyzarebouliae*sp. nov. **A–D **holotype male **A** habitus **B** lateral **C** frontal **D** dorsal **E, F** paratype female (MK-AG-a424) **E** posterior **F** lateral **G** live female fly **H–K** male genitalia **H** whole genitalia, ventral **I** phallic complex, lateral **J** epandrium, ventral **K** ejaculatory apodeme, lateral.

**Adult male** (Fig. 33A–E).

***Head*:** Head light yellow, with ocellar tubercle dark brown, frons yellowish brown with reflective pruinosity, back of head dark brown excluding margins (Fig. 33C). Antenna porrect, first flagellomere black, pedicel and scape brown (Fig. 33B). Arista subbasal, black, pubescent. Clypeus, face, gena, parafacial and postgena yellow. Proboscis normal, yellow; palpus brown, cylindrical (Fig. 33C). ***Chaetotaxy*:** Front orbitals three pairs; one ori directed inward; two ors directed upward (Fig. 33B). Orbital setulae minute and erect, in a single row.

***Thorax*:** Thorax pruinose. Scutum pruinose gray, with a small yellow patch along midposterior margin, with a medial dark gray band on anterior 2/3 and a pair of dark gray bands along lateral margins (Fig. 33D). Scutellum light yellow with lateral corner brown, subscutellum light yellow. Mediotergite, anatergite and katatergite dark brown (Fig. 33B). Pleuron largely light yellow, with brown spots on anterior postpronotal lobe, lower margin of notopleuron, lower half of anepisternum and anepimeron, and venters of katepisternum and meron (Fig. 33B). Haltere dark brown. Calypter margin and hairs gray. Leg segments brownish, basal half of femur paler. ***Chaetotaxy*:** Scutum with 1+3 dorsocentrals, shortened anteriorly (Fig. 33D). Acrostichal setulae six or seven pairs largely in two rows. ***Wing*:** Wing length 1.7 mm, costa reaching M_1_ (Fig. 33A). Length of ultimate section of vein M_4_ divided by penultimate section 1.3.

***Abdomen*:** Abdomen dorsally subshiny brown; epandrium dark brown (Fig. 33E). ***Genitalia*:** (Fig. 33H–K) Epandrium rounded apically; inner-posterior margin with one tubercle-like seta; inner-anterior surface with a comb comprising seven fused long tubercle-like setae (Fig. 33J). Surstylus rounded, curved inwards, setose apically (Fig. 33J). Cercus narrow, setose. Subepandrial sclerite with a pair of plate-like arms, the dorsal lobes of which curve ventrally and basally with a spine directed ventrally (Fig. 33J). Hypandrium slightly sclerotized along outer margin (Fig. 33H). Postgonite bare, goose barnacle-shaped (Fig. 33H, I). Phallophorus sclerotized with deep incision below, articulated with phallapodeme, fused to epiphallus (Fig. 33I). Basiphallus with a dorsal sclerite, the anterior lobes of which extend laterally, supporting hypophallus (Fig. 33H, I). Hypophallus broad, membranous, covered with microtrichia dorsally and with distal margins serrated; basally with one pair of narrow sclerites, medially with one pair of fused linear sclerites (Fig. 33I). Paraphallus lobate, lightly sclerotized; paraphalli diverging, angled anteroventrally, jointed basally (Fig. 33H, I). Mesophallus dark, cylindrical, widest subbasally, slightly shorter than distiphallus (Fig. 33H). Distiphallus comprising one pair of stout tubules; basal half composed of ventral dark arrowhead-like sclerite and weaker medial region; distal half cylindrical, dorsally and laterally pigmented, with truncated unpigmented apex (Fig. 33I). Ejaculatory apodeme pale brown, fan-shaped with broad stalk; base wide to one side; sperm pump clear (Fig. 33K).

**Figure 34.**
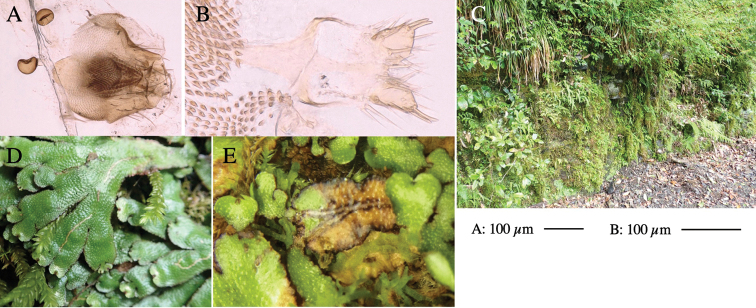
Female morphology and larval ecology of *Phytoliriomyzarebouliae*sp. nov. **A, B** female postabdomen **A** oviscape and spermatheca **B** tergite 10 **C** habitat at type locality **D, E** mined thalli of *Rebouliahemisphaericaorientalis* (**E** at type locality **F** at Okujisso).

**Female** (Fig. 33E–G). Similar to male. Wing length 2.3 mm. ***Postabdomen*:** (Fig. 34A, B) Oviscape dark brown, setigerous. Tergite 10 cruciform, laterally uniting narrow pleural sclerites. Each cercus with two stout, apical, trichoid sensilla, ½ length of cercus. Spermathecae semi-orbicular.

#### Etymology.

The specific name refers to the larval life within *Reboulia* thalli.

#### Japanese name.

Kainohi-jingasagoke-hamoguribae.

#### Host plant.

*Rebouliahemisphaericaorientalis* (Aytoniaceae).

#### Mine.

Larvae at first construct linear mines in the thallus at first, then enter the midrib, and pupate in the mine (Fig. 34D, E).

#### Biological notes.

 The habitats of this species are rocky cliffs in warm temperate evergreen forests (Fig. 34C). It is sympatric with *P.argentifasciata* in some localities. Our rearing records suggest that this species is bivoltine, with adults emerging in spring and summer.

#### Distribution.

Japan: Honshu, Shikoku, Tsushima Island (Fig. 31)

#### Remarks.

 This species resembles *P.marchantiae*,*P.lanternaria*, and *P.conocephali* in having a narrow yellow posterior margin of the scutum and a medial yellow stripe on the scutellum; it is distinguished from *P.marchantiae* by the number of tubercle-like setae in a comb of the male epandrium (7 in *P.rebouliae*; 8 in *P.marchantiae*) and by a tubercle-like seta at posterior end of inner margin (absent in *P.marchantiae*), and from *P.lanternaria* and *P.conocephali* by the number of tubercle-like setae on the surstylus of the male epandrium (0 in *P.rebouliae*; 1 or 2 in *P.lanternaria* and *P.conocephali*).

##### Species associated with *Wiesnerella*

### ﻿18.﻿ Phytoliriomyza wiesnerellae

Taxon classificationAnimaliaDipteraAgromyzidae

Kato sp. nov.

91A3EB1C-0EEA-520A-BF24-2E5D235EE9E8

https://zoobank.org/0F0C5717-47CB-4BB4-8D7B-CB92FAA79F29

Fig. 35 , 36

#### Material examined.

***Holotype*:** Japan: 1♂ (MK-AG-a400), Sendan-todoro, Izumi, Yatsushiro, Kumamoto Pref., 10-IV-2021 (as larva on *Wiesnerelladenudata*), emerged on 8-V-2021, NSMT-I-Dip 31992. ***Paratypes*:** Japan: 1♀ (MK-AG-a342), same data as holotype, NSMT-I-Dip 31993; 2♂1♀ (MK-AG-a303, a489, a464), Mt. Osuzu, Tsuno, Miyazaki Pref., 10-IV-2021 (as larva), emerged on 2–13-V-2021, NSMT-I-Dip 31994–31996.

#### Other material.

Japan: 1♂1♀, Sendan-todoro, Izumi, Yatsushiro, Kumamoto Pref. (32.5215°N, 130.888517°E, 710 m asl), 10-IV-2021 (as larva), emerged on 8–10-V-2021; 1♂, Itsuki, Yatsushiro, Kumamoto Pref., 23-III-2015 (as larva), emerged on 3-V-2015; 2♂5♀, Mt. Osuzu, Tsuno, Miyazaki Pref., 10-IV-2021 (as larva), emerged on 2–14-V-2021.

#### Diagnosis.

 A large dark species (wing length 2.0–2.3 mm) having subshiny dark gray scutum, yellow scutellum, black 1^st^ flagellomere, dark maxillary palpus, gray halteres, and dark brown legs. Male epandrium inner-basally with a protruding, plate-like arm bearing one strong, tubercle-like seta apically. Larva mines the thallus of *Wiesnerelladenudata*.

#### Description.

**Figure 35.**
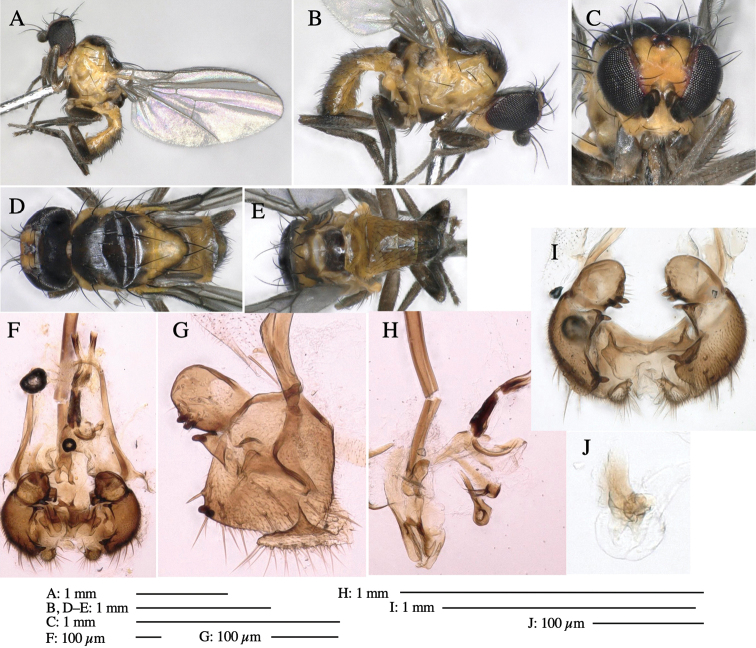
*Phytoliriomyzawiesnerellae*sp. nov. **A–D **holotype male **A** habitus **B** lateral **C** frontal **D** dorsal **E **paratype female (MK-AG-a342), posterior **F–J** male genitalia **F** whole genitalia, ventral **G** epandrium, lateral **H** phallic complex, lateral **I** epandrium, ventral **J** ejaculatory apodeme, lateral.

**Adult male** (Fig. 35A–D).

***Head*:** Head yellow; ocellar tubercle dark brown; front-orbital plate brown; back of head dark brown above foramen (Fig. 35C). Antenna porrect, first flagellomere black, pedicel dark brown and scape brown (Fig. 35B). Arista subbasal, pubescent. Face, gena, parafacial and postgena yellow. Proboscis normal, yellow; palpus brown, clubbed (Fig. 35C). ***Chaetotaxy*:** Front orbitals three pairs; one ori directed inward; two ors directed upward (Fig. 35B). Orbital setulae minute and erect, in a single row.

***Thorax*:** Thorax subshiny. Scutum dark brown, with marginal inflated yellow band adjoining scutellum (Fig. 35D). Scutellum yellow with lateral corner brown. Subscutellum yellow with narrow brown area along posterior margin. Mediotergite brown, katatergite yellow and anatergite yellow with lower brown patch (Fig. 35B). Pleuron yellow with small pale brown patches on central propleuron and lower anepisternum, and with distinct brown patches on lower katepisternum and lower meron (Fig. 35C). Haltere brown with yellow stalk. Calypter margin and hairs gray. Leg segments entirely dark brown (Fig. 35A). ***Chaetotaxy*:** Scutum with 1+3 dorsocentrals, shortened anteriorly (Fig. 35D). Acrostichal setulae seven pairs in two irregular rows. ***Wing*:** Wing length 2.0 mm, costa reaching M_1_ (Fig. 35A). Length of ultimate section of vein M_4_ divided by penultimate section 0.83.

***Abdomen*:** Abdomen dorsally subshiny yellow; epandrium dark brown (Fig. 35B). ***Genitalia*:** (Fig. 35F–J) Epandrium rounded apically, but angled dorso-posteriorly in a lateral view; inner-posterior margin with two tubercle-like setae on protruding arm; inner basal margin with a protruding, plate-like arm, which bears one strong tubercle-like seta apically (Fig. 35G, I). Surstylus rounded, directed inwards, setose on anterior margin; basally with two tubercle-like setae (Fig. 35I). Cercus narrow, setose. Subepandrial sclerite with one pair of plate-like arms (Fig. 35I). Hypandrium sclerotized (Fig. 35F). Postgonite bare and broadly rounded apically (Fig. 35H). Phallophorus with deep incision below (Fig. 35F), articulated with phallapodeme, fused to epiphallus (Fig. 35H). Basiphallus dorsolaterally sclerotized, with distal margin pigmented (Fig. 35H). Hypophallus broad and membranous; with one pair of pale narrow plate-like sclerites; medially with a pair of narrow, fused, ventrally incurved sclerites (Fig. 35H). Mesophallus dark, cylindrical, widest basally, ¾ length of distiphallus, tapering toward distiphallus (Fig. 35H). Distiphallus comprising one pair of stout tubules basally parallel to each other; basal 1/3 ventrally pigmented; medial 1/3 unpigmented; distal 1/3 pigmented with truncated, unpigmented apex (Fig. 35H). Ejaculatory apodeme dark, fan-shaped, with short broad stalk; base bulbous; sperm pump clear (Fig. 35J).

**Figure 36.**
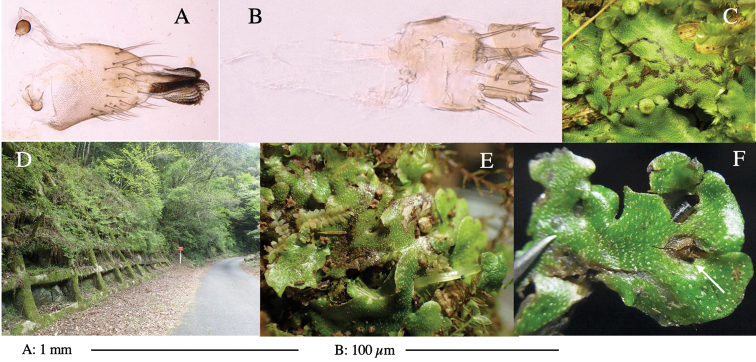
Female morphology and larval ecology of *Phytoliriomyzawiesnerellae*sp. nov. **A, B **female postabdomen **A **oviscape and spermatheca, **B** tergite 10 **C, E, F** mined thalli of *Wiesnerelladenudata* (**C** at Mt. Nokeeboshi **E, F** at type locality) **D** habitat at Mt. Osuzu. An arrow in **F** indicates an internal puparium.

**Female** (Fig. 35F). Similar to male, but larger, frons narrower, yellowish brown, abdomen darker. Wing length 2.3 mm. ***Postabdomen*:** (Fig. 36A, B) Oviscape dark brown, setigerous (Fig. 36A). Tergite 10 cruciform, laterally uniting narrow pleural sclerites (Fig. 36B). Each cercus with two stout, apical, trichoid sensilla, 1/3 length of cercus (Fig. 36B). Spermathecae subspheroidal, with truncate proximal ends (Fig. 36A).

#### Etymology.

 The specific name refers to the host plant genus, *Wiesnerella*.

#### Japanese name.

Azumazenigoke-hamoguribae.

#### Host plant.

*Wiesnerelladenudata* (Wiesnerellaceae).

#### Mine.

Larvae construct linear mines in the thallus in early instars, later expanding their mines, and pupate in the mines (Fig. 36C, E, F). It is difficult to find the mines, because older larvae often mine in the lower layer of the thallus, so that the mines are often not externally visible.

#### Biological notes.

The habitats of this species are mesic slopes in warm temperate evergreen forests. Our rearing records suggest that it is univoltine, with adults emerging from overwintered pupae in spring.

#### Distribution.

Japan: Kyushu (Fig. 31).

#### Remarks.

This species is superficially very similar to *P.dumortierae* in coloration of the head, thorax, abdomen, and legs, but is distinguished from the latter by the largely yellowish pleuron (pleuron yellow only in upper half in *P.dumortierae*). The anteroposteriorly flattened head and the absence of a comb of tubercle-like setae in the male epandrium of this species suggest that this is not closely related the other liverwort-associated species.

##### Species associated with *Conocephalum*

### ﻿19.﻿ Phytoliriomyza luna

Taxon classificationAnimaliaDipteraAgromyzidae

Kato sp. nov.

5C4DC810-1BD8-50B2-ACDA-588EEB5E8911

https://zoobank.org/F82867E-1994-4F2E-B904-1A3F4852F4CF

Figs 37 , 38

#### Material examined.

***Holotype*:** Japan: 1♂ (MK-AG-a401), Yashajin-toge, Minami-arupusu, Yamanashi Pref. (35.6327°N, 138.3519°E, 1110 m asl), 10-XII-2016 (as larva on *C.salebrosum*), emerged on 8-IV-2017, NSMT-I-Dip 31997. ***Paratypes*:** Japan: 1 ♀ (MK-AG-512), same data as holotype; emerged on 8-IV-2017 NSMT-I-Dip 31998; 1♂ (MK-AG-a245), Horoka, Kamishihoro, Hokkaido, 7-VI-2010 (as larva on *C.purpureorubrum*), emerged on 21-VI-2010, NSMT-I-Dip 31999; 1♂ (MK-AG-493), Aizankei, Kamikawa, Hokkaido, 4-X-2011 (as larva on *C.salebrosum*), emerged on 11-V-2012, NSMT-I-Dip 32000; 1♀ (MK-AG-591), Yachi, Kawaba, Gunma Pref., 14-IV-2012 (as larva on *C.purpureorubrum*), emerged on 4-V-2012, NSMT-I-Dip 32001; 1♀ (MK-AG-a362), Mt. Kiyosumi, Kamogawa, Chiba Pref.2, 31-III-2021 (as larva on *C.salebrosum*), emerged on 23-IV-2021, NSMT-I-Dip 32002.

#### Other material.

 Japan: On *Conocephalumsalebrosum*: 3♂3♀, Aizankei, Kamikawa, Hokkaido, 10-IV-2011 (as larva), emerged on 6–11-VI-2011; 2♀, Mt. Upepesanke, Shihoro, Kamishihoro, Hokkaido, 7-VI-2010 (as larva), emerged on 15–18-VI-2010; 1♂1♀, Horoka, Kamishihoro, Hokkaido, 7-VI-2010 (as larva), emerged on 21–26-VI-2010; ; 1♂, Tanneso, Rubeshibetsu, Hiroo, Hokkaido, 2-X-2011 (as larva), emerged on 3-V-2012; 4♂10♀, Yashajin-toge, Minami-arupusu, Yamanashi Pref., 10-XII-2016 (as larva), emerged on 26-III–11-IV-2016; 1♀, Nakabusa-onsen, Azumino, Nagano Pref., 9-VI-2013 (as larva), emerged on 29-IV-2013; 1♂2♀, Shirahone-onsen, Matsumoto, Nagano Pref., 15-X-2013 (as larva), emerged on 18–25-IV-2013.

On *Conocephalumpurpureorubrum*: 1♂1♀, Yachi, Kawaba, Gunma Pref., 15-X-2013 (as larva), emerged on 4–10-IV-2014; 1♂2♀, Haccho-toge, Ogano, Chichibu, Saitama Pref., 14-XI-2010 (as larva), emerged on 27-III–16-IV-2010.

#### Diagnosis.

 A large yellow species (wing length 2.7–2.9 mm) having pruinose yellow scutum with a medial and a pair of dark brown lateral stripes, entirely yellow scutellum, black 1^st^ flagellomere, yellow maxillary palpus, yellow halteres, and yellow legs. Male epandrium inner-distally with a long tubercle-like seta, and inner-basally with a comb consisting of 7–9 long fused tubercle-like setae. Larva mines the thallus of *Conocephalumsalebrosum* and *C.purpureorubrum*.

#### Description.

**Figure 37.**
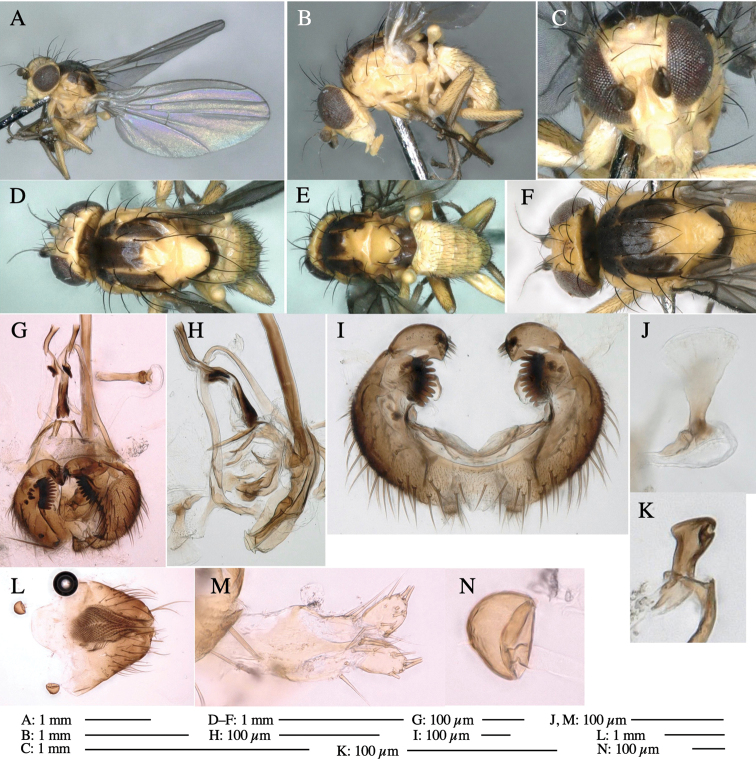
*Phytoliriomyzaluna*sp. nov. **A–E **holotype male **A** habitus **B** lateral **C** frontal **D** dorsal **E** posterior** F** paratype female (MK-AG-512), dorsal **G–K** male genitalia **G** whole genitalia, ventral and lateral **H** phallic complex, lateral **I** epandrium, ventral **J** ejaculatory apodeme, lateral **K** postgonite **L–N** female postabdomen at Aizankei **L** oviscape and spermatheca **M** tergite 10 **N** spermatheca.

**Adult male** (Fig. 37A–E).

***Head*:** Head entirely yellow including ocellar tubercle and back of head (Fig. 37C). Antenna porrect, first flagellomere black, pedicel and scape brown (Fig. 37B). Arista subbasal, pubescent. Face, gena, parafacial and postgena yellow. Proboscis normal, yellow; palpus yellow, cylindrical (Fig. 37C). ***Chaetotaxy*:** Front orbitals three pairs; one ori directed inward; two ors directed upward (Fig. 37B). Orbital setulae minute and erect, in a single row.

***Thorax*:** Thorax subshiny. Scutum yellow with a black medial stripe on anterior 2/3, one pair of black suborbicular presutural patches confluent with the medial stripe, and a pair of wide black bands (i.e., fused complex of intra-alar and supra-alar stripes) on anterior 7/8, which is confluent with the presutural patches (Fig. 37D). Scutellum and subscutellum entirely yellow (Fig. 37D). Mediotergite brown, but katatergite yellow, anatergite yellow with distal brown patch (Fig. 37E). Pleuron yellow with brown patches on propleuron centrally and on distal margins of anepisternum, katepisternum, and meron (Fig. 37B). Haltere yellow. Calypter margin and hairs gray. Leg segments yellow; tibia and tarsus darker (Fig. 37B). ***Chaetotaxy*:** Scutum with 1+3 dorsocentrals, shortened anteriorly (Fig. 37D). Acrostichal setulae 8–10 pairs in two irregular rows. ***Wing***: Wing length 2.7 mm, costa reaching M_1_ (Fig. 37A). Length of ultimate section of vein M_4_ divided by penultimate section 1.3.

***Abdomen*:** Abdomen dorsally subshiny yellow; epandrium dark brown (Fig. 37E). ***Genitalia*:** (Fig. 37G–J) Epandrium rounded apically; posterior end of inner margin with one tubercle-like seta; inner-anterior surface with a comb comprising 7–9 fused long tubercle-like setae (rarely unfused in part) and an irregular row of several (4–7) small tubercle-like setae immediately outward from the comb (Fig. 37I). Surstylus rounded, directed inwards, setose apically, with one (rarely 2 or 3) tubercle-like seta ventrally (Fig. 37I). Cercus narrow, setose. Subepandrial sclerite V-shaped (Fig. 37I). Hypandrium slightly sclerotized along outer margin (Fig. 37H). Postgonite bare, rounded apically, cleft subapically (Fig. 37K). Phallophorus with deep incision below, articulated with phallapodeme, fused to epiphallus (Fig. 37H). Basiphallus dorsally sclerotized (Fig. 37H). Hypophallus broad, membranous, with one pair of dark narrow long sclerites along margins; medially with one pair of fused linear sclerites (Fig. 37G, H). Mesophallus dark, cylindrical, widest subbasally, 3/5 shorter than distiphallus, tapering distally (Fig. 37H). Distiphallus comprising one pair of stout tubules parallel to each other at base; basal half composed of ventral dark subrectangular sclerite and weaker medial region; distal half cylindrical, dorsally pigmented; apex truncated, shortly flared unpigmented (Fig. 37H). Ejaculatory apodeme pale brown, fan-shaped with short broad stalk, stout base, and clear sperm pump (Fig. 37J).

**Female** (Fig. 37F). Similar to male, but larger, frons wider, black lateral stripes of scutum wider and almost confluent with medial stripe. Wing length 2.3 mm. ***Postabdomen*:** (Fig. 37L–N) Oviscape dark brown, setigerous (Fig. 37L). Tergite 10 cruciform, laterally uniting narrow pleural sclerites (Fig. 37M). Each cercus with two stout, apical, trichoid sensilla, ¼ length of cercus (Fig. 37M). Spermathecae semi-orbicular, with truncate proximal ends (Fig. 37N).

#### Variation.

The number of tubercle-like setae in a comb in the male genitalia varies from 7 to 9, but the variation did not involve in a geographical cline.

#### Etymology.

The specific name refers to the moon; a clear, rounded, yellow pattern on the scutum was likened to a full moon.

#### Japanese name.

Meigetsu-jagoke-hamoguribae.

#### Host plants.

*Conocephalumsalebrosum* and *C.purpureorubrum* (Conocephalaceae).

#### Mine.

**Figure 38:**
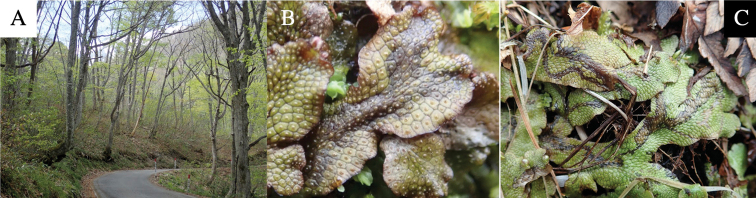
Larval ecology of *Phytoliriomyzaluna*sp. nov. **A **habitat at Mt. Shirouma **B, C** mined thalli of *Conocephalumsalebrosum* (**B** Jozankei **C** Yashajin-toge).

(Fig. 38C, D) Larvae construct linear mines in the thallus in early instars, later entering the midrib, and pupate in the mines.

#### Biological notes.

 The habitats of this species are stream banks and mesic slopes in subalpine coniferous forests dominated by *Abies* spp., *Picea* spp., and *Betula* spp. (Fig. 38A–B). This species was sympatric with *P.nigroflava* in some localities. Our rearing records suggest that this species is univoltine, and that adults emerge from overwintered pupae in spring.

#### Distribution.

**Figure 39.**
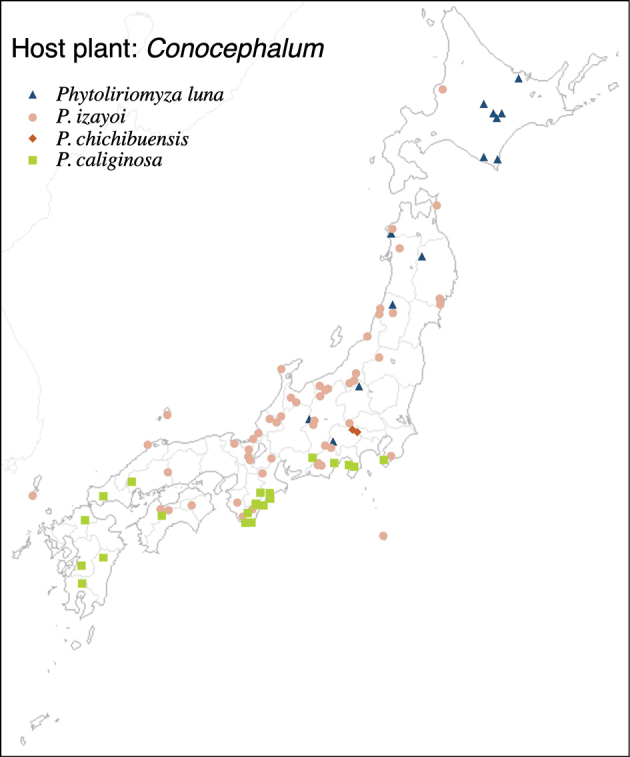
Locality records of three *Phytoliriomyza* species associated with *Conocephalum* spp.: *P.luna*, *P.izayoi*, *P.chichibuensis* and *P.caliginosa*.

Japan: Hokkaido, Honshu, Shikoku (Fig. 39).

#### Remarks.

 The characteristics of this species and the following three related species (*P.izayoi*,*P.chichibuensis*, and *P.caliginosa*) coincide with those of *Lemurimyza* described by Spencer (1965). These four new species can be distinguished from the four species previously placed in *Lemurimyza* species (*P.enormis* (Spencer, 1963), *P.admirabilis* (Spencer, 1965), *P.dorsata*, and *P.pectoralis* (Becker, 1908)) by the following characteristics: halteres yellow; maxillary palpus yellow; 1^st^ flagellomere black; scutum with one pair of dark lateral bands.

This species also resembles *P.islandica* and *P.bornholmensis* recorded respectively from Iceland and Denmark; it is distinguished from them by the lateral black bands terminating before reaching the scutellum (lateral black bands confluent with scutellum in the other two species); scutellum entirely yellow (scutellum with dark bands on lateral margins in the other two); male epandrium with a comb of 7–9 tubercle-like setae (6 in *P.islandica*, 8 in *P.bornholmensis*); male epandrium with one tubercle-like seta on middle inner surface (1 or 2 setae on middle inner margin in *P.islandica*; 3 setae along inner margin in *P.bornholmensis*); basal half of distiphallus curved outward and with weaker medial region (basal half of distiphallus curved outward and without weaker medial region in *P.islandica*; angular and with weaker medial region in *P.bornholmensis*).

This species resembles *P.pacifica* reported from North America but is distinguished by having a single pair of lateral bands on the scutum (two pairs of lateral stripes in *P.pacifica*), the number of tubercle-like setae on the male epandrium (7–9 in *P.luna*; 6 in *P.pacifica*), and position of the isolated tubercle-like seta on the inner surface of the male epandrium (distal margin in *P.luna*; basal inner surface in *P.pacifica*).

Among Japanese species, this species resembles *P.izayoi*,*P.chichibuensis*, and *P.caliginosa*, in size and in having a pair of dark broad lateral bands on scutum; it is distinguished from them by the wholly yellow scutellum (scutellum with dark bands on lateral margins in the other species).

### ﻿20.﻿ Phytoliriomyza izayoi

Taxon classificationAnimaliaDipteraAgromyzidae

Kato sp. nov.

4889FE47-0D59-5ABC-85AD-C98B34251F8D

https://zoobank.org/87323495-E2C3-44EF-A003-F6F9E4C24B6B

Figs 40 , 41

#### Material examined.

***Holotype*:** Japan: 1♂ (MK-AG-a402), Ashiu, Nantan, Kyoto Pref. (35.3261°N, 135.7239°E, 450 m asl), 8-V-2007 collected on thallus of *Conocephalumorientalis*, NSMT-I-Dip 32003. ***Paratypes*:** Japan: 1♀ (MK-AG-a262), Ashiu, Nantan, Kyoto Pref., 28-XI-1999 (as larva on *C.orientalis*), emerged on 17-IV-2000, NSMT-I-Dip 32004; 1♀ (MK-AG-520), Renge-onsen, Itoigawa, Niigata Pref., 14-VII-2009 (as larva on *C.salebrosum*), emerged on 5-V-2010, NSMT-I-Dip 32005; 1♂ (MK-AG-595), Mt. Hakusan, Hakusan, Ishikawa Pref., 3-V-2013 (as larva *C.orientalis*), emerged on 18-V-2013 NSMT-I-Dip 32006; 1♂1♀ (MK-AG-a249, 524), Nekata, Hamakita, Hamamatsu, Shizuoka Pref., 2-IV-2011 (as larva on *C.orientalis*), emerged on 18–20-IV-2011, NSMT-I-Dip 32007, 32007; 1♀ (MK-AG-574), Naiku, Oe, Fukuchiyama, Kyoto Pref., 17-III-2013 (as larva on *C.orientalis*), emerged on 5-IV-2013, NSMT-I-Dip 32009; 1♀ (MK-AG-624), Muramatsu, Iwakura, Sakyo-ku, Kyoto Pref., 5-IV-2019 (as larva on *C.orientalis*), emerged on 22-IV-2019, NSMT-I-Dip 32010; 1♀ (MK-AG-a392), Mt. Daimanji, Oki Is. Shimane Pref., 22-XI-2010 (as larva on *C.orientalis*), emerged on 7-IV-2011, NSMT-I-Dip 32011; 1♀ (MK-AG-587), Gakuen-ji, Bessho, Izumo, Shimane Pref., 11-I-2010 (as larva on *C.orientalis*), emerged on 14-IV-2011, NSMT-I-Dip 32012; 1♂ (MK-AG-a225), Koyadaira, Tokushima Pref., 22-IV-2019 (as larva on *C.orientalis*) ; emerged on 5-V-2019, NSMT-I-Dip 32013.

#### Other material.

 Japan: On *Conocephalumsalebrosum*: 1♂1♀, Renge-onsen, Itoigawa, Niigata Pref., 2-X-2011 (as larva), emerged on 29-IV-2012; 1♀, Sarukura, Hakuba, Nagano Pref., 9-VI-2013 (as larva), emerged on 28-VI-2013.

On *Conocephalumorientalis*: 5♀, Shokan-zawa, Mashike, Hokkaido, 4-X-2011 (as larva), emerged on 29-IV–6-V-2012; 1♂, Mt. Nanakura, Noshiro, Akita Pref., 14-X-2012 (as larva), emerged on 11-IV-2012; 1♂, Mt. Kiyosumi, Kamogawa, Chiba Pref., 24-I-2012 (as larva), emerged on 20-IV-2012; 5♂10♀, Nekata, Hamakita, Hamamatsu, Shizuoka Pref., 8-III-2012 (as larva), emerged on 27-III–26-IV-2012; 1♀, Takeda-gawa, Maruoka, Sakai, Fukui Pref., 18-III-2014 (as larva), emerged on 18-IV-2014; 3♂2♀, Akka, Iwaizumi, Iwate Pref., 20-II-2011 (as larva), emerged on 24-III–4-IV-2011; 2♂2♀, Suizu, Tsuruga, Fukui Pref., 11-III-2012 (as larva), emerged on 1–12-IV-2012; 3♂3♀, Seryo, Sakyo-ku, Kyoto Pref., 6-IV-2010 (as larva), emerged on 26-IV–12-V-2010; 2♂3♀, Naiku, Oe, Fukuchiyama, Kyoto Pref., 17-III-2013 (as larva), emerged on 4–18-IV-2013; 3♂6♀, Kibune, Sakyo-ku, Kyoto Pref., 6-IV-2010 (as larva), emerged on 20-IV-2010; 1♂2♀, Muramatsu, Iwakura, Sakyo-ku, Kyoto Pref., 28-IV-2015 (as larva), emerged on 10–18-IV-2015; 1♀, Mt. Kanpu, Ino, Agawa, Kochi Pref., 10-X-2016 (as larva), emerged on 30-II-2016.

On *Conocephalumpurpureorubrum*: 1♀, Mt. Kiyosumi, Kamogawa, Chiba Pref., 14-IV-2010 (as larva), emerged on 2-V-2010; 1♀, Shirabiso-toge, Kamimura, Iida, Nagano Pref., 14-X-2011 (as larva), emerged on 28-IV-2011; 1♂, Kibune, Sakyo-ku, Kyoto Pref., 94-IV-2012 (as larva), emerged on 24-V-2012.

#### Diagnosis.

 A large yellow species (wing length 2.4–2.5 mm) having a pruinose yellow scutum with a medial and a pair of dark brown lateral stripes, a yellow scutellum with dark lateral corners, black 1^st^ flagellomere, yellow maxillary palpus, yellow halteres, and yellow legs. Male epandrium inner-distally with a long tubercle-like seta, and inner-basally with a comb consisting of 9–12 long fused tubercle-like setae. Larva mines the thallus of *Conocephalumsalebrosum* and *C.orientalis*.

#### Description.

**Figure 40:**
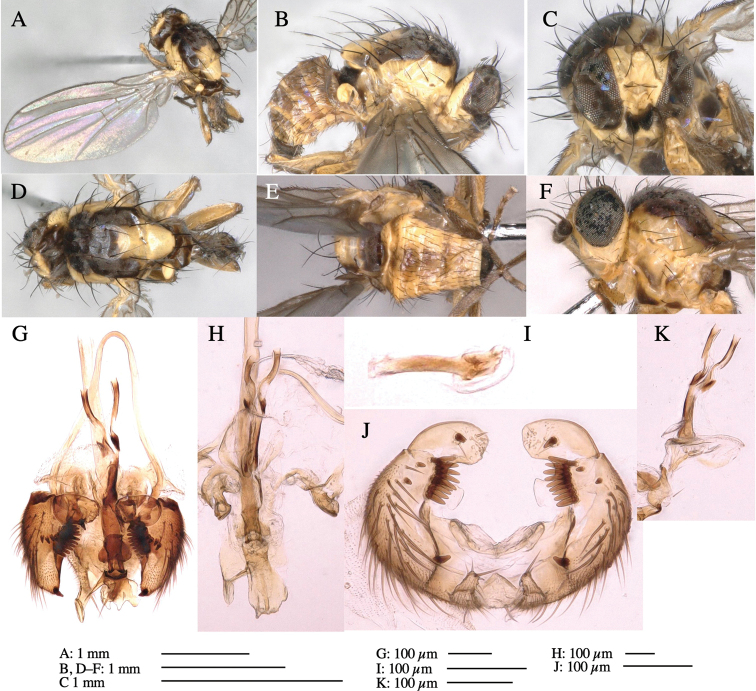
. *Phytoliriomyzaizayoi*sp. nov. **A–D **holotype male **A** habitus **B** lateral **C** frontal **D** dorsal **E, F** paratype female (MK-AG-a262) **E** posterior **F** lateral **G–K** male genitalia (**G–I** type locality** J, K** Renge-onsen) **G** whole genitalia, ventral **H** phallic complex, ventral **I** ejaculatory apodeme, dorsal **J** epandrium, ventral **K** phallic complex, lateral.

**Adult male** (Fig. 40A–D).

***Head*:** Head entirely yellow including ocellar tubercle and back of head (Fig. 40C). Antenna porrect, first flagellomere black, pedicel and scape brown (Fig. 40B). Arista subbasal, pubescent. Face, gena, parafacial and postgena yellow. Proboscis normal, yellow; palpus yellow, cylindrical (Fig. 40C). ***Chaetotaxy*:** Front orbitals three pairs; one ori directed inward; two ors directed upward (Fig. 40B). Orbital setulae minute and erect, in a single row.

***Thorax*:** Thorax subshiny. Scutum yellow with a black medial stripe on anterior 2/3, one pair of black suborbicular presutural patches confluent with the medial stripe, and a pair of wide black bands (i.e., fused complex of intra-alar and supra-alar stripes) on anterior 7/8, which is confluent with the presutural patches (Fig. 40D). Scutellum yellow with lateral margins brown (Fig. 40A, B). Subscutellum yellow. Mediotergite brown, but katatergite yellow, anatergite yellow with distal brown patch (Fig. 40B). Pleuron yellow with brown patches on propleuron centrally and on distal margins of notopleuron, anepisternum, katepisternum and meron (Fig. 40C). Haltere yellow. Calypter margin and hairs gray. Leg segments yellow; tibia and tarsus darker (Fig. 40A). ***Chaetotaxy*:** Scutum with 1+3 dorsocentrals, shortened anteriorly (Fig. 40D). Acrostichal setulae in two rows. ***Wing***: Wing length 2.4 mm, costa reaching M_1_ (Fig. 40A). Length of ultimate section of vein M_4_ divided by penultimate section 0.87.

***Abdomen*:** Abdomen dorsally subshiny grayish yellow; epandrium dark brown (Fig. 40B). ***Genitalia*:** (Fig. 40G, K) Epandrium rounded apically; posterior end of inner margin with one tubercle-like seta; inner-anterior surface with a comb comprising 9–12 fused long tubercle-like setae (rarely unfused in part) and an irregular row of several (3–5) small tubercle-like setae immediately outward from the comb (Fig. 40J). Surstylus rounded, directed inwards, setose apically, with one (rarely 2 or 3) long tubercle-like seta on posterior margin (Fig. 40J). Cercus narrow, setose. Subepandrial sclerite V-shaped in a posterior view, dorsal lobe with one seta (Fig. 40J). Hypandrium slightly sclerotized along outer margin (Fig. 40G). Postgonite bare, goose barnacle-shaped, and cleft apically (Fig. 40H). Phallophorus with deep incision below, articulated with phallapodeme, fused to epiphallus (Fig. 40H, K). Basiphallus with dark broad lateral plate on left side and sclerotized anterodorsal margin (Fig. 40H). Hypophallus broad, membranous, and bilaterally asymmetrical; with a dark narrow sclerite on right side; medially with a pair of fused linear sclerites (Fig. 40K). Mesophallus dark, cylindrical, widest subbasally, 3/5 length of distiphallus, tapering distally (Fig. 40K). Distiphallus comprising one pair of stout tubules basally parallel to each other; basal half composed of ventral dark cuneiform sclerite and weaker medial region; distal half cylindrical, dorsally pigmented; with truncated, shortly flared unpigmented apex (Fig. 40H, K). Ejaculatory apodeme pale brown, fan-shaped with short broad stalk and clear sperm pump (Fig. 40I).

**Figure 41.**
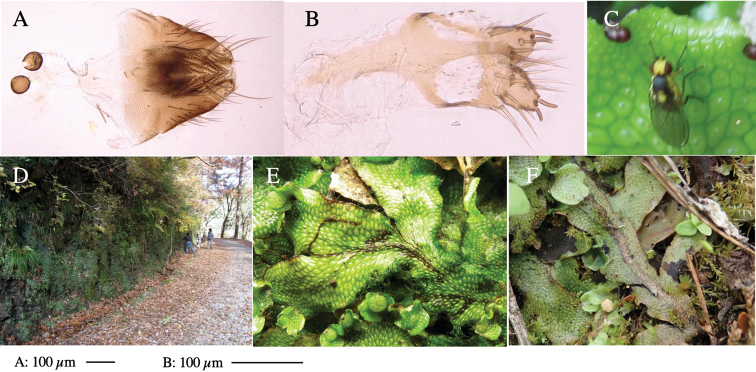
Female morphology and larval ecology of *Phytoliriomyzaizayoi*sp. nov. **A, B **female postabdomen **A** oviscape and spermatheca **B** tergite 10 **C** a female flay on *Conocephalumorientalis* at type locality **D** habitat at type locality **D, E** mined thalli (**D***Conocephalumorientalis* at Hamakita **F***C.salebrosum*at Renge-onsen).

**Female** (Fig. 40E, F). Similar to male, but larger, frons wider. Wing length 2.3&nbsp;mm. ***Postabdomen*:** (Fig. 41A, B) Oviscape dark brown, setigerous (Fig. 41A). Tergite 10 cruciform, laterally uniting narrow pleural sclerites (Fig. 41B). Each cercus with two stout, apical, trichoid sensilla, ¼ length of cercus (Fig. 41B). Spermathecae semi-orbicular, with truncate proximal ends (Fig. 41A).

#### Variation.

The number of tubercle-like setae in a comb in the male genitalia varied from 9 to 12. Although the number varied among individuals within a population and even between left and right sides of the epandrium in an individual, and the number was generally greater in western Honshu and Shikoku than in northern regions.

#### Etymology.

 The specific name *izayoi* is a Japanese word meaning 16^th^ moon, and refers to the non-circular yellow pattern of scutum.

#### Japanese name.

Izayoi-jagoke-hamoguribae.

#### Host plants.

*Conocephalumsalebrosum*,*C.orientalis* and *C.purpureorubrum* (Conocephalaceae).

#### Mine.

(Fig. 41G–I) Larvae construct linear mines in the thallus in early instars, later entering the midrib, and pupate in the mines.

#### Biological notes.

 The habitats of this species are stream banks and mesic slopes in temperate deciduous forests dominated by *Faguscrenata*, *Quercuscrispula* and *Cryptomeriajaponica* (Fig. 41D–F). It is sympatric with *P.luteola* and *P.conocephali* in some localities. Our rearing records suggest that this species is univoltine, and that adults emerge from overwintered pupae in spring. The female’s oviposition behavior on *C.orientalis* thalli was observed on 1 July 2021 in a beech forest at Renge-Onsen.

#### Distribution.

Japan: Hokkaido, Honshu, Shikoku (Fig. 39).

#### Remarks.

 This species resembles *P.islandica* and *P.bornholmensis*; it is distinguished from them based on the following characters: lateral black bands terminate before reaching scutellum (lateral black bands confluent with scutellum in the latter two); male epandrium with a comb of 9–12 tubercle-like setae (6 in *P.islandica*, 8 in *P.bornholmensis*); male epandrium with one tubercle-like seta on middle inner surface (1–2 on middle inner margin in *P.islandica*; three on inner margin in *P.bornholmensis*); basal half of distiphallus curved outward and with weaker medial region (basal half of distiphallus curved outward and without weaker medial region in *P.islandica*; angular and with weaker medial region in *P.bornholmensis*). This species also resembles *P.admirabilis* recorded from Nepal; it is distinguished from the latter based on the following characters: halteres yellow (black in the latter); male genitalia lack paraphallus (paraphallus present in the latter); surstylus with one tubercle-like seta (without tubercle-like seta in the latter); ejaculatory apodeme with a short broad stalk (with a slender stalk in the latter).

Among the Japanese species, *P.izayoi* resembles *P.luna*,*P.chichibuensis*, and *P.caliginosa* in size and in having a pair of dark broad lateral bands on the scutum; it is distinguished from *P.luna* by the dark-sided scutellum (scutellum only yellow in *P.luna*), from *P.chichibuensis* and *P.caliginosa* by the dark lateral bands not confluent with medial stripe (lateral bands confluent with medial stripe in the other species) and by the tubercle-like setae borne on the distal margin of the male epandrium (tubercle-like setae borne on inner surface of epandrium in the others).

### ﻿21.﻿ Phytoliriomyza chichibuensis

Taxon classificationAnimaliaDipteraAgromyzidae

Kato sp. nov.

FC6F3E2C-F587-56C7-995E-2ED5F310F63F

https://zoobank.org/7087598F-48A6-4158-B88E-CE998922EFD4

Fig. 42

#### Material examined.

***Holotype*:** Japan: 1♂ (MK-AG-a547), Mt. Futago, Ogano, Chichibu-gun, Saitama Pref. (36.0702°N, 138.8672°E, 930 m asl), 26-III-2021 (as larva on *Conocephalumpurpureorubrum*), emerged on 21-IV-2021, NSMT-I-Dip 32014. ***Paratypes*:** Japan: 1♂ (MK-AG-a387), Kanna-gawa, Nakatsugawa, Chichibu, Saitama Pref., 26-III-2021 (as larva), emerged on 20-IV-2021, NSMT-I-Dip 32015; 1♂ (MK-AG-a393), Kanna-gawa, Nakatsugawa, Chichibu, Saitama Pref., 13-III-2017 (as larva, emerged on 12-IV-2017, NSMT-I-Dip 32016.

#### Other material.

Japan: 1♂, Mt. Futago, Ogano, Chichibu-gun, Saitama Pref., 26-III-2021 (as larva), emerged on 21-IV-2021.

#### Diagnosis.

 A large yellow species (wing length 2.2–2.9 mm) having a pruinose dark brown scutum with an obscure oval yellow pattern extending from the mid-posterior margin to the scutellum, a black 1^st^ flagellomere, yellow maxillary palpus, yellow halteres, and yellow legs. Male epandrium inner-laterally with a long tubercle-like seta, and inner-basally with a comb consisting of six long, fused, tubercle-like setae. Larva mines the thallus of *Conocephalumpurpureorubrum*.

#### Description.

**Figure 42.**
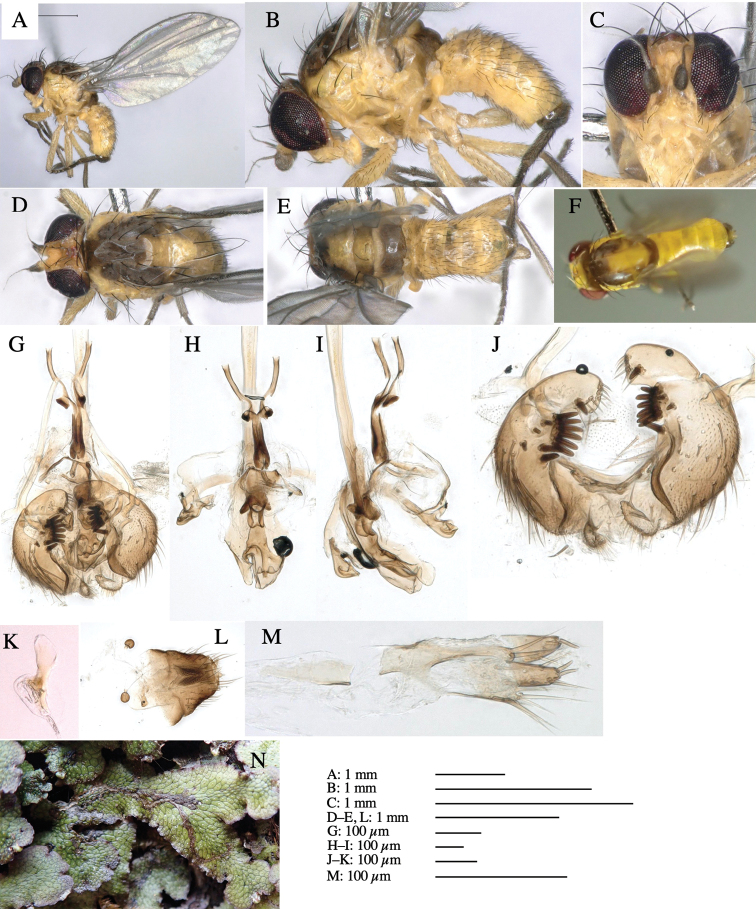
*Phytoliriomyzachichibuensis*sp. nov. **A–E **holotype male **A** habitus **B** lateral **C** frontal **D** dorsal **E** posterior **F** live male fly **G–K** male genitalia **G** whole genitalia, ventral **H, I** phallic complex, ventral and lateral **J** epandrium, ventral **K** ejaculatory apodeme, lateral, **L, M** female postabdomen **L** oviscape and spermatheca **M** tergite 10 **N** mined thallus of *Conocephalum* sp. 2.

**Adult male** (Fig. 42A–E).

***Head*:** Head entirely yellow including ocellar tubercle and back of head (Fig. 42C). Antenna porrect, first flagellomere brown, pedicel and scape brown (Fig. 42B). Arista subbasal, pubescent. Face, gena, parafacial and postgena yellow. Proboscis normal, yellow; palpus yellow, cylindrical (Fig. 42C). ***Chaetotaxy*:** Front orbitals three pairs; one ori directed inward; two ors directed upward (Fig. 42D). Orbital setulae minute and erect, in a single row.

***Thorax*:** Thorax pruinose. Scutum yellow with a black medial stripe on anterior 2/3, one pair of black suborbicular presutural patches confluent with the medial stripe, and a pair of wide black bands (i.e., fused complex of intra-alar and supra-alar stripes) on anterior 7/8, which is confluent with the presutural patches and the medial stripe (Fig. 42D). Scutellum yellow with lateral margins brown. The yellow patch ranging from posterior scutum to scutellum oblong and ill-defined. Subscutellum yellow. Mediotergite brown, but katatergite yellow, anatergite yellow with distal brown patch (Fig. 42B). Pleuron yellow with brown patches on propleuron centrally and on distal margins of notopleuron, anepisternum, katepisternum and meron (Fig. 42B). Haltere yellow. Calypter margin and hairs gray. Leg segments yellow; tibia and tarsus darker. ***Chaetotaxy*:** Scutum with 1+3 dorsocentrals, shortened anteriorly (Fig. 42D). Acrostichal setulae eight or nine pairs in two irregular rows. ***Wing*:** Wing length 2.4 mm, costa reaching M_1_ (Fig. 42A). Length of ultimate section of vein M_4_ divided by penultimate section 0.87.

***Abdomen*:** Abdomen dorsally subshiny yellow; epandrium dark brown (Fig. 42E). ***Genitalia*:** (Fig. 42G–K) Epandrium rounded apically; inner-lateral surface with one long tubercle-like seta; inner-anterior surface with a comb comprising six fused, long, tubercle-like setae and an irregular row of several (2–5) small tubercle-like setae immediately outward from the comb (Fig. 42J). Surstylus rounded, directed inwards, setose apically, with one tubercle-like seta on posterior margin (Fig. 42J). Cercus narrow, setose. Subepandrial sclerite V-shaped in a posterior view (Fig. 42J). Hypandrium slightly sclerotized along outer margin (Fig. 42G). Postgonite bare, goose barnacle-shaped, pointed, and cleft apically (Fig. 42I). Phallophorus with deep incision below (Fig. 42H), articulated with phallapodeme, fused to epiphallus (Fig. 42I). Basiphallus with a dark anterolateral plate on left side (Fig. 42G). Hypophallus broad, membranous, and bilaterally asymmetrical; with a dark narrow sclerite on right side; medially with a pair of fused linear sclerites (Fig. 42H–I). Mesophallus dark, cylindrical, widest subbasally, as long as distiphallus, tapering distally (Fig. 42H, I). Distiphallus comprising one pair of stout tubules basally parallel to each other; basal half composed of ventral dark cuneiform sclerite and weaker medial region; distal half cylindrical, dorsally pigmented; with truncated, shortly flared unpigmented apex (Fig. 42H, I). Ejaculatory apodeme pale brown, with rounded blade and broad stalk; base wide to one side; sperm pump clear (Fig. 42K).

**Female** (Fig. 42F). Similar to male, but larger, frons wider. Wing length 2.3 mm. ***Postabdomen*:** (Fig. 42L, M) Oviscape dark brown, setigerous (Fig. 42L). Tergite 10 cruciform, laterally uniting narrow pleural sclerites (Fig. 42M). Each cercus with two stout, apical, trichoid sensilla, ¼ length of cercus (Fig. 42M). Spermathecae semi-orbicular, with truncate proximal ends (Fig. 42L).

#### Etymology.

The specific name (*chichibu*) refers to the region where this species was found.

#### Japanese name.

Shungetsu-jagoke-hamoguribae.

#### Host plants.

*Conocephalumsalebrosum* and *C.purpureorubrum* (Conocephalaceae).

#### Mine.

(Fig. 42N) Larvae construct linear mines in the thallus in early instars, later entering the midrib, and pupate in the mines.

#### Biological notes.

The habitats of this species are stream banks and cliffs in temperate deciduous forests dominated by *Quercuscrispula*. Our rearing records suggest that this species is univoltine, and that adults emerge from overwintered pupae in spring.

#### Distribution.

Japan: Honshu, around Chichibu mountains in the Kanto Region (Fig. 39).

#### Remarks.

This species resembles *P.islandica* and *P.bornholmensis* in yellow pattern of scutum; it is distinguished from *P.islandica* by the distiphallus with weaker medial region (distiphallus without weaker medial region in *P.islandica*), from *P.bornholmensis* by the number of tubercle-like setae in a comb of the male epandrium (6 in *P.chichibuensis*; 8 in *P.bornholmensis*). This species also resembles *P.caliginosa* in yellow pattern of scutum; it is distinguished from the latter by the number of tubercle-like setae in a comb of the male epandrium (6 in *P.chichibuensis*; 8–11 in *P.caliginosa*) and by the color of the first flagellomere (brown in *P.chichibuensis*; black in *P.caliginosa*).

### ﻿22.﻿ Phytoliriomyza caliginosa

Taxon classificationAnimaliaDipteraAgromyzidae

Kato sp. nov.

4C8994C0-1E1F-5D90-B4CE-259490FFD52B

https://zoobank.org/8A5B5E5E-3A7D-416C-8CAE-2E0BC4108A1B

Fig. 43

#### Material examined.

***Holotype*:** Japan: 1♂ (MK-AG-a403), Kuki, Owase, Mie Pref. (34.0297°N, 136.2506°E, 270 m asl), 1-IV-2009 (as larva), emerged on 9-IV-2009, NSMT-I-Dip 32017. ***Paratypes*:** Japan: 1♀ (MK-AG-a224), same data as holotype, emerged on 11-IV-2009, NSMT-I-Dip 32018; 1♀ (MK-AG-a250), Asahi-daki, Shuzenji, Izu, Shizuoka Pref., 7-III-2012 (as larva), emerged on 28-III-2012, NSMT-I-Dip 32019; 1♂1♀ (MK-AG-a238, a239), Yunokuchi-onsen, Kiwa, Kumano, Mie Pref., 9–13-IV-2019 (as larva), emerged on 22-IV-2019, NSMT-I-Dip 32020, 32021; 1♂1♀ (MK-AG-a237, a390), Mt. Kosho, Asakura, Fukuoka Pref., 11-IV-2010 (as larva), emerged on 24-IV-2010, NSMT-I-Dip 32022.

#### Other material.

Japan: 1♂1♀, Mt. Nokogiri, Kyonan, Awa, Chiba Pref., 24-I-2012 (as larva), emerged on 24-IV–8-V-2012 1♂1♀, Asahi-daki, Shuzenji, Izu, Shizuoka Pref., 7-III-2012 (as larva), emerged on 28-III–1-IV-2012; 3♂3♀, Tamaki-gawa, Totsugawa, Yoshino, Nara Pref., 9-III-2014 (as larva), emerged on 26-III–9-IV-2014; 2♂1♀, Wabuka, Kushimoto, Wakayama Pref., 4-III-2012 (as larva), emerged on 26-III–12-IV-2012; 1♂2♀, Kibune, Sakyo-ku, Kyoto Pref., 1-III-2011 (as larva), emerged on 25-III–3-IV-2011; 2♂, Yasukawa-keikoku, Tanabe, Wakayama Pref., 9-VII-2012 (as larva), emerged on 16–21-IV-2002; 1♂1♀, Takinohai, Kozagawa, Wakayama Pref., 13-IV-2014 (as larva), emerged on 2–15-V-2014; 1♀, Yoshiwa, Hatsukaichi, Hiroshima Pref., 30-V-2014 (as larva), emerged on ?-V-2014; 2♀, Mt. Ryuso, Aoi, Shizuoka Pref., 13-X-2015 (as larva), emerged on 14-IV-2015; 1♂, Shinkawa-keikoku, Kirishima, Kagoshima Pref., 31-III-2017 (as larva), emerged on 16-IV-2017.

#### Diagnosis.

 A large yellow species (wing length 2.1–2.3 mm) having pruinose dark brown scutum with an obscure oval yellow pattern extending from the mid-posterior margin to the scutellum, a black 1^st^ flagellomere, yellow maxillary palpus, yellow halteres, and yellow legs. Male epandrium inner-laterally with a long tubercle-like seta and inner-basally with a comb comprising eight or nine long fused tubercle-like setae. Larva mines the thallus of *Conocephalumorientalis*.

#### Description.

**Figure 43.**
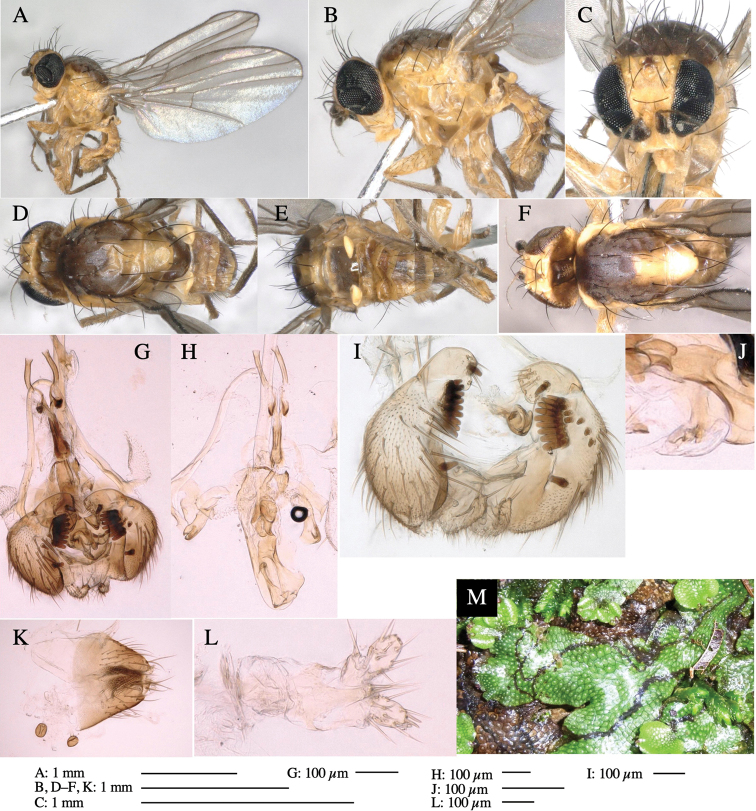
*Phytoliriomyzacaliginosa*sp. nov. **A–E **holotype male **A** habitus **B** lateral **C** frontal **D** dorsal **E** posterior **F** paratype female (MK-AG-a224) **G–J** male genitalia **G** whole genitalia, ventral **H **phallic complex, ventral **I** epandrium, ventral **J** ejaculatory apodeme, lateral **K, L** female postabdomen **K** oviscape and spermatheca **L** tergite 10 **M **mined thallus of *Conocephalumorientalis* at Tsurara-kannon.

**Adult male** (Fig. 43A–E).

***Head*:** Head entirely yellow including ocellar tubercle and back of head (Fig. 43C). Antenna porrect, first flagellomere black, pedicel and scape brown (Fig. 43B). Arista subbasal, pubescent. Face, gena, parafacial and postgena yellow. Proboscis normal, yellow; palpus yellow, cylindrical (Fig. 43C). ***Chaetotaxy*:** Front orbitals three pairs; one ori directed inward; two ors directed upward (Fig. 43B). Orbital setulae minute and erect, in a single row.

***Thorax*:** Thorax pruinose. Scutum yellow with a black medial stripe on anterior 2/3, one pair of black suborbicular presutural patches confluent with the medial stripe, and a pair of wide black bands (i.e., fused complex of intra-alar and supra-alar stripes) on anterior 7/8, which is confluent with the presutural patches and the medial stripe (Fig. 43D). Scutellum yellow with lateral margins brown. The yellow patch ranging from posterior scutum to scutellum oblong and ill-defined. Subscutellum yellow except for brown posterior half. Mediotergite brown, but katatergite yellow, anatergite yellow with distal brown patch (Fig. 43E). Pleuron yellow with brown patches on propleuron centrally and on distal margins of notopleuron, anepisternum, katepisternum and meron (Fig. 43B). Haltere yellow. Calypter margin and hairs gray. Leg segments yellow; tibia and tarsus darker (Fig. 43A). ***Chaetotaxy*:** Scutum with 1+3 dorsocentrals, shortened anteriorly. Acrostichal setulae eight or nine pairs in two irregular rows. ***Wing*:** Wing length 2.2 mm, costa reaching M_1_ (Fig. 43A). Length of ultimate section of vein M_4_ divided by penultimate section 1.1.

***Abdomen*:** Abdomen dorsally subshiny grayish yellow; epandrium dark brown (Fig. 43E). ***Genitalia*:** (Fig. 43G–J) Epandrium rounded apically; inner-lateral surface with one long tubercle-like seta; inner-anterior surface with a comb comprising eight or nine fused long tubercle-like setae and an irregular row of several (4–5) small tubercle-like setae immediately outward from the comb (Fig. 43I). Surstylus rounded, directed inwards, setose apically, with one tubercle-like seta on posterior margin (Fig. 43I). Cercus narrow, setose. Subepandrial sclerite V-shaped in a posterior view (Fig. 43I). Hypandrium slightly sclerotized along outer margin (Fig. 43G). Postgonite bare, goose barnacle -shaped, rounded apically (Fig. 43H). Phallophorus with deep incision below, articulated with phallapodeme, fused to epiphallus (Fig. 43H). Basiphallus with broad pale plate on left side and lightly sclerotized anterodorsal margin (Fig. 43H). Hypophallus broad, membranous, and bilaterally asymmetrical; with a dark narrow sclerite on right side; medially with a pair of fused linear sclerites (Fig. 43G, H). Mesophallus dark, cylindrical, widest subbasally, as long as distiphallus, tapering distally (Fig. 43H). Distiphallus comprising one pair of stout tubules basally parallel to each other; basal half composed of dark short cuneiform sclerite and weaker medial region; distal half cylindrical, dorsally pigmented; with truncated, shortly flared unpigmented apex (Fig. 43H). Ejaculatory apodeme pale brown, with fan-shaped blade and broad stalk; base wide to one side; sperm pump clear (Fig. 43J).

**Female** (Fig. 43F). Similar to male. Wing length 2.3 mm. ***Postabdomen*:** (Fig. 43K–L) Oviscape dark brown, setigerous (Fig. 43K). Tergite 10 cruciform, laterally uniting narrow pleural sclerites. Each cercus with two stout, apical, trichoid sensilla, ¼ length of cercus (Fig. 43L). Spermathecae semi-orbicular, with truncate proximal ends (Fig. 43K).

#### Variation.

The number of tubercle-like setae in a comb in the male genitalia varies from 8 to 9, but the variation does not involve a geographic cline.

#### Etymology.

 The specific name (*caliginosus* = misty) refers to the obscure yellow mark on the scutum, which resembles a dim spring moon.

#### Japanese name.

Oborozuki-jagoke-hamoguribae.

#### Host plant.

*Conocephalumorientalis* (Conocephalaceae).

#### Mine.

Larvae construct linear mines in the thallus in early instars, later entering the midrib, and pupate in the mines (Fig. 43M).

#### Biological notes.

 The habitats of this species are stream banks and mesic slopes in warm temperate evergreen forests dominated by *Castanopsiscuspidata* and *Quercussessilifolia*. This species was sympatric with *P.pallidofasciata* and *P.conocephali* in some localities. Our rearing records suggested that this species was univoltine; it overwinters as a pupa and the adult emerged in spring.

#### Distribution.

Japan: Honshu, Shikoku, Kyushu (Fig. 39).

#### Remarks.

 This species resembles *P.islandica* and *P.bornholmensis* recorded respectively from Iceland and Denmark; it is distinguished from *P.islandica* by the number of tubercle-like setae in a comb of the male epandrium (8–9 in *P.caliginosa*, 6 in *P.islandica*), and from *P.bornholmensis* by the form of the sclerite of the basal distiphallus (short cuneiform in *P.caliginosa*; long triangular in *P.bornholmensis*). This species also resembles *P.igniculus* and *P.chichibuensis* in the yellow oblong obscure pattern of the scutum; it is distinguished from *P.igniculus* by the absence of a pair of lateral brown patches on the 2^nd^ abdominal tergite (present in *P.igniculus*), and from *P.chichibuensis* by the number of tubercle-like setae in a comb of the male epandrium (8–11 in *P.caliginosa*; 6–8 in *P.chichibuensis*).

### ﻿23.﻿ Phytoliriomyza ugetsu

Taxon classificationAnimaliaDipteraAgromyzidae

Kato sp. nov.

8DA6D0FD-C704-515D-B658-97C2EDA2E939

https://zoobank.org/C786D7C2-26CE-42B4-B67F-7F74FD146759

Fig. 44

#### Material examined.

***Holotype*:** Japan: 1♂ (MK-AG-a404), Mt. Kora, Kurume, Fukuoka Pref. (33.2954°N, 130.5747°E, 180 m asl), 11-IV-2010 (as larva), emerged on 20-IV-2010, NSMT-I-Dip 32024. ***Paratypes*:** Japan: 1♂2♀ (MK-AG-a466, 547, 554), same data as holotype, emerged on 1–3-I-2010, NSMT-I-Dip 32025–32027; 1♀ (MK-AG-532), Ashizuri-misaki, Tosashimizu, Kochi Pref., 26-II-2011 (as larva), emerged on 31-III-2011, NSMT-I-Dip 32028; 2♀ (MK-AG-559, 563), Han-yama, Yaku Is., Kumage, Kagoshima Pref., 29-III-2017 (as larva), emerged on 8-IV-2017, NSMT-I-Dip 32029, 32030.

#### Other material.

 Japan: 6♂6♀, Mikisato, Owase, Mie Pref., 30-XII-2020 (as larva), emerged on 20-II–15-III-2021; 3♂5♀, Tategasaki, Kumano, Mie Pref., 23-IV-2021 (as larva), emerged on 26-IV–18-V-2021; 1♀, Mt. Kosho, Asakura, Fukuoka Pref., 11-IV-2010 (as larva), emerged on 18-IV-2010; 12♂15♀, Mt. Kora, Kurume, Fukuoka Pref., 29-IV-2008 (as larva), emerged on 1–3-V-2008; 1♂2♀, Mt. Osuzu, Tsuno, Miyazaki Pref., 15-XII-2020 (as larva), emerged on 23–28-II-2013; 1♂2♀, Shinkawa-keikoku, Kirishima, Kagoshima Pref., 31-III-2071 (as larva), emerged on 13–27-IV-2017; 1♀, Tashiro, Kinko, Kimotsuki, Kagoshima Pref., 18-V-2013 collected on thallus of *C.orientalis*; 1♀, Mt. Inao, Kimotsuki, Kagoshima Pref., 28-II-2000 (as larva), emerged on 4-IV-2000.

#### Diagnosis.

 A large dark species (wing length 2.1–2.7 mm) having pruinose dark brown scutum, black 1^st^ flagellomere with yellow arista, yellow maxillary palpus, yellow halteres, and yellow legs. Male epandrium inner-basally with a comb comprising six long fused tubercle-like setae, but lacking an inner-lateral tubercle-like seta. Larva mines the thallus of *Conocephalumorientalis*.

#### Description.

**Figure 44.**
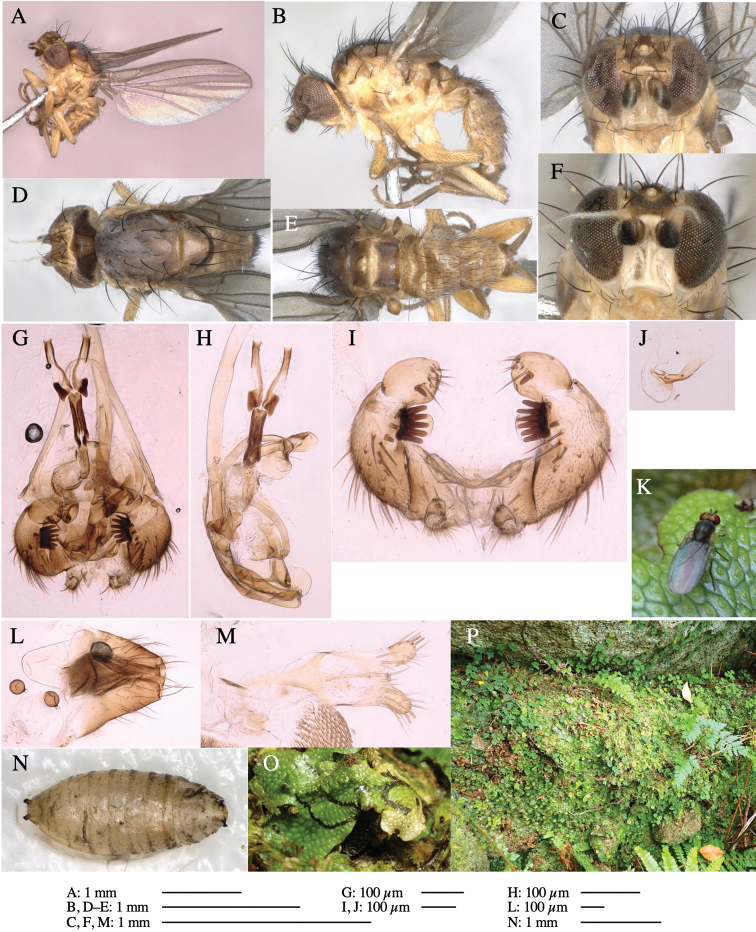
*Phytoliriomyzaugetsu*sp. nov. **A–E **holotype male **A** habitus **B **lateral **C** frontal **D** dorsal **E** posterior **F** paratype female (MK-AG-554), frontal **G–J **male genitalia (**G, H** at type locality **I** Yaku Is. **J** Mikisato) **G** whole genitalia, ventral **H **phallic complex, lateral **I** epandrium, ventral **J** ejaculatory apodeme, lateral **K** a live female fly foraging on *Conocephalumorientalis* thallus at Tategasaki **L, M** female postabdomen **L** oviscape and spermatheca **M** tergite 10 **N** puparia **O** mined thallus of *Conocephalumorientalis*at type locality **P** habitat at Tategasaki.

**Adult male** (Fig. 44A–E).

***Head*:** Head yellow, but frons and ocellar triangle brown, back of head dark brown (Fig. 44C). Antenna porrect, first flagellomere black, pedicel and scape brown (Fig. 44B). Arista subbasal, yellow, pubescent. Clypeus, face, gena, parafacial and postgena yellow (Fig. 44C). Proboscis normal, yellow; palpus yellow, cylindrical. ***Chaetotaxy*:** Front orbitals three pairs; one ori directed inward; two ors directed upward (Fig. 44B). Orbital setulae minute and erect, in a single row.

***Thorax*:** Thorax pruinose. Scutum and scutellum entirely dark brown (Fig. 44D). Subscutellum yellow except brown posterior margin. Mediotergite, katatergite and anatergite brown (Fig. 44E). Pleuron yellow with large brown patches on notopleuron, anepisternum, katepisternum and meron (Fig. 44B). Haltere yellow. Calypter margin and hairs gray. Leg segments yellow; tibia and tarsus darker (Fig. 44A). ***Chaetotaxy*:** Scutum with 1+3 dorsocentrals, shortened anteriorly (Fig. 44B). Acrostichal setulae 10–12 pairs in two irregular rows. ***Wing***: Wing length 2.2 mm, costa reaching M_1_ (Fig. 44A). Length of ultimate section of vein M_4_ divided by penultimate section 1.3.

***Abdomen*:** Abdomen dorsally subshiny yellowish gray; epandrium dark brown (Fig. 44E). ***Genitalia*:** (Fig. 44G–I) Epandrium rounded apically; inner-anterior margin with a row of several (2–4) short tubercle-like setae; inner-anterior surface with a comb comprising six fused long tubercle-like setae (Fig. 44I). Surstylus rounded, directed inwards, setose apically, with one tubercle-like seta on posterior margin (Fig. 44I). Cercus narrow, setose. Subepandrial sclerite V-shaped in a posterior view, with one seta on each dorsal lobe (Fig. 44I). Hypandrium slightly sclerotized along outer margin (Fig. 44G). Postgonite bare, goose barnacle-shaped, rounded apically (Fig. 44H). Phallophorus with deep incision below, articulated with phallapodeme, fused to epiphallus (Fig. 44G). Basiphallus with broad pale plate on left side and lightly sclerotized anterodorsal margin (Fig. 44G). Hypophallus hood-like, membranous; lateral margins lightly sclerotized; covered with microtrichia ventrally; medially with a pair of fused narrow sclerites incurved ventrally; a dark tubule protruding ventrally from subdistal center of the membrane (Fig. 44G, H). Mesophallus dark, cylindrical, widest subbasally, as long as distiphallus (Fig. 44H). Distiphallus comprising one pair of stout tubules basally parallel to each other; basal half composed of ventral dark subrectangular sclerite and weaker medial region; distal half cylindrical, dorsally pigmented with flared apex; with truncated, unpigmented apex (Fig. 44H). Ejaculatory apodeme pale brown, with fan-shaped blade and broad stalk; base wide to one side; sperm pump clear (Fig. 44J).

**Female** (Fig. 44F). Similar to male, but larger, brown patches on pleuron larger and thicker. Wing length 2.3 mm. ***Postabdomen*:** (Fig. 44L, M) Oviscape dark brown, setigerous (Fig. 44L). Tergite 10 cruciform, laterally uniting narrow pleural sclerites (Fig. 44M). Each cercus with two stout, apical, trichoid sensilla, which are ½ length of cercus (Fig. 44M). Spermathecae semi-orbicular, with truncate proximal ends (Fig. 44L).

#### Variation.

The number of tubercle-like setae in a comb of the male epandrium varies from 6 to 8 within the same population and among localities.

**Immatures.** (Fig. 44N) Puparium internal, slender, and pale brown.

#### Etymology.

 The specific name *ugetsu* is a Japanese word meaning rainy moon, and refers to dark scutum without a yellow mark.

#### Japanese name.

Ugetsu-jagoke-hamoguribae.

#### Host plant.

*Conocephalumorientalis* (Conocephalaceae) growing on mesic soils in warm-temperate broadleaf evergreen forests.

#### Mine.

(Fig. 44O) Larvae construct linear mines in the thallus in early instars, later entering the midrib, and pupate in the mines.

#### Biological notes.

 The habitats of this species are stream banks and mesic slopes in warm temperate evergreen forests dominated by *Castanopsissieboldii* and *Quercusglauca* (Fig. 44P). Our rearing records suggest that this species is univoltine, and that adults emerge from overwintered pupae in spring. The female’s oviposition behavior on *C.orientalis* thalli was observed on 23 April 2021 in a *Castanopsis* forest at Tategasaki, Wakayama Pref. (Fig. 44K).

#### Distribution.

**Figure 45.**
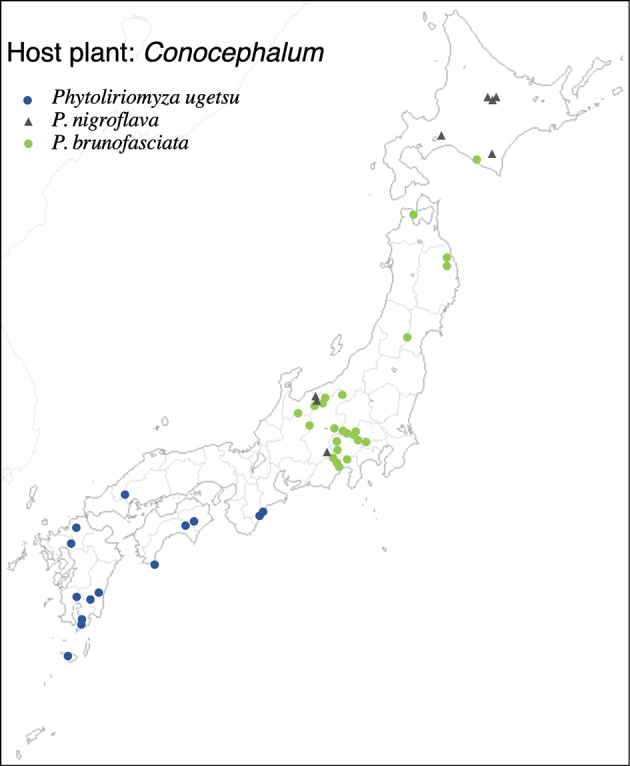
Locality records of three *Phytoliriomyza* species associated with *Conocephalum* spp.: *P.caliginosa*, *P.ugetsu*, *P.nigroflava* and *P.brunofasciata*.

Japan: Honshu, Shikoku, Kyushu (Fig. 45).

#### Remarks.

 This species is unique in the wholly dark brown color of both scutum and scutellum, and easily distinguished from all other *Phytoliriomyza* species.

### ﻿24.﻿ Phytoliriomyza nigroflava

Taxon classificationAnimaliaDipteraAgromyzidae

Kato sp. nov.

2AA427AB-452E-5FE5-BEEA-B5696048C57E

https://zoobank.org/53FC15E5-7A32-4A81-B79D-70C9A564BEF3

Fig. 46 , 47

#### Material examined.

***Holotype*:** Japan: 1♂ (MK-AG-a300), Yuni-ishikari-gawa, Soun-kyo, Kamikawa, Hokkaido (43.640°N,143.048°E, 820 m asl), 31-V-2021 (as larva), emerged on 3-VII-2021, NSMT-I-Dip 32031. ***Paratypes*:** Japan: 1♂1♀ (MK-AG-a467, a22), Yuni-ishikari-gawa, Soun-kyo, Kamikawa, Hokkaido, 1-VI-2020 (as larva), emerged on 8–12-VII-2020, NSMT-I-Dip 32032, 32033; 2♂ (MK-AG- a301, a468), Shirabiso-toge, Kamimura, Iida, Nagano Pref., 17-IV-2021 (as larva), emerged on 22-V-2021, NSMT-I-Dip 32034, 32035; 1♂1♀ (MK-AG-a469, a302), Sarukura, Hakuba, Nagano Pref., 11-V-2021 (as larva), emerged on 21–24-VI-2021, NSMT-I-Dip 32036, 32037.

#### Other material.

Japan: 7♂12♀, Soun-kyo, Kamikawa, Hokkaido, 31-V-2021 (as larva), emerged on 11–22-VII-2021; 2♂4♀, Aizankei, Kamikawa, Hokkaido, 4-X-2011 (as larva), emerged on 26-V–2-VI-2011; 2♂6♀, Yuni-ishikari-gawa, Soun-kyo, Kamikawa, Hokkaido, 1-VI-2020 (as larva), emerged on 5–14-VII-2020; 10♂4♀, Nozuka-toge, Urakawa, Hokkaido, 30-IV-2011 (as larva), emerged on 5–18-VI-2021; 1♂2♀, Mt. Tengu, Jozan-kei, Minami-ku, Sapporo, Hokkaido, 2-V-2021 (as larva), emerged on 7–10-VI-2021; 2♂1♀, Jozan-kei, Minami-ku, Sapporo, Hokkaido, 2-V-2021 (as larva), emerged on 7–19-VI-2021; 1♂, Horoman-kyo, Samani, Hokkaido, 30-IV-2021 (as larva), emerged on 19-VI-2021.

#### Diagnosis.

 A medium-sized yellow species (wing length 2.2–2.3 mm) uniquely having sexual dimorphism in color of the 1^st^ flagellomere: male yellow, female black. The adult has a pruinose yellow scutum with a medial and two pairs of black stripes, yellow maxillary palpus, yellow halteres, and yellow legs. Male epandrium inner-laterally with a long tubercle-like seta, and inner-basally with a comb comprising 6–8 long fused tubercle-like setae. Larva mines the thallus of *Conocephalumsalebrosum*.

#### Description.

**Figure 46.**
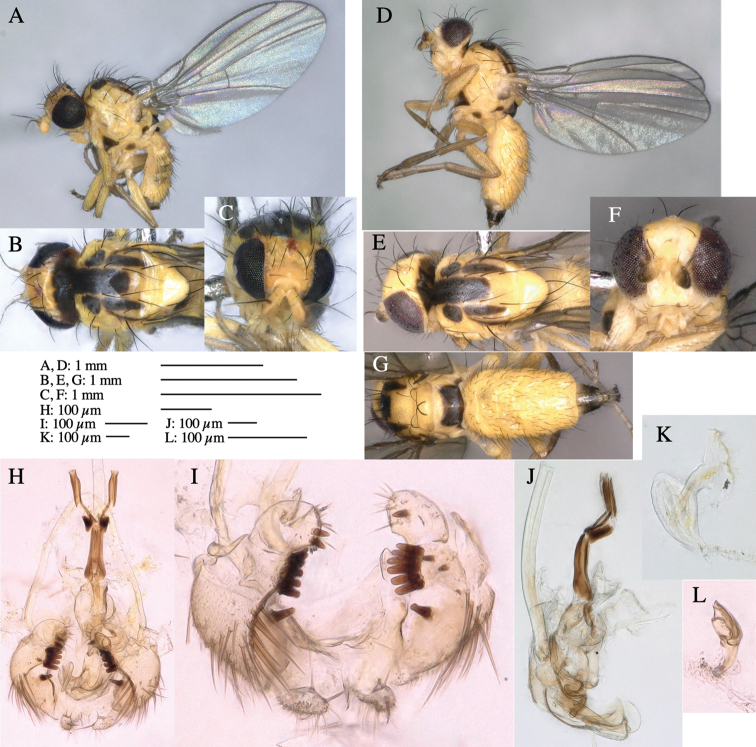
*Phytoliriomyzanigroflava*sp. nov. **A–C **holotype male **A** habitus **B** dorsal **C** frontal **D–G** paratype female (MK-AG-a22) **D** habitus **E** dorsal **F** frontal **G** posterior **H–K** male genitalia **H** whole genitalia, ventral **I** epandrium, ventral **J** phallic complex, lateral **K** ejaculatory apodeme, lateral B postgonite.

**Adult male** (Fig. 46A–C).

***Head*:** Head yellow, with ocellar tubercle pale brown and back of head dark brown excluding margins (Fig. 46C). Antenna porrect, first flagellomere yellow, pedicel and scape brown (Fig. 46A). Arista subbasal, black, pubescent. Clypeus, face, gena, parafacial and postgena yellow (Fig. 46C). Proboscis normal, yellow; palpus yellow, cylindrical (Fig. 46C). ***Chaetotaxy*:** Front orbitals three pairs; one ori directed inward; two ors directed upward (Fig. 46B). Orbital setulae minute and erect, in a single row.

***Thorax*:** Thorax subshiny. Scutum yellow with medial black stripe on anterior 2/3, with a pair of narrow black supra-alar stripes and a pair of wider black intra-alar stripes, which adjoin a pair of lateral presutural black ovoid spots (Fig. 46B). Scutellum and subscutellum yellow. Mediotergite brown, katatergite yellow, and anatergite yellow with venter brown (Fig. 46A). Pleuron yellow with postpronotal lobe faintly brown anteriorly, venter of katepisternum and meron brown (Fig. 46A). Haltere yellow. Calypter margin and hairs gray. Leg segments entirely yellow; tibia and tarsus darker (Fig. 46A). ***Chaetotaxy*:** Scutum with 1+3 dorsocentrals, shortened anteriorly (Fig. 46B). Acrostichal setulae 8–10 pairs in two irregular rows. ***Wing*:** Wing length 2.2 mm, costa reaching M_1_ (Fig. 46A). Length of ultimate section of vein M_4_ divided by penultimate section 1.1–1.2.

***Abdomen*:** Abdomen dorsally subshiny yellow; epandrium brown (Fig. 46A). ***Genitalia*:** (Fig. 46H–L) Epandrium rounded apically; inner-lateral surface with a long tubercle-like seta; inner-anterior surface with a comb comprising 6–8 fused long tubercle-like setae and an irregular row of several (2–4) small tubercle-like setae immediately outward from the comb (Fig. 46I). Surstylus rounded, directed inwards, setose apically, with one (rarely 2) long tubercle-like seta on posterior margin (Fig. 46I). Cercus narrow, setose. Subepandrial sclerite V-shaped in posterior view; dorsal lobe with one seta (Fig. 46I). Hypandrium slightly sclerotized along outer margin (Fig. 46H). Postgonite bare, goose barnacle-shaped, pointed and cleft apically (Fig. 46L). Phallophorus with deep incision below, articulated with phallapodeme, fused to epiphallus (Fig. 46H). Basiphallus with pale broad lateral plate on left side and lightly sclerotized dorsal margin (Fig. 46H). Hypophallus hood-shaped, membranous; lateral margins lightly sclerotized; medially with a pair of dark fused sclerites incurved ventrally, which have small lateral transparent wings (Fig. 46H, I). Mesophallus dark, cylindrical, widest basally, as long as distiphallus (Fig. 46I). Distiphallus comprising one pair of stout tubules; basal half composed of ventral dark subtriangular sclerite and weaker medial region; distal half cylindrical, dorsally pigmented, constricted subdistally; with truncated, unpigmented apex (Fig. 46I). Ejaculatory apodeme pale brown, with hammerhead-shaped blade and broad stalk; base wide to one side; sperm pump clear (Fig. 46K).

**Figure 47.**
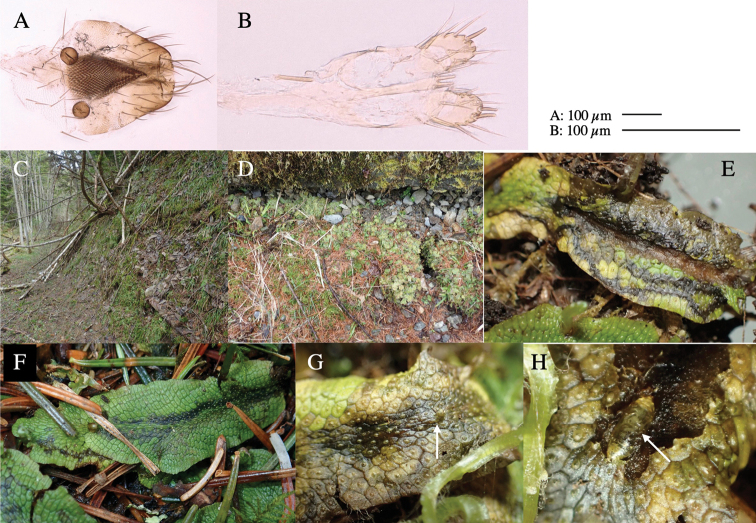
Female morphology and larval ecology of *Phytoliriomyzanigroflava*sp. nov. **A, B **female postabdomen **A** oviscape and spermatheca **B** tergite 10 **C, D** habitat (**C** type locality **D** at Shirabiso-toge) **E–H** mined thalli of *Conocephalumsalebrosum*at type locality. Arrows in **G** and **H** shows internal puparia.

**Female** (Fig. 46D–G). Similar to male, but larger, first flagellomere black with yellowish base; pedicel and scape yellow (Fig. 46F). Wing length 2.32 mm. ***Postabdomen*:** (Fig. 47A, B) Oviscape dark brown, setigerous (Fig. 47A). Tergite 10 trifurcate, laterally uniting narrow pleural sclerites (Fig. 47B). Each cercus with two stout, apical, trichoid sensilla, 1/3 length of cercus (Fig. 47B). Spermathecae semi-orbicular, with truncate proximal ends (Fig. 47A).

#### Variation.

The color of the lateral stripes on the scutum varies among populations, with specimens in the southern population having darker stripes. The number of tubercle-like setae in a comb in male genitalia varies from 5 to 7 among localities.

#### Etymology.

 The specific name (*nigra* = black, *flava* = yellow) refers to heterosexually different colors of flagellomere: precisely, the male and female of this species have a yellow and a black flagellomere, respectively.

#### Japanese name.

Murasame-jagoke-hamoguribae.

#### Host plants.

*Conocephalumsalebrosum* and *C.purpureorubrum* (Conocephalaceae).

#### Mine.

Larvae construct linear mines in the thallus in early instars, later entering the midrib, and pupate in the mines (Fig. 47E–H).

#### Biological notes.

The habitats of this species are mesic slopes in subalpine coniferous forests dominated by *Abies* spp., *Picea* spp. And *Betula* spp. (Fig. 47C, D). Our rearing records suggest that this species is univoltine, and that adults emerge from overwintered pupae in spring.

#### Distribution.

Japan: Hokkaido, Honshu (Fig. 45). The distribution is restricted in cool-temperate subalpine forests in high altitudes.

#### Remarks.

This species is unique in that male and female respectively has yellow and black 1^st^ flagellomere of antenna; intersexual color dimorphism in 1^st^ flagellomere was observed only in this species among the studied species. This species resembles *P.brunofasciata* and *P.pallidofasciata* in having two pairs of dark lateral stripes on scutum and similar genitalia; it is distinguished from them by the color of the two pairs of dark lateral stripes (black in *P.nigroflava*; brown in *P.brunofasciata*; pale brown in *P.pallidofasciata*). It also resembles *P.bifasciata* in having black stripes on scutum; it is distinguished from the latter by the morphology of surstylus of male genitalia (rounded in *P.nigroflava*; elongated in *P.bifasciata*) and the number of the black stripes (two pairs in *P.nigroflava*; one pair in *P.bifasciata*).

### ﻿25.﻿ Phytoliriomyza brunofasciata

Taxon classificationAnimaliaDipteraAgromyzidae

Kato sp. nov.

6A411C5F-5B58-5EF5-862C-67C2F1B4E11C

https://zoobank.org/524B0462-AA14-4B7C-83A2-B048C7CFBCFA

Fig. 48

#### Material examined.

***Holotype*:** Japan: 1♂ (MK-AG-a380), Yashajin-toge, Minami-arupusu, Yamanashi Pref. (35.6327°N, 138.3519°E, 1110 m asl), 25-III-2021 (as larva on *C.salebrosum*), emerged on 6-V-2021, NSMT-I-Dip 32038. ***Paratypes*:** Japan: 1♀ (MK-AG-a429), same data as holotype, emerged on 5-V-2021, NSMT-I-Dip 32039; 1♂ (MK-AG-a405), Yashajin-toge, Minami-arupusu, Yamanashi Pref., 10-XII-2016 (as larva on *C.salebrosum*), emerged on 3-V-2017, NSMT-I-Dip 32040; 1♀ (MK-AG-498), Akka, Iwaizumi, Iwate Pref., 8-V-2010 (as larva on *C.salebrosum*), emerged on 8-VI-2010, NSMT-I-Dip 32041; 1♂1♀ (MK-AG-a344, a345), Nippara, Okutama, Tokyo Pref., 27-III-2021 (as larva on *C.salebrosum*), emerged on 8-V-2021, NSMT-I-Dip 32042, 32043; 1♂ (MK-AG-a298), Nishiyama-onsen, Hayakawa, Yamanashi Pref., 18-III-2017 (as larva on *C.salebrosum*), emerged on 4-V-2017, NSMT-I-Dip 32044.

#### Other material.

 Japan: On *Conocephalumsalebrosum*: 3♂1♀, Hashigami, Yamane, Kuji, Iwate Pref., 5-V-2012 (as larva), emerged on 5-29–5-VI-2012; 2♂6♀, Mt. Futago, Ogano, Chichibu-gun, Saitama Pref., 28-XI-2014 (as larva), emerged on 19-IV–10-VI-2015; 4♂12♀, Nippara, Okutama, Tokyo Pref., 15-III-2016 (as larva), emerged on 5–13-V-2016; 2♂1♀, Akiyama-go, Sakae-mura, Nagano Pref., 3-V-2015 (as larva), emerged on 26-V–14-VII-2020; 8♂18♀, Yashajin-toge, Minami-arupusu, Yamanashi Pref., 15-V-2018 (as larva), emerged on 1–4-VI-2018; 2♂5♀, Sengataki, Uminokuchi, Minami-maki, Nagano Pref., 28-IV-2014 (as larva), emerged on 3-V–10-VI-2014; 4♂6♀, Azusayama, Kawakami-mura, Nagano Pref., 28-IV-2014 (as larva), emerged on 25–2-V-2014.

On *Conocephalumorientalis*: 1♂, Tairadate, Sotogahama, Higashitsugaru, Aomori Pref., 26-V-2012 (as larva), emerged on 1–15-VI-2012; 1♀, Yusen-kyo, Yamadera, Yamagata Pref., 15-IV-2014 (as larva), emerged on 3-V–3-VI-2014.

On *Conocephalumpurpureorubrum*: 2♂1♀, Akka, Iwaizumi, Iwate Pref., 5-V-2012 (as larva), emerged on 2–6-VI-2012; 20♂22♀, Mitsumine-jinja, Chichibu, Saitama Pref., 26-III-2021 (as larva), emerged on 30-IV–2-V-2021; 1♀, Sarukura, Hakuba, Nagano Pref., 9-VI-2013 (as larva), emerged on 22-VII-2013; 1♂, Mitsumine-jinja, Chichibu, Saitama Pref., 13-V-2011 (as larva), emerged on 12-VI-2011.

#### Diagnosis.

 A medium-sized yellow species (wing length 1.9–2.2 mm) having pruinose yellow scutum with a medial and two pairs of gray stripes, a black 1^st^ flagellomere, yellow maxillary palpus, yellow halteres, and yellow legs. Male epandrium inner-laterally with a long tubercle-like seta, and inner-basally with a comb comprising 5–7 long fused tubercle-like setae. Larva mines the thallus of *Conocephalumsalebrosum*,*C.orientalis* and *C.purpureorubrum*.

#### Description.

**Figure 48.**
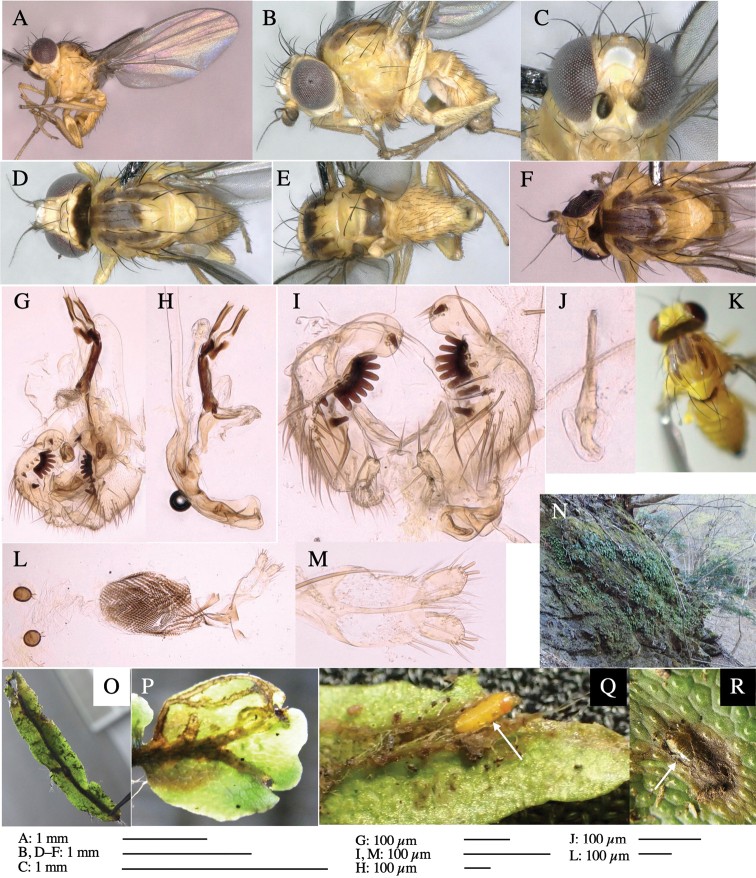
*Phytoliriomyzabrunofasciata*sp. nov. **A–E **holotype male **A** habitus** B** dorsal **C** frontal **D** dorsal **E** posterior **F** paratype female (MK-AG-a42), dorsal **G–J** male genitalia **G** whole genitalia, ventral **H** phallic complex, ventral** I** epandrium, ventral **J** ejaculatory apodeme, dorsal **L, M** female postabdomen **L** oviscape and spermatheca **M** tergite 10 **N** habitat at Nishiyama-onsen **O–R** mined thalli of *Conocephalumsalebrosum*. Arrows in **Q** and **R** indicate puparia.

**Adult male** (Fig. 48A–E).

***Head*:** Head yellow, with back of head dark brown excluding margins (Fig. 48C). Antenna porrect, first flagellomere black, pedicel and scape yellow (Fig. 48B). Arista subbasal, black, pubescent. Clypeus, face, gena, parafacial and postgena yellow. Proboscis normal, yellow; palpus yellow, cylindrical (Fig. 48C). ***Chaetotaxy*:** Front orbitals three pairs; one ori directed inward; two ors directed upward (Fig. 48D). Orbital setulae minute and erect, in a single row.

***Thorax*:** Thorax pruinose. Scutum yellow with a medial brown stripe on anterior 2/3, with a pair of narrow brown supra-alar stripes and a pair of wider brown intra-alar stripes, which adjoin a pair of lateral presutural brown ovoid spots (Fig. 48D). Scutellum and subscutellum yellow. Mediotergite and anatergite brown, katatergite yellow with venter brown (Fig. 48E). Pleuron yellow with venter of katepisternum and meron brown (Fig. 48B). Haltere yellow. Calypter margin and hairs gray. Leg segments entirely yellow; tibia and tarsus darker (Fig. 48A). ***Chaetotaxy*:** Scutum with 1+3 dorsocentrals, shortened anteriorly. Acrostichal setulae seven or eight pairs in two rows. ***Wing***: Wing length 2.2 mm, costa reaching M_1_. Length of ultimate section of vein M_4_ divided by penultimate section 1.1–1.2.

***Abdomen*:** Abdomen dorsally subshiny yellow; epandrium brown. ***Genitalia*:** (Fig. 48G–J) Epandrium rounded apically; inner-lateral surface with a long tubercle-like seta; inner-anterior surface with a comb comprising six or seven fused (rarely unfused in part) long tubercle-like setae (rarely unfused in part) and an irregular row of several (2–3) short tubercle-like setae immediately outward from the comb (Fig. 48I). Surstylus rounded, directed inwards, setose apically, with one long tubercle-like seta on posterior margin (Fig. 48I). Cercus narrow, setose. Subepandrial sclerite V-shaped in a posterior view; a pair of dorsal lobes, each with one seta. Hypandrium slightly sclerotized along outer margin (Fig. 48I). Postgonite bare, goose barnacle-shaped, rounded apically (Fig. 48G). Phallophorus with deep incision below, articulated with phallapodeme, fused to epiphallus (Fig. 48H). Basiphallus with pale broad lateral plate on left side and lightly sclerotized dorsal margin (Fig. 48G). Hypophallus hood-shaped, membranous; covered with microtrichia ventrally; medially with a pair of dark fused, ventrally incurved, narrow sclerites (Fig. 48G, H). Paraphallus membranous, rounded or like 4-winged; paraphalli diverging, angled anteroventrally, jointed basally (Fig. 48H). Mesophallus dark, cylindrical, as long as distiphallus. Distiphallus comprising one pair of stout tubules basally parallel to each other; basal half composed of ventral dark subtriangular sclerite and weaker medial region; distal half cylindrical, dorsally pigmented, with truncated unpigmented apex (Fig. 48H). Ejaculatory apodeme pale brown, with fan-shaped blade and broad stalk; base wide to one side; sperm pump clear (Fig. 48J).

**Female** (Fig. 48F). Similar to male, but larger, first flagellomere black, rarely base yellowish; pedicel and scape yellow. Wing length 2.3 mm. ***Postabdomen*:** (Fig. 48L, M) Oviscape dark brown, setigerous (Fig. 48L). Tergite 10 trifurcate, laterally uniting narrow pleural sclerites (Fig. 48M). Each cercus with two stout, apical, trichoid sensilla, 1/3 length of cercus (Fig. 48M). Spermathecae orbicular (Fig. 48A).

**Immatures.** (Fig. 48Q) Puparium internal, slender, and brown.

#### Etymology.

 The specific name (*brunus* = brown, *fascia* = stripe) refers to the brown stripes on the scutum.

#### Japanese name.

Harusame-jagoke-hamoguribae.

#### Host plants.

*Conocephalumsalebrosum*,*C.orientalis* and *C.purpureorubrum* (Conocephalaceae) growing on mesic soils in cool-temperate broadleaf deciduous forests.

#### Mine.

Larvae construct linear mines in the thallus in early instars, later entering the midrib, and pupate in the mines (Fig. 48O–R).

#### Biological notes.

 The habitats of this species are stream banks and mesic slopes in cool temperate deciduous forests dominated by *Faguscrenata*,*Cercidiphyllumjaponicum*, and *Quercuscrispula* (Fig. 48N). Our rearing records suggest that this species is univoltine, and that adults emerge from overwintered pupae in spring.

#### Distribution.

Japan: Hokkaido, Honshu (Fig. 45). The distribution is restricted to broadleaf deciduous forests in the cool temperate zone at high altitudes.

#### Remarks.

 This species resembles *P.nigroflava*,*P.pallidofasciata*, and *P.bifasciata* in having two pairs of dark lateral stripes on the scutum; it is distinguished from them by the color of the stripes (brown in *P.brunofasciata*; black in *P.nigroflava*; pale brown in *P.pallidofasciata*; inner pairs black and outer pairs pale brown in *P.bifasciata*).

### ﻿26.﻿ Phytoliriomyza pallidofasciata

Taxon classificationAnimaliaDipteraAgromyzidae

Kato sp. nov.

D6B14157-6CCE-5DCB-81FD-78514B4D73F6

https://zoobank.org/C8B683ED-E6C7-401F-ADF4-73F11F414EF8

Fig. 49

#### Material examined.

***Holotype*:** Japan: 1♂ (MK-AG-a519), Tazukawa-keikoku, Katsuura, Tokushima Pref. (33.8907°N,134.4580°E, 310 m asl), 30-III-2021 (as larva), emerged on 27-IV-2021, NSMT-I-Dip 32045. ***Paratypes*:** Japan: 1♂2♀ (MK-AG-a538, a520, a537), same data as holotype, emerged on 27-IV–1-V-2016, NSMT-I-Dip 32046–32048; 1♀ (MK-AG-676), Asahi-daki, Shuzenji, Izu, Shizuoka Pref., 7-III-2012 (as larva), emerged on 20-IV-2012, NSMT-I-Dip 32049; 1♀ (MK-AG-a240) Mt. Ichifusa, Mizukami, Kuma, Kumamoto Pref., 14-XII-2012 (as larva), emerged on 22-III-2013, NSMT-I-Dip 32050.

#### Other material.

Japan: 3♂1♀, Momiki, Izumi, Yatsushiro, Kumamoto Pref., 23-III-2015 (as larva), emerged on ?-VI-2015; 2♂, Yoro-keikoku, Otaki, Isumi, Chiba Pref., 17-III-2016 (as larva), emerged on 18–20-IV-2016; 1♂2♀, Amagi-toge, Izu, Shizuoka Pref., 19-IV-2012 (as larva), emerged on 8-V–3-VI-2021; 2♂2♀, Kuki, Owase, Mie Pref., 29-III-2019 (as larva), emerged on 9–30-IV-2019; 1♀, Takinohai, Kozagawa, Wakayama Pref., 13-IV-2014 (as larva), emerged on 19-IV-2014; 2♀, Wabuka, Kushimoto, Wakayama Pref., 4-V-2012 (as larva), emerged on 9-IV-2012; 7♂8♀, Narutaki, Ichiu, Tsurugi, Tokushima Pref., 31-III-2021 (as larva), emerged on 28-IV–20-V-2021; 1♂2♀, Yasui-keikoku, Niyodogawa, Agawa, Kochi Pref., 27-II-2011 (as larva), emerged on 25-IV-2011; 3♀, Mt. Kosho, Asakura, Fukuoka Pref., 11-IV-2010 (as larva), emerged on 1–13-V-2016; 1♂, Amagi-toge, Izu, Kaeda-keikoku, Kagamisu, Miyazaki, Miyazaki Pref. Pref., 11-IV-2021 (as larva), emerged on 19-IV-2021.

#### Diagnosis.

 A medium-sized yellow species (wing length 1.9–2.0 mm) having pruinose yellow scutum with two pairs of pale brown stripes, a black 1^st^ flagellomere, yellow maxillary palpus, yellow halteres, and yellow legs. Male epandrium inner-laterally with an extended, apically flattened tubercle-like seta, and inner-basally with a comb comprising 3–5 long fused tubercle-like setae. Larva mines the thallus of *Conocephalumorientalis*.

#### Description.

**Figure 49.**
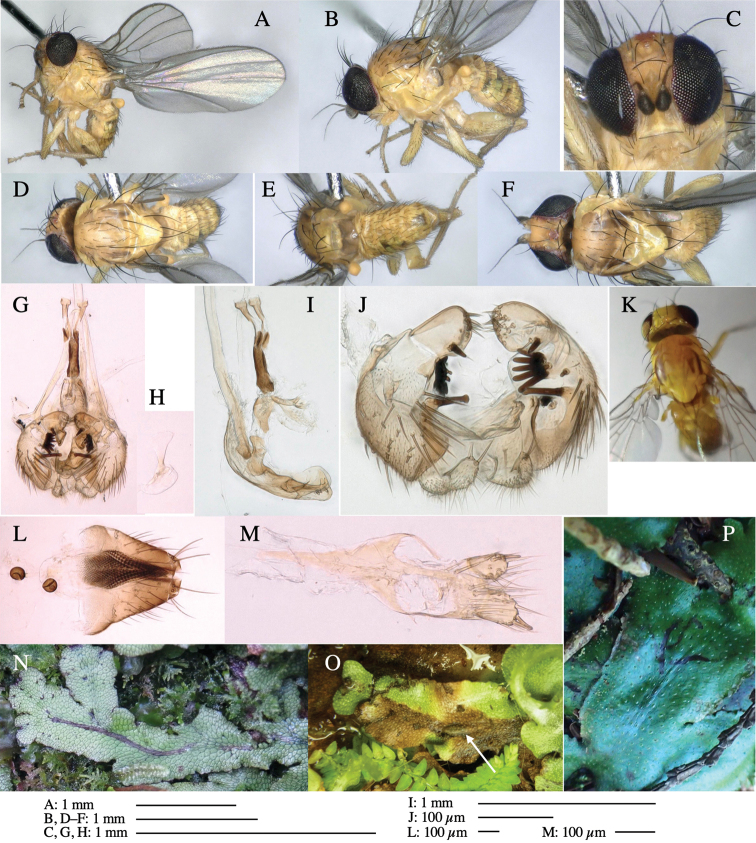
*Phytoliriomyzapallidofasciata*sp. nov. **A–E **holotype male **A** habitus **B** dorsal **C** frontal **D** dorsal **E** posterior **F** paratype female (MK-AG-a520), dorsal **G–J** male genitalia **G** whole genitalia **H** ejaculatory apodeme, lateral **I** phallic complex, lateral **J** epandrium, ventral **K** live fly **L, M** female postabdomen **L** oviscape and spermatheca **M** tergite 10 **N–P** mined thalli of *Conocephalumorientalis*. An arrow in **O** indicates an internal puparium.

**Adult male** (Fig. 49A–E).

***Head*:** Head yellow, with back of head dark brown excluding margins (Fig. 49C). Antenna porrect, first flagellomere black, pedicel and scape yellow (Fig. 49B). Arista subbasal, black, pubescent. Clypeus, face, gena, parafacial and postgena yellow. Proboscis normal, yellow; palpus yellow, cylindrical (Fig. 49C). ***Chaetotaxy*:** Front orbitals three pairs; one ori directed inward; two ors directed upward (Fig. 49B). Orbital setulae minute and erect, in a single row.

***Thorax*:** Thorax pruinose. Scutum yellow with a medial brownish yellow stripe on anterior 2/3, with a pair of narrow pale brown supra-alar stripes and a pair of wider pale brown intra-alar stripes, which adjoin a pair of lateral presutural pale brown ovoid spots (Fig. 49B). Scutellum, subscutellum, mediotergite, anatergite and katatergite yellow (Fig. 49E). Pleuron entirely yellow (Fig. 49B). Haltere yellow. Calypter margin and hairs gray. Leg segments entirely yellow; tibia and tarsus darker. ***Chaetotaxy*:** Scutum with 1+3 dorsocentrals, shortened anteriorly (Fig. 49D). Acrostichal setulae eight or nine pairs in two irregular rows. ***Wing*:** Wing length 2.0 mm, costa reaching M_1_ (Fig. 49A). Length of ultimate section of vein M_4_ divided by penultimate section 1.1–1.3.

***Abdomen*:** Abdomen dorsally subshiny yellow; epandrium brown (Fig. 49E). ***Genitalia*:** (Fig. 49) Epandrium rounded apically;inner-lateral surface with an elongated tubercle-like seta, whose tip papillate; inner-anterior surface with a comb comprising six or seven fused long tubercle-like setae (rarely unfused in part) and a row of 2–5 short tubercle-like setae immediately outward from the comb (Fig. 49J). Surstylus rounded, directed inwards, setose apically, with one long tubercle-like seta on posterior margin (Fig. 49J). Cercus narrow, setose. Subepandrial sclerite V-shaped in a posterior view; dorsal lobe plate-like, with a pair of setae basally (Fig. 49J). Hypandrium slightly sclerotized along outer margin (Fig. 49G). Postgonite bare and goose barnacle-shaped (Fig. 49G). Phallophorus with deep incision below, articulated with phallapodeme, fused to epiphallus (Fig. 49G, I). Basiphallus with broad plate on left side and lightly sclerotized anterodorsal margin (Fig. 49G). Hypophallus membranous, covered with microtrichia ventrally; with margins lightly sclerotized; medially with a pair of dark fused, ventrally incurved narrow sclerites; a small tubule protruding ventrally from subdistal center of the membrane (Fig. 49I). Paraphallus absent. Mesophallus dark, cylindrical, as long as distiphallus (Fig. 49I). Distiphallus comprising one pair of stout tubules basally parallel to each other; basal half composed of ventral dark subtriangular sclerite and weaker medial region; distal half cylindrical, pigmented, with inflated, truncated apex (Fig. 49I). Ejaculatory apodeme pale brown, with fan-shaped blade and broad stalk; base wide to one side; sperm pump clear (Fig. 49H).

**Female** (Fig. 49F). Similar to male, mediotergite sometimes brownish. Wing length 1.9 mm. ***Postabdomen*:** (Fig. 49L, M) Oviscape dark brown, setigerous (Fig. 49L). Tergite 10 trifurcate, laterally uniting narrow pleural sclerites (Fig. 49M). Each cercus with two stout, apical, trichoid sensilla, 1/3 length of cercus (Fig. 49M). Spermathecae semi-orbicular, with truncate proximal ends (Fig. 49L).

#### Variation.

The color of the lateral stripes on the scutum varied among populations, but a geographical cline was not observed. The number of tubercle-like setae in a comb of the male epandrium varied from 5 to 6 among localities.

#### Etymology.

 The specific name (*pallidus* = pale, *fascia* = stripe) refers to the two pairs of pale brown stripes on the scutum.

#### Japanese name.

Kirisame-jagoke-hamoguribae.

#### Host plant.

*Conocephalumorientalis* (Conocephalaceae).

#### Mine.

Larvae construct linear mines in the thallus in early instars, later entering the midrib, and pupate in the mines (Fig. 49N–P).

#### Biological notes.

The habitats of this species are stream banks and mesic slopes in warm temperate evergreen forests dominated by *Castanopsiscuspidata* and *Quercusglauca*. Our rearing records suggest that this species is univoltine, and that adults emerge from overwintered pupae in spring.

#### Distribution.

**Figure 50:**
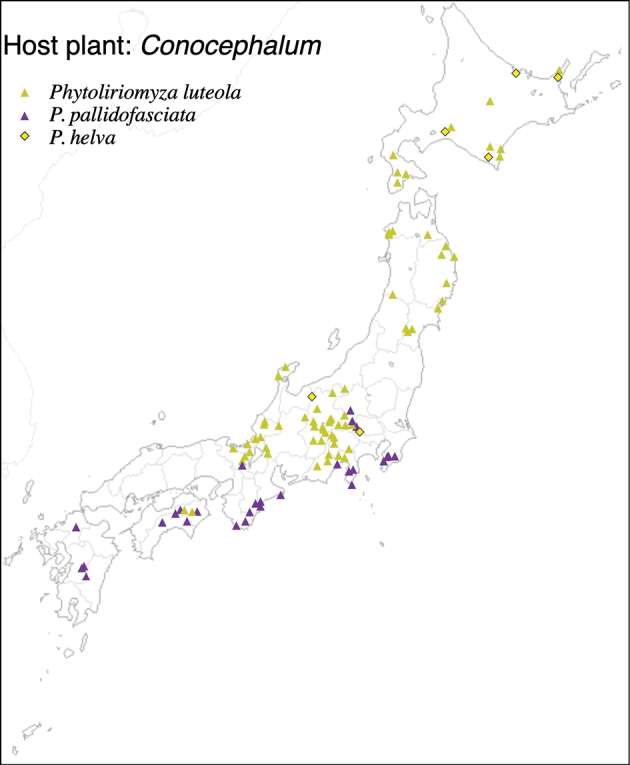
Locality records of three *Phytoliriomyza* species associated with *Conocephalum* spp.: *P.pallidofasciata*, *P.luteola* and *P.helva*.

Japan: Honshu, Shikoku and Kyushu (Fig. 50).

#### Remarks.

This species resembles *P.nigroflava*,*P.brunofasciata*, and *P.bifasciata* in having two pairs of dark lateral stripes on the scutum; it is distinguished from them by the color of the stripes (pale brown in *P.pallidofasciata*; black in *P.nigroflava*; brown in *P.brunofasciata*; inner pairs black and outer pairs pale brown in *P.bifasciata*. This species also resembles *P.luteola* in having wholly yellow body; it is distinguished from the latter by having two pairs of lateral stripes on the scutum (absent in *P.luteola*), and by the number of tubercle-like setae in a comb of the male epandrium (4–5 in *P.pallidofasciata*; 3–4 in *P.luteola*). The locality records of *P.pallidofasciata* are concentrated along southern sea coasts, while those of *P.luteola* are scattered in higher altitudes and in northern areas.

### ﻿27.﻿ Phytoliriomyza luteola

Taxon classificationAnimaliaDipteraAgromyzidae

Kato sp. nov.

5B4B5EE7-E88D-522D-93BA-A57B159DDE3D

https://zoobank.org/CFF5A73E-5132-4E3D-80F6-7AD3BFB6FBDD

Fig. 51

#### Material examined.

***Holotype*:** Japan: 1♂ (MK-AG-a407), Yashajin-toge, Minami-arupusu, Yamanashi Pref. (35.6327°N, 138.3519°E, 1110 m asl), 25-III-2021 (as larva on *C.salebrosum*), emerged on 5-V-2021, NSMT-I-Dip 32051. ***Paratypes*:** Japan: 1♂ (MK-AG-a241), type locality, 15-V-2018 (as larva on *C.salebrosum*), emerged on 31-V-2018, NSMT-I-Dip 32052; 2♀ (MK-AG-a1, 666), type locality, 10-XII-2016 (as larva on *C.salebrosum*), emerged on 3-V-2017, NSMT-I-Dip 32053, 32054; 1♀ (MK-AG-319), Iwaobetsu, Shari, Hokkaido, 1-V-2021 (as larva on *C.salebrosum*), emerged on 11-VI-2021, NSMT-I-Dip 32055; 1♀ (MK-AG-a263), Ashiu, Nantan, Kyoto Pref., 28-IV-2010 (as larva on *C.orientalis*), emerged on 30-V-2010, NSMT-I-Dip 32056.

#### Other material.

 Japan: On *Conocephalumsalebrosum*: 3♂3♀, Iwaobetsu, Shari, Hokkaido, 1-V-2021 (as larva), emerged on 7–15-VI-2019; 1♂1♀, Horoka, Kamishihoro, Hokkaido, 31-V-2021 (as larva), emerged on 24-VI–2-VII-2021; 3♂3♀, Horoman-kyo, Samani, Hokkaido, 30-IV-2021 (as larva), emerged on 22-V–8-VI-2021; 5♂7♀, Mt. Horoiwa, Saroma, Tokoro, Hokkaido, 1-V-2021 (as larva), emerged on 1–8-VI-2021; 5♂7♀, Soun-kyo, Kamikawa, Hokkaido, 1-V-2021 (as larva), emerged on 31-V–26-VI-2021; 4♂5♀, Samani-dam, Samani, Hokkaido, 30-IV-2021 (as larva), emerged on 5–13-VI-2021; 6♂10♀, Narahara, Ueno, Tano, Gunnma Pref., 18-IV-2011 (as larva), emerged on 15–22-V-2021; 3♂3♀, Yashajin-toge, Minami-arupusu, Yamanashi Pref., 10-XII-2016 (as larva), emerged on 24-IV–1-V-2016; 13♂9♀, Sarukura, Hakuba, Nagano Pref., 11-V-2021 (as larva), emerged on 7–19-VI-2021; 4♂4♀, Shirahone-onsen, Matsumoto, Nagano Pref., 14-V-2011 (as larva), emerged on 9–17-VI-2011; 3♂2♀, Kibune, Sakyo-ku, Kyoto Pref., 29-IV-2012 (as larva), emerged on 25–30-V-2012; 1♂4♀, Mt. Toyoguchi, Ooshika, Shimo-ina, Nagano Pref., 29-IV-2012 (as larva), emerged on 30–31-V-2012; 2♂, Azusayama, Kawakami-mura, Nagano Pref., 28-IV-2014 (as larva), emerged on 27-V-2014; 1♂1♀, Abe-toge, Aoi-ku, Shizuoka Pref., 30-IX-2014 (as larva), emerged on 24-IV–1-V-2014;.

On *Conocephalumorientalis*: 1♂1♀, Nakanomata, Hachimori, Yatsumine, Aomori Pref., 6-XI-2014 (as larva), emerged on 30-IV–6-V-2014; 6♂12♀, Yusen-kyo, Yamadera, Yamagata Pref., 15-IV-2012 (as larva), emerged on 7-V–4-VI-2012; 1♂1♀, Saruyama, Monzen, Wajima, Ishikawa Pref., 4-V-2013 (as larva), emerged on 22-V-2013; 3♂7♀, Uchinami, Katsuhara, Oono, Fukui Pref., 13-IV-2011 (as larva), emerged on 13–18-V-2011; 3♂4♀, Suizu, Tsuruga, Fukui Pref., 11-III-2012 (as larva), emerged on 15–20-IV-2012; 2♂3♀, Muramatsu, Iwakura, Sakyo-ku, Kyoto Pref., 5-IV-2017 (as larva), emerged on 8–12-V-2017; 41♂42♀, Tazukawa-keikoku, Katsuura, Tokushima Pref., 30-III-2021 (as larva), emerged on 25–30-IV-2021; 7♂8♀, Narutaki, Ichiu, Tsurugi, Tokushima Pref.2, 31-III-2021 (as larva), emerged on 28-IV–2-V-2021.

On *Conocephalumpurpureorubrum*: 2♂3♀: Tanneso, Rubeshibetsu, Hiroo, Hokkaido, 2-X-2011 (as larva), emerged on 19–21-V-2011; 1♂1♀, Toyoni-gawa, Erimo, Toyoizumi, Hokkaido, 1-VI-2021 (as larva), emerged on 24–26-VI-2021; 4♀, Eniwa-keikoku, Eniwa, Hokkaido, 2-V-2021 (as larva), emerged on 1–6-VI-2021; 1♂1♀, Namari-kawa, Yakumo, Futami, Hokkaido, 2-VI-2021 (as larva), emerged on 18–27-VI-2021; 3♂6♀, Kamiyasse, Kesennuma, Miyagi Pref., 25-III-2016 (as larva), emerged on 30-IV–5-V-2016; 6♂10♀, Narahara, Ueno, Tano, Gunnma Pref., 18-IV-2021 (as larva), emerged on 15–21-V-2021; 2♀, Nakabusa-onsen, Azumino, Nagano Pref., 5-V-2016 (as larva), emerged on 8-VI-2016; 3♂2♀, Kibune, Sakyo-ku, Kyoto Pref., 29-IV-2012 (as larva), emerged on 25–30-V-2012; 2♂3♀, Irisawai, Oshika, Nagano Pref., 26-V–5-VI-2011 (as larva), emerged on 22-V-2013; 1♂, Usuzuka, Fujinomiya, Shizuoka Pref., 25-IV-2011 (as larva), emerged on 22-V-2011; 2♂2♀, Yugashima, Izu, Shizuoka Pref., 7-III-2012 (as larva), emerged on 9–18-IV-2012; 3♂7♀, Uchinami, Katsuhara, Oono, Fukui Pref., 13-IV-2011 (as larva), emerged on 13–18-V-2011.

#### Diagnosis.

 A medium-sized yellow species (wing length 1.9–2.0 mm) having pruinose, entirely yellow scutum and scutellum, a black 1^st^ flagellomere, yellow maxillary palpus, yellow halteres, and yellow legs. Male epandrium inner-laterally with an extended, apically flattened tubercle-like seta, and inner-basally with a comb comprising 3–5 long fused tubercle-like setae. Larva mines the thallus of *Conocephalumsalebrosum*,*C.orientalis*, and *C.purpureorubrum*.

#### Description.

**Figure 51.**
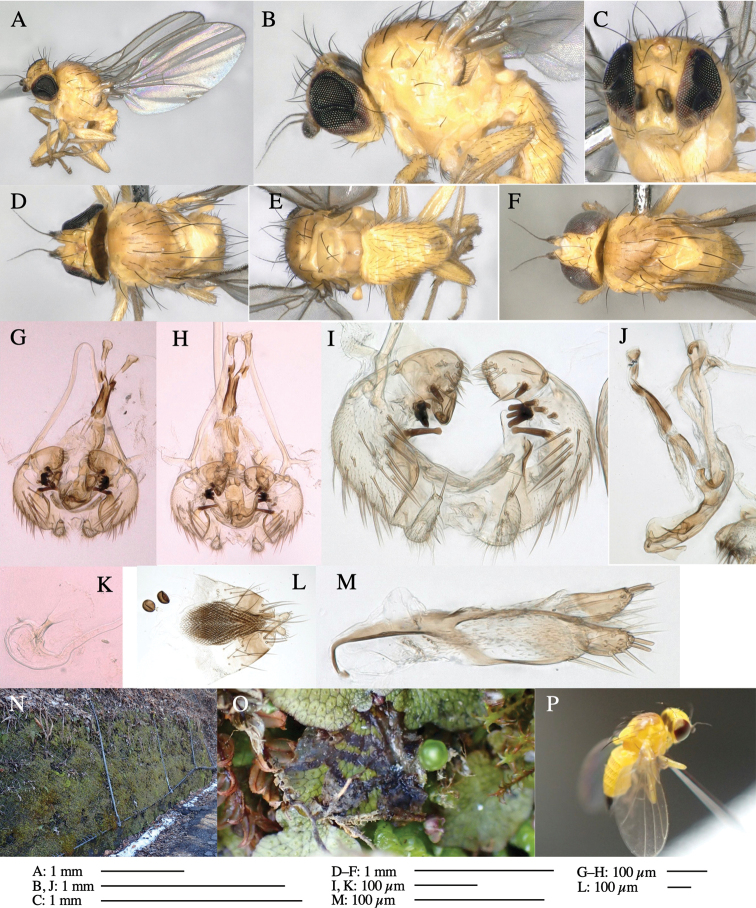
*Phytoliriomyzaluteola*sp. nov. **A–E **holotype male **A** habitus **B** dorsal **C** frontal **D** dorsal **E** posterior **F** paratype female (MK-AG-a1), dorsal **G–K** male genitalia (**G** at type locality **K–M** at Mt. Horoiwa **K** at Sounkyo) **G, H** whole genitalia, ventral **I** epandrium, ventral **J** phallic complex, lateral **K** ejaculatory apodeme, lateral **L, M** female postabdomen **L** oviscape and spermatheca **M** tergite 10 **N** habitat at type locality **O** mined thallus of *Conocephalumsalebrosum***P** live female fly at Eniwa.

**Adult male** (Fig. 51A–E).

***Head*:** Head yellow, with back of head dark brown excluding margins (Fig. 51C). Antenna porrect, first flagellomere black, pedicel and scape yellow (Fig. 51B). Arista subbasal, black, pubescent. Clypeus, face, gena, parafacial and postgena yellow. Proboscis normal, yellow; palpus yellow, cylindrical (Fig. 51C). ***Chaetotaxy*:** Front orbitals three pairs; one ori directed inward; two ors directed upward (Fig. 51B). Orbital setulae minute and erect, in a single row.

***Thorax*:** Thorax pruinose. Scutum yellow with medial pale brownish stripe on anterior 2/3, with a pair of narrow pale brownish supra-alar stripes and a pair of wider, pale brownish intra-alar stripes, which adjoin a pair of lateral presutural pale brownish ovoid spots (Fig. 51D). Scutellum, subscutellum, mediotergite, anatergite and katatergite yellow (Fig. 51D). Pleuron entirely yellow (Fig. 51B). Haltere yellow. Calypter margin and hairs gray. Leg segments entirely yellow; tibia and tarsus darker (Fig. 51A). ***Chaetotaxy*:** Scutum with 1+3 dorsocentrals, shortened anteriorly (Fig. 51D). Acrostichal setulae eight or nine pairs in two irregular rows. ***Wing*:** Wing length 2.0 mm, costa reaching M_1_ (Fig. 51A). Length of ultimate section of vein M_4_ divided by penultimate section 1.1–1.3.

***Abdomen*:** Abdomen dorsally subshiny yellow; epandrium brown (Fig. 51E). ***Genitalia*:** (Fig. 51G–K) Epandrium rounded apically; inner-lateral surface with an elongated tubercle-like seta, whose tip is slightly spread and flattened; inner-anterior surface with a comb comprising three to five fused long tubercle-like setae (rarely unfused in part) and a row of 1–3 short tubercle-like setae immediately outward from the comb (Fig. 51I). Surstylus rounded, directed inwards, setose apically, with one long tubercle-like seta on posterior basal margin (Fig. 51I). Cercus narrow, setose. Subepandrial sclerite V-shaped. Hypandrium slightly sclerotized along outer margin (Fig. 51G). Postgonite bare, goose barnacle-shaped, rounded apically (Fig. 51J). Phallophorus with deep incision below, articulated with phallapodeme, fused to epiphallus (Fig. 51G). Basiphallus with broad plate on left side and with lightly sclerotized anterodorsal margin (Fig. 51G, H). Hypophallus membranous, covered with microtrichia ventrally; with margins lightly sclerotized; medially with a pair of dark fused, dorsally incurved narrow sclerites (Fig. 51J). Paraphallus absent. Mesophallus dark, cylindrical, as long as distiphallus (Fig. 51G). Distiphallus comprising one pair of stout tubules basally parallel to each other; basal half composed of ventral dark subtriangular sclerite and weaker medial region; distal half cylindrical, pigmented; widening toward inflated, truncated apex (Fig. 51J). Ejaculatory apodeme pale, with fan-shaped blade and broad stalk; base widened to one side; sperm pump clear (Fig. 51K).

**Female** (Fig. 51F). Similar to male, but slightly larger and frons wider., mediotergite sometimes brownish. Wing length 1.9 mm. ***Postabdomen*:** (Fig. 51L, M) Oviscape dark brown, setigerous. Tergite 10 trifurcate, laterally uniting narrow pleural sclerites. Each cercus with two stout, apical, trichoid sensilla, 1/3 length of cercus. Spermathecae semi-orbicular, with truncate proximal ends.

#### Variation.

The number of tubercle-like setae in a comb of the male epandrium varied from 3 to 5 among localities, with the specimens from northern populations and at high altitudes having fewer tubercle-like setae.

#### Etymology.

 The specific name (*luteola* = yellow) refers to totally yellow body of the species.

#### Japanese name.

Kiiro-jagoke-hamoguribae.

#### Host plants.

*Conocephalumsalebrosum*, *C.orientalis* and *C.purpureorubrum* (Conocephalaceae).

#### Mine.

Larvae construct linear mines in the thallus in early instars, later entering the midrib, and pupate in the mines (Fig. 51O).

#### Biological notes.

 The habitats of this species are stream banks, mesic slopes and stone wall in cool temperate deciduous forests dominated by *Quercuscrispula*,*Aesculusturbinata*, and *Pterocaryarhoifolia* (Fig. 51N). Our rearing records suggest that this species is univoltine, and that adults emerge from overwintered pupae in spring.

#### Distribution.

Japan: Hokkaido, Honshu, Shikoku, Kyushu (Fig. 50).

#### Remarks.

 This species resembles *P.pallidofasciata and P.helva* in having wholly yellow body; it is distinguished from *P.pallidofasciata* by the absence of two pairs of pale brown lateral stripes, and from *P.helva* by the color of the 1^st^ flagellomere (black in *P.luteola*; yellow in *P.helva*).

### ﻿28.﻿ Phytoliriomyza helva

Taxon classificationAnimaliaDipteraAgromyzidae

Kato sp. nov.

C2B4E586-FB37-519D-A92E-05D8E21AC92F

https://zoobank.org/F54BEE66-8544-474C-B9DB-2DD1C37B7E65

Figs 52 , 53

#### Material examined.

***Holotype*:** Japan: 1♂ (MK-AG-a540), Mitsumine, Chichibu, Saitama Pref. (35.9299°N, 138.9171°E, 630 m asl), 26-III-2021 (as larva on *C.purpureorubrum*), emerged on 17-V-2021, NSMT-I-Dip 32057. ***Paratypes*:** Japan: 1♀ (MK-AG-a541), same data as holotype, NSMT-I-Dip 32058. 2♂2♀ (MK-AG-a406, a471, a318, a470), Eniwa-keikoku, Eniwa, Hokkaido, 2-V-2021 (as larva on *C.salebrosum*), emerged on 10–15-VI-2021, NSMT-I-Dip 32059–32062.

#### Other material.

 Japan: On *Conocephalumsalebrosum*: 1♂, Mt. Horoiwa, Saroma, Tokoro, Hokkaido, 1-X-2016 (as larva), emerged on 4-V-2016; 1♂, Usuzuka, Fujinomiya, Funbe, Hiroo, Hokkaido Pref., 27-VIII-2014 (as larva), emerged on 16-V-2014.

On *Conocephalumpurpureorubrum*: 1♂1♀, Iwaobetsu, Shari, Hokkaido, 1-V-2021 (as larva), emerged on 11–15-VI-2021; 1♀, Samani-dam, Samani, Hokkaido, 30-IV-2021 (as larva), emerged on 15-VI-2021.

#### Diagnosis.

 A medium-sized yellow species (wing length 1.8–2.1 mm) having a pruinose light yellow scutum and scutellum, a yellow 1^st^ flagellomere, yellow maxillary palpus, yellow halteres, and yellow legs. Male epandrium inner-laterally with an extended, apically flattened tubercle-like seta, and inner-basally with a comb comprising three or four long fused tubercle-like setae. Larva mines the thallus of *Conocephalumsalebrosum* and *C.purpureorubrum*.

#### Description.

**Figure 52.**
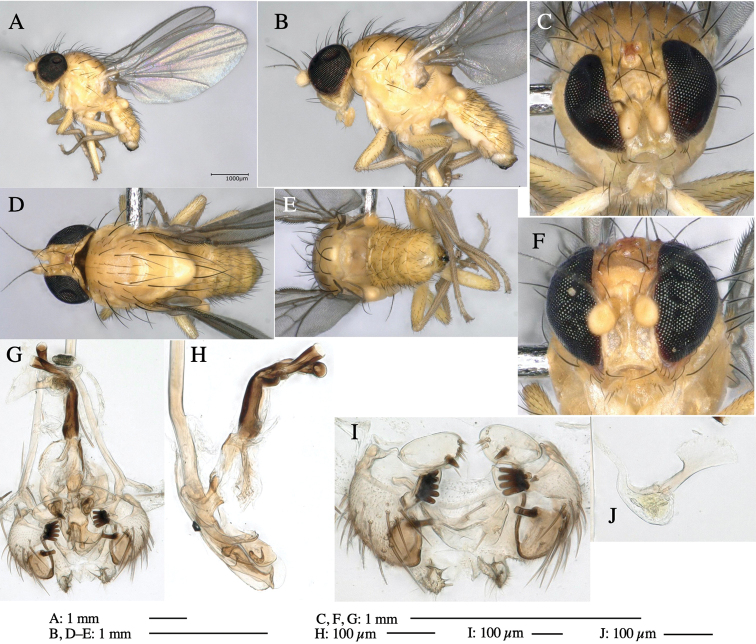
*Phytoliriomyzahelva*sp. nov. **A–E **holotype male **A** habitus **B** dorsal **C** frontal **D** dorsal **E** posterior **F** paratype female (MK-AG-a541), frontal **G–J** male genitalia **G** whole genitalia, ventral **H** phallic complex, lateral **I** epandrium, ventral **J** ejaculatory apodeme, lateral.

**Adult male** (Fig. 52A–E).

***Head*:** Head yellow, with back of head dark brown excluding margins (Fig. 52C). Antenna porrect, first flagellomere, pedicel and scape yellow (Fig. 52B). Arista subbasal, black but basally yellow, pubescent. Clypeus, face, gena, parafacial and postgena yellow. Proboscis normal, yellow; palpus yellow, cylindrical (Fig. 52C). ***Chaetotaxy*:** Front orbitals three pairs; one ori directed inward; two ors directed upward (Fig. 52D). Orbital setulae minute and erect, in a single row.

***Thorax*:** Thorax pruinose. Scutum entirely light yellow (Fig. 52D). Scutellum, subscutellum, mediotergite, anatergite, and katatergite yellow (Fig. 52E). Pleuron entirely yellow (Fig. 52B). Haltere yellow. Calypter margin and hairs gray. Leg segments entirely yellow; tibia and tarsus darker. ***Chaetotaxy*:** Scutum with 1+3 dorsocentrals, shortened anteriorly (Fig. 52D). Acrostichal setulae five or six pairs in two irregular rows. ***Wing*:** Wing length 2.0 mm, costa reaching M_1_ (Fig. 52A). Length of ultimate section of vein M_4_ divided by penultimate section 1.1–1.3.

***Abdomen*:** Abdomen dorsally subshiny yellow; epandrium brown (Fig. 52E). ***Genitalia*:** (Fig. 52G–J) Epandrium rounded apically;inner-lateral surface with an elongated tubercle-like seta, whose tip is slightly spread and flattened; inner-basal surface with a comb comprising 3–5 fused long tubercle-like setae (rarely unfused in part) and a row of two short tubercle-like setae immediately outward from the comb (Fig. 52I). Surstylus rounded, directed inwards, setose apically, with one long tubercle-like seta on posterior margin (Fig. 52I). Cercus narrow, setose. Subepandrial sclerite V-shaped, with bilobed dorsal plate and a pair of pale plate-like arms (Fig. 52I). Hypandrium slightly sclerotized along outer margin (Fig. 52G). Postgonite bare, goose barnacle-shaped, rounded apically (Fig. 52G). Phallophorus with deep incision below, articulated with phallapodeme, fused to epiphallus (Fig. 52G, H). Basiphallus with broad plate on left side and lightly sclerotized anterodorsal margin (Fig. 52G, H). Hypophallus hood-shaped, membranous covered with microtrichia ventrally; lateral margins lightly sclerotized; medially with a pair of fused narrow sclerites (Fig. 52H). Paraphallus pale membranous, undefined (Fig. 52H). Mesophallus dark, cylindrical, as long as distiphallus. Distiphallus comprising one pair of stout tubules; basal half composed of dark sclerite and weaker medial region; distal half cylindrical, pigmented; widening toward inflated, truncated apex (Fig. 52H). Ejaculatory apodeme pale, with fan-shaped blade and broad stalk; base wide to one side; sperm pump clear (Fig. 52J).

**Figure 53.**
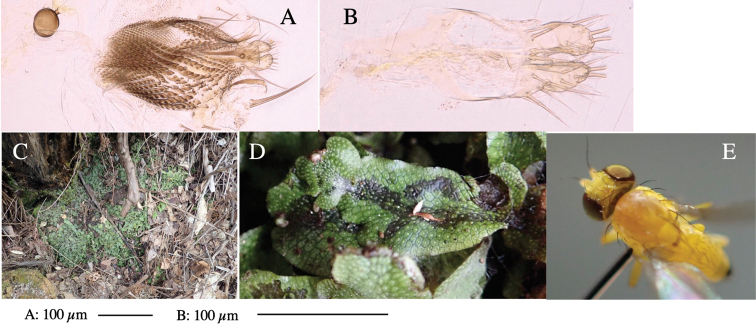
Female morphology and larval/adult ecology of *Phytoliriomyzahelva*sp. nov. **A, B **female postabdomen **A** oviscape and spermatheca **B** tergite 10 **C** habitat at Eniwa **D** mined thallus of *Conocephalumsalebrosum***E** live fly at Eniwa.

**Female** (Figs 52F, 53E). Similar to male, but slightly larger and frons wider. Wing length 2.1 mm. ***Postabdomen*:** (Fig. 53A, B) Oviscape dark brown, setigerous (Fig. 53A). Tergite 10 trifurcate, laterally uniting narrow pleural sclerites (Fig. 53B). Each cercus with two stout, apical, trichoid sensilla, 1/3 length of cercus (Fig. 53B). Spermathecae orbicular (Fig. 53A).

#### Variation.

The number of tubercle-like setae in a comb of the male epandrium varied from 3 to 5 among localities, with the individuals from northern localities having fewer tubercle-like setae. Pigmentation pattern in distiphallus and morphology of paraphallus also differed between Hokkaido and Honshu populations.

#### Etymology.

 The specific name (*helvus* = pale yellow) refers to pale yellow body and antennae of this species.

#### Japanese name.

Usuki-jagoke-hamoguribae.

#### Host plants.

*Conocephalumsalebrosum* and *C.purpureorubrum* (Conocephalaceae).

#### Mine.

Larvae construct linear mines in the thallus in early instars, later entering the midrib, and pupate in the mines (Fig. 53D).

#### Biological notes.

 The habitats of this species are stream banks and mesic slopes in cool temperate deciduous forests dominated by *Quercuscrispula* and *Ulmusdavidiana* (Fig. 53C). This species is sympatric with *P.luteola* at some localities. Our rearing records suggest that it is univoltine, and that adults emerge from overwintered pupae in spring.

#### Distribution.

 Japan: Hokkaido, Honshu (Fig. 50). Co-occurs with *P.luteola*.

#### Remarks.

This species resembles *P.pallidofasciata and P.luteola* in having wholly yellow body; it is distinguished from them by the color of the 1^st^ flagellomere (yellow in *P.helva*; black in the latter).

### ﻿29.﻿ Phytoliriomyza bifasciata

Taxon classificationAnimaliaDipteraAgromyzidae

Kato sp. nov.

6DCE2C37-2A7D-56CB-9E9B-3C41AFCC55B5

https://zoobank.org/37D392D3-B139-456D-A40D-A341EEDF2341

Fig. 54

#### Material examined.

***Holotype*:** Japan: 1♂ (MK-AG-a349), Ikawa-toge, Aoi-ku, Shizuoka Pref. (35.2768°N, 138.279°E, 1570 m asl), 26-V-2021 (as larva on *C.salebrosum*), emerged on 21-VI-2021, NSMT-I-Dip 32063. ***Paratypes*:** Japan: 1♂1♀ (MK-AG-a472, a473), same data as holotype, emerged on 11–16-V-2021, NSMT-I-Dip 32064, 32065; 1♀ (MK-AG-a382), Namari-kawa, Yakumo, Futami, Hokkaido, 6-VI-2021 (as larva on *C.purpureorubrum*), emerged on 15-VI-2021, NSMT-I-Dip 32066; 1♂ (MK-AG-542), Akka, Iwaizumi, Iwate Pref., 17-XI-2014 (as larva on *C.salebrosum*), emerged on 26-IV-2015, NSMT-I-Dip 32067; 1♂ (MK-AG-a242), Haccho-toge, Ogano, Chichibu, Saitama Pref., 14-XI-2010 (as larva on *C.purpureorubrum*), emerged on 6-V-2011, NSMT-I-Dip 32068; 1♀ (MK-AG-a272), Yoro-keikoku, Otaki, Isumi, Chiba Pref., 24-II-2012 (as larva on *C.salebrosum*), emerged on 9-V-2012, NSMT-I-Dip 32069; 1♀ (MK-AG-a274), Yashajin-toge, Minami-arupusu, Yamanashi Pref., 10-XII-2016 (as larva on *C.salebrosum*), emerged on 5-V-2017, NSMT-I-Dip 32070.

#### Other material.

 Japan: On *Conocephalumsalebrosum*: 1♂2♀, Akka, Iwaizumi, Iwate Pref., 17-XI-2014 (as larva), emerged on 26-IV–3-V-2014; 2♀, Otaki, Akiu, Taihaku, Sendai, Miyagi Pref., 14-XI-2014 (as larva), emerged on 22-IV-2014; 1♂, Yusen-kyo, Yamadera, Yamagata Pref., 15-XI-2014 (as larva), emerged on 1-V-2014; 2♀, Yashajin-toge, Minami-arupusu, Yamanashi Pref., 10-XII-2016 (as larva), emerged on 3–5-V-2016; 1♂1♀, Mt. Hakusan, Hakusan, Ishikawa Pref., 3-V-2013 (as larva), emerged on 24–31-V-2013.

On *Conocephalumorientalis*: 1♂1♀, Yachi, Kawaba, Gunma Pref., 14-IV-2012 (as larva), emerged on 20–24-V-2012; 1♀, Yoro-keikoku, Otaki, Isumi, Chiba Pref., 24-I-2012 (as larva), emerged on 9-V-2012; 1♂, Amagi-toge, Izu, Kaeda-keikoku, Kagamisu, Miyazaki, Miyazaki Pref. Pref., 17-II-2009 (as larva), emerged on 26-III-2009; 3♂, Ashikubo, Aoi-ku, Shizuoka Pref., 13-IV-2012 (as larva), emerged on 30-IV–1-V-2012; 1♀, Yasui-keikoku, Niyodogawa, Agawa, Kochi Pref., 27-II-2011 (as larva), emerged on 15-IV-2011; 1♀, Gokanosho, Itsuki, Kumamoto Pref., 23-III-2015 (as larva), emerged on 25-IV-2015; 1♂1♀, Shiibarui, Izumi, Yatsushiro, Kumamoto Pref., 23-III-2015 (as larva), emerged on 5–20-V-2015; 1♀, Mt. Kosho, Asakura, Fukuoka Pref., 11-IV-2010 (as larva), emerged on 4-V-2010.

On *Conocephalumpurpureorubrum*: 1♂1♀, Haccho-toge, Ogano, Chichibu, Saitama Pref., 14-XI-2010 (as larva), emerged on 26-IV–6-V-2010; 2♀, Mt. Toyoguchi, Ooshika, Shimo-ina, Nagano Pref., 30-IV-2012 (as larva), emerged on 1–5-VI-2012; 1♀, Mt. Ishizuchi, Kuma-kogen, Ehime Pref., 4-V-2014 (as larva), emerged on 16-V-2014.

#### Diagnosis.

 A large yellow species (wing length 2.2–2.3 mm) having a shiny yellow scutum with a medial and two pairs of black stripes, a black 1^st^ flagellomere, yellow maxillary palpus, yellow halteres, and yellow legs. Male epandrium inner-laterally with a hypertrophied tubercle-like seta, and inner-basally with a comb comprising three or four long fused tubercle-like setae. Larva mines the thallus of *Conocephalumsalebrosum*,*C.orientalis* and *C.purpureorubrum*.

#### Description.

**Adult male.**

**Figure 54.**
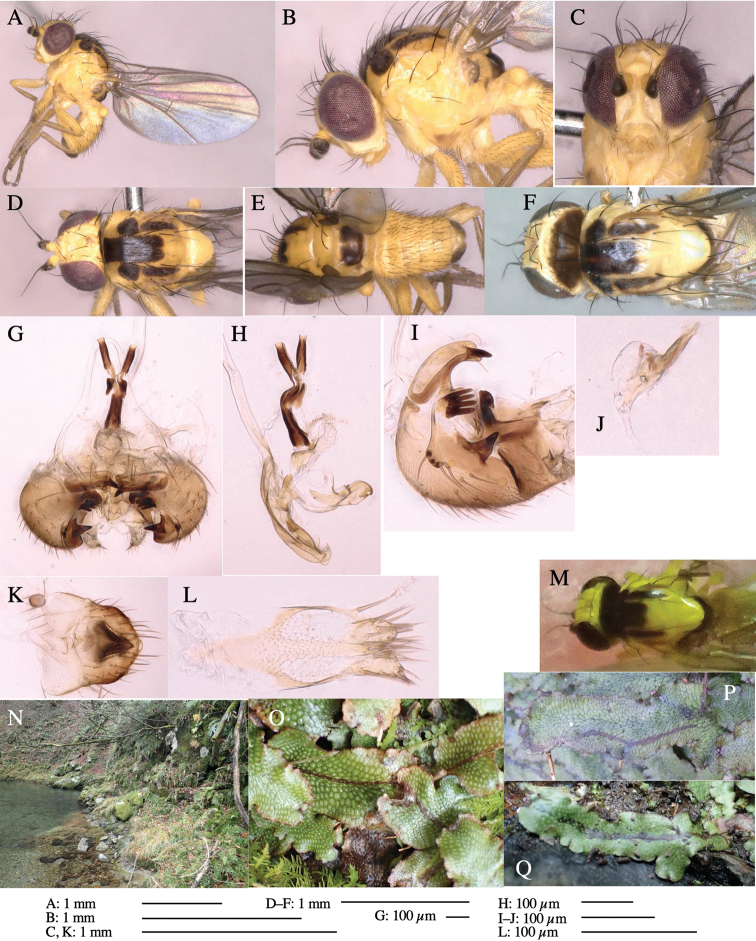
*Phytoliriomyzabifasciata*sp. nov. **A–E **holotype male **A** habitus **B** dorsal **C** frontal **D** dorsal **E** posterior **F** paratype female (MK-AG-a272), dorsal **G–J** male genitalia **G** whole genitalia **H** phallic complex **I** epandrium **J** ejaculatory apodeme, dorsal **K, L** female postabdomen **K** oviscape and spermatheca **L** tergite 10 **M** live female fly at Hozaka **N** habitat at Hachimori **O–Q** mined thalli (**O***Conocephalumorientalis* at Hachimori **P***C.salebrosum* at Haccho-toge **Q***C.salebrosum* at Akka).

***Head*:** (Fig. 54A–E) Head yellow, with back of head dark brown excluding margins. (Fig. 54C). Antenna porrect, first flagellomere black, pedicel and scape yellow (Fig. 54B). Arista subbasal, black, pubescent. Clypeus, face, gena, parafacial and postgena yellow. Proboscis normal, yellow; palpus yellow, cylindrical (Fig. 54C). ***Chaetotaxy*:** Front orbitals three pairs; one ori directed inward; two ors directed upward (Fig. 54B). Orbital setulae minute and erect, in a single row.

***Thorax*:** Thorax shiny. Scutum yellow with medial dark stripe on anterior 2/3, with a pair of narrow pale brown supra-alar stripes and a pair of wider black intra-alar stripes, which adjoin a pair of lateral presutural dark ovoid spots (Fig. 54D). Scutellum yellow, subscutellum yellow with brown margin. Mediotergite brown, anatergite yellow with small brown spot near lower margin, and katatergite yellow (Fig. 54E). Pleuron yellow with brownish patches on venter of katepisternum and meron (Fig. 54B). Haltere yellow. Calypter margin and hairs gray. Leg segments entirely yellow; tibia and tarsus darker (Fig. 54A). ***Chaetotaxy*:** Scutum with 1+3 dorsocentrals, shortened anteriorly. 20–26 acrostichal setulae in four irregular rows (Fig. 54D). ***Wing*:** Wing length 2.2 mm, costa reaching M_1_ (Fig. 54A). Length of ultimate section of vein M_4_ divided by penultimate section 1.3.

***Abdomen*:** Abdomen dorsally subshiny yellow; epandrium dark brown (Fig. 54E). ***Genitalia*:** (Fig. 54G–J) Epandrium rounded apically; inner-posterior margin with a dark ridge; inner-lateral surface with a flattened triangular tubercle-like seta; inner-anterior surface with a comb comprising three or four basally fused long tubercle-like setae (rarely unfused in part); inner-lateral margin with a row of three or four small tubercle-like setae (Fig. 54I). Surstylus elongated, curved inwards; with a few short setae apically; with one stout tubercle-like seta subapically (Fig. 54I). Cercus narrow, setose. Subepandrial sclerite with a pair of plate-like arms, the dorsal lobe of which is dark, hooked toward dorsum (Fig. 54I). Hypandrium slightly sclerotized along outer margin (Fig. 54G). Postgonite bare, goose barnacle-shaped, rounded apically, cleft subapically (Fig. 54H). Phallophorus with deep incision below, articulated with phallapodeme, fused to epiphallus (Fig. 54G). Basiphallus with a pale broad, plate-like sclerite on left side (Fig. 54G). Hypophallus broad, membranous covered with microtrichia ventrally; lateral margins lightly sclerotized; medially with a pair of fused narrow sclerites (Fig. 54H). Paraphallus absent. Mesophallus dark, cylindrical, widest basally, as long as distiphallus (Fig. 54H). Distiphallus comprising one pair of stout tubules basally parallel to each other; covered by a membrane bearing 7–9 pairs of minute oval lateral sclerites basal half composed of ventral dark subtriangular sclerite and weaker medial region; distal half cylindrical, dark; constricted subdistally; with truncated unpigmented apex (Fig. 54H). Ejaculatory apodeme pale brown, with fan-shaped blade and short stalk; base wide to one side; sperm pump clear (Fig. 54J).

**Female** (Fig. 54F, M). Similar to male, but slightly larger and frons wider. Wing length 2.3 mm. ***Postabdomen*:** (Fig. 54K, L) Oviscape dark brown, setigerous (Fig. 54K). Tergite 10 trifurcate, laterally uniting narrow pleural sclerites (Fig. 54L). Each cercus with two stout, apical, trichoid sensilla, 1/3 length of cercus (Fig. 54L). Spermathecae semi-orbicular, with truncate proximal ends (Fig. 54K).

#### Etymology.

 The specific name (*bifasciata* = two stripes) refers to a pair of black stripes on the yellow scutum.

#### Japanese name.

Tsuyasuji-jagoke-hamoguribae.

#### Host plants.

*Conocephalumsalebrosum* and *C.orientalis* (Conocephalaceae).

#### Mine.

Larvae construct linear mines in the midrib of the thallus, and pupate in the mines (Fig. 54O–Q).

#### Biological notes.

 The habitats of this species are stream banks and mesic slopes in cool temperate deciduous forests dominated by *Quercuscrispula*,*Faguscrenata* and *Aesculusturbinata* (Fig. 54N). It was sympatric with *P.izayoi*,*P.luteola*, *and P.conocephali* at some localities. Our rearing records suggest that this species is univoltine, and that adults emerge from overwintered pupae in spring.

#### Distribution.

**Figure 55.**
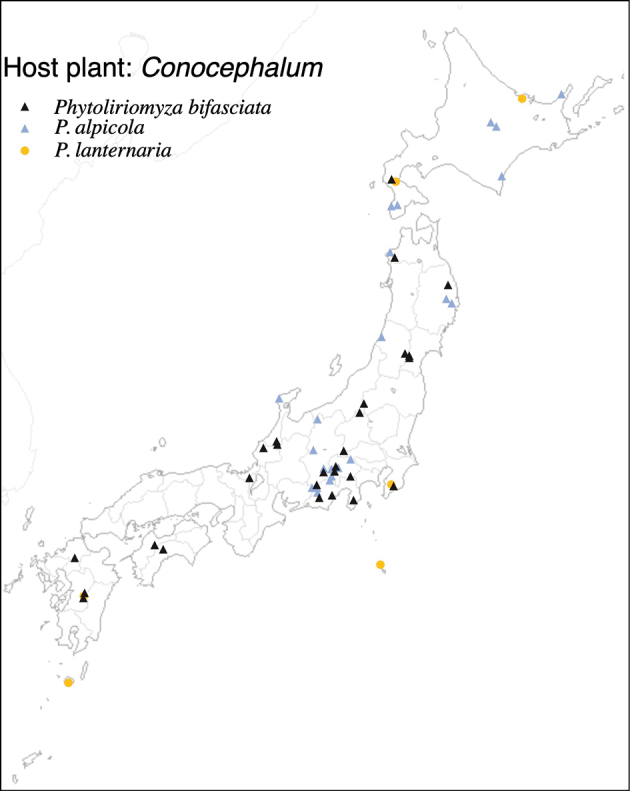
Locality records of three *Phytoliriomyza* species associated with *Conocephalum* spp.: *P.bifasciata*, *P.alpicola* and *P.lanternaria*.

Japan: Hokkaido, Honshu, Shikoku and Kyushu (Fig. 55).

#### Remarks.

 This species resembles *P.dorsata*,*P.calcicola*,*P.argentifasciata*,*P.longifurcae*,*P.brunofasciata*, and *P.pallidofasciata* in having two pairs of dark lateral stripes on the scutum; it is distinguished from all of them by the glossy scutum (subshiny in the other species) and by the dissimilarity of color between the outer and inner pairs of the stripes (color is similar between outer and inner pairs in the other species).

### ﻿30.﻿ Phytoliriomyza alpicola

Taxon classificationAnimaliaDipteraAgromyzidae

Strobl

9D688E02-82AE-5D24-A0A6-2FADBDDB1B2F

Figs 56 , 57

Agromyza alpicola Strobl, 1898: 272.
Liriomyza alpicola Hendel, 1931: 206.
Phytoliriomyza alpicola Spencer, 1971: 162.
Lemurimyza alpicola .Papp, 1984: 306.
Phytoliriomyza alpicola Sasakawa, 2008: 137; Černý et al. 2020: 213.

#### Material examined.

 Japan: On *Conocephalumsalebrosum*: 1♀, Yuni-ishikari-gawa, Soun-kyo, Kamikawa, Hokkaido, 5-VI-2016 (as larva), emerged on 22-VI-2016; 1♂, Samani-dam, Samani, Hokkaido, 1-VI-2021 (as larva), emerged on 3-VII-2021; 1♀, Renge-onsen, Itoigawa, Niigata Pref., 15-X-2011 (as larva), emerged on ?-VI-2011; 27♂36♀, Ikawa-toge, Aoi-ku, Shizuoka Pref., 26-V-2021 (as larva), emerged on 30-V–27-VI-2021.

On *Conocephalumorientalis*: 5♀, Iwadate, Hachimori, Happo, Yamamoto, Akita Pref., 16-XI-2014 (as larva), emerged on 2–18-V-2014; 1♀, Futto, Toei, Kitashidara, Aichi Pref., 9-III-2013 (as larva), emerged on 2-V-2013; 1♀, Nekata, Hamakita, Hamamatsu, Shizuoka Pref., 8-III-2012 (as larva), emerged on 8-V-2012; 1♀, Saruyama, Monzen, Wajima, Ishikawa Pref., 4-V-2013 (as larva), emerged on 3-VI-2013; 1♀, Chiisago, Kaminokuni, Hiyama, Hokkaido, 11-VI-2012 (as larva), emerged on 16-VI-2012.

On *Conocephalumpurpureorubrum*: 1♀, Iwaobetsu, Shari, Hokkaido, 3-X-2011 (as larva), emerged on 26-V-2011; 1♀, Irisawai, Oshika, Nagano Pref., 29-IV-2011 (as larva), emerged on 2-VI-2011; 1♂, Horoman-kyo, Samani, Hokkaido, 30-IV-2021 (as larva), emerged on 25-V-2021; 1♀, Tanneso, Rubeshibetsu, Hiroo, Hokkaido, 2-X-2011 (as larva), emerged on 19-V-2011; 1♂1♀, Kanna-gawa, Nakatsugawa, Chichibu, Saitama Pref., 19-VIII-2002 (as larva), emerged on 5-V-2002; 1♀, Irisawai, Oshika, Nagano Pref., 20-IV-2011 (as larva), emerged on 2-VI-2011.

#### Diagnosis.

A medium-sized dark species (wing length 1.7–1.8 mm) having pruinose dark gray scutum, yellow scutellum, a black 1^st^ flagellomere, dark maxillary palpus, dark halteres, and dark gray legs. Male epandrium inner-subdistally with a hypertrophied tubercle-like seta, and inner-basally with a comb comprising six or seven long fused tubercle-like setae. Larva mines the thallus of *Conocephalumsalebrosum*,*C.orientalis* and *C.purpureorubrum*.

#### Description.

**Adult male**.

**Figure 56.**
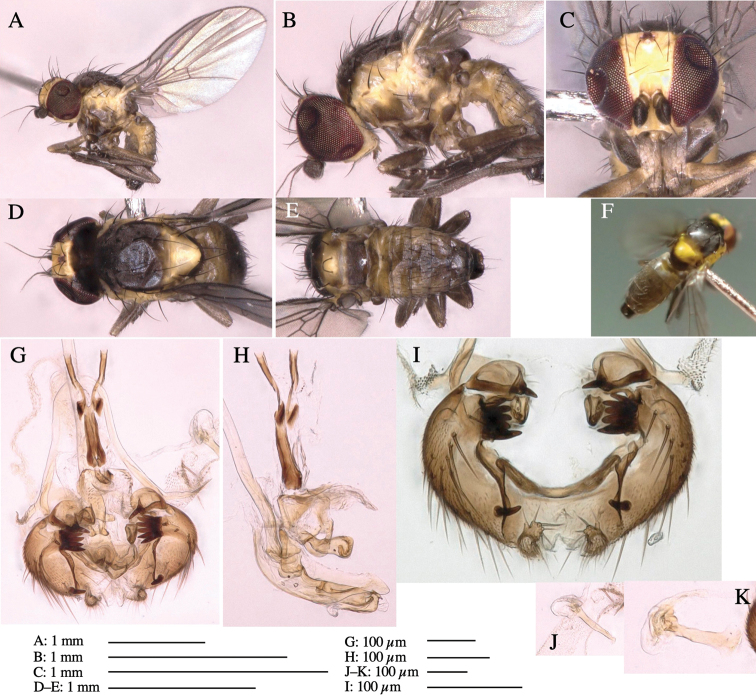
*Phytoliriomyzaalpicola***A–E **male at Ikawa-toge **A** habitus **B** dorsal **C** frontal **D** dorsal **E** posterior **F** live female fly at Mitsumine-Jinja **G–K** male genitalia (**G–J** at Ikawa-toge **K** at Samani) **G** whole genitalia **H** phallic complex **I** epandrium **J, K** ejaculatory apodeme, ventral and lateral.

***Head*:** (Fig. 56A–E) Head yellow, with ocellar tubercle brown, back of head dark brown (Fig. 56C). Antenna porrect, first flagellomere black, pedicel and scape yellow (Fig. 56B). Arista subbasal, black, pubescent. Clypeus, face, gena, parafacial and postgena yellow (Fig. 56C). Proboscis normal, yellow; palpus yellow, cylindrical. ***Chaetotaxy*:** Front orbitals three pairs; one ori directed inward; two ors directed upward (Fig. 56B). Orbital setulae minute and erect, in a single row.

***Thorax*:** Thorax subshiny. Scutum pruinose, dark gray, sometimes with very narrow terminal yellow band along posterior margin (Fig. 56D). Scutellum light yellow with lateral corner narrowly grayish. Subscutellum yellow except brown posterior margin. Mediotergite, anatergite and katatergite dark gray (Fig. 56E). Pleuron largely yellow, but notopleuron with narrow brown patch along ventral margin, and venters of propleuron, anepisternum, katepisternum, anepimeron, and meron dark brown (Fig. 56B). Haltere dark gray. Calypter margin and hairs gray. Leg segments entirely brown; tibia and tarsus darker (Fig. 56A). ***Chaetotaxy*:** Scutum with 1+3 dorsocentrals, shortened anteriorly (Fig. 56D). Acrostichal setulae five or six pairs in two irregular rows. ***Wing*:** Wing length 1.8 mm, costa reaching M_1_ (Fig. 56A). Length of ultimate section of vein M_4_ divided by penultimate section 1.5–1.6.

***Abdomen*:** Abdomen dorsally subshiny brown; epandrium dark brown (Fig. 56E). ***Genitalia*:** (Fig. 56G–K) Epandrium rounded apically; posterior end of inner margin with a long, inward-curved tubercle-like seta; inner-anterior surface with a comb comprising six or seven fused long tubercle-like setae (rarely unfused in part), and with a separate long tubercle-like seta, which is located immediately beyond the dorsal-most tubercle-like seta of the comb and directed to different angle; inner-basal margin with a row of 1–3 small tubercle-like setae immediately outward from the comb (Fig. 56I). Surstylus rounded, directed inwards, setose apically; with one long tubercle-like seta on posterior margin (Fig. 56I). Cercus narrow, setose. Subepandrial sclerite dark with a pair of plate-like arms, dorsal lobe spatula-shaped, with one seta subapically (Fig. 56I). Hypandrium slightly sclerotized along outer margin (Fig. 56G). Postgonite bare, goose barnacle-shaped, rounded apically (Fig. 56H). Phallophorus with deep incision below, articulated with phallapodeme, fused to epiphallus (Fig. 56G). Basiphallus with dark broad plate-like sclerite on left side (Fig. 56G). Hypophallus broad, membranous covered with microtrichia ventrally; lateral margins lightly sclerotized; medially with a pair of fused narrow sclerites (Fig. 56G, H). Paraphallus membranous, wing-like; paraphalli spread laterally, jointed basally (Fig. 56H). Mesophallus dark, cylindrical, widest basally, as long as distiphallus (Fig. 56H). Distiphallus comprising one pair of stout tubules basally parallel to each other; covered by a membrane bearing several pairs of minute oval lateral sclerites; basal half composed of ventral dark subtriangular sclerite and weaker medial region; distal half cylindrical, pigmented, widening toward truncated, unpigmented apex (Fig. 56H). Ejaculatory apodeme pale brown, fan-shaped with long stalk; sperm pump clear, with dark globular sclerites (Fig. 56I, J).

**Figure 57.**
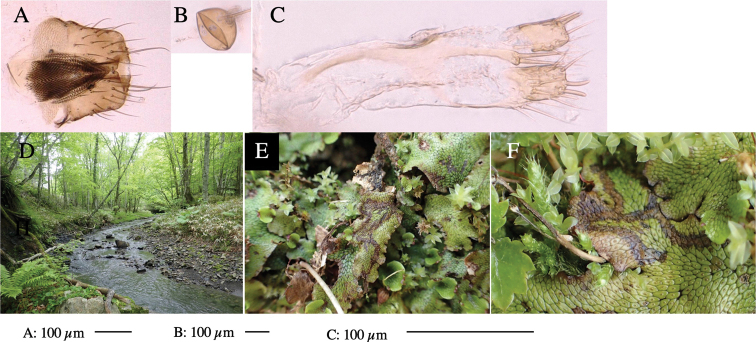
Female morphology and larval ecology of *Phytoliriomyzaalpicola*sp. nov. **A–C **female postabdomen **A** oviscape and spermatheca **B** spermatheca **C** tergite 10 **D** habitat at Samani **E, F** mined thalli of *Conocephalumsalebrosum*at Ikawa-toge).

**Female** (Fig. 56F). Similar to male, but slightly larger and frons wider. Wing length 1.7 mm. ***Postabdomen*:** (Fig. 57A–C) Oviscape dark brown, setigerous (Fig. 57A). Tergite 10 trifurcate, laterally uniting narrow pleural sclerites (Fig. 57C). Each cercus with two stout, apical, trichoid sensilla, 1/3 length of cercus (Fig. 57C). Spermathecae semi-orbicular, with truncate proximal ends (Fig. 57B).

#### Variation.

The morphology of the tubercle-like seta on the inner-distal margin of the male epandrium varied among localities from a simple seta to a flattened, basally enlarged acute spine. The relative position of a comb of tubercle-like setae and the separate tubercle-like seta neighboring the comb also varied among localities.

#### Japanese name.

Mihikari-jagoke-hamoguribae.

#### Host plants.

*Conocephalumsalebrosum*,*C.orientalis* and *C.purpureorubrum* (Conocephalaceae).

#### Mine.

Larvae construct linear mines in the thallus in early instars, later entering the midrib, and pupate in the mines (Fig. 57E, F).

#### Biological notes.

 The habitats of this species are stream banks and mesic slopes in cool temperate deciduous forests dominated by *Quercuscrispula* and *Faguscrenata*, riparian forests dominated by *Cercidiphyllumjaponicum* (Fig. 57D), and subalpine coniferous forests dominated by *Abies* spp. And *Picea* spp. It is sympatric with *P.luna*,*P.brunofasciata*, *and P.luteola* at some localities. Our rearing records suggest that this species is univoltine, and that adults emerge from overwintered pupae in spring.

#### Distribution.

Japan: Hokkaido, Honshu (Fig. 55).

#### Remarks.

 This species was reported from Scotland by Strobl (1898), and later recorded from Austria (Spencer 1972) and Taiwan (Sasakawa 2008). It resembles *P.tsukuyomi* in having an almost wholly dark scutum and almost wholly yellow scutellum, but is distinguished by the color of the pedicel of the antenna (brown in *P.alpicola*; yellow in *P.tsukuyomi*) and by the number and arrangement of tubercle-like setae in a comb on the male epandrium (6 fused in *P.alpicola*; 4 hand-like in *P.tsukuyomi*). *P.alpicola* also resembles *P.marchantiae*,*P.lanternaria*,*P.rebouliae*, and *P.conocephali* in having dark brown scutum and yellow scutellum; it is distinguished from them by the absence of a small medial yellow mark on the posterior margin of the scutum, and by the absence of dark bands at the lateral margins of the yellow scutellum.

### ﻿31.﻿ Phytoliriomyza lanternaria

Taxon classificationAnimaliaDipteraAgromyzidae

Kato sp. nov.

39222827-34A0-505D-9F06-6B46FD8A05CF

https://zoobank.org/A8BEF529-86DC-4CBC-AA58-4ADFFAF30AAD

Fig. 58

#### Material examined.

***Holotype*:** Japan: 1♂ (MK-AG-a290), Hachijo Is., Tokyo Pref. (33.1114°N, 139.8271°E, 190 m asl), 17-II-2012 (as larva on *C.orientalis*), emerged on 23-IV-2013, NSMT-I-Dip 32071. ***Paratypes*:** Japan: 1♂2♀ (MK-AG-a5, a430, 730), same data as holotype, emerged on 8-IV–2-V-2013, NSMT-I-Dip 32072–32074; 1♀ (MK-AG-a473), Anbo, Yaku Is., Kumage, Kagoshima Pref., 30-III-2017 (as larva on *C.orientalis*), emerged on 15-VII-2017, NSMT-I-Dip 32075.

#### Other material.

 Japan: On *Conocephalumorientalis*: 2♂, Mt. Horoiwa, Saroma, Tokoro, Hokkaido, 1-V-2021 (as larva), emerged on 7-VI-2021; 1♀, Namari-kawa, Yakumo, Futami, Hokkaido, 2-VI-2021 (as larva), emerged on 16-VI-2016; 3♂9♀, Hachijo Is., Tokyo Pref., 17-II-2012 (as larva), emerged on 8-IV–2-V-2012; 1♀, Fuchigasawa, Kimitsu, Chiba Pref., 13-V-2008 (as larva), emerged on 31-V-2013.

#### Diagnosis.

 A medium-sized dark species (wing length 1.8–1.9 mm) having pruinose dark gray scutum with mid-posterior yellow margin, yellow scutellum with dark lateral corners, black 1^st^ flagellomere, dark maxillary palpus, dark halteres, and yellowish brown legs. Male epandrium inner-laterally with a long ventrally directed tubercle-like seta, and inner-basally with a siku-shaped comb comprising seven fused tubercle-like setae.

Larva mines the thallus of *Conocephalumorientalis*.

#### Description.

**Figure 58.**
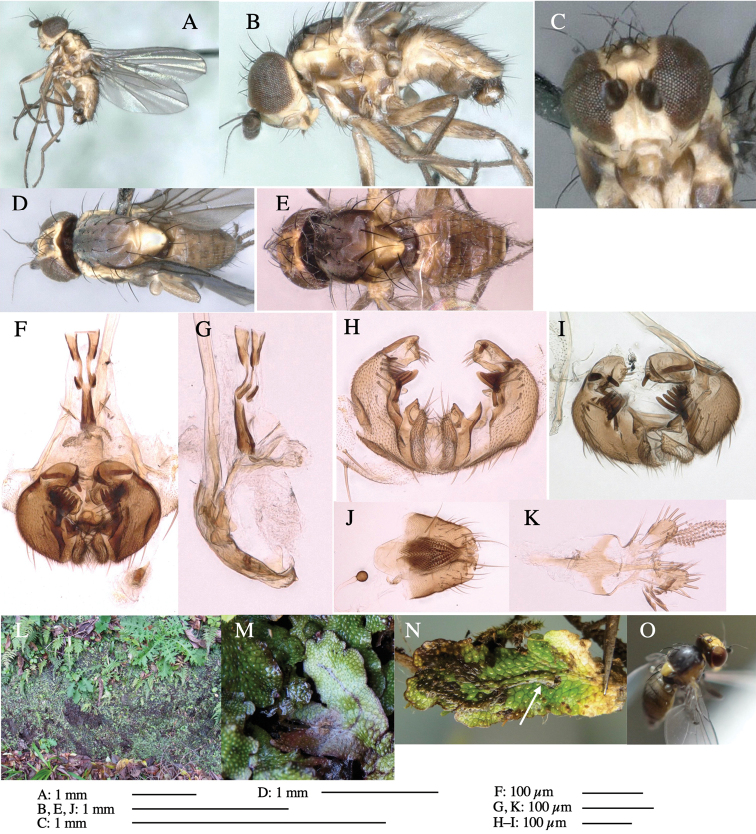
*Phytoliriomyzalanternaria*sp. nov. **A–D **holotype male **A** habitus **B** dorsal **C** frontal **D** dorsal **E** paratype female (MK-AG-430), dorsal **F–I** male genitalia (**F–H** at type locality **I** Mt. Horoiwa) **F** whole genitalia, ventral **G** phallic complex, lateral **H, I** epandrium, ventral, **J, K** female postabdomen **J** oviscape and spermatheca **K** tergite 10 **L** habitat at type locality **M, N** mined thalli of *Conocephalumorientalis*. An arrow in **N** indicates an internal puparium **O** live female fly at Yakumo.

**Adult male** (Fig. 58A–D).

***Head*:** Head light yellow, with ocellar tubercle dark brown, frons yellowish brown, back of head dark brown excluding margins (Fig. 58C). Antenna porrect, first flagellomere black, pedicel and scape brown (Fig. 58B). Arista subbasal, black, pubescent. Clypeus, face, gena, parafacial and postgena yellow. Proboscis normal, yellow; palpus brown, cylindrical (Fig. 58C). ***Chaetotaxy*:** Front orbitals three pairs; one ori directed inward; two ors directed upward (Fig. 58B). Orbital setulae minute and erect, in a single row.

***Thorax*:** Thorax pruinose. Scutum pruinose gray, with a small yellow patch along midposterior margin (Fig. 58D). Scutellum light yellow with lateral corner brown, subscutellum light yellow. Mediotergite and anatergite brown, katatergite light yellow (Fig. 58B). Pleuron yellow with brownish patches on venter of propleuron, anepisternum, katepisternum, anepimeron, and meron (Fig. 58C). Haltere yellow but light yellow basally. Calypter margin and hairs gray. Leg segments brownish, basal half of femur paler (Fig. 58A). ***Chaetotaxy*:** Scutum with 1+3 dorsocentrals, shortened anteriorly (Fig. 58B). Acrostichal setulae five or six pairs in two rows. ***Wing*:** Wing length 2.2 mm, costa reaching M_1_ (Fig. 58A). Length of ultimate section of vein M_4_ divided by penultimate section 1.3.

***Abdomen*:** Abdomen dorsally subshiny brown; epandrium dark brown (Fig. 58B). ***Genitalia*:** (Fig. 58F–I) Epandrium rounded apically; inner-lateral surface with a long, anteriorly directed, tubercle-like seta; inner-anterior surface with a siku-shaped comb comprising seven fused tubercle-like setae, which are reduced in length toward base of surstylus (Fig. 58H, I). Surstylus rounded, curved inwards, setose subapically, with one long tubercle-like seta apically (Fig. 58H). Cercus narrow, setose. Subepandrial sclerite with a pair of plate-like arms, each having a posterior lobe with trilobed projection and ventrally projected lateral plates (Fig. 58H). Hypandrium slightly sclerotized along outer margin (Fig. 58F). Postgonite bare, goose barnacle-shaped, pointed apically (Fig. 58F). Phallophorus with deep incision below, articulated with phallapodeme, fused to epiphallus (Fig. 58F, G). Basiphallus with pale broad plate-like sclerite on left side (Fig. 58F). Hypophallus broad, membranous covered with microtrichia ventrally; lateral margins lightly sclerotized; medially with a pair of fused, narrow, ventrally incurved sclerites (Fig. 58F, G). Paraphallus pale, membranous, and wing-like; posterior margins lightly sclerotized; paraphalli diverging, angled anteroventrally, jointed basally (Fig. 58F, G). Mesophallus dark, cylindrical, widest subbasally, as long as distiphallus (Fig. 58G). Distiphallus comprising one pair of stout tubules basally parallel to each other; basal half composed of ventral dark subtriangular sclerite and weaker medial region; distal half cylindrical, dorsally pigmented, widening toward truncated, flared apex (Fig. 58G). Ejaculatory apodeme pale brown, fan-shaped with short broad stalk and clear sperm pump (Fig. 58F).

**Female** (Fig. 58E, O). Similar to male, but slightly larger and frons wider. Wing length 2.3 mm. ***Postabdomen*:** (Fig. 58J, K) Oviscape dark brown, setigerous (Fig. 58J). Tergite 10 cruciform, laterally uniting narrow pleural sclerites (Fig. 58K). Each cercus with two stout, apical, trichoid sensilla, 1/3 length of cercus (Fig. 58K). Spermathecae orbicular (Fig. 58J).

#### Variation.

Color pattern of scutum and subscutellum varied among localities. In specimens from Hachijo Island, the subscutellum had a large lateral dark corner.

#### Etymology.

 The specific name (*lanterna* = lantern) refers to the faint yellow spot on the scutellum, which reminds us of a lantern light.

#### Japanese name.

Tomoshibi-jagoke-hamoguribae.

#### Host plants.

*Conocephalumorientalis* (Conocephalaceae) growing on mesic soils in various types of forests.

#### Mine.

Larvae construct linear mines in the thallus in early instars, later entering the midrib, and pupate in the mines (Fig. 58M, N).

#### Biological notes.

 The habitats of this species are stream banks and mesic slopes in warm temperate evergreen forests dominated by *Castanopsiscuspidata* and cool temperate deciduous forests dominated by *Quercuscrispula* (Fig. 58L). It is sympatric with *P.luteola* and *P.conocephali* at some localities. Our rearing records suggest that this species is univoltine, and that adults emerge from overwintered pupae in spring.

#### Distribution.

Japan: Hokkaido, Honshu, Hachijo Island, and Yaku Island (Fig. 55).

#### Remarks.

 This species resembles *P.marchantiae*,*P.rebouliae*, and *P.conocephali* in having a small yellow mark lying between the posterior scutum and subscutellum, but is distinguished from them by its larger size (wing length ≥ 1.9 mm in *P.lanternaria*;&nbsp;<&nbsp;1.8&nbsp;mm in the other species). It is also distinguished from *P.marchantiae* and *P.rebouliae* by the absence of a tubercle-like seta on the surstylus of the male epandrium, and from *P.conocephali* by the number of tubercle-like setae in a comb of the male epandrium (7 in *P.lanternaria*; 5–6 in *P.conocephali*). This species resembles *P.alpicola* in the color patterns of the scutum, but is distinguished from the latter by its gray scutum (scutum darker in *P.alpicola*), dark-sided scutellum (scutum dark only on a marginal narrow lateral area in *P.alpicola*), and number and arrangement of tubercle-like setae on the male epandrium (a siku-shaped comb composed of seven differently sized setae in *P.lanternaria*; a comb composed of six equally long setae in *P.alpicola*).

### ﻿32.﻿ Phytoliriomyza conocephali

Taxon classificationAnimaliaDipteraAgromyzidae

Kato sp. nov.

6A8ECAF1-C853-5FE0-87DE-E0C6F054A932

https://zoobank.org/10851865-0EEE-4AE0-BB92-C5858AB04C1B

Figs 59 , 60

#### Material examined.

***Holotype*:** Japan: 1♂ (MK-AG-a269), Ashiu, Nantan, Kyoto Pref. (35.3261°N, 135.7239°E, 450 m asl), 29-XI-1998 (as larva on *Conocephalumorientalis*), emerged on 26-V-1999, NSMT-I-Dip 32076. ***Paratypes*:** Japan: 1♂ (MK-AG-a408), type locality, 8-IV-2012 (as larva on *Conocephalumorientalis*), emerged on 13-V-2012, NSMT-I-Dip 32077; 2♂ (MK-AG-a444, a445), Ashiu, Nantan, Kyoto Pref., 13-XI-2001 (as larva on *C.japonicum*), emerged on ?-IV-2019, NSMT-I-Dip 32078–32079; 1♂ (MK-AG-a9), Soun-kyo, Kamikawa, Hokkaido, 18-X-2018 (as larva on *C.japonicum*), emerged on 7-V-2019, NSMT-I-Dip 32080; 1♂ (MK-AG-a8), Dainichi, Kakegawa, Shizuoka Pref., 3-I-2016 (as larva on *C.japonicum*), emerged on 24-IV-2016, NSMT-I-Dip 32081; 1♀ (MK-AG-726), Saruyama, Monzen, Wajima, Ishikawa Pref., 4-V-2013 (as larva on *C.orientalis*), emerged on 3-VI-2013, NSMT-I-Dip 32082; 1♀ (MK-AG-a7), Muramatsu, Iwakura, Sakyo-ku, Kyoto Pref., 2-III-2019 (as larva on *C.orientalis*), emerged on 16-IV-2019, NSMT-I-Dip 32083; 1♀ (MK-AG-a6), Chikatsuyu, Nakaheji, Tanabe, Wakayama Pref., 3-III-2012 (as larva on *C.orientalis*), emerged on 9-IV-2012, NSMT-I-Dip 32084.

#### Other material.

 Japan: On *C.orientalis*: 3♂3♀, Yusen-kyo, Yamadera, Yamagata Pref., 15-IV-2012 (as larva), emerged on 19-V–2-VI-2012; Hosorogi, Awara, Ishikawa Pref., 1-IV-2011 (as larva), emerged on 5–24-V-2011; 4♂3♀, Suizu, Tsuruga, Fukui Pref., 11-III-2012 (as larva), emerged on 12-IV–8-V-2012; 1♂♀, Seryo, Sakyo-ku, Kyoto Pref., 22-IX-2002 (as larva), emerged on 15–16-V-2002; 5♂15♀, Muramatsu, Iwakura, Sakyo-ku, Kyoto Pref., 5-IV-2017 (as larva), emerged on 12–22-IV-2017; 1♂2♀, Mt. Gyojagaeri, Kamikitayama, Nara Pref., 31-VII-1999 (as larva), emerged on 25-VIII–5-X-1999; 4♂5♀, Wadagawa-kyo, Kumanogawa, Shingu, Wakayama Pref., 7-VII-2021 (as larva), emerged on 26-VII–5-VIII-2021; 6♀, Wabuka, Kushimoto, Wakayama Pref., 4-III-2012 (as larva), emerged on 9-IV–23-V-2017; 2♂8♀, Taishaku-kyo, Shobara, Hiroshima Pref., 9-IV-2011 (as larva), emerged on 15–27-V-2011; 2♂4♀, Narutaki, Ichiu, Tsurugi, Tokushima Pref., 31-III-2021 (as larva), emerged on 11-V–2-VI-2021; 1♂1♀, Yasui-keikoku, Niyodogawa, Agawa, Kochi Pref., 27-II-2011 (as larva), emerged on 24–26-IV-2011; 2♂14♀, Nanatsudake, Tamanoura, Fukue Is. Goto, Pref., 9-X-1998 (as larva), emerged on 20-XI-1998–4-IV-1999; 3♂4♀, Gokanosho, Itsuki, Kumamoto Pref., 10-IV-2021 (as larva), emerged on 10–221-IV-2021; 1♂4♀, Anbo, Yaku Is., Kumage, Kagoshima Pref., 30-II-2017 (as larva), emerged on ?-V-2017.

On *Conocephalumpurpureorubrum*: 2♀, Toikanbetsu, Horonobe, Teshio, Hokkaido Pref., 5-X-2013 (as larva), emerged on ?-V-2013; 1♀, Yoro-keikoku, Otaki, Isumi, Chiba Pref., 17-III-2016 (as larva), emerged on 22-IV-2013; 1♀, Shirabiso-toge, Kamimura, Iida, Nagano Pref., 14-X-2011 (as larva), emerged on 18-V-2012.

On *Conocephalumsalebrosum*: 1♀, Shirabiso-toge, Kamimura, Iida, Nagano Pref., 14-X-2011 (as larva), emerged on 18-V-2012; 1♂, Usuzuka, Fujinomiya, Shizuoka Pref., 15-VI-2013 (as larva), emerged on 8-VII-2013.

On *Conocephalumjaponicum*: 1♂1♀, Mt. Teine, Teine-ku, Sapporo, Hokkaido, 24-VII-2011 (as larva), emerged on 15–17-VIII-2011; 1♂4♀, Nishikawa, Nishimurayama, Yamagata Pref., 15-IV-2011 (as larva), emerged on 19-V–8-VI-2012; 4♂8♀, Dainichi, Kakegawa, Shizuoka Pref., 3-I-2016 (as larva), emerged on 21-IV–1-V-2016; 4♂8♀, Soun-kyo, Kamikawa, Hokkaido, 18-X-2016 (as larva), emerged on 20-IV–7-V-2016; 4♂5♀, Wadagawa-kyo, Kumanogawa, Shingu, Wakayama Pref., 7-VII-2021 (as larva), emerged on 26-VII–5-VIII-2021; 3♂4♀, Gokanosho, Itsuki, Kumamoto Pref., 10-IV-2021 (as larva), emerged on 10–221-IV-2021.

#### Diagnosis.

 A small dark species (wing length 1.3–1.7 mm) having a pruinose dark gray scutum with a mid-posterior yellow margin, a yellow scutellum with dark lateral corners, a black 1^st^ flagellomere, dark maxillary palpus, dark halteres, and yellowish brown legs. Male epandrium inner-subdistally with a long ventrally directed tubercle-like seta, inner-laterally with a tubercle like seta, and inner-basally with a comb comprising five fused tubercle-like setae. Larva mines the thallus of all Japanese *Conocephalum* spp.

#### Description.

**Figure 59:**
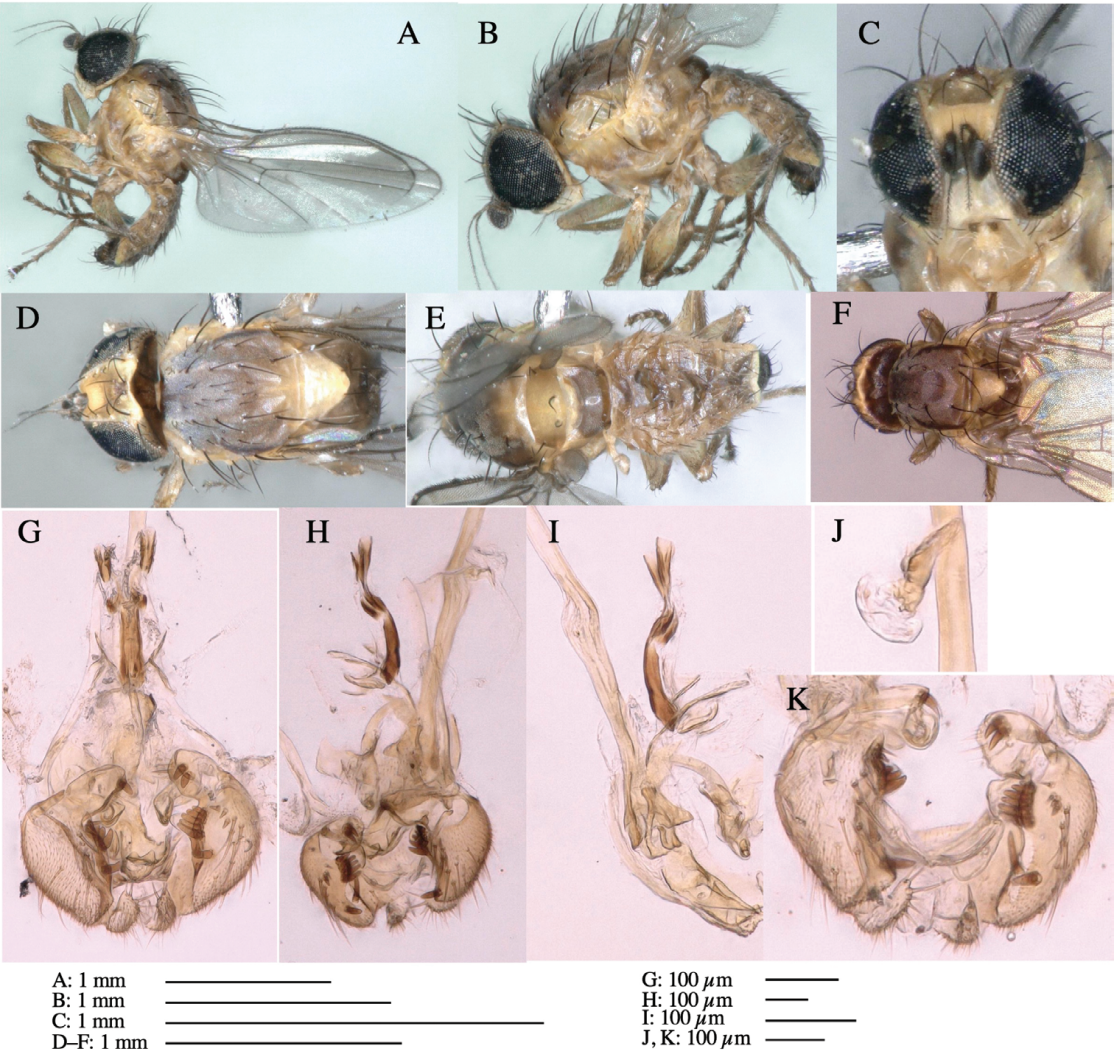
*Phytoliriomyzaconocephali*sp. nov. **A–E **holotype male **A** habitus **B** dorsal **C** frontal **D** dorsal **E** posterior **F** paratype female (MK-AG-a444), dorsal **G–K** male genitalia (**G **on *Conocephalumorientalis***H–K** on *C.japonicum*) **G, H** whole genitalia, ventral **I** phallic complex, lateral **J** ejaculatory apodeme, dorsal **K** epandrium, ventral.

**Adult male** (Fig. 59A–E).

***Head*:** Head light yellow, with ocellar tubercle dark brown, frons yellowish brown, back of head dark brown excluding margins (Fig. 59C). Antenna porrect, first flagellomere black, pedicel and scape brown (Fig. 59B). Arista subbasal, black, pubescent. Clypeus, face, gena, parafacial and postgena yellow. Proboscis normal, yellow; palpus brown, cylindrical (Fig. 59C). ***Chaetotaxy*:** Front orbitals three pairs; one ori directed inward; two ors directed upward (Fig. 59D). Orbital setulae minute and erect, in a single row.

***Thorax*:** Thorax pruinose. Scutum pruinose gray, with a small yellow patch along midposterior margin (Fig. 59D). Scutellum light yellow with lateral corner brown, subscutellum light yellow. Mediotergite and anatergite brown, katatergite light yellow (Fig. 59E). Pleuron yellow with brownish patches on venter of propleuron, anepisternum, katepisternum, anepimeron, and meron (Fig. 59B). Haltere brown. Calypter margin and hairs gray. Leg segments brownish, basal half of femur paler (Fig. 59A). ***Chaetotaxy*:** Scutum with 1+3 dorsocentrals, shortened anteriorly (Fig. 59D). Acrostichal setulae five or six pairs in irregular two rows. ***Wing*:** Wing length 2.2 mm, costa reaching M_1_ (Fig. 59A). Length of ultimate section of vein M_4_ divided by penultimate section 1.3.

***Abdomen*:** Abdomen dorsally subshiny brown; epandrium dark brown (Fig. 59E). ***Genitalia*:** (Fig. 59G–K) Epandrium rounded apically; inner-subposterior surface with a l tubercle-like seta; inner-lateral margin with a long, anteriorly directed, tubercle-like seta; inner-anterior surface with a comb comprising five or six fused long tubercle-like setae (Fig. 59K). Surstylus oblong, curved inwards, setose apically, with two long tubercle-like setae on posterior subdistal margin (Fig. 59K). Cercus narrow, setose. Subepandrial sclerite V-shaped in a posterior view, with pale plate-like arms. Hypandrium slightly sclerotized along outer margin (Fig. 59G). Postgonite bare, goose barnacle-shaped, rounded apically (Fig. 59I). Phallophorus with deep incision below, articulated with phallapodeme, fused to epiphallus (Fig. 59G–I). Basiphallus with broad plate-like sclerite on left side. Hypophallus broad, membranous covered with microtrichia ventrally; medially with a pair of fused narrow ventrally incurved sclerites (Fig. 59G–I). Paraphallus pale membranous, bilobed; medial axis and margin lightly sclerotized; paraphalli spread laterally, jointed basally, resembling 4 wings (Fig. 59G–I). Mesophallus dark, cylindrical, as long as distiphallus (Fig. 59I). Distiphallus comprising one pair of stout tubules basally parallel to each other; basal half composed of ventral dark subtriangular sclerite and weaker medial region; distal half cylindrical, dorsally pigmented, widening toward truncated, flared apex (Fig. 59I). Ejaculatory apodeme pale brown, with fan-shaped blade and short broad stalk; base wide to one side; sperm pump clear (Fig. 59J).

**Figure 60.**
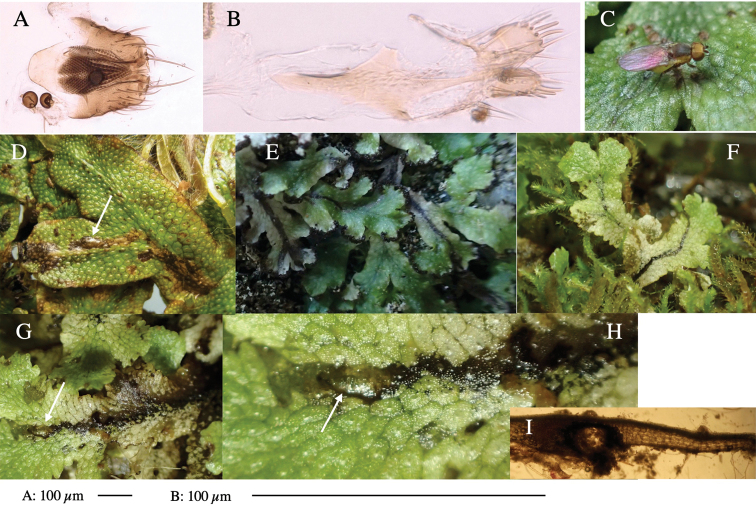
Female morphology and larval/adult ecology of *Phytoliriomyzaconocephali*sp. nov. **A, B **female postabdomen **A** oviscape and spermatheca **B** tergite 10 **C** a fly walking on thallus of *Conocephalumjaponicum* at Kawazako-gawa **D–H** mined thalli (**D** on *C.orientalis***E–H** on *C.japonicum***D** at Hamakita **E** at Kakegawa **F–H** at Wada-gawa), arrows indicate internal puparia **I** cross section of mined thallus of *C.orientalis* at Nishiyama-onsen.

**Female** (Figs 59F, 60C). Similar to male, but slightly larger; color of scutellum darker. Wing length 2.3 mm. ***Postabdomen*:** (Fig. 60A, B) Oviscape dark brown, setigerous (Fig. 60A). Tergite 10 cruciform, laterally uniting narrow pleural sclerites (Fig. 60B). Each cercus with two stout, apical, trichoid sensilla, 1/3 length of cercus (Fig. 60B). Spermathecae semi-orbicular, with truncate proximal ends (Fig. 60A).

#### Variation.

The color pattern of the scutellum varied among localities; individuals at some localities had a larger dark corner on the scutum. The number of tubercle-like setae in a comb of the male epandrium varied from 5 to 6 among localities, among individuals in the same locality and even between right and left combs of an individual. The number of tubercle-like setae on the surstylus was consistently two, but the direction of each varied among localities.

#### Etymology.

 The specific name refers to the larval feeding on *Conocephalum* liverworts.

#### Japanese name.

Komorebi-jagoke-hamoguribae.

#### Host plants.

*Conocephalumsalebrosum*, *C.orientalis*, *C.purpureorubrum* and *C.japonicum* (Conocephalaceae).

#### Mine.

 Larvae construct linear mines in the midrib of the thallus, and pupate in the mines (Fig. 60D–I). The mines on thick thalli of perennial *C.orientalis* are inconspicuous (Fig. D), while those on thin thalli of annual *C.japonicum* (Fig. E–I) are blackish and conspicuous.

#### Biological notes.

 The habitats of this species are stream banks and mesic slopes in warm temperate evergreen forests dominated by *Castanopsiscuspidata* and cool temperate deciduous forests dominated by *Quercuscrispula*. It is sympatric with *P.izayoi*,*P.luteola*, and *P.lanternaria* at some localities. Our rearing records suggest that this species is bivoltine, with adults emerging twice in spring and summer.

#### Distribution.

**Figure 61.**
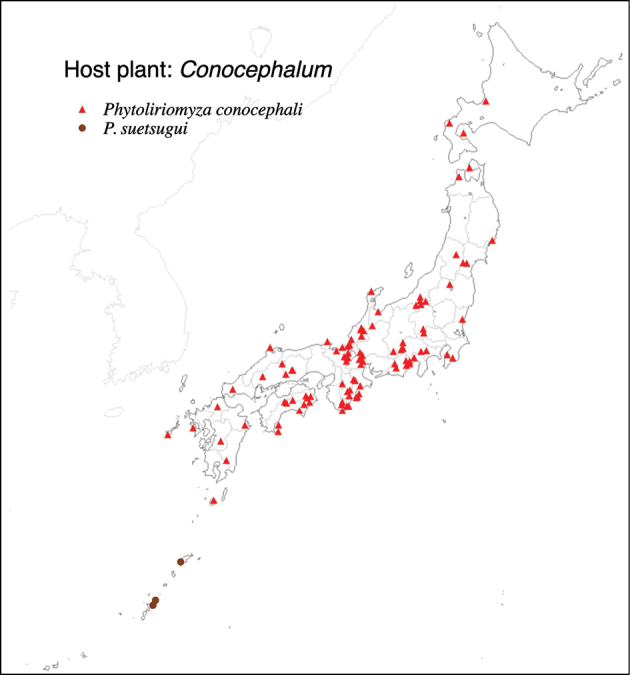
Locality records of three *Phytoliriomyza* species associated with *Conocephalum* spp.: *P.conocephali*and *P.suetsugui*.

Japan: Hokkaido, Honshu, Shikoku, Kyushu, and Yaku Island (Fig. 61).

#### Remarks.

 This species is the second smallest (next to *P.suetsugui*) among the *Phytoliriomyza* species associated with *Conocephalum*, and is the only species that mines the small thalli of *C.japonicum*. This species resembles *P.marchantiae*,*P.rebouliae*, and *P.lanternaria* in having a small yellow mark lying between the posterior scutum and the scutellum; it is distinguished from *P.marchantiae* and *P.rebouliae* by the presence of tubercle-like seta on the surstylus of the male epandrium, from *P.lanternaria* by the number of tubercle-like setae in a comb of the male epandrium (5–6 in *P.conocephali*; 7 in *P.lanternaria*).

This species also resembles *P.miki* and *P.fumicosta* in scutum color and male genitalia; it is distinguished from *P.miki* by the rounded surstylus (slender and elongated in *P.miki*), from *P.fumicosta* by the number of fused tubercle-like setae in a comb of the male epandrium (5–6 in *P.conocephali*; 7 in *P.fumicosta*).

### ﻿33.﻿ Phytoliriomyza suetsugui

Taxon classificationAnimaliaDipteraAgromyzidae

Kato sp. nov.

96B78587-0248-5718-9A8F-EB676DE689CC

https://zoobank.org/C6207F26-047E-4F73-B42B-B92CB9291F39

Fig. 62

#### Material examined.

***Holotype*:** Japan: 1♂ (MK-AG-a221), Arakawa, Takae, Higashi-son, Okinawa Pref. (26.6655°N, 128.2542°E, 45 m asl), 22-II-2011 (as larva on *Conocephalumorientalis* collected by K. Suetsugu), emerged on 16-IV-2011, NSMT-I-Dip 32085. ***Paratypes*:** Japan: 2♂1♀ (MK-AG-a433, a434, 698), same data as holotype emerged on 14–22-IV-2011, NSMT-I-Dip 32086–32088; 1♀ (MK-AG-766), Naon, Yamato, Oshima, Kagoshima Pref., 12-XII-2014 (as larva), emerged on 17-III-2015, NSMT-I-Dip 32089; 1♀ (MK-AG-761), Mt. Yonaha, Kunigami, Okinawa Pref., 18-VII-2016 (as larva on *C.orientalis*), emerged on 14-X-2016, NSMT-I-Dip 32090.

#### Other material.

 Japan: 3♂3♀, Arakawa, higashi-son, Okinawa Pref., 10-XI-2021 (as larva on *C.orientalis*), emerged on 27-I–12-II-2022.

#### Diagnosis.

 A small dark species (wing length 1.3–1.5 mm) having a pruinose dark gray scutum and scutellum, a black 1^st^ flagellomere, dark maxillary palpus, dark halteres, and brown legs. Male epandrium inner-laterally with a tubercle like seta, and inner-basally with a comb comprising six long fused tubercle-like setae. Larva mines the thallus of *Conocephalumorientalis*.

#### Description.

**Figure 62.**
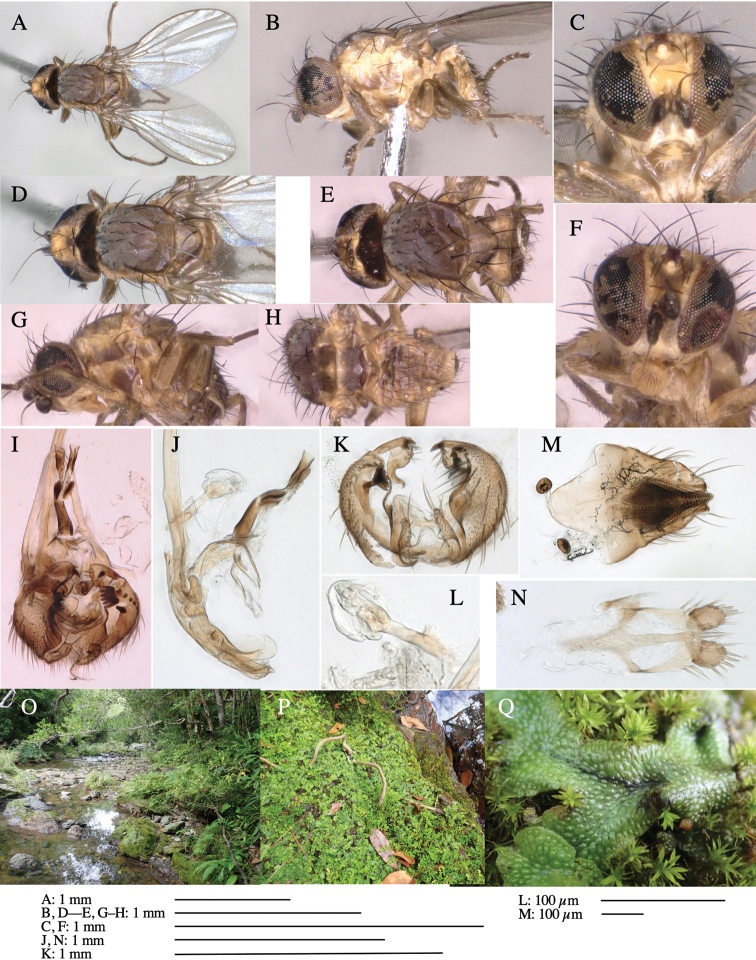
*Phytoliriomyzasuetsugui*sp. nov. **A–D **holotype male **A** habitus **B** lateral **C** frontal **D** dorsal **E–H** paratype female (MK-AG-a434) **E** dorsal **F** frontal **G** lateral **H** posterior **I–L** male genitalia **I** whole genitalia, ventral **J** phallic complex, lateral **K** epandrium, ventral **L** ejaculatory apodeme **M, N** female postabdomen **M** oviscape and spermatheca **N** tergite 10 **O, P** habitat at type locality **Q** mined thallus of *Conocephalumorientalis*.

**Adult male** (Fig. 62A–D).

***Head*:** Head light yellow, with ocellar tubercle brown, back of head dark brown (Fig. 62C). Antenna porrect, first flagellomere dark brown, pedicel and scape brown (Fig. 62B). Arista subbasal, black, pubescent. Clypeus, face, gena, parafacial and postgena yellow. Proboscis normal, yellow; palpus brown, cylindrical (Fig. 62C). ***Chaetotaxy*:** Front orbitals three pairs; one ori directed inward; two ors directed upward (Fig. 62B). Orbital setulae minute and erect, in a single row.

***Thorax*:** Thorax pruinose. Scutum and scutellum pruinose gray (Fig. 62D). Subscutellum light yellow except for brown posterior half. Mediotergite and anatergite brown, katatergite light yellow (Fig. 62B). Pleuron yellow light yellow with brownish patches on venter of propleuron, anepisternum, katepisternum, anepimeron, and meron. Haltere brown. Calypter margin and hairs gray. Leg segments brownish, basal half of femur paler (Fig. 62A). ***Chaetotaxy*:** Scutum with 1+3 dorsocentrals, shortened anteriorly (Fig. 62D). Acrostichal setulae five or six pairs in two irregular rows. ***Wing*:** Wing length 1.3 mm, costa reaching M_1_ (Fig. 62A). Length of ultimate section of vein M_4_ divided by penultimate section 1.3.

***Abdomen*:** Abdomen dorsally subshiny brown; epandrium dark brown (Fig. 62B). ***Genitalia*:** (Fig. 62I–L) Epandrium rounded apically; inner-lateral surface with a long tubercle-like seta; inner-anterior surface with a comb comprising six fused long tubercle-like setae; inner-basal margin with a row of six or seven small tubercle-like setae (Fig. 62I, K). Surstylus rounded, curved inwards, setose apically, with one long tubercle-like seta on posterior subdistal margin (Fig. 62K). Cercus narrow, setose. Subepandrial sclerite H-shaped. Hypandrium slightly sclerotized along outer margin (Fig. 62I). Postgonite bare, goose barnacle-shaped, rounded apically (Fig. 62I). Phallophorus with deep incision below, articulated with phallapodeme, fused to epiphallus (Fig. 62J). Basiphallus with a plate-like sclerite on left side (Fig. 62J). Hypophallus broad, membranous covered with microtrichia ventrally; lateral margins lightly sclerotized; medially with a pair of fused, narrow ventrally incurved sclerites; laterally with a pair of converging narrow sclerites (Fig. 62J). Paraphallus pale membranous; paraphalli spread laterally, jointed basally (Fig. 62J). Mesophallus dark, cylindrical, widest subbasally, 4/5 as long as distiphallus (Fig. 62J). Distiphallus comprising one pair of stout tubules basally parallel to each other; covered by a transparent membrane with a pair of 7 oval minute sclerites; basal half composed of ventral dark subtriangular sclerite and weaker medial region; distal half cylindrical, dorsally pigmented, widening toward truncated unpigmented apex (Fig. 62J). Ejaculatory apodeme pale brown, with fan-shaped blade and long stalk; base wide to one side; sperm pump clear (Fig. 62L).

**Female** (Fig. 62E–H). Similar to male, but slightly larger; frons and pleuron darker. Wing length 1.5 mm. ***Postabdomen*:** (Fig. 62M, N) Oviscape dark brown, setigerous (Fig. 62M). Tergite 10 cruciform, laterally uniting narrow pleural sclerites (Fig. 62N). Each cercus with two stout, apical, trichoid sensilla, ½ length of cercus (Fig. 62N). Spermathecae semi-orbicular, with truncate proximal ends (Fig. 62M).

#### Etymology.

 The specific name honors a botanist, Dr. Kenji Suetsugu, who collected thalli of *Conocephalumorientalis*. at the type locality.

#### Japanese name.

Yanbaru-jagoke-hamoguribae.

#### Host plant.

*Conocephalumorientalis*.

#### Mine.

(Fig. 62Q) Larvae construct linear mines in the midrib of the thallus, and pupate in the mines.

#### Biological notes.

 The habitats of this species are stream banks in warm temperate evergreen forests dominated by *Castanopsissieboldii* (Fig. 62O, P).

#### Distribution.

Japan: Amami and Okinawa Islands (Fig. 61).

#### Remarks.

 This species resembles *P.ricciae* in having a wholly dark scutum, yellow pedicel and scape of the antenna, and dark maxillary palpus; it is distinguished from the latter by the presence of a comb of tubercle-like setae on the male epandrium. This species also resembles *P.marchantiae*,*P.rebouliae*,*P.lanternaria*, and *P.conocephali* in having dark scutum and a comb comprising 5–8 tubercle-like setae on the male epandrium; it is distinguished from them by lacking a small yellow mark on both the scutellum and the posterior margin of the scutum.

##### Species associated with *Riccia*

### ﻿34.﻿ Phytoliriomyza ricciae

Taxon classificationAnimaliaDipteraAgromyzidae

Kato sp. nov.

8FE0ADBF-9528-5890-9362-C5B8DBB3E5B7

https://zoobank.org/E56E7ECF-0CC4-4B0E-A38A-4FE2FF875C98

Figs 63 , 64

#### Material examined.

***Holotype*:** Japan: 1♂ (MK-AG-a416), Iwakura-muramatsu, Sakyo-ku, Kyoto Pref. (35.0931°N, 135.7900°E, 150 m asl), 12-XI-2020 (as larva on *Ricciahuebeneriana*), emerged on 1-XII-2020, NSMT-I-Dip 32091. ***Paratypes*:** Japan: 1♂1♀ (MK-AG-a477, a478), same data as holotype, emerged on 2–16-XII-2020, NSMT-I-Dip 32092–32093; 1♀ (MK-AG-201), type locality, 27-X-2017 (as larva on *R.huebeneriana*), emerged on 13-XI-2017, NSMT-I-Dip 32094; 1♀ (MK-AG-205), Muramatsu, Iwakura, Sakyo-ku, Kyoto Pref., 16-XI-2017 (as larva on *R.nipponica*), emerged on 8-XII-2017, NSMT-I-Dip 32095; 1♀ (MK-AG-234), Niken-chaya, Shizuichi-ichihara, Sakyo, Kyoto Pref., 18-XII-2015 (as larva on *R.nipponica*), emerged on 15-IV-2016, NSMT-I-Dip 32096;1♂ (MK-AG-a440), Nienami, Nango, Nichinan, Miyazaki Pref., 25-IX-2017 (as larva on *R.canaliculata*), emerged on 29-X-2017, NSMT-I-Dip 32097.

#### Other material.

 Japan: On *R.nipponica*: 39♂40♀, Niken-chaya, Shizuichi-ichihara, Sakyo, Kyoto Pref., 31-X-2015 (as larva), emerged on 25-XI-2015–8-IV-2016; 1♀, Midai-gawa. Tatsuoka, Nirasaki, Yamanashi Prec., 10-XII-2016 (as larva), emerged on 14-V-2017.

On *R.miyakeana*: 1♀, Muramatsu, Iwakura, Sakyo-ku, Kyoto Pref., 16-XI-2017 (as larva), emerged on 10-XII-2017.

On *R.lamellosa*: 1♂, Joja, Joso, Ibaragi Pref., 2-XI-2021 (as larva), emerged on 3-XII-2021.

On *R.oryzicola*: 1♂, Somada, Wazuka, Soraku, Kyoto Pref., 15-X-2021 (as larva), emerged on 15-XI-2021; 1♀, Megami, Makinohara, Shizuoka Pref., 20-X-2017 (as larva), emerged on 3-I-2018; 1♀, Aono-gawa, Sanda, Hyogo Pref., 30-X-2017 (as larva), emerged on 26-XI-2017.

On *R.bifurca*: 1♂, Inago, Shibakawa, Fujinomiya, Shizuoka Pref., 10-X-2021 (as larva), emerged on 21-X-2021.

On *R.huebeneriana*: 2♀, Muramatsu, Iwakura, Sakyo-ku, Kyoto Pref., 16-X-2017 (as larva), emerged on 8–18-XII-2017; 1♂1♀, Mt. Osuzu, Tsuno, Miyazaki Pref., 24-IX-2017 (as larva), emerged on 15-X-2017; 1♂1♀, Urauchi, Iriomote-Is. Yaeyama, Okinawa Pref., 9-XI-2021 (as larva), emerged on 30-XI-2021; 1♂, Nametoko, Matsuno, Kita-uwa, Ehime Pref., 3-X-2021 (as larva), emerged on 20-X-2021; 3♀, Kayo, Nago, Okinawa Pref., 10-XI-2021 (as larva), emerged on 22–28-XI-2021.

On *R.canaliculata*: 3♂, Nienami, Nango, Nichinan, Miyazaki Pref., 25-IX-2017 (as larva), emerged on 17–29-IX-2017.

#### Diagnosis.

 A small species (wing length 1.0–1.3 mm) having a pruinose grayish yellow scutum with a medial and two pairs of lateral dark gray stripes, a gray scutellum, yellow pleuron, black 1^st^ flagellomere, dark maxillary palpus, yellowish gray halteres, and yellow legs. Male epandrium with dorso-ventrally bilobed surstylus; dorsal arm with two short tubercle-like setae. Male epandrium with bilobed, dorsoventrally elongated surstyli. Distiphalli tapering toward apex and bilaterally asymmetrical. Larva mines the thallus of *Riccia* spp.

#### Description.

**Figure 63.**
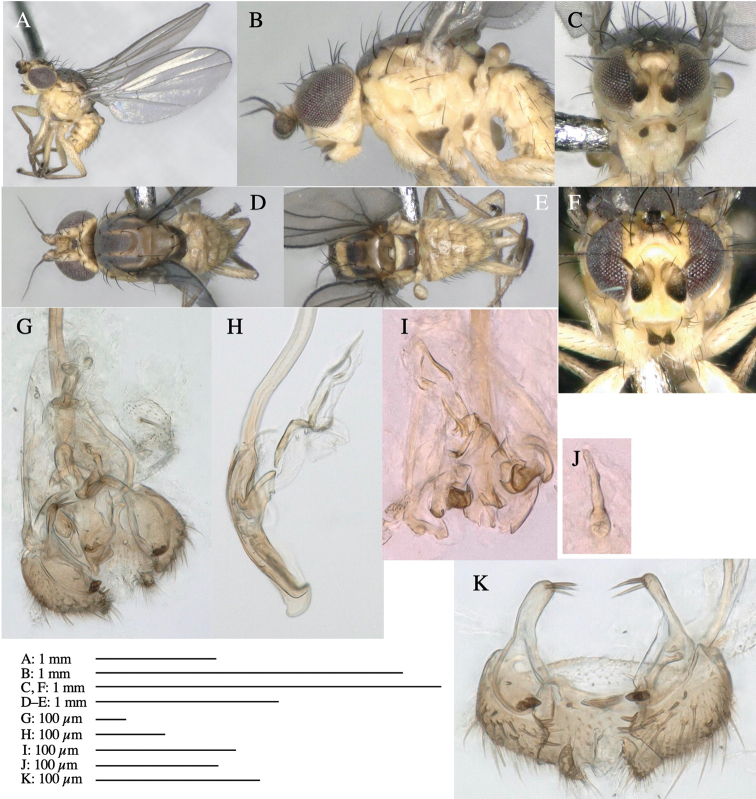
*Phytoliriomyzaricciae*sp. nov. **A–E **holotype male **A** habitus **B** lateral **C** frontal **D** dorsal **E** posterior **F** paratype female, frontal (MK-AG-201) **G–K** male genitalia (**G–J** on *Riccianipponica***K** on *R.huebeneriana*) **G** whole genitalia, ventral **H** phallic complex, lateral **I** phallic complex, ventral **L** ejaculatory apodeme, lateral **M** epandrium, ventral.

**Adult male** (Fig. 63A–E).

***Head*:** Head entirely light yellow, with ocellar tubercle dark brown, and back of head dark brown (Fig. 63C). Antenna porrect; first flagellomere dark brown with arista base yellowish, pedicel and scape grayish yellow (Fig. 63B). Arista subbasal, brown, pubescent. Frons brownish yellow, with reflective pruinosity. Face, gena, parafacial and postgena yellow. Proboscis normal, yellow; palpus dark brown, dorso-ventrally flattened, spatula-shaped (Fig. 63C). ***Chaetotaxy*:** Front orbitals three pairs; one ori directed inward; two ors directed upward (Fig. 63B). Orbital setulae minute and proclinate, in a single row.

***Thorax*:** Thorax pruinose light yellow. Scutum grayish yellow with medial blackish stripe on anterior 2/3, one pair of blackish suborbicular presutural spots confluent with the medial blackish stripe, a pair of narrow blackish supra-alar stripes, and a pair of wider blackish intra-alar stripes, which adjoin the pair of lateral presutural suborbicular spots (Fig. 63D). The background color of the scutum is paler in live than in dried specimens. Scutellum gray. Subscutellum yellow except brown posterior half. Mediotergite dark gray, anatergite and katatergite light yellow (Fig. 63E). Pleuron largely light yellow; postpronotal lobe with anterior brown spot; propleuron with a small brown patch on mid-anterior corner; anepisternum and anepimeron light yellow; katepisternum and meron with large brown patches on venter (Fig. 63B). Haltere yellowish gray, with stalk light yellow. Calypter margin and hairs gray. Leg segments yellow; tibia and tarsus darker (Fig. 63A). ***Chaetotaxy*:** Scutum with 1+3 dorsocentrals, shortened anteriorly (Fig. 63D). Acrostichal setulae almost absent or with one (or rarely two) pair of minute setae. ***Wing*:** Wing length 1.4 mm, costa reaching M_1_ (Fig. 63A). M_4_ disappears immediately before reaching wing margin. Length of ultimate section of vein M_4_ divided by penultimate section 2.2–2.4.

***Abdomen*:** Abdomen dorsally subshiny grayish yellow; epandrium dark brown (Fig. 63E). ***Genitalia*:** (Fig. 63G–K) Epandrium rounded apically; posterior end of inner margin with two or three short tubercle-like setae directed ventrally (Fig. 63G, K). Surstylus dorso-ventrally bilobed; dorsal lobe broad with two bulbous tubercle-like setae apically; ventral lobe narrow with three or four long setae apically (Fig. 63K). Cercus narrow, setose. Subepandrial sclerite pale, plate-like (Fig. 63K). Hypandrium thin, slightly sclerotized along outer margin (Fig. 63G). Postgonite bare, goose barnacle-shaped, rounded apically (Fig. 63I). Phallophorus with shallow incision below, articulated with phallapodeme, fused to epiphallus (Fig. 63H, I). Basiphallus dorsally sclerotized, with a pale narrow sclerite on left side (Fig. 63I). Hypophallus membranous and trilobed, with a pair of small dark lobate sclerites basally (Fig. 63H). Mesophallus pale cylindrical and laterally pigmented (Fig. 63H). Distiphallus comprising one pair of bilaterally asymmetrical tubules; tubule tapering toward apex; right one shorter and thicker than left one (Fig. 63H, I). Ejaculatory apodeme pale brown, fan-shaped with short stalk and clear sperm pump (Fig. 63J).

**Figure 64.**
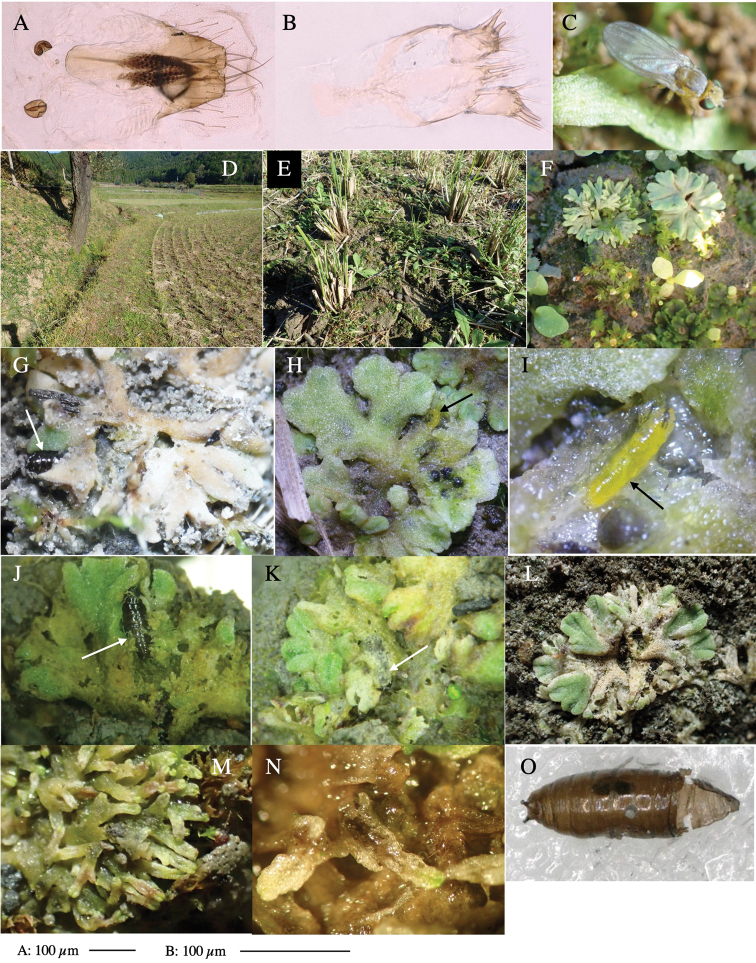
Female morphology and larval ecology of *Phytoliriomyzaricciae*sp. nov. **A, B **female postabdomen **A** oviscape and spermatheca **B** tergite 10 **C** an adult female fly on thallus of *Ricciahuebeneriana*at Kayo **D–F** landscape of harvested rice fields at type locality **F** thalli of *R.huebeneriana* (left) and *R.oryzicola* (right) growing on soil **G–N** mined thalli (**G***R.miyakeana* at type locality **H, I***R.nipponica* at type locality **J, K***R.oryzicola* at type locality **L***R.oryzicola* at Wazuka **M***R.huebeneriana*at type locality **N***R.canaliculata* at Nienami) **O** puparium. Arrows in **G, J, L** indicate internal puparia.

**Female** (Fig. 63F). Similar to male, but slightly larger and frons wider. Wing length 1.4 mm. ***Postabdomen*:** (Fig. 64A, B) Oviscape dark brown, setigerous (Fig. 64A). Tergite 10 cruciform, laterally uniting narrow pleural sclerites (Fig. 64B). Each cercus with two stout, apical, trichoid sensilla, 1/2 length of cercus (Fig. 64B). Spermathecae semi-orbicular, with truncate proximal ends (Fig. 64A).

#### Variation.

Geographical variation was found in the presence of an acrostichal seta on the scutum; the seta was almost absent in individuals at most localities but present in those from the Okinawa Island.

**Immatures.** (Fig. 64G–K, O) Puparium internal, slender and dark brown (Fig. 64O).

#### Etymology.

 The specific name refers to the host plant genus *Riccia*.

#### Japanese name.

Yosame-hatakegoke-hamoguribae.

#### Host plants.

*Riccianipponica* (Fis. 64G), *R.miyakeana* (Fig. 64H, I), *R.oryzicola* (Fig. 64J–L), *R.bifurca*,*R.lamellosa*,*R.huebeneriana* (Fig. 64M) and *R.canaliculata* (Fig. 64N) (Ricciaceae). Although the European species, *P.mesnili* has been recorded from *Riccia* and *Ricciocarpos*, *P.ricciae* has been recorded only from *Riccia* spp., even though *Ricciocarposnatans* was abundant in the same rice fields.

#### Mine.

(Fig. 64G–N) Larvae constructed linear-blotch mines in the thallus from autumn to winter, and pupated inside or outside the mines. This variation in pupation site is due to the thalli of the host plants being small and thin; only the surface layer of the thalli remained intact, with internal parenchymatous tissues largely consumed. The larvae sometimes transfer to neighboring/overlapping thalli and fed on them.

#### Biological notes.

 The host liverwort species, *R.nipponica*,*R.miyakeana*,*R.oryzicola* and *R.huebeneriana* grow only in the paddy fields that have not experienced land improvement projects or spraying herbicides (Fig. 64D–F). Thus, *P.ricciae* is now rare and threatened due to decrease of its potential habitats, and additionally due to recent overuse of insecticides. Because the host liverworts appear in paddy fields only after harvest of rice in autumn, it is unknown how the species pass other seasons from spring to autumn. The rice fields applied with organic farming also harbors various hornwort species, and harbor hornwort-associated species, *P.phaeocerotis*.

#### Distribution.

**Figure 65.**
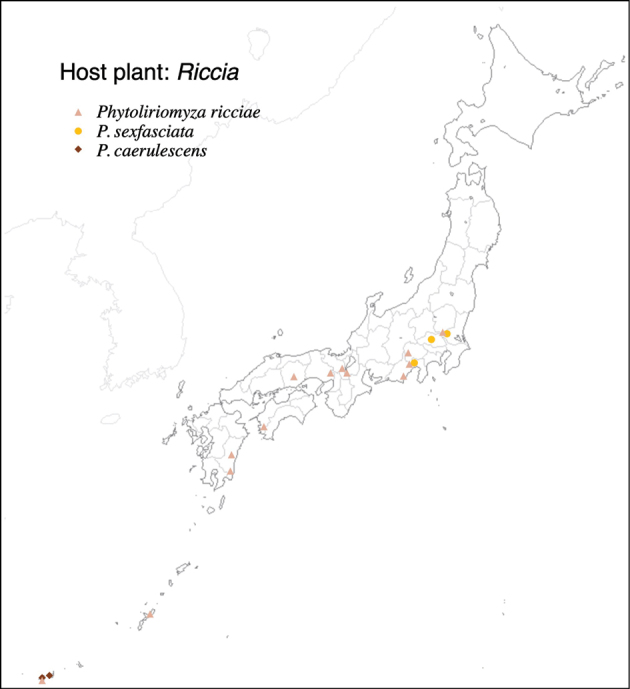
Locality records of three *Phytoliriomyza* species associated with *Riccia* spp.: *P.ricciae*, *P.sexfasciata*and *P.caerulescens*.

Japan: Honshu, Shikoku, Kyushu, Okinawa Is. and Iriomote Is. (Fig. 65).

#### Remarks.

This species resembles an European *Ricciocarpos*/*Riccia*-associated species, *P.mesnili*; it is distinguished from the latter by the dark color of scutum and scutellum (paler in *P.mesnili*), the obscure dark lateral bands on scutum (more distinct in *P.mesnili*), the vestigial acrostichal seta (almost absent in *P.ricciae*; with four pairs of acrostichal setae in *P.mesnili*), the small hypophallus (developed and sclerotized in *P.mesnili*; hypophallus is reported as paraphallus in Sellier 1947).

This species is also closely related to another European species, *P.venustula* Spencer (host unknown); it is distinguished from the latter by the vestigial acrostichal setae on the scutum (present in *P.venustula*), weaker sclerotization of male genitalia (well sclerotized in *P.venustula*), number of apical long setae on the ventral lobe of the surstylus (3–4 in *P.ricciae*; 6 in *P.venustula*), and the small but distinct hypophallus (hypophallus lacking in *P.venustula*).

Among the Japanese species, *P.ricciae* resembles *P.suetsugui*,*P.sexfasciata*, and *P.megacerotis* in having wholly dark scutum and dark maxillary palpus; it is distinguished from them by the absence of a comb of tubercle-like setae on the male epandrium. This species also resembles *P.ugetsu*,*P.caerulescens*, and *P.phaeocerotis* in having a wholly dark scutum; it is distinguished from them by the color of maxillary pulps (dark in *P.ricciae*; yellow in the others).

### ﻿35.﻿ Phytoliriomyza sexfasciata

Taxon classificationAnimaliaDipteraAgromyzidae

Kato sp. nov.

65D8B741-B29D-5499-93F1-55905D51DA07

https://zoobank.org/23B90D2D-BB82-494F-94B2-EA5703FDC06B

Fig. 66 , 67

#### Material examined.

***Holotype*:** Japan: 1♂ (MK-AG-a574), Ookura, Arashiyama, Hiki, Saitama Pref. (36.0276°N,139.3284°E, 50 m asl), 2-XI-2021 (as larva on *Riccialamellosa*), emerged on 25-XI-2020, NSMT-I-Dip 32098. ***Paratypes*:** Japan: 1♂2♀ (MK-AG-a573, a583, a584), same data as holotype, emerged on 17–25-XI-2021, NSMT-I-Dip 32099–32101; 1♀ (MK-AG-a568), Joja, Joso, Ibaragi Pref., 2-XI-2021 (as larva on *Riccialamellosa*), emerged on 19-XI-2021, NSMT-I-Dip 32102; 1♂ (MK-AG-a585), Joja, Joso, Ibaragi Pref., 10-X-2021 (as larva on *Ricciabifurca*), emerged on 8-XI-2021, NSMT-I-Dip 32103.

#### Other material.

 Japan; On *R.lamellosa*:11♂14♀, Negishi, Arashiyama, Hiki, Saitama Pref., 2-XI-2021 (as larva), emerged on 17-XI–12-XII-2021; 3♂5♀, Joja, Joso, Ibaragi Pref., 2-XI-2021 (as larva, emerged on 17–25-XI-2021.

On *R.bifurca*: 7♂9♀, Joja, Joso, Ibaragi Pref., 2-XI-2021 (as larva), emerged on 8–29-XI-2021.

On *R.sorocarpa*: 3♂3♀, Joja, Joso, Ibaragi Pref., 2-XI-2021 (as larva), emerged on 22-XI–26-XII-2021.

#### Diagnosis.

A small species (wing length 1.2–1.5 mm) having a pruinose grayish scutum with six longitudinal dark gray bands, a gray scutellum, brown 1^st^ flagellomere, brown maxillary palpus, yellowish gray halteres, and yellow legs. Male epandrium inner-distally with a strong tubercle-like seta and inner-basally with a cluster of 29–35 dense tubercle-like setae. Distiphalli bilaterally asymmetrical, with left one tapering toward apex. Larva mines the thallus of *Riccialamellosa*,*R.sorocarpa* and *R.bifurca*.

#### Description.

Adult male.

**Figure 66.**
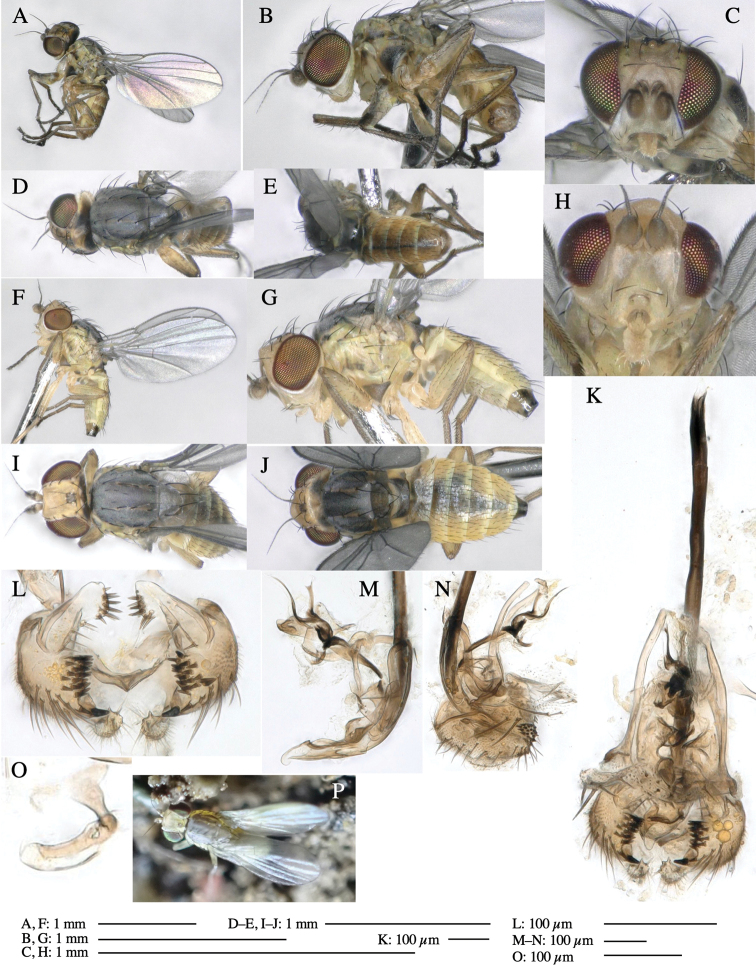
*Phytoliriomyzasexfasciata*sp. nov. **A–E **holotype male **A** habitus **B** lateral **C** frontal **D** dorsal **E** posterior **F–J** paratype female (MK-AG-a573) **F** habitus **F** lateral **G** frontal **H** dorsal **I** posterior **K–O** male genitalia **K** whole genitalia, ventral **L** epandrium, ventral **M** phallic complex, lateral **N** genitalia, lateral **O** ejaculatory apodeme, lateral **P** live female fly walking on soil.

***Head*:** (Fig. 66A–E) Head largely light yellow, with ocellar tubercle dark brown, and back of head dark brown (Fig. 66C). Antenna porrect; first flagellomere dark brown, pedicel and scape brown (Fig. 66B). Arista subbasal, brown, pubescent. Frons brownish yellow, with reflective pruinosity (Fig. 66C). Face, gena, parafacial and postgena light yellow. Proboscis normal, light yellow; maxillary palpus light yellow, cylindrical (Fig. 66C). ***Chaetotaxy*:** Front orbitals three pairs; one ori directed inward; two ors directed upward (Fig. 66B). Orbital setulae minute and proclinate, in a single row.

***Thorax*:** Thorax pruinose gray. Scutum gray with three pairs of longitudinal dark gray stripes, with the four inner stripes continuing into dark gray bands of gray scutellum (Fig. 66D). Subscutellum light yellow except brown posterior half. Mediotergite dark gray, anatergite and katatergite light yellow (Fig. 66E). Pleuron largely light yellow; postpronotal lobe with anterior brown spot; propleuron, light yellow; anepisternum light yellow with L-shaped anterior brown spot; anepimeron light yellow with an upper linear macule and a lower spot; katepisternum and meron with large brown patches on venter (Fig. 66C). Haltere yellowish gray, with stalk light yellow. Calypter margin and hairs gray. Leg segments brown; tibia and tarsus darker; distal half of femur of foreleg darkened on frontal side (Fig. 66A). ***Chaetotaxy*:** Scutum with 1+3 dorsocentrals, shortened anteriorly (Fig. 66D). Acrostichal setae 1–3 pairs in two rows. ***Wing*:** Wing length 1.2 mm, costa reaching M_1_ (Fig. 66A). Length of ultimate section of vein M_4_ divided by penultimate section 2.0–2.3.

***Abdomen*:** Abdomen dorsally subshiny brownish yellow, with a medial brown longitudinal band; epandrium brown (Fig. 66E). ***Genitalia*:** (Fig. 66K–O) Epandrium rounded apically; posterior end of inner margin with two tubercle-like setae, the dorsal one stouter and longer; inner-lateral surface with a dense cluster of 29–35 short tubercle-like setae (Fig. 66L). Surstylus narrow and elongated, with 12–14 strong setae apically (Fig. 66L). Cercus narrow, setose. Subepandrial sclerite pale, plate-like (Fig. 66L). Hypandrium thin, slightly sclerotized along outer margin (Fig. 66K). Postgonite bare, goose barnacle-shaped, cleft apically (Fig. 66M). Phallophorus with shallow incision below, articulated with phallapodeme, fused to epiphallus (Fig. 66K). Basiphallus with a dark dorsal narrow sclerite, the distal lobes of which extended laterally; 2–2.5 × as long as mesophallus (Fig. 66M, N). Hypophallus broad and membranous, medially with a pair of dark, fused, ventrally incurved sclerites (Fig. 66M). Mesophallus with a pair of short basal, ventral sclerites and a pair of dark, long, ventral sclerite (Fig. 66M). Distiphallus comprising one pair of bilaterally asymmetrical dark tubules; left tubule longer than the right one, dorsally pigmented, tapering out; right tubule dorsally pigmented, truncated distally and bifid (Fig. 66M, N). Ejaculatory apodeme pale brown and fan-shaped with constricted stalk; base dark and elongated to one side; sperm pump sausage-shaped, clear (Fig. 66O).

**Figure 67.**
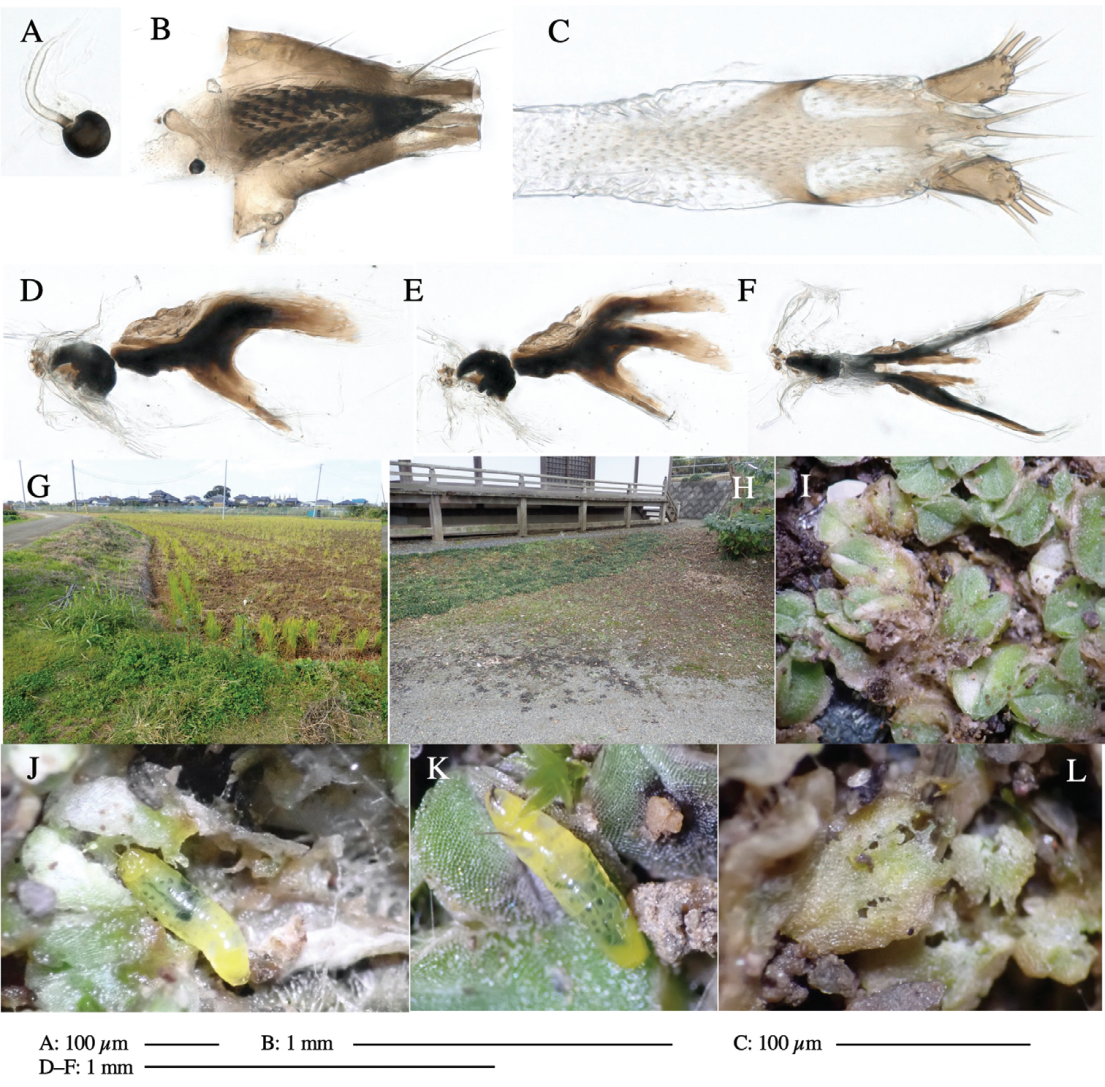
Female morphology and larval morphology/ecology of *Phytoliriomyzasexfasciata*sp. nov. **A–C **female postabdomen **A** spermatheca **B** oviscape and spermatheca **C** tergite 10 **D–F** Pharyngeal skeleton of 3^rd^ larva **D** lateral **E** dorso-lateral **F** dorsal **G, H** habitat (**G** at Joso **H** at type locality) **I–L** mined thalli and mining larvae (**I–K***Riccia lamellose *at type locality **L***R.bifurca* at Joso).

**Female** (Fig. 66F–J, P). Similar to male, but slightly larger and frons wider; color of pleuron, hind abdomen and legs much paler. Wing length 1.5 mm. ***Postabdomen*:** (Fig. 67A–C) Oviscape dark brown, setigerous (Fig. 67B). Tergite 10 cruciform, laterally uniting narrow pleural sclerites (Fig. 67C). Each cercus with two stout, apical, trichoid sensilla, 1/2 length of cercus (Fig. 67C). Spermathecae semi-orbicular, with truncate proximal ends (Fig. 67A).

**Immatures.** (Fig. 67D–F, N, O) At 3^rd^ instar, larval body yellow and cylindrical. Mandibles paired, fused, asymmetrical and strongly sclerotized, each with two teeth; left teeth larger than right ones. Mandibles confluent with short sclerotized hypopharyngeal sclerite, connecting to a pair of sclerotized tentoropharyngeal sclerites; dorsal cornu broad and long, ventral cornu shorter, comprising two fused arms; upper arm dark sclerotized only in anterior half.

#### Etymology.

 The specific name (*sex* = six, *fasciatus* = stripe) refers to the six dark gray stripes on the scutum.

#### Japanese name.

Mutsusuji-hatakegoke-hamoguribae.

#### Host plants.

*Riccialamellosa*, *R.bifurca*, *and R.sorocarpa*.

#### Mine.

 Larvae construct linear-blotch mines in the thallus, and pupate in or out of the mines (Fig. 67I–L). Larvae sometimes relocate to fresh thalli as with *P.ricciae*.

#### Biological notes.

 The host liverwort species, *Riccialamellosa*, *R.bifurca*, and*R.sorocarpa* grow on bare mesic soil in orchards, parks, shrines and levees of paddy fields (Fig. 67G, H). While *R.lamellosa* is a recently naturalized alien species (Furuki 2000), the other hosts are native. Larvae of *P.sexfasciata* are found mining thalli of these liverwort species in early November, and adults emerge in late November.

#### Distribution.

Japan: Honshu (Fig. 65). So far, recorded only from alluvial plains in the Kanto Region.

#### Remarks.

This species is unique in that the scutum is six-banded (medial bands on the scutum are confluent in other species), and in the male epandrium having a clump (not a comb) of 25–30 short tubercle-like setae on the basal margin. This species resembles *P.suetsugui*,*P.ricciae* and *P.megacerotis* in having a wholly dark scutum and dark maxillary palpus, but is distinguished from them by the above-mentioned genital characteristics. It also resembles *P.ugetsu*,*P.caerulescens* and *P.phaeocerotis* in having wholly dark scutum, but is distinguished from them by the color of the maxillary palpus (dark in *P.sexfasciata*; yellow in the others).

### ﻿36.﻿ Phytoliriomyza caerulescens

Taxon classificationAnimaliaDipteraAgromyzidae

Kato sp. nov.

C42A1498-7562-5367-A225-1AA98BE82238

https://zoobank.org/7DA0D0D4-177C-474E-BFC2-7E0F1F180508

Figs 68 , 69

#### Material examined.

***Holotype*:** Japan: 1♂ (MK-AG-a562), Ugan-zaki, Ishigaki-Is. Yaeyama, Okinawa Pref. (24.4479°N, 124.0826°E, 10 m asl), 7-XI-2021 (as larva on *Ricciabillardieri*), emerged on 19-XI-2021, NSMT-I-Dip 32104. ***Paratypes*:** Japan: 1♀ (MK-AG-a558), same data as holotype, NSMT-I-Dip 32105; 1♂2♀ (MK-AG-a589, a559, a590), Komi, Iriomote-Is. Yaeyama, Okinawa Pref., 9-XI-2021 (as larva on *Ricciabillardieri*), emerged on 17-XI–8-XII-2021, NSMT-I-Dip 32106–32108; 1♂ (MK-AG-a576), Urauchi, Iriomote-Is. Yaeyama, Okinawa Pref., 8-XI-2021 (as larva on *Ricciahuebeneriana*), emerged on 25-XI-2021, NSMT-I-Dip 32109.

#### Other material.

 Japan: On *R.billardieri*: 65♂70♀, same data as holotype, 7-XI-2021 (as larva), emerged on 19-XI–5-XII-2021; 85♂82♀, Komi, Iriomote-Is. Yaeyama, Okinawa Pref., 9-XI -2021 (as larva), emerged on 22-XI–811-XII-2021; 18♂25♀, Urauchi, Iriomote-Is. Yaeyama, Okinawa Pref., 9-XI -2021 (as larva), emerged on 22-XI–811-XII-2021.

On *R.huebeneriana*: 2♂3♀, Ugan-zaki, Ishigaki-Is. Yaeyama, Okinawa Pref. 7-XI-2021 (as larva), emerged on 22–24-XI-2021; 10♂4♀, Komi, Iriomote-Is. Yaeyama, Okinawa Pref., 9-XI-2021 (as larva), emerged on 16–29-XI-2021; 13♂10♀, Urauchi, Iriomote-Is. Yaeyama, Okinawa Pref., 9-XI-2021 (as larva), emerged on 27-XI–15-XII-2021.

#### Diagnosis.

 A small species (wing length 1.1–1.3 mm) having a pruinose gray scutum and scutellum, brown 1^st^ flagellomere, yellow maxillary palpus, gray halteres, and yellowish brown legs. Male scutum uniquely with a pair of bluish bands. Male epandrium inner-laterally with two strong tubercle-like setae, and with ventrally elongated surstylus. Distiphalli bilaterally asymmetrical and tapering toward apex. Larva mines the thallus of *Ricciabillardieri* and *R.huebeneriana*.

#### Description.

**Figure 68.**
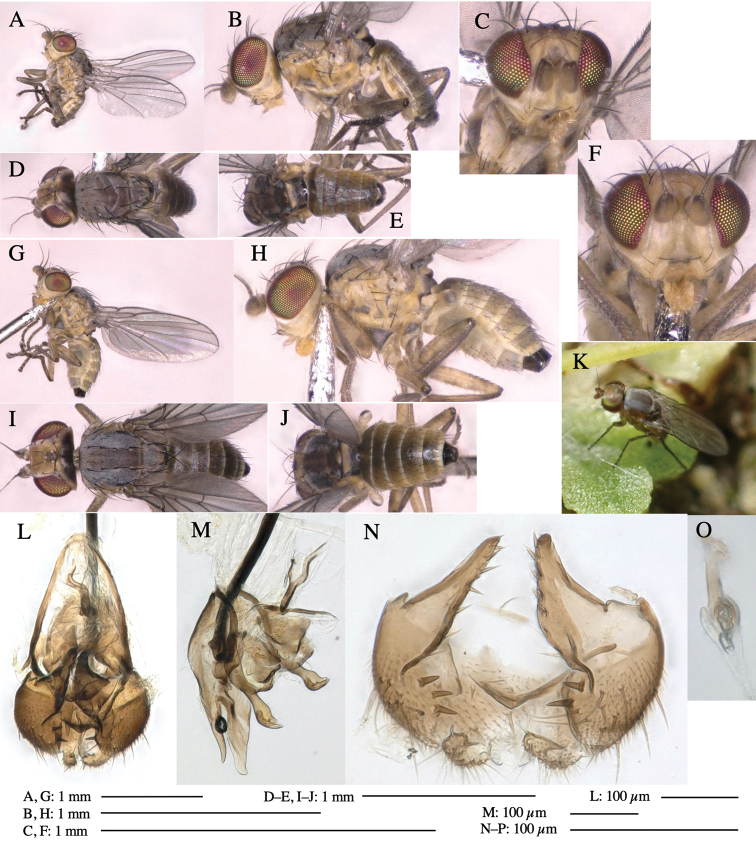
*Phytoliriomyzacaerulescens*sp. nov. **A–E **holotype male **A** habitus **B** lateral **C** frontal **D** dorsal **E** posterior **F–J** paratype female (MK-AG-a558) **F** frontal **G** habitus **H **lateral **I **dorsal **J** posterior **K** female fly ovipositing to thallus of *Ricciabillardieri***L–O** male genitalia **L** whole genitalia, ventral **M** phallic complex, lateral **N** epandrium, ventral **O** ejaculatory apodeme, ventral.

**Adult male** (Fig. 68A–E).

***Head*:** Head largely light yellow, with frons and ocellar tubercle pruinose brown, and back of head dark brown (Fig. 68C). Antenna porrect; first flagellomere, pedicel and scape brown (Fig. 68B). Arista subbasal, brown, pubescent. Face, gena, parafacial and postgena yellow. Proboscis normal, yellow; palpus light yellow, cylindrical (Fig. 68C). ***Chaetotaxy*:** Front orbitals three pairs; one ori directed inward; two ors directed upward (Fig. 68B). Orbital setulae minute and proclinate, in a single row.

***Thorax*:** Thorax pruinose. Scutum and scutellum, bluish gray with a medial and a pair of lateral longitudinal obscure dark gray bands (Fig. 68D). Subscutellum brownish yellow except brown posterior half. Mediotergite dark brown, anatergite light yellow except ventral dark brown corner, and katatergite light yellow (Fig. 68E). Pleuron largely brownish yellow; anepisternum with brown patch along lower margin; anepimeron with a small spot on anterior ventral corner; katepisternum and meron with large brown patches on venter (Fig. 68B). Haltere gray with inner surface light yellow, with stalk light yellow. Calypter margin and hairs gray. Leg segments yellowish brown, subdistally and basally with dark patches; tibia and tarsus darker (Fig. 68A). ***Chaetotaxy*:** Scutum with 1+3 dorsocentrals, shortened anteriorly (Fig. 68D). Acrostichal seta three or four pairs in two irregular rows. ***Wing*:** Wing length 1.1 mm, costa reaching M_1_ (Fig. 68A). Length of ultimate section of vein M_4_ divided by penultimate section 1.8.

***Abdomen*:** Abdomen dorsally subshiny brown, posterior margin of each tergite narrowly yellow; epandrium dark brown (Fig. 68E). ***Genitalia*:** (Fig. 68L–O) Epandrium rounded apically; inner-lateral surface with 2–3 sharp, dark tubercle-like setae (Fig. 68N). Surstylus large and ventrally extended, tapering toward apex, with a row of strong setae on ventral margin (Fig. 68N). Cercus narrow, setose. Subepandrial sclerite with a pair of pointed dorsal lobes converging together distally, with a pair of narrow lateral plate-like lobes protruding dorsally (Fig. 68N). Hypandrium thin, sclerotized along outer margin (Fig. 68L). Postgonite bare, goose barnacle-shaped, cleft apically (Fig. 68M). Phallophorus with shallow incision below, articulated with dark phallapodeme, fused to epiphallus (Fig. 68L). Basiphallus with a pale dorsal broad sclerite; as long as mesophallus + distiphallus (Fig. 68M). Hypophallus broad membranous; lateral margins darkly sclerotized (Fig. 68M). Paraphallus absent. Mesophallus dark, cylindrical; with a pair of narrow lateral sclerites. Distiphallus comprising one pair of dark bilaterally asymmetrical tubules; left one is S-shaped, longer and thicker than the right one, tapering toward apex; right tubule short, truncated (Fig. 68M). Ejaculatory apodeme pale brown and fan-shaped with broad stalk; base dark and wide to one side; sperm pump clear (Fig. 68O).

**Figure 69.**
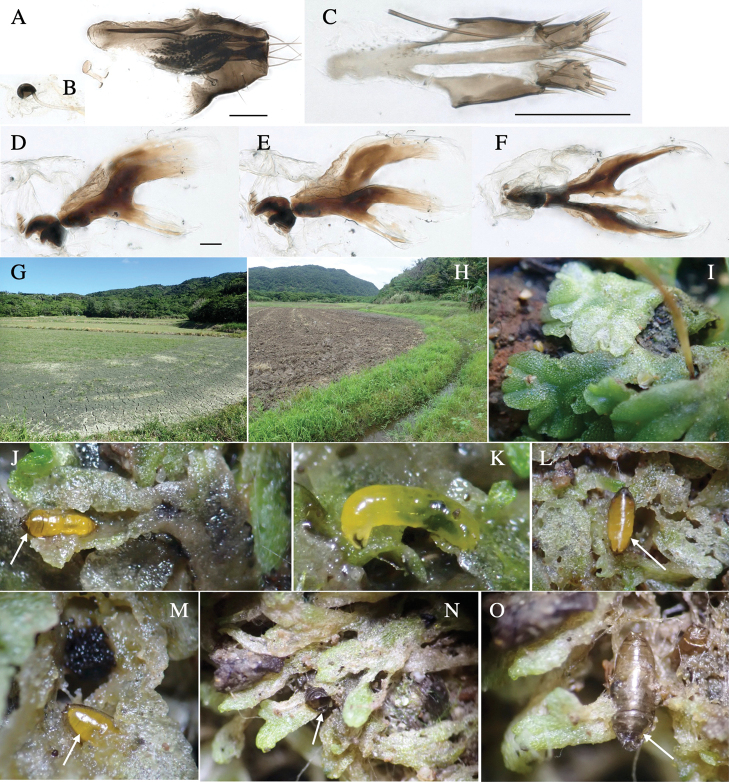
Female morphology and larval morphology/ecology of *Phytoliriomyzacaerulescens*sp. nov. **A–C **female postabdomen **A** oviscape **B** spermatheca **C** tergite 10 **D–F** Pharyngeal skeleton of 3^rd^ larva **D** lateral **E** dorso-lateral **G, H** landscape of the habitat (**G** at type locality **H** at Urauchi) **I–O** mined thalli, mining larvae and puparia (**I–M** on *Ricciabillardieri***N–O** on *R.huebeneriana*) **K** larva extracted from mine **J, L–O **puparia. Scale: 100µm.

**Female** (Fig. 68F–K). Similar to male, but slightly larger and frons wider than male. Ground color of scutum is more bluish, and two bluish bands on scutum are more intense than those of male. Wing length 1.3 mm. ***Postabdomen*:** (Fig. 69A–C) Oviscape dark brown, setigerous (Fig. 69A). Tergite 10 trifurcate, laterally uniting narrow pleural sclerites (Fig. 69C). Each cercus with two stout, apical, trichoid sensilla, 1/2 length of cercus (Fig. 69A). Spermathecae semi-orbicular; with truncate proximal ends (Fig. 69B).

**Immatures.** (Fig. 69D–F, J–O) At 3^rd^ instar, larval body yellow and cylindrical. Mandibles paired, fused, asymmetrical and strongly sclerotized, each with two teeth; left teeth larger than right ones (Fig. 69D–F). Mandibles confluent with short sclerotized hypopharyngeal sclerite, connecting to a pair of tentoropharyngeal sclerites; dorsal cornu broad and long, ventral cornu shorter, comprising two fused arms; upper arm darkly sclerotized only at basal part. Puparium internal, slender and brown (Fig. 69J–O).

#### Etymology.

The specific name (*caerulescens* = blue) refers to the bluish bands on the scutum, which are especially prominent in the female.

#### Japanese name.

Aosuji-hatakegoke-hamoguribae.

#### Host plants.

*Ricciabillardieri* (Fig. 69I–M) and *R.huebeneriana* (Fig. 69N, O) (Ricciaceae).

#### Mine.

(Fig. 69I–O) Larvae construct linear-blotch mines in the thallus, and pupate in the mines. Because the thalli of the host plants are small and thin, the larva sometimes relocates to a fresh thallus. In the mine of the thallus, sporangia are left intact (Fig. 69M).

#### Biological notes.

 The two host liverwort species grow on levees of paddy fields in subtropical islands (Fig. 69G, H). Recent overuse of insecticides and herbicides and abandonment of rice cultivation in these islands has caused a drastic decrease of diverse paddy-field-dependent plants, and these liverworts and the liverwort-associated fly species are considered to be threatened. Larvae of *P.caerulescens* were found mining thalli of these liverwort species in early November and adults emerged from late November to December.

#### Distribution.

Japan: Ishigaki and Iriomote Islands (Fig. 65).

#### Remarks.

This species is unique in that the female has blue lateral bands on the scutum. It resembles *P.iriomotensis*,*P.ugetsu*, and *P.phaeocerotis* in having a wholly dark scutum and yellow maxillary palpi, but it is distinguished from them by the blueish scutum and by the shape of the surstylus of the male epandrium (well-sclerotized, prolonged, and tapering ahead in *P.caerulescens*; less-sclerotized, not prolonged, and curved inward in the other species).

##### Species associated with hornworts

### ﻿37.﻿ Phytoliriomyza foliocerotis

Taxon classificationAnimaliaDipteraAgromyzidae

Kato sp. nov.

65846D67-9717-5F3C-BBC6-1D8052652938

https://zoobank.org//CFCA1020-3149-4BAB-98D8-32D80783407F

Fig. 70

#### Material examined.

***Holotype*:** Japan: 1♂ (MK-AG-a310), Mt. Osuzu, Tsuno, Miyazaki Pref. (32.251°N, 131.481°E, 150 m asl), 11-IV-2021 (as larva), emerged on 26-IV-2021, NSMT-I-Dip 32110. ***Paratypes*:** Japan: 1♂2♀ (MK-AG-a451, a449, a450), type locality, 14-VII-2021 (as larva), emerged on 1–6-VIII-2021, NSMT-I-Dip 32111–32113.

#### Other material.

Japan: 2♂1♀, Mt. Osuzu, Tsuno, Miyazaki Pref., 14-VII-2021 (as larva), emerged on 31-VII–18-VIII-2021.

#### Diagnosis.

A small species (wing length 1.1–1.3 mm) having a subshiny black scutum, black scutellum with small yellow spot centrally, yellow 1^st^ flagellomere, yellow maxillary palpus, yellow halteres, and yellow legs. Male epandrium inner-laterally with one strong tubercle-like seta. Distiphalli tapering toward apex, fused after meeting, elongated over the length of phallapodeme. Larva mines the thallus of a hornwort, *Foliocerosfuciformis*.

#### Description.

**Figure 70.**
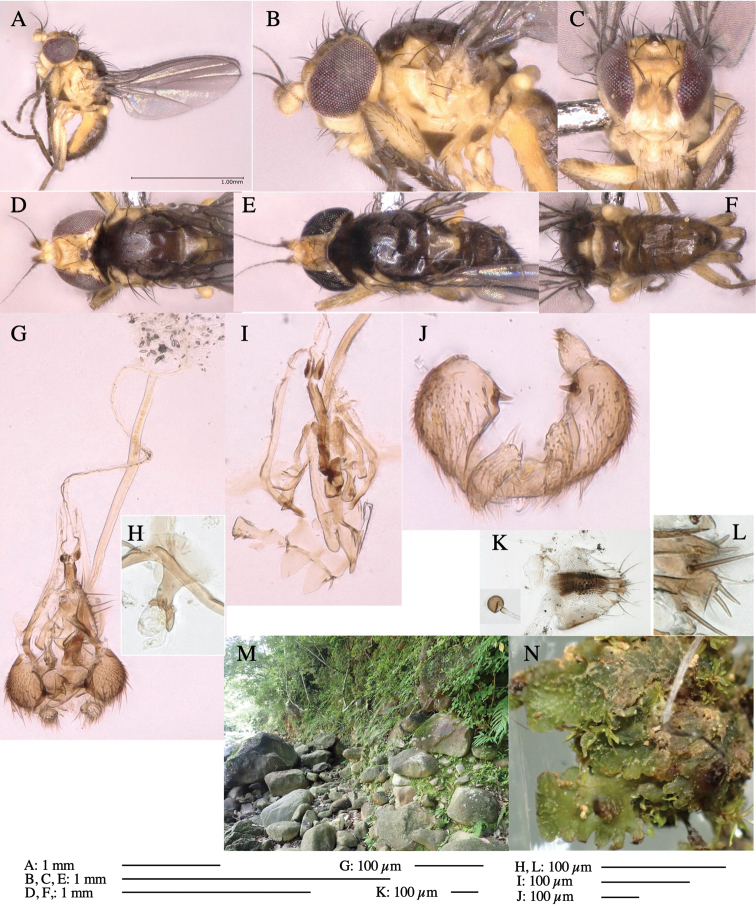
*Phytoliriomyzafoliocerotis*sp. nov. **A–D **holotype male **A** habitus **B** lateral **C** frontal **D** dorsal **E, F** paratype female (MK-AG-a450) **E** dorsal **F** posterior **G–J** male genitalia **G** whole genitalia, ventral **H** ejaculatory apodeme, lateral **I** phallic complex, lateral **J** epandrium, posterior-ventral **K, L** female postabdomen **K** oviscape and spermatheca with spermatheca **L** tergite 10 **M** habitat at type locality **N** mined thallus of *Foliocerosfuciformis*

**Adult male** (Fig. 70A–D).

***Head*:** Head light yellow, with ocellar tubercle dark brown, and back of head dark brown excluding margins (Fig. 70C). Antenna porrect; first flagellomere yellow, only narrow area around base of arista grayish; pedicel and scape yellow (Fig. 70B). Arista subbasal, brown, pubescent. Frons brownish yellow, with reflective pruinosity. Face, gena, parafacial and postgena light yellow. Proboscis normal, light yellow; palpus light yellow, ovate (Fig. 70C). ***Chaetotaxy*:** Front orbitals three pairs; one ori directed inward; two ors directed upward (Fig. 70D). Orbital setulae minute and proclinate, in a single row.

***Thorax*:** Thorax subshiny. Scutum pruinose black (Fig. 70D). Scutellum black, medially with a small, obscure yellow patch. Subscutellum pale yellow. Mediotergite dark gray, anatergite brown, and katatergite pale yellow (Fig. 70B). Pleuron largely pale yellow; postpronotal lobe with anterior brown spot; propleuron with a small brown patch on mid-anterior corner; notopleuron with narrow brown patch on anterior-lower margin; anepisternum and anepimeron without brown spot; katepisternum and meron with large brown patches on venter (Fig. 70B). Haltere grayish yellow, with stalk paler. Calypter margin and hairs gray. Leg segments pale yellow; femur of foreleg with narrow brown subdistal patch frontally; tibia and tarsus darker (Fig. 70A). ***Chaetotaxy*:** Scutum with 1+3 dorsocentrals, shortened anteriorly (Fig. 70D). Acrostichal setae three or four pairs in two rows. ***Wing*:** Wing length 1.3 mm, costa reaching M_1_ (Fig. 70A). M_4_ disappears immediately before reaching wing margin. Length of ultimate section of vein M_4_ divided by penultimate section 1.7.

***Abdomen*:** Abdomen dorsally subshiny yellowish brown; epandrium brown (Fig. 70E). ***Genitalia*:** (Fig. 70G–J) Epandrium rounded apically; posterior end of inner margin with one strong tubercle-like seta (Fig. 70J). Surstylus small, spatula-shaped, setose apically (Fig. 70J). Cercus narrow, setose. Subepandrial sclerite vestigial. Hypandrium thin, slightly sclerotized along outer margin (Fig. 70G). Postgonite bare, goose barnacle-shaped, rounded apically (Fig. 70I). Phallophorus with shallow incision below, articulated with phallapodeme, fused to epiphallus (Fig. 70I). Basiphallus with dark narrow dorsal sclerite and apically bilobed pale ventral sclerite (Fig. 70I). Hypophallus membranous with microtrichia, (Fig. 70I). Paraphallus absent. Mesophallus cylindrical, laterally and dorsally sclerotized, parallel-sided (Fig. 70I). Distiphallus comprising one pair of tubules, with dark dorsal shoehorn-shaped sclerites basally; the tubules thin, fused after meeting, elongated over the length of phallapodeme (Fig. 70G). Ejaculatory apodeme fan-shaped, with short broad stalk and dark base; sperm pump clear (Fig. 70H).

**Female** (Fig. 70E–F). Similar to male, but larger, frons wider. Wing length 1.1&nbsp;mm. ***Postabdomen*:** (Fig. 70K, L) Oviscape dark brown, setigerous (Fig. 70K). Tergite 10 cruciform, laterally uniting narrow pleural sclerites. Each cercus with two stout, apical, trichoid sensilla, same length as cercus (Fig. 70L). Spermathecae semi-orbicular; with truncate proximal ends (Fig. 70K).

#### Etymology.

The specific name refers to the host plant genus *Folioceros*.

#### Japanese name.

Miyabetsunogoke-hamoguribae.

#### Host plant.

*Foliocerosfuciformis* (Anthocerotaceae).

#### Mine.

Mines are extremely inapparent because the thalli are thick and often overlapping (Fig. 70N).

#### Biological notes.

The habitat of this species is a cliff along a river bank in warm temperate evergreen forests dominated by *Castanopsissieboldii* and *Quercushelva* (Fig. 70M), and the host hornwort grows with the liverwort *Marchantiapapillatagrossibarba*. Our rearing records suggest that this species is at least bivoltine, with adults emerging in April and August.

#### Distribution.

**Figure 71.**
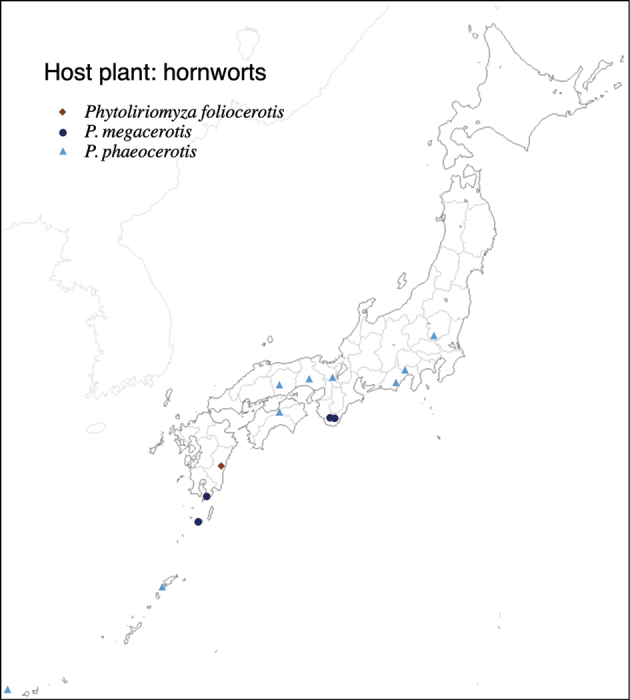
Locality records of three *Phytoliriomyza* species associated with hornworts: *P.foliocerotis*, *P.megacerotis*and *P.phaeocerotis*.

Japan: Kyushu (Fig. 71). Recorded only from the type locality.

#### Remarks.

 This species resembles *P.nubatama* in having a shiny black dorsal scutum and a small yellow spot in the black scutellum; it is distinguished from the latter by the yellow 1^st^ flagellomere of the antenna (dark in *P.nubatama*). These two species were found sympatrically at the type locality, where their host plants, *Marchantiapapillatagrossibarba* and *Foliocerosfuciformis*, grow in similar riparian habitats. Irrespective of their similar external morphology, these species are evidently distantly related, given their greatly differing genital morphology.

The three agromyzid species recorded from hornworts all had a dark scutum, but varied among species in color of antenna, color of maxillary palpus, and comb of tubercle-like setae on male epandrium. They also had common characteristics in the male genitalia; the distiphallus is little sclerotized, elongated, and tapering toward the apex. These characteristics of the male genitalia in hornwort-associated species suggest their monophyletic origin.

### ﻿38.﻿ Phytoliriomyza megacerotis

Taxon classificationAnimaliaDipteraAgromyzidae

Kato sp. nov.

B329DE62-E344-544C-9E07-4C524418D728

https://zoobank.org/658789E1-B6A7-464B-B701-5D17528CC4E3

Figs 72 , 73

#### Material examined.

***Holotype*:** Japan: 1♂ (MK-AG-a417), Kotonotaki, Susami, Wakayama Pref. (33.5639°N, 135.5437°E, 200 m asl), 24-III-2020 (as larva), emerged on 1-V-2020, NSMT-I-Dip 32114. ***Paratypes*:** Japan: 1♂2♀ (MK-AG-a479, a480, a14), same data as holotype, emerged on 30-IV–2-V-2020, NSMT-I-Dip 32115–32117; 1♀ (MK-AG-146), Tashiro, Kinko, Kimotsuki, Kagoshima Pref., 22-III-2015 (as larva), emerged on 1-V-2015, NSMT-I-Dip 32118; 1♂1♀ (MK-AG-154, a15), Yasukawa-keikoku, Tanabe, Wakayama Pref., 31-VII-2015 (as larva), emerged on 3-IX-2015, NSMT-I-Dip 32119–32120.

#### Other material.

Japan: 8♂9♀, Kotonotaki, Susami, Wakayama Pref., 24-III-2020 (as larva), emerged on 31-I–2-V-2020; 16♂28♀, Yasukawa-keikoku, Tanabe, Wakayama Pref., 31-VII-2015 (as larva), emerged on 25-VIII–2-IX-2020; 1♀, Wadagawa-kyo, Kumanogawa, Shingu, Wakayama Pref., 7-VII-2021 (as larva), emerged on 18-VIII-2021; 9♂25♀, Tashiro, Kinko, Kimotsuki, Kagoshima Pref., 22-III-2015 (as larva), emerged on 30-IV–30-IV–10-V-2020; 1♀, Isso, Yaku Is., Kumage, Kagoshima Pref., 29-III-2017 (as larva), emerged on 12-V-2021.

#### Diagnosis.

 A small species (wing length 1.2–1.4 mm) having a pruinose gray scutum and scutellum, black 1^st^ flagellomere, black maxillary palpus, dark gray halteres, and dark gray legs. Male epandrium inner-basally with a comb comprising five or six fused long tubercle-like setae; surstylus with a comb comprising five or six fused long tubercle-like setae. Larva mines the thallus of a riparian hornwort, *Megacerosflagellaris*.

#### Description.

**Figure 72.**
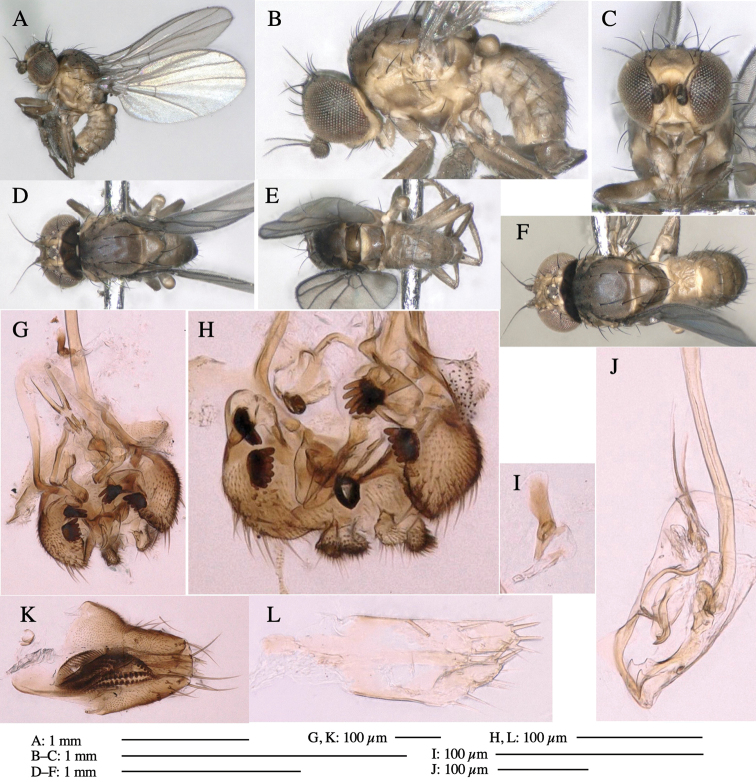
*Phytoliriomyzamegacerotis*sp. nov. **A–E **holotype male **A** habitus **B** lateral **C** frontal **D** dorsal **E** posterior **F** paratype female (MK-AG-a14), dorsal **G–J** male genitalia (**G** at type locality **H, I** at Tashiro **J** at Yasukawa-keikoku) **G** whole genitalia, ventral **H** epandrium, ventral **I** ejaculatory apodeme, lateral **J** phallic complex, lateral **K, L** female postabdomen** K** oviscape and spermatheca **L** tergite 10.

**Adult male** (Fig. 72A–E).

***Head*:** Head dark yellow, with ocellar tubercle dark brown, and back of head dark brown excluding margins (Fig. 72C). Antenna porrect; first flagellomere black, pedicel and scape brown (Fig. 72B). Arista subbasal, brown, pubescent. Frons brownish yellow, with reflective pruinosity. Clypeus narrow, brown with apical half yellow; slightly protruding. Face, gena, parafacial and postgena dark yellow (Fig. 72C). Proboscis normal, light yellow; palpus dark brown, cylindrical. ***Chaetotaxy*:** Front orbitals three pairs; one ori directed inward; two ors directed upward (Fig. 72B). Orbital setulae minute and proclinate, in a single row.

***Thorax*:** Thorax pruinose, dark gray. Scutum pruinose, dark brown; scutellum pruinose, brown; subscutellum light yellow (Fig. 72D). Mediotergite subshiny, dark brown, anatergite and katatergite brown (Fig. 72E). Pleuron largely dark yellow; postpronotal lobe with anterior brown patch; propleuron with a small brown patch on anterior corner; notopleuron yellowish brown; anepisternum dark yellow with lower-half yellowish brown; anepimeron with anterior brown patch; katepisternum and meron with large brown patches on venter (Fig. 72B). Haltere brown. Calypter margin and hairs gray. Leg segments dark brown; tibia and tarsus darker (Fig. 72A); distal half of coxa and basal half of femur of foreleg pale yellow frontally (Fig. 72C). ***Chaetotaxy*:** Scutum with 1+3 dorsocentrals, shortened anteriorly (Fig. 72D). Acrostichal setae two or three pairs in two rows. ***Wing*:** Wing length 1.2 mm, costa reaching M_1_ (Fig. 72A). Length of ultimate section of vein M_4_ divided by penultimate section 1.6–1.8.

***Abdomen*:** Abdomen dorsally subshiny brown; epandrium brown (Fig. 72E). ***Genitalia*:** (Fig. 72G–J) Epandrium rounded apically; distal margin with several long setae; inner-basal margin with a comb comprising five or six fused dark long tubercle-like setae (Fig. 72H). Surstylus rounded, apically with a comb comprising five or six fused dark long tubercle-like setae (Fig. 72H). Cercus narrow, setose. Subepandrial sclerite dark, with a pair of pale, elongated, fused, plate-like dorsal lobes (Fig. 72H). Hypandrium thin, sclerotized along outer margin (Fig. 72G). Postgonite bare, goose barnacle-shaped, cleft apically (Fig. 72G). Phallophorus with deep incision below, articulated with phallapodeme, fused to epiphallus (Fig. 72J). Basiphallus membranous, with dorsal surface sclerotized. Hypophallus small, with a pair of asymmetrical narrow sclerites; the left one is longer than the right one (Fig. 72G, J). Paraphallus absent. Mesophallus cylindrical, with a pair of dorsolateral sclerites (Fig. 72J). Distiphallus comprising one pair of tubules; the tubules adjoining only at the base, tapering toward tip, with outer-lateral sides pigmented, extending up to half point of phallapodeme, becoming paler apically (Fig. 72J). Ejaculatory apodeme brownish, spatula-shaped with short, broad stalk; base dark and wide to one side; sperm pump clear (Fig. 72I).

**Female** (Fig. 72F). Similar to male, but larger, frons wider. Wing length 1.4 mm. ***Postabdomen*:** (Fig. 72K, L) Oviscape dark brown, setigerous (Fig. 72K). Tergite 10 cruciform, laterally uniting narrow pleural sclerites (Fig. 72L). Each cercus with two stout, apical, trichoid sensilla, 1/2 length of cercus (Fig. 72L). Spermathecae semi-orbicular, with truncate proximal ends (Fig. 72K).

**Figure 73.**
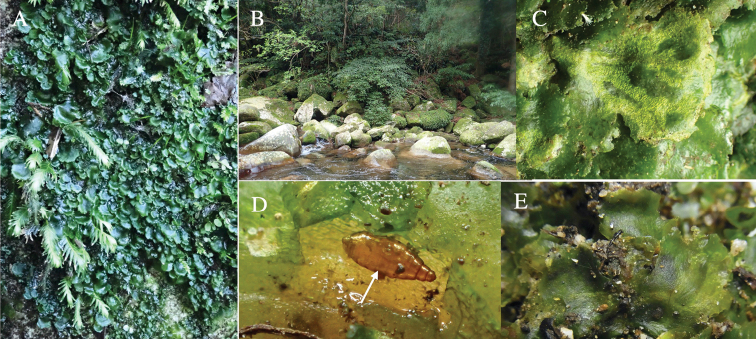
Larval ecology of *Phytoliriomyzamegacerotis*sp. nov. **A, B **habitats **(A **at type locality **B** at Yakushima) **C–E** mined thalli of *Megacerosflagellaris***C, D** at type locality **E** at Tashiro. An arrow indicates internal puparium.

**Immatures.** (Fig. 73D) Puparium internal, slender, brown, and slightly flattened, with posterior 1/3 tapering.

#### Etymology.

The specific name refers to the host plant genus *Megaceros*.

#### Japanese name.

Ananashitsunogoke-hamoguribae.

#### Host plant.

*Megacerosflagellaris* (Dendrocerotaceae).

#### Mine.

Larvae construct linear-blotch mines in the thallus, and pupate in and rarely out of the mines (Fig. 73C–E).

#### Biological notes.

 The habitats of this species are watersides along river banks or near water fall in warm temperate evergreen forests dominated by *Castanopsissieboldii* (Fig. 73B), and is unique in that the host hornworts are always wet due to splashed water (Fig. 73A). Our rearing records suggest that this species is at least bivoltine, with adults emerging in May and August.

#### Distribution.

Japan: Honshu, Kyushu and Yaku Island (Fig. 71).

#### Remarks.

 This species resembles *P.suetsugui* and *P.ricciae* in having a wholly dark scutum and dark maxillary palpus, but is distinguished from them by the color of the pedicel and scape of the antenna (dark in *P.megacerotis*; yellow in *P.suetsugui* and *P.ricciae*).

### ﻿39.﻿ Phytoliriomyza phaeocerotis

Taxon classificationAnimaliaDipteraAgromyzidae

Kato sp. nov.

D6949971-FB23-58D2-A867-C02605D5CA43

https://zoobank.org/B6F924D3-86D3-4A6E-9C66-DE45C0188495

Figs 74 , 75

#### Material examined.

***Holotype*:** Japan: 1♂ (MK-AG-150), Muramatsu, Iwakura, Sakyo-ku, Kyoto Pref. (35.0931°N, 135.7900°E, 150 m asl), 16-XI-2017 (as larva on *Notothylastemperata*), emerged on 2-I-2018, NSMT-I-Dip 32121. ***Paratypes*:** Japan: 2♂1♀ (MK-AG-a481, a483, a482), same data as holotype, emerged on 24-XI-2017–5-I-2018, NSMT-I-Dip 32122–32124; 1♀ (MK-AG-135), type locality, 22-IV-2016 (as larva on *Phaeoceroscarolinianus*), emerged on 10-V-2016, NSMT-I-Dip 32125; 1♀ (MK-AG-a10), type locality, 15-XI-2019 (as larva on *Ph.carolinianus*), emerged on 18-XII-2019, NSMT-I-Dip 32126; 1♂ (MK-AG-a415), Inago, Shibakawa, Fujinomiya, Shizuoka Pref., 17-XII-2019 (as larva on *Anthocerospunctatus*), emerged on 24-I-2020, NSMT-I-Dip 32127; 1♀ (MK-AG-a375), Mt. Gion, Takahashi, Okayama Pref., 9-X-2017 (as larva on *Notothylastemperata*), emerged on 3-XI-2017, NSMT-I-Dip 32128; 1♀ (MK-AG-a11), Shodon, Kakeroma Is., Setouchi, Kagoshima Pref., 24-I-2019 (as larva on *Ph.carolinianus*), emerged on 24-II-2019, NSMT-I-Dip 32129; 1♂ (MK-AG-a12), Minami-bokujo, Yonaguni Is. Yaeyama, Okinawa Pref., 5-III-2019 (as larva on *Ph.carolinianus*), emerged on 2-IV-2019, NSMT-I-Dip 32130.

#### Other material.

 Japan: On *Phaeoceroscarolinianus*: 27♂52♀, Yudenno-sato, Sugegaya, Makinohara, Shizuoka Pref., 9-I-2018 (as larva), emerged on 2–26-II-2018; 2♂5♀, Megami, Makinohara, Shizuoka Pref., 10-XII-2017 (as larva), emerged on 30-I–7-II-2018; 11♂14♀, Muramatsu, Iwakura, Sakyo-ku, Kyoto Pref., 3-I-2018 (as larva), emerged on 19-I–23-II-2018; 18♂26♀, Shiozuka-kogen, Yamashiro, Miyoshi, Tokushima Pref., 5-XI-2017 (as larva), emerged on 29-XI-2017–9-II-2018; 6♂15♀, Shodon, Kakeroma Is., Setouchi, Kagoshima Pref., 23-I-2019 (as larva), emerged on 2-II–11-III-2019; 2♂, Minami-bokujo, Yonaguni Is. Yaeyama, Okinawa Pref., 5-III-2019 (as larva), emerged on 2-IV-2019.

On *Notothylastemperata*: 1♀, Inago, Shibakawa, Fujinomiya, Shizuoka Pref., 17-XII-2019 (as larva), emerged on 19-I-2020; 8♂17♀, Muramatsu, Iwakura, Sakyo-ku, Kyoto Pref., 16-XI-2017 (as larva), emerged on 20-XI–21-II-2017; 1♂, Mita-ike, Toyokura, Kasai, Hyogo Pref., 30-X-2017 (as larva), emerged on 17-XII-2017; 9♂10♀, Mt. Gion, Takahashi, Okayama Pref., 6-XI-2017 (as larva), emerged on 12-XI–5-XII-2017.

On *Notothylasorbicularis*: 1♂, Izuruhara, Tamura, Tochigi Pref., 2-XI-2021 (as larva), emerged on 10-XII-2021.

On *Anthocerospunctatus*: 1♂1♀, Inago, Shibakawa, Fujinomiya, Shizuoka Pref., 7-XII-2019 (as larva), emerged on 8–28-I-2020; 1♂1♀, Mita-ike, Toyokura, Kasai, Hyogo Pref., 30-X-2017 (as larva), emerged on 28–29-XI-2017.

#### Diagnosis.

 A small species (wing length 1.2–1.5 mm) that has a pruinose gray scutum and scutellum, brown 1^st^ flagellomere, yellow maxillary palpus, brown halteres, and yellow legs. Male epandrium inner-laterally with a short tubercle-like seta. Distiphalli elongated, tapering toward apex, more than 2 × longer than the phallapodeme. the larvae mine thalli of hornworts belonging to the following genera: *Notothylas*,*Phaeoceros* and *Anthoceros*.

#### Description.

**Figure 74.**
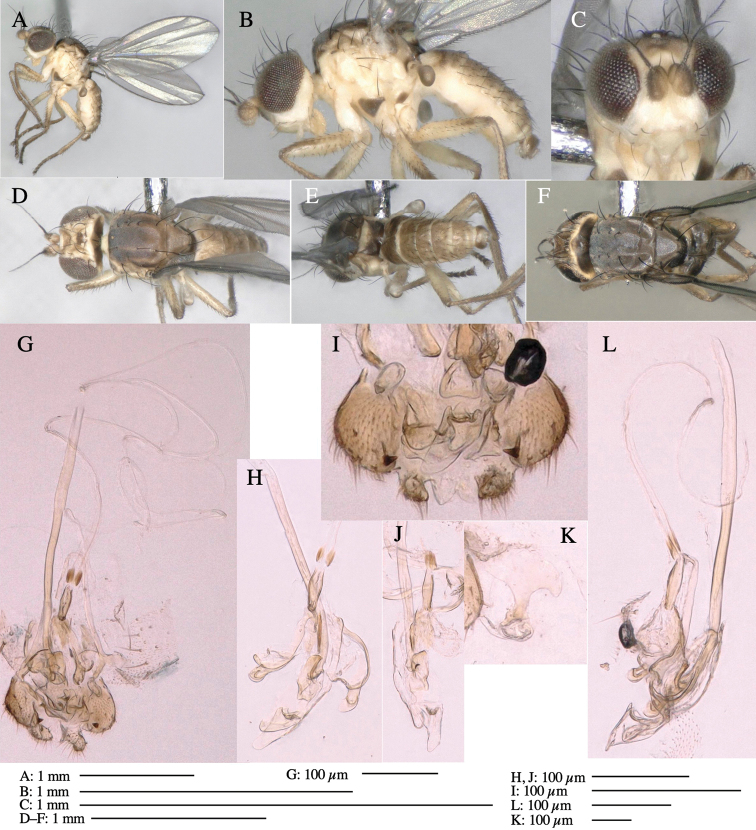
*Phytoliriomyzaphaeocerotis*sp. nov. **A–E **holotype male **A** habitus **B** lateral **C** frontal **D** dorsal **E** posterior **F** paratype female (MK-AG-a10), dorsal **G–K** male genitalia (**G, H** emerged from *Notothylastemperata* at type locality **I** emerged from *Phaeoceroscarolinianus* at Kakeroma Is. **J** emerged from *Notothylastemperata* at Kibi-kogen **K** emerged from *Anthocerospunctatus* at Inago) **G** whole genitalia, ventral **H, J** phallic complex, lateral and ventral **I** epandrium, ventral **K** ejaculatory apodeme, lateral **L** phallic complex, lateral.

**Adult male** (Fig. 74A–E).

***Head*:** Head entirely pale yellow, with ocellar tubercle dark brown, and back of head dark brown excluding margins (Fig. 74C). Antenna porrect; first flagellomere brown, pedicel and scape grayish yellow (Fig. 74B). Arista subbasal, brown, pubescent. Frons brownish yellow, with reflective pruinosity. Face, gena, parafacial and postgena yellow. Proboscis normal, pale yellow; palpus pale yellow, ovate (Fig. 74C). ***Chaetotaxy*:** Front orbitals three pairs; one ori directed inward; two ors directed upward (Fig. 74B). Orbital setulae minute and erect, in a single row.

***Thorax*:** Thorax subshiny, pale yellow. Scutum and scutellum pruinose gray (Fig. 74D). Subscutellum pale yellow. Mediotergite dark gray, anatergite brown, and katatergite pale yellow (Fig. 74E). Pleuron largely pale yellow; postpronotal lobe with anterior brown spot; propleuron with a small brown patch on mid-anterior corner; notopleuron with narrow brown patch on anterior-lower margin; anepisternum and anepimeron without brown spot; katepisternum and meron with large brown patches on venter (Fig. 74B). Haltere grayish yellow, with stalk paler. Calypter margin and hairs gray. Leg segments brownish yellow with coxa pale yellow; femur of foreleg with narrow brown subdistal patch on frontal side; tibia and tarsus darker (Fig. 74A). ***Chaetotaxy*:** Scutum with 1+3 dorsocentrals, shortened anteriorly (Fig. 74D). Acrostichal seta absent. ***Wing*:** Wing length 1.2–1.3 mm, costa reaching M_1_ (Fig. 74A). M_4_ disappears immediately before reaching wing margin. Length of ultimate section of vein M_4_ divided by penultimate section 1.8–2.5.

***Abdomen*:** Abdomen dorsally subshiny yellowish brown; epandrium brown (Fig. 74E). ***Genitalia*:** (Fig. 74G–L) Epandrium rounded apically; posterior end of inner margin with one or two tubercle-like setae (Fig. 74G, I). Surstylus small, ovate, membranous, without setae (Fig. 74I). Cercus narrow, setose. Subepandrial sclerite with a pair of pale plate-like dorsal arms directed posteriorly and a pair of pale elongated plate-like ventral arms directed ventrally (Fig. 74I). Hypandrium thin, slightly sclerotized along outer margin (Fig. 74G). Postgonite bare, goose barnacle-shaped, pointed and cleft apically (Fig. 74H, J). Phallophorus with shallow incision below, articulated with phallapodeme, connecting to asymmetric epiphallus (Fig. 74H, J, L). Basiphallus supported by a pair of rod-like lateral sclerites (Fig. 74H). Hypophallus membranous, centrally with a pair of narrow sclerites (Fig. 74G–J). Paraphallus absent. Mesophallus cylindrical, dorsolaterally sclerotized, medially widest. Distiphallus is a fused tubule comprising one pair of elongated tubules, with dark shoehorn-shaped sclerites basally; the tubule pale, extremely elongated, longer than phallapodeme; easily unfolded in the course of dissection (Fig. 74G). Ejaculatory apodeme pale, fan-shaped with short brown stalk and clear sperm pump (Fig. 74K).

**Figure 75.**
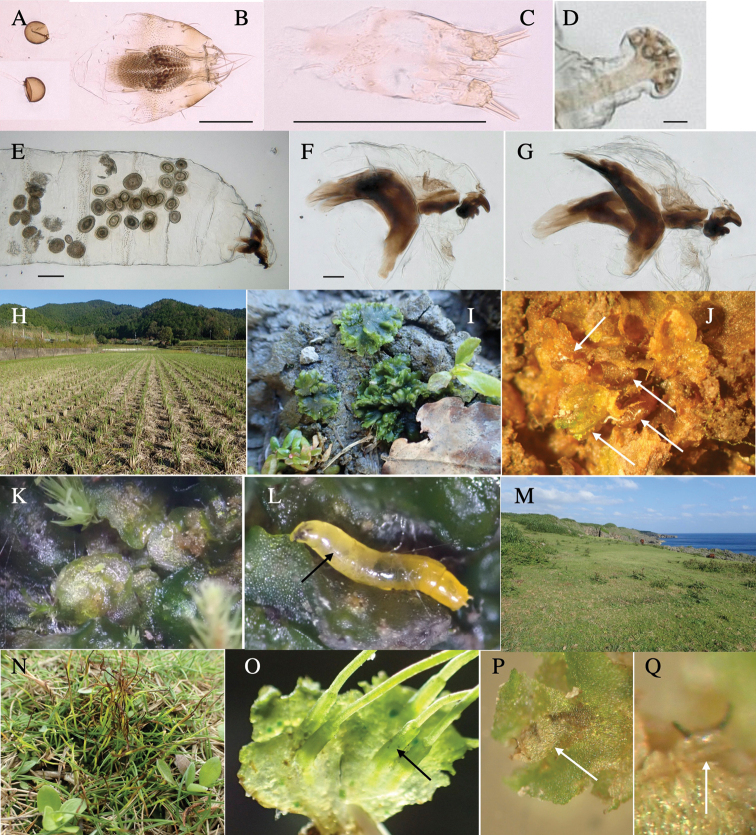
Female morphology and larval morphology/ecology of *Phytoliriomyzaphaeocerotis*sp. nov. **A–C **female postabdomen **A** spermatheca **B** oviscape and spermatheca **C** tergite 10 **D–G**, larval morphology **D** posterior spiracle **E** anterior body **F, G** pharyngeal skeleton in lateral and ventral-lateral view **H, I** habitat at type locality **H** harvested rice field **I***Notothylastemperata* thalli growing on soil after harvest of rice **J–L** mined thalli of *Phaeoceroscarolinianus* at type locality **M–Q** habitat (**M**) thalli bearing sporophytes (**N**) and mined thallus (**O**) of *P.carolinianus* at Yonaguni Is., Black and white arrows i indicate larvae and puparia, respectively. Scale: 100 µm (**A–C, E–G)**; 10 µm (**D**).

**Female** (Fig. 74F). Similar to male, but larger, frons wider. Wing length 1.5 mm. ***Postabdomen*:** (Fig. 75A–C) Oviscape dark brown, setigerous (Fig. 75B). Tergite 10 cruciform, laterally uniting narrow pleural sclerites (Fig. 75C). Each cercus with two stout, apical, trichoid sensilla, ca. same length as cercus (Fig. 75C). Spermathecae semi-orbicular, with truncate proximal ends (Fig. 75A).

**Immatures.** (Fig. 75D–G, L–Q) At 3^rd^ instar, larval body yellow and cylindrical. Mandibles paired, fused and strongly sclerotized, each with two teeth; left teeth larger than right teeth (Fig. 75D–G). Mandibles confluent with short sclerotized hypopharyngeal sclerite, connecting to a pair of tentoropharyngeal sclerites; dorsal cornu broad and long, ventral cornu shorter. Puparium internal, slender, pale brown, with anterior spiracles elongated and protruded from epidermis of mined thallus (Fig. 75P–S).

#### Etymology.

 The specific name refers to its host plant genus *Phaeoceros*.

#### Japanese name.

Niwatsunogoke-hamoguribae.

#### Host plants.

 Four hornwort species belonging to 3 families were recorded to be host plants: *Phaeoceroscarolinianus*,*Notothylastemperata* (Notothyladaceae), and *Anthocerospunctatus* (Anthocerotaceae).

#### Mine.

Larvae construct linear-blotch mines in the thallus, and pupate in the mines (Fig. 75J–M, P–S). The larvae occasionally also mine into sporophytes (Fig. 75P).

#### Biological notes.

The main habitats of this species are the paddy fields that have not experienced land improvement projects or spraying with herbicides, as mentioned for *P.ricciae* (Fig. 75H, I). These paddy fields harbor various *Riccia* species and hornwort species such as *Phaeoceroscarolinianus*,*Notothylas temperate*, and *Anthocerospunctatus* after harvesting of rice. Another habitat of this species was lawny ground in horse pasturelands in Yonaguni Island, where *Phaeoceroscarolinianus* grows among turf grasses (Fig. 75N, O). Thus, this species is the only species among the Japanese bryophyte-associated *Phytoliriomyza* species that utilizes multiple genera of bryophytes. Our rearing records suggest that it is multivoltine.

#### Distribution.

Japan: Honshu, Shikoku, Kyushu, the Ryukyu Archipelago (Fig. 71).

#### Remarks.

 This species resembles *P.iriomotensis*,*P.ugetsu*, and *P.caerulescens* in that having wholly dark scutum and yellow maxillary palpus; it is distinguished from them by the absence of tubercle-like setae on the surstylus of the male epandrium. This species resembles *P.foliocerotis* in that the distiphallus is extremely elongated, but it is distinguished from *P.foliocerotis* by its pruinose gray scutum and scutellum (shiny black in *P.foliocerotis*). This species also resembles *P.scotica* in morphology of epandrium and in having an extremely elongated distiphallus (Papp and Černý 2017); it is distinguished from the latter by the bare surstylus (distally setose in *P.scotica* Spencer) and the broad shoehorn-shaped sclerites on the basal distiphallus (sclerites narrow *in P.scotica*).

Immature stages, but not adults, were reported from a hornwort, *Megacerosvincentianus* in Mexico by Hering (1966). *Phytoliriomyzaphaeocerotis* sp. nov. (Fig. 75R) and the Mexican species share the elongated anterior spiracles.

## ﻿Discussion

### ﻿Diversity of bryophyte-associated agromyzids

Our extensive rearing of phytophagous insects on bryophytes has revealed that thalloid liverworts and hornworts in the Japanese Archipelago harbor 39 bryophyte-associated agromyzid species. This diversity was unexpectedly diverse because previously just one species was known as a liverwort thallus-miner. Our taxonomical study based on a vast collection of reared specimens has elucidated a great cryptic diversity of bryophyte-associated agromyzid species in the world.

The monophyly of the thallus-mining *Phytoliriomyza* was supported by the morphological synapomorphy of distiphallus comprising a pair of unfused long tubules in male, and cercus with two stout, apical, trichoid sensilla in female.

### ﻿Association with bryophytes

*Phytoliriomyza* species are associated with thalloid liverworts belonging to Marchantiaceae, Dumortieraceae, Aytoniaceae, Wiesnerellaceae, Conocephalaceae, and Ricciaceae, and hornworts (Notothyladaceae, Anthocerotaceae and Dendrocerotaceae). Given the low species diversity of liverworts, the number of *Phytoliriomyza* species using two common liverwort genera, *Conocephalum* and *Reboulia*, 15 and 6 species, respectively, is notably high.

The larvae of all of these species are thallus-miners; they pupate within mines unless the thalli are particularly thin and minute (e.g., *Riccia*). The mines are generally linear (particularly in early instars) and many larvae, particularly those mining in complex thalloid liverworts, excavate the lower parenchymatous layers of thalli, which makes their mines often obscure or invisible. As such, the mines are inconspicuous and cryptic, and even the biology of a well-known species, *Phytoliriomyzadorsata*, had been unknown until we reported it herein.

Among the 39 *Phytoliriomyza* species, 37 and 20 were host-specific at the genus and species levels, respectively. This host specificity is as high as that of agromyzids using angiosperms (Scheffer et al. 2007, Winkler et al. 2009). Bryophytes have diverse secondary metabolites, such as lipophilic sesqui- and diterpenoids, phenolic compounds, and polyketides; the compositions of these secondary metabolites differ among bryophyte genera and sometimes among congeneric species (Asakawa and Ludwiczuk 2017). The high host specificity of bryophyte-associated agromyzids may reflect the chemical uniqueness of each host bryophyte genus/species.

### ﻿Biogeography

Although we explored the species diversity of *Phytoliriomyza* mainly in Japan, we predict that *Phytoliriomyza* may be broadly distributed in the world. *Phytoliriomyzadorsata* is distributed from Europe to North America (Papp and Černý 2017), as well as Japan; its wide distribution mirrors that of the host liverwort, *Marchantiapolymorpha*. Similarly, *P.alpicola* is distributed in Europe, Taiwan (Sasakawa 2008), and Japan; its host, *Conocephalum*, is distributed in both regions, while *Conocephalum* species differ between Europe and East Asia: *C.conicum* in Europe; *C.salebrosum*, *C.japonicum*, and *C.* spp. 1–4 in East Asia. In North America, at least four additional species are considered members of *Phytoliriomyza* (*Phytoliriomyzafumicosta*,* P. leechei*,*P.pacifica*, and *P.volatilis*) (Spencer and Steyskal 1986); *P.pacifica* resembles *P.luna*, the *Conocephalum*-associated species, suggesting that *P.pacifica* may also be associated with *Conocephalum*. We examined a specimen of an undescribed species that induces gall formation on thalli of *Monoclea* in Peru (Ohgue et al. 2018); this species was also confirmed to be a member of *Phytoliriomyza*. Because the type species of *Lemurimyza*, *Phytoliriomyzaenormis*, was reported in Madagascar, the distribution of *Lemurimyza* also includes Africa.

Among the Japanese *Phytoliriomyza* species, 33 were recorded in Honshu, 19 in Shikoku, 17 in Kyushu, and ten in Hokkaido (Table 2). Therefore, there is no latitudinal gradient in the species diversity of *Phytoliriomyza*. The high species richness in Honshu results from the area of the island, diversity of climatic zones and vegetation, and diversity of bryophyte flora.

Some species (e.g., *Phytoliriomyzairiomotensis*, *P.calcicola*,*P.plagiochasmatos*, and *P.foliocerotis*) were local and rare, the distribution of which are mainly restricted by the narrow range of host bryophytes. The marked loss of bryophytes growing in rice fields, *Riccia*,*Phaeoceros*,*Notothylas*, and *Anthoceros* spp., due to farmland consolidation, overuse of herbicides and insecticides, and abandonment of rice cultivation, is now threatening the agromyzid species associated with these bryophytes.

### ﻿Morphological differentiation

The liverwort-associated *Phytoliriomyza* was strongly characterized by the armaments of the specialized comb of fused tubercle-like setae, or unusually elongated or modified tubercle-like setae, on the inner surface of the epandrium; the number and arrangement of these tubercle-like setae tended to vary particularly among related species. In the *dorsata* group, for example, four closely related *Phytoliriomyza* species (*P.luna*,*P.izayoi*,*P.chichibuensis*, and *P.caliginosa*) are associated with *Conocephalum*; they differed in the armaments of tubercle-like setae, especially the number of tubercle-like setae in a comb and the position of the long tubercle-like seta on the inner-lateral surface of the epandrium (Figs 37I, 40J, 42J, and 43I, respectively). Some members in *dorsata* group are associated with *Reboulia*,*Plagiochasma*, and *Asterella*, eight *Phytoliriomyza* species (*P.arcus*, *P.plagiochasmatos*, *P.calcicola*, *P.longifurcae*, *P.falcata*, and *P.aratriformis*) and they had various armaments of uniquely elongated/curved arms on the inner surface of the epandrium (Figs 14K, 17I, 18G, 27H, 29L, and 32H, respectively). The variations in the armaments of tubercle-like setae on the male epandrium may contribute to reproductive isolation.

In addition to male genitalia, these *Phytoliriomyza* species on the same host bryophytes can often be discriminated by the combined color patterns of the following external body parts: antenna, maxillary pulp, haltere, scutum, scutellum, and legs (Table 3). These results suggest that the difference of these color patterns may also contribute to pre-mating reproductive isolation of related species.

## Supplementary Material

XML Treatment for Phytoliriomyza

XML Treatment for Phytoliriomyza dorsata

XML Treatment for Phytoliriomyza igniculus

XML Treatment for Phytoliriomyza tsukuyomi

XML Treatment for Phytoliriomyza marchantiae

XML Treatment for Phytoliriomyza nubatama

XML Treatment for Phytoliriomyza dumortierae

XML Treatment for Phytoliriomyza crepusculum

XML Treatment for Phytoliriomyza arcus

XML Treatment for Phytoliriomyza plagiochasmatos

XML Treatment for Phytoliriomyza calcicola

XML Treatment for Phytoliriomyza iriomotensis

XML Treatment for Phytoliriomyza cometiformis

XML Treatment for Phytoliriomyza argentifasciata

XML Treatment for Phytoliriomyza longifurcae

XML Treatment for Phytoliriomyza falcata

XML Treatment for Phytoliriomyza aratriformis

XML Treatment for Phytoliriomyza rebouliae

XML Treatment for Phytoliriomyza wiesnerellae

XML Treatment for Phytoliriomyza luna

XML Treatment for Phytoliriomyza izayoi

XML Treatment for Phytoliriomyza chichibuensis

XML Treatment for Phytoliriomyza caliginosa

XML Treatment for Phytoliriomyza ugetsu

XML Treatment for Phytoliriomyza nigroflava

XML Treatment for Phytoliriomyza brunofasciata

XML Treatment for Phytoliriomyza pallidofasciata

XML Treatment for Phytoliriomyza luteola

XML Treatment for Phytoliriomyza helva

XML Treatment for Phytoliriomyza bifasciata

XML Treatment for Phytoliriomyza alpicola

XML Treatment for Phytoliriomyza lanternaria

XML Treatment for Phytoliriomyza conocephali

XML Treatment for Phytoliriomyza suetsugui

XML Treatment for Phytoliriomyza ricciae

XML Treatment for Phytoliriomyza sexfasciata

XML Treatment for Phytoliriomyza caerulescens

XML Treatment for Phytoliriomyza foliocerotis

XML Treatment for Phytoliriomyza megacerotis

XML Treatment for Phytoliriomyza phaeocerotis

## References

[B1] Asakawa Y Ludwiczuk A (2017) Chemical constituents of bryophytes: structures and biological activity. Journal of Natural Products 81: 641–660. 10.1021/acs.jnatprod.6b01046 29019405

[B2] Becerra JX Noge K Venable, DL (2009) Macroevolutionary chemical escalation in an ancient plant–herbivore arms race. Proceedings of the National Academy of Sciences 106: 18062–18066. 10.1073/pnas.0904456106 PMC2775328 19706441

[B3] Becker T (1908) Dipteren der Kanarischen Inseln. Zoologischen Museum in Berlin. Berlin, 180 pp.

[B4] Černý M von Tschirnhaus M Winqvist K (2020) First records of Palaearctic Agromyzidae (Diptera) from 40 countries and major islands. Acta Musei Silesiae. Scientiae Naturales 69 (3): 193–229. 10.2478/cszma-2020-0017

[B5] d’Aguilar J (1944) Description d’un Liriomyza nouveau vivant sur Riccianatans L. Bulletin de la Société Entomologique de France 84: 143–146. 10.3406/bsef.1979.21700

[B6] Ding Q Labandeira CC (2014) Biology of a leaf miner (Coleoptera) on *Liaoningocladusboii* (Coniferales) from the Early Cretaceous of northeastern China and the leaf-mining biology of possible insect culprit clades. Arthropod Systematics & Phylogeny 72: 281–308.

[B7] Doorenweerd C Van Nieukerken EJ Sohn JC Labandeira CC (2015) A revised checklist of Nepticulidae fossils (Lepidoptera) refers to an Early Cretaceous origin. Zootaxa 3963 (3): 295–334. 10.11646/zootaxa.3963.3.2 26249403

[B8] Frick KE (1952) A generic revision of the family Agromyzidae (Diptera) with a catalogue of New World species. University of California Publications in Entomology 8: 339–452.

[B9] Frick KE (1959) Synopsis of the species of agromyzid leaf miners described from North America (Diptera). Proceedings of the United States National Museum 108 (3407): 347–465. 10.5479/si.00963801.108-3407.347

[B10] Furuki T (2000) *Riccialamellosa*, Raddi newly found in Japan. Biological Research 7: 314–316.

[B11] Hendel F (1931) Agromyzidae. Die Fliegen der palaearktischen Region 52: 1–256.

[B12] Hering EM (1951) Biology of the leaf miners. Springer Science & Business Media, 293 pp. 10.1007/978-94-015-7196-8

[B13] Hering EM (1957) Minierfliegen in Lebermoosen (Dipt. Agromyzidae). Mitteilungen der Deutschen Entomologischen Gesellschaft 16: 48–51. 10.1002/mmnd.4820160311

[B14] Hering EM (1966) Minierfliegen in Lebermoosen II* (Dipt. Agromyzidae). Deutsche Entomologische Zeitschrift 13: 231–236.

[B15] Hofmeister WFB (1862) On the germination, development, and fructification of the higher Cryptogamia: and on the fructification of the Coniferae. Robert Hardwicke, London, 506 pp. 10.5962/bhl.title.23191

[B16] Imada Y Kato M (2016a) Bryophyte-feeding of *Litoleptis* (Diptera: Rhagionidae) with descriptions of new species from Japan. Zootaxa 4097 (1): 41–58. 10.11646/zootaxa.4097.1.2 27394524

[B17] Imada Y Kato M (2016b) Bryophyte-feeders in a basal brachyceran lineage (Diptera: Rhagionidae: Spaniinae): adult oviposition behavior and changes in the larval mouthpart morphology accompanied with the diet shifts. PLoS ONE 11(11): e0165808. 10.1371/journal.pone.0165808 PMC5094795 27812169

[B18] Imada Y Oyama N Shinoda K Takahashi H Yukawa H (2022) Oldest leaf mine trace fossil from East Asia provides insight into ancient nutritional flow in a plant-herbivore interaction. Scientific Reports 12 (1): 5254. 10.1038/s41598-022-09262-1 35347200 PMC8960907

[B19] Labandeira CC (2006) Silurian to Triassic plant and hexapod clades and their associations: New data, a review, and interpretations. Arthropod Systematics & Phylogeny 64: 53–94.

[B20] Lonsdale O (2021) Manual of North American Agromyzidae (Diptera, Schizophora), with revision of the fauna of the “Delmarva” states. ZooKeys 1051: 1–481. 10.3897/zookeys.1051.64603 34393548 PMC8342412

[B21] Lonsdale O von Tschirnhaus M (2021) Agromyzidae. In: Kirk-Spriggs AH, Sinclair BJ (Eds) Manual of Afrotropical Diptera 3, Silverton, 1913–1938.

[B22] Maccracken SA Sohn J-C Miller IM Labandeira CC (2021) A new Late Cretaceous leaf mine *Leucopteropsaspiralae* gen. et sp. nov. (Lepidoptera: Lyonetiidae) represents the first confirmed fossil evidence of the Cemiostominae. Journal of Systematic Palaeontology 19 (2): 131–144. 10.1080/14772019.2021.1881177

[B23] Ministry of the Environment Japan (2015) Red list of bryophytes. Ministry of the Environment, Japan, Tokyo, 1–4. https://www.env.go.jp/press/files/jp/28076.pdf

[B24] Nowakowski JT (1962) Introduction to a systematic revision of the family Agromyzidae (Diptera) with some remarks on host plant selection by these flies. Annales Zoologici Polska Akademia Nauk. Instytut Zoologiczny 20: 67–183.

[B25] Ohgue T Imada Y Sato AAW Salazar JRL Kato M (2018) The first insect-induced galls in bryophytes. Bryophyte Diversity and Evolution 40 (1): 1–5. 10.11646/bde.40.1.1

[B26] Ólafsson E (1988) A new agromyzid from Iceland: *Phytoliriomyzaislandica* sp. n. (Diptera: Agromyzidae). Insect Systematics & Evolution 19 (3): 359–361. 10.1163/187631289X00249

[B27] Papp L (1984) Family Agromyzidae. In: Soós A (Ed.) Catalogue of Palaearctic Diptera 9.Elsevier, Amsterdam, 263–343.

[B28] Papp L Černý M (2017) Agromyzidae (Diptera) of Hungary (Vol. 3). Phytomyzinae II. Pars Ltd., Hungary. Nagykovácsi, 427 pp. 10.18655/Agromyzidae.Vol.3

[B29] Puttick MN Morris JL Williams TA Cox CJ Edwards D Kenrick P Pressel S Wellman CH Schneider H Pisani D Donoghue PCJ (2018) The interrelationships of land plants and the nature of the ancestral embryophyte. Current Biology 28 (5): 733–745. 10.1016/j.cub.2018.01.063 29456145

[B30] Sasakawa M (2008) Agromyzidae (Insecta: Diptera) from the Alishan Mountains, Taiwan, with descriptions of five new species. Species Diversity 13(2–3): 133–148. 10.12782/specdiv.13.133

[B31] Scheffer SJ Winkler IS Wiegmann BM (2007) Phylogenetic relationships within the leaf-mining Xies (Diptera: Agromyzidae) inferred from sequence data from multiple genes. Molecular Phylogenetics and Evolution 42 (3): 765–775. 10.1016/j.ympev.2006.12.018 17291785

[B32] Sellier R (1947) Contribution a l’étude de *Liriomyzamesnili* d’Aguilar (Diptere Agromyzidae). Annales des sciences naturelles Zoologie 11 ser. 9: 27–38.

[B33] Spencer KA (1963) Notes on the Agromyzidae (Diptera) of Madagascar 1. Proceedings of the Royal Entomological Society of London (B) 32(7–8): 114–116. 10.1111/j.1365-3113.1963.tb01674.x

[B34] Spencer KA (1965) Agromyzidae. Diptera from Nepal. Bulletin of the British Museum (Natural History), Entomology 16: 25–31. 10.5962/bhl.part.21862

[B35] Spencer KA (1969) The Agromyzidae of Canada and Alaska. Memoirs of the Entomological Society of Canada 64: 1–311. 10.4039/entm10164fv

[B36] Spencer KA (1971) Notes on a revision of the British Agromyzidae (Diptera) including the description of 14 new species. Entomologist’s Gazette 22: 141–195.

[B37] Spencer KA (1972) . Agromyzidae. Handbooks for the identification of British insects. Diptera: 10. 5 g, Royal Entomological Society, London, 140 pp.

[B38] Spencer KA (1973) The Agromyzidae of Venezuela. Revista de la Facultad de Agronomía 7: 5–107.

[B39] Spencer KA (1976) The Agromyzidae (Diptera) of Fennoscandia and Denmark. Fauna Entomologica Scandinavica 5: 1–606.

[B40] Spencer KA (1990) Host specialization in the world Agromyzidae (Diptera). Series Entomologica 45. Kluwer Academic Publishers, Dordrecht, 444 pp. 10.1007/978-94-009-1874-0

[B41] Spencer KA Stegmaier Jr CE (1973) The Agromyzidae of Florida with a supplement on Species from the Caribbean. Arthropods of Florida 7. Florida Department of Agriculture Consumer Services, Gainesville, 205 pp.

[B42] Spencer KA Steyskal GC (1986) Manual of the Agromyzidae (Diptera) of the United States. Agriculture Handbook (638): 1–478. 10.5962/bhl.title.119606

[B43] Strobl G (1898) Die Dipteren von Steiermark. IV. Mitteilungendes Naturwissonschaftiichen Vereinsfilr Steiermark 34: 192–298.

[B44] von Tschirnhaus M (1971) Unbekannte Stridulationsorgane bei Dipteren und ihre Bedeutung für Taxonomie und Phylogenetik der Agromyziden. Beiträge zur Entomologie 21: 551–579.

[B45] Winkler IS Mitter C Scheffer SJ (2009) Repeated climate-linked host shifts have promoted diversification in a temperate clade of leaf-mining flies. Proceedings of the National Academy of Sciences of the United States of America 106 (43): 18103–18108. 10.1073/pnas.0904852106&nbsp; 19805134 PMC2775340

[B46] Zlobin VV (2005) Studies on European species of the genus Phytoliriomyza Hendel (Diptera: Agromyzidae). Russian Entomological Journal 14: 119–123.

